# Unlocking the Alkynyl Bromide Radical Anion With Alternating Current for Inverse Sonogashira C─H Coupling

**DOI:** 10.1002/advs.76881

**Published:** 2026-09-16

**Authors:** Junya Wang, Xiaozhou Wang, Pui Ying Choy, Chi‐Wai Cheung, Li‐Wen Xu, Hung Kay Lee, Gui‐Juan Cheng, Fuk Yee Kwong

**Affiliations:** ^1^ Department of Chemistry and State Key Laboratory of Synthetic Chemistry The Chinese University of Hong Kong Shatin Hong Kong China; ^2^ Warshel Institute for Computational Biology School of Medicine The Chinese University of Hong Kong, Shenzhen Guangdong China; ^3^ Key Laboratory of Organosilicon Chemistry and Material Technology of of Education Key Laboratory of Organosilicon Material Technology of Zhejiang Province Hangzhou Normal University Hangzhou China

**Keywords:** catalysis, C─H bond functionalization, electrochemistry, inverse Sonogashira, radical coupling

## Abstract

Carbon–bromine bond cleavage is a fundamental step in many chemical transformations. Over the past two decades, the reductive cleavage of C_(Ar)_─Br bonds (via a stepwise pathway) and C_(alkyl)_─Br bonds (via a concerted pathway) has been extensively studied. In contrast, the cleavage of the C_(alkynyl)_─Br bond remains unreported. We hypothesized that this bond may follow a distinct dissociation pathway, diverging from the established mechanisms. Indeed, the possible generation of reactive alkynyl bromide radical anion intermediates could significantly enrich the synthetic toolbox. However, chemical additives are less attractive than an electrochemical approach. Herein, we report an alternating current (AC)‐promoted, Pd‐catalyzed *ortho*‐aryl C─H alkynylation employing unprecedented alkynyl bromide radical anion species. This “inverse” Sonogashira synthetic scheme accommodates a broad substrate scope, delivering alkynylated stilbene derivatives with excellent arene site selectivity. Mechanistic investigations, EPR spectroscopy, and DFT calculations suggest the involvement of a Pd(II)/Pd(III) pathway assisted by transient alkynyl bromide radical anion intermediates. This work opens a new method for bromoalkyne activation, demonstrates the capability of electrochemistry to enable efficient electrophilic alkynylation, and expands the synthetic toolkit for constructing complex stilbene‐based frameworks.

## Graphical abstract

An alternating current (AC)‐promoted, Pd‐catalyzed *ortho*‐aryl C─H alkynylation of 2,3‐diaryl acrylaldehydes is described. This synthetic scheme accommodates a broad substrate scope, delivering alkynylated stilbene derivatives with excellent arene site selectivity. Mechanistic investigations, including control experiments, EPR spectroscopy and DFT calculations, suggest the involvement of a Pd(II)/Pd(III) pathway assisted by transient alkynyl bromide radical anion intermediates.

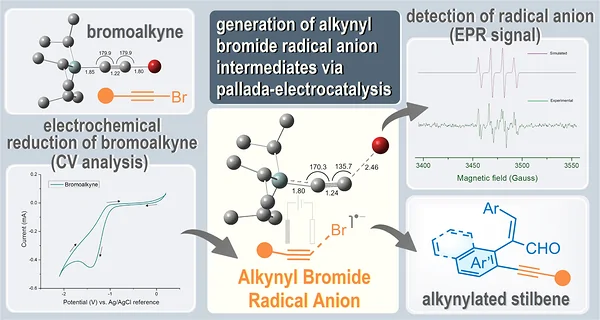

## 1 Introduction

**SCHEME 1:**
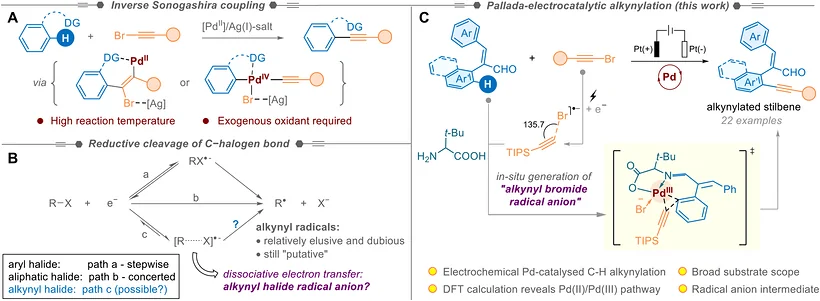
Background and prior work done of inverse Sonogashira coupling.

Alkynes represent one of the most versatile classes of building blocks in organic chemistry owing to their readily modifiable carbon–carbon triple bond and exceptional inherent reactivity. These distinctive features enable a wide range of chemical transformations, rendering alkynes invaluable in the synthesis of pharmaceuticals, organic materials, and complex natural products [1, 2, 3, 4]. Consequently, the development of robust methods for installing alkyne moieties onto organic frameworks is often highly desirable in synthetic methodology. In this context, the “inverse” Sonogashira coupling [5, 6] has emerged as a complementary approach to the classical nucleophilic Sonogashira coupling (Scheme 1A) [7, 8, 9, 10, 11]. This protocol essentially flips the conventional reactivity paradigm by using haloalkynes as the electrophilic component [12, 13]. Recent investigations suggested that the directing group‐assisted activation of an inert C─H bond in the “inverse Sonogashira coupling” generally proceeds via a Pd(II)/(IV) cycle or a migratory insertion pathway [14, 15]. However, these transformations often require demanding reaction conditions, such as elevated temperatures or stoichiometric Ag(I) additives, to facilitate halide abstraction and catalyst oxidation. To circumvent these drawbacks, the development of an efficient and sustainable electrochemical protocol is highly desirable.

Recent advances have shown that merging transition metal‐catalyzed C─H functionalization with electrochemical oxidation represents a promising paradigm in catalysis [16, 17, 18, 19, 20, 21, 22, 23, 24, 25, 26, 27, 28, 29, 30, 31]. This synergistic approach provides precise control over metal oxidation states at the anode and facilitates efficient catalyst regeneration at the cathode. Within this framework, the cathode serves as a sustainable electron source for the one‐electron reductive cleavage of organohalides to generate carbon‐centered radicals and halide anions for subsequent transformations [32, 33, 34]. Carbon‐centered radicals [35, 36], particularly alkyl [37, 38, 39], alkenyl [40], allenyl [41, 42], and aryl radicals [43, 44, 45, 46, 47], have been extensively studied, whereas alkynyl radicals have remained relatively elusive in the scientific literature [48]. Generally, the reductive cleavage follows one of two pathways (Scheme 1B): a stepwise sequence, where electron transfer leads to a transient radical anion intermediate prior to C─X bond dissociation (path a: often observed with aryl halides); or a concerted process, in which electron transfer and bond breaking occur in a single elementary step (path b: mostly for aliphatic halides) [49, 50, 51, 52]. Furthermore, a refined model of the concerted pathway, known as “sticky” dissociative electron transfer (DET), has been proposed to account for ion‐dipole interactions between the two fragments, which can significantly influence the dynamics of electron transfer preceding the cleavage (path c) [32, 33, 34, 53, 54, 55]. Notably, both the alkynyl radical and the alkynyl bromine radical anion are expected to be favorable for the transformation to the Pd(II) complex due to their lower reaction energy barriers than bromoalkyne itself. Despite these insights, investigations into the reductive cleavage and radical/radical anion behavior of alkynyl halides remain unexplored, and successful examples of pallada‐electrochemical C─H alkynylation remain scarce [56, 57, 58], representing a significant knowledge gap in the field. Motivated by the untapped potential of radical/radical anions of alkynyl halides and our prior work in alkyne chemistry [59, 60, 61, 62, 63, 64], we herein report a synergistic pallada‐electrochemical system for site‐selective, direct C─H alkynylation of acrylaldehydes. This methodology utilizes an electrochemical cell in which the cathode drives the one‐electron reduction cleavage of bromoalkyne, while the anode facilitates the efficient oxidation of [Pd] catalyst (Scheme 1C).

## 2 Results and Discussion

We initiated our investigation by employing (*E*)‐2‐(naphthalen‐1‐yl)‐3‐phenylacrylaldehyde (**1a**) and (bromoethynyl)triisopropylsilane (**2a**) as model substrates to probe the effective pallada‐electrochemical conditions (Table 1). Since alternating current (AC) can significantly enhance reaction efficiency [65, 66, 67], the prototype reaction was conducted in an undivided cell fitted with two Pt electrodes under a controlled square‐waveform AC (voltage range: 1.9–2.3 V; 0.6 mA; 1/80 Hz) (entry 1). Under these conditions, the desired product was afforded in 68% yield. It is worth noting that this alkynylation exhibited exclusive regioselectivity at the *β*‐position of the naphthalene ring, while the C(sp^2^)─H bonds located at the 3‐phenyl and alkenyl groups remained untouched. Better performance was observed for AC mode compared to direct current (DC), highlighting the ability of AC to suppress the cathodic precipitation of metal catalyst complexes (entry 1 vs. 2) [57, 68, 69]. When DC or AC at either higher or lower frequencies was applied, lower yields of **3aa** were obtained (entry 1 vs. entries 3–4). Various transient directing groups [70, 71, 72, 73, 74, 75, 76] were tested, and *D, L‐tert‐*leucine gave the best product yields (entry 1 vs. entries 8–9). Varying other reaction parameters, such as Pd catalyst, solvent, electrolyte, base, and temperatures led to diminished yields (entry 1 vs. entries 6–16). Control experiments revealed the essential roles of Pd(TFA)_2_, *D, L‐tert‐*leucine and pyridone [77, 78, 79], as the absence of any one component completely suppressed the product formation (entries 7, 10 and 17). Notably, when iodoalkyne was used instead of bromoalkyne, only trace product was detected (entry 18).

**TABLE 1:** Optimization of reaction conditions^a^.

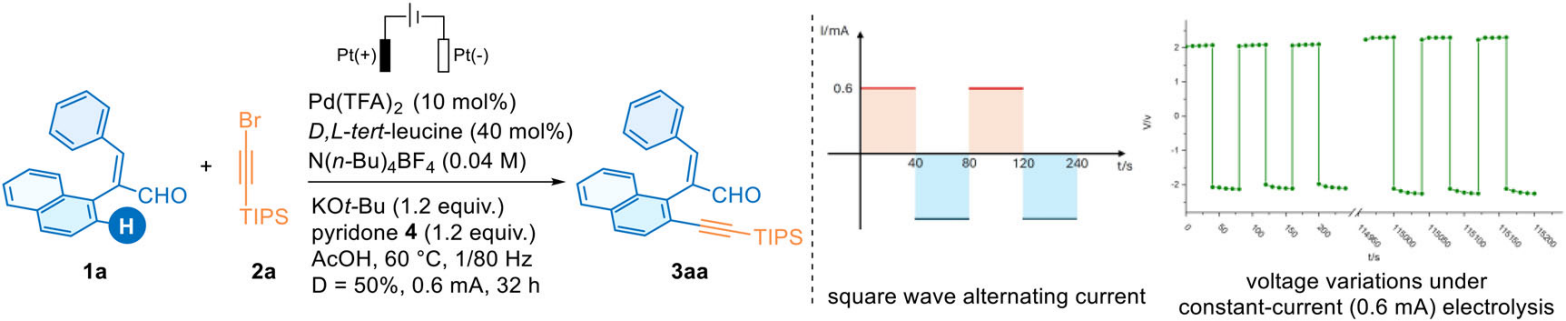		
Entry	Variation From Standard Conditions	Yield [%]^b^
1	none	72 (68)^c^
2	DC instead of AC	44
3	1/40 Hz instead of 1/80 Hz	38
4	1/120 Hz instead of 1/80 Hz	65
5	no electric current	26 (23)^c^
6	Pd(OAc)_2_ instead of Pd(TFA)_2_	57
7	no Pd(TFA)_2_	n.r.^d^
8	*D, L*‐Valine instead of *D, L*‐*tert*‐leucine	47
9	*L*‐*tert*‐leucine instead of *D, L*‐*tert*‐leucine	70
10	no *D, L*‐*tert*‐leucine	n.r.
11^e^	HFIP instead of AcOH	<10
12^f^	TFE instead of AcOH	<10
13	N(*n*‐Bu)_4_OAc instead N(*n*‐Bu)_4_BF_4_	33
14	KOAc instead of KO*t*‐Bu	55
15	KH_2_PO_4_ instead of KO*t*‐Bu	38
16	r.t. instead of 60°C; 0.3 mA instead of 0.6 mA	30
17^g^	no pyridone **4**	60
18	iodoalkyne instead bromoalkyne	trace

^a^ Reaction conditions: 2,3‐Diaryl acrylaldehyde **1a** (0.2 mmol), (bromoethynyl)triisopropylsilane (**2a**) (0.4 mmol), Pd(TFA)_2_ (10 mol%), *D, L‐tert*‐leucine (40 mol%), N(*n*‐Bu)_4_BF_4_ (0.04 M), pyridone **4** (1.2 equiv.), and AcOH (5 mL) were stirred in an undivided cell with two Pt electrodes at 60°C under air atmosphere for 32 h under constant current mode. D = Duty ratio

^b^ Calibrated GC yields were reported using tetradecane as the internal standard

^c^ Isolated yields

^d^ n.r. = no reaction

^e^ HFIP = 1,1,1,3,3,3‐hexafluoro‐2‐propanol

^f^ TFE = 2,2,2‐trifluoroethanol

^g^ pyridone **4** = 5‐chloro‐3‐nitropyridin‐2(1*H*)‐one.

**SCHEME 2:**
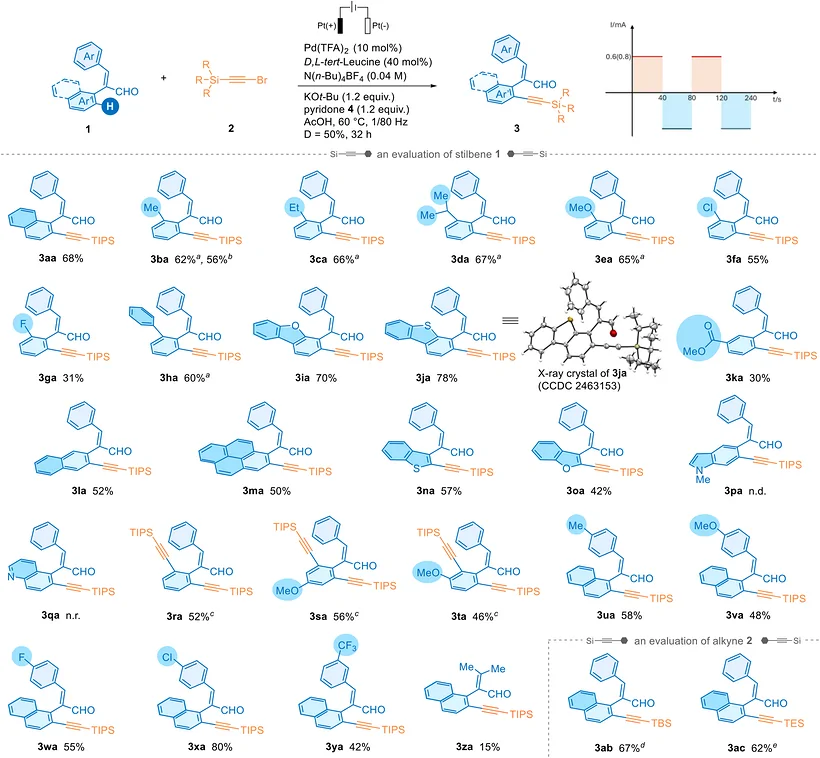
Substrate Scope. Reaction conditions: Stilbene **1** (0.2 mmol), alkyne **2** (0.4 mmol), Pd(TFA)_2_ (10 mol%), *D, L‐tert*‐Leucine (40 mol%), N(*n*‐Bu)_4_BF_4_ (0.04 M), KO*t*‐Bu (0.24 mmol), pyridone **4** (0.24 mmol), AcOH (5 mL) were stirred in an undivided cell with two Pt electrodes at 60°C under air atmosphere for 32 h using alternating current (0.6 mA, 1/80 Hz) under constant current(cc) mode. Isolated yields were reported. n.d. = not determined. n.r. = no reaction. ^a^Stilbene **1** (0.25 mmol), alkyne **2** (0.5 mmol), Pd(TFA)_2_ (10 mol%), *D, L‐tert*‐Leucine (40 mol%), N(*n*‐Bu)_4_BF_4_ (0.04 M), KO*t*‐Bu (0.3 mmol), pyridone **4** (0.3 mmol), AcOH (5 mL) were stirred in an undivided cell with two Pt electrodes at 60°C under air atmosphere for 32 h using alternating current (0.8 mA, 1/80 Hz) under constant current(cc) mode. ^b^Gram‐scale synthesis. **1b** (5.0 mmol), **2a** (10.0 mmol), AC (2.0 mA, 1/80 Hz) were used, and the reaction was stirred for 7 days. ^c^Alkyne **2** (1.0 mmol, 4.0 equiv.) was used. ^d^TBS = (*tert*‐Butyl)dimethylsilyl. ^e^TES = Triethylsilyl.

With the optimized conditions in hand, we next investigated the substrate scope (Scheme 2). Diaryl acrylaldehydes having *ortho*‐substituted aryl groups underwent the alkynylation smoothly to furnish the desired products in good yields (i.e., methyl – **3ba**; ethyl – **3ca**; isopropyl – **3** **da**; methoxy – **3ea**; chloro – **3fa**; phenyl – **3** **ha**). A gram‐scale synthesis of **3ba** (1.13 g) was attempted with higher current (2.0 mA). Substrate **1** featuring α‐substituted polyaromatic groups, e.g., 2‐naphthyl and pyrenyl units, were applicable for the selective alkynylation at the vicinal C─H bond, affording alkynylated **3la** and **3ma**, respectively. Ester‐containing stilbene was a feasible substrate to afford the desired product **3ka**. Moreover, substrates bearing heteroaromatic motifs such as benzofuran and benzothiophene were well‐tolerated, delivering alkynylated products **3ia**, **3ja**, **3na** and **3oa** in good yields. The structure of product **3ja** was confirmed by X‐ray crystallographic analysis (CCDC 2463153, see SI for details). Furthermore, di‐alkynylation was successfully accomplished using non‐*ortho*‐substituted stilbene as the substrate (products **3ra**, **3sa**, and **3ta**). Acrylaldehydes with substituents at the *β*‐aromatic ring were also found compatible, leading to the corresponding alkynylated analogues (i.e., methyl – **3** **ua**; methoxy – **3va**; fluoro – **3wa**; chloro – **3xa**; trifluoromethyl – **3ya**) with high selectivity. It is noteworthy that the chloro group remained intact under the stated reaction conditions, in which it is highly beneficial for further transformation through established cross‐coupling strategies [80, 81, 82]. Alkyl‐substituted stilbene substrates afforded the corresponding product **3za** with low yield. When the triisopropylsilyl (TIPS) group of **2a** was replaced by (*tert*‐butyl)dimethylsilyl (TBS) or triethylsilyl (TES) substituent, the corresponding silylethynyl‐functionalized products **3ab** and **3ac** were also well afforded.

**SCHEME 3:**
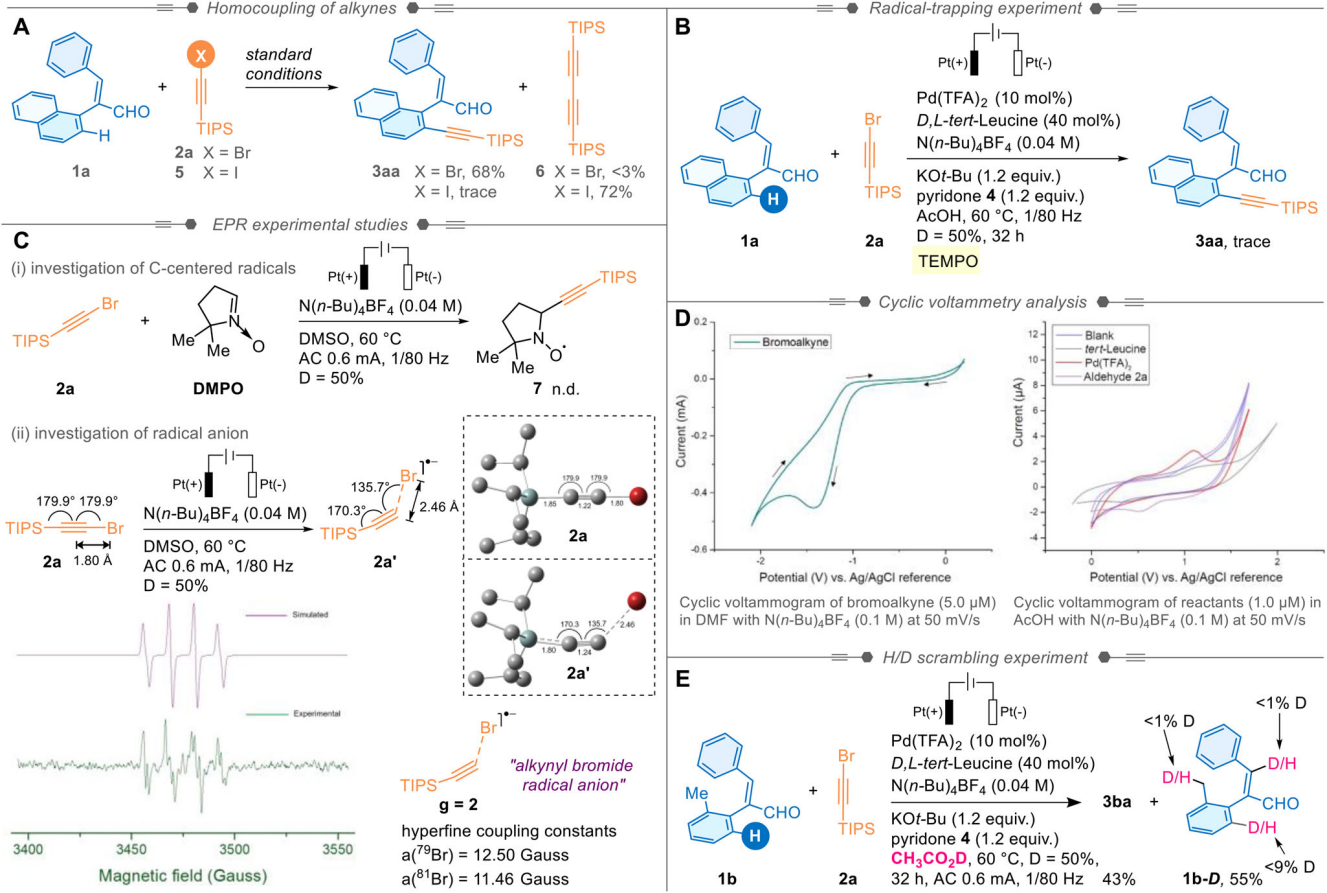
Control experiments, cyclic voltammetry (CV) analysis and electron paramagnetic resonance (EPR) studies. All standard conditions are the same as in Scheme 2.

To elucidate the mechanism, a series of control experiments were carried out. Replacing bromoalkynes **2a** with iodoalkynes **5** resulted in only a trace amount of desired alkynylated product, whereas homocoupled diyne **6** was obtained in 72% yield (Scheme 3A). In contrast, bromoalkynes afforded less than 3% diyne. This suggests that the generation of free alkynyl radicals from bromoalkyne **2a** is much less favorable due to a higher kinetic barrier. A radical‐trapping experiment with TEMPO completely inhibited the alkynylation (Scheme 3B), implicating the involvement of a radical‐like intermediate, which was proposed to be the alkynyl bromide radical anion **2a’**. Furthermore, complex and undecipherable signals were observed in an electron paramagnetic resonance (EPR) spin‐trapping experiment with 5,5‐dimethyl‐1‐pyrroline‐*N*‐oxide (DMPO) (Scheme 3C‐i), while radical signal peaks derived from the proposed alkynyl bromide anion **2a’** were detected (Scheme 3C‐ii), compellingly indicating the one‐electron reduction of bromoalkyne **2a** under AC conditions. Density functional theory (DFT) calculations further revealed that one‐electron reduction of the bromoalkyne **2a** leads to barrierless dissociation of the C(sp)─Br bond to form a loosely associated radical anion [C(sp)···Br]•^−^ [53, 54, 55]. Additionally, the optimized structure indicates that the alkynyl bromide anion **2a’** is bent, with a C(sp)–C(sp)···Br angle of 135.7° compared to the linear structure of **2a** (179.9°), accompanied by an elongation of the C(sp)···Br bond from 1.80 to 2.46 Å. While these structural distortions enhance the reactivity toward reductive cleavage of the C(sp)–Br bond, subsequent dissociation of this radical anion intermediate to release the free alkynyl radical is thermodynamically disfavoured due to the high energy and extremely short lifetime of the corresponding alkynyl radicals [48, 83, 84, 85, 86, 87], consequently accounting for the absence of homocoupling products. Complementary cyclic voltammetry (CV) analysis was performed to characterize the electrochemical behavior of the system (Scheme 3D). A reduction peak at −1.4 V (vs. aqueous Ag/AgCl) was assigned to the reduction of bromoalkyne to its radical anion, while an oxidation peak at +1.1 V (vs. aqueous Ag/AgCl) corresponds to the oxidation of low‐valent palladium species to the active Pd(II) intermediate [88]. This suggests the regeneration of the active Pd(II) species after each catalytic cycle. Finally, a H/D scrambling experiment was conducted using acetic acid‐*d* (CH_3_CO_2_D) as the solvent, and no H/D scrambling was observed (Scheme 3E‡).

**SCHEME 4:**
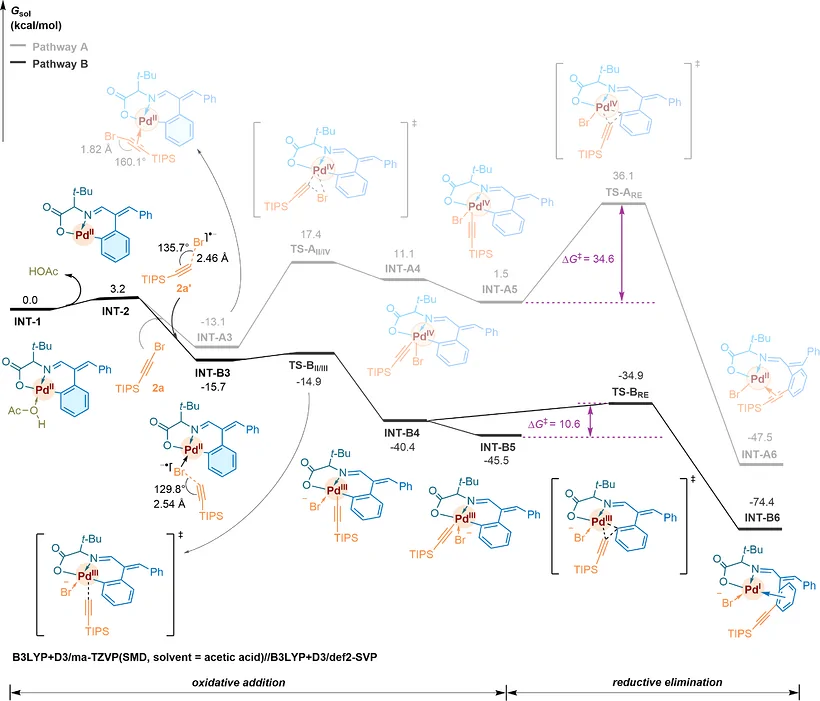
Reaction energy profile for electrochemical palladium‐catalyzed alkynylation of acryladehydes.

As depicted in Scheme 4 for Pd(II)/Pd(IV) pathway (Pathway A), **INT‐2** reacts with bromoalkyne **2a** to form Pd(IV) intermediate **INT‐A5** via a transition state **TS‐A_II/IV_** in the absence of current. Reductive elimination via transition state **TS‐A_RE_** furnishes **INT‐A6**, which is a direct precursor to alkynylated product **3aa** upon hydrolysis. However, this pathway involves high‐energy transition states (**Δ*G* ^‡^_TS‐AII/IV_** = 30.5 kcal/mol; **Δ*G* ^‡^_TS‐ARE_** = 34.6 kcal/mol), suggesting the Pd(II) center in **INT‐2** lacks sufficient electron density to facilitate oxidative addition. Thus, the Pd(II)/Pd(IV) route appears to be energetically unfavorable. For Pd(II)/Pd(III) pathway (Scheme 4, Pathway B), an electrochemical reduction of bromoalkyne generates alkynyl bromide radical anion **2a’**, which interacts with Pd(II)‐palladacycle **INT‐2** to form **INT‐B3**. The alkynyl bromide radical anion undergoes single‐electron oxidative addition via a transition state **TS‐B_II/III_** to afford the Pd(III) species **INT‐B4**, which subsequently undertakes reductive elimination to produce Pd(I) intermediate **INT‐B6**, a precursor to alkynylated product **3aa**. Then, the Pd(I) species is reoxidized to Pd(II) via an anodic oxidation. This reaction pathway is both kinetically and thermodynamically favorable, featuring low activation barriers for mutual oxidative addition and reductive elimination steps (**Δ** ***G*** ^**‡**^ = 0.8 kcal/mol; **Δ*G*** **^‡^** = 10.6 kcal/mol).

**SCHEME 5:**
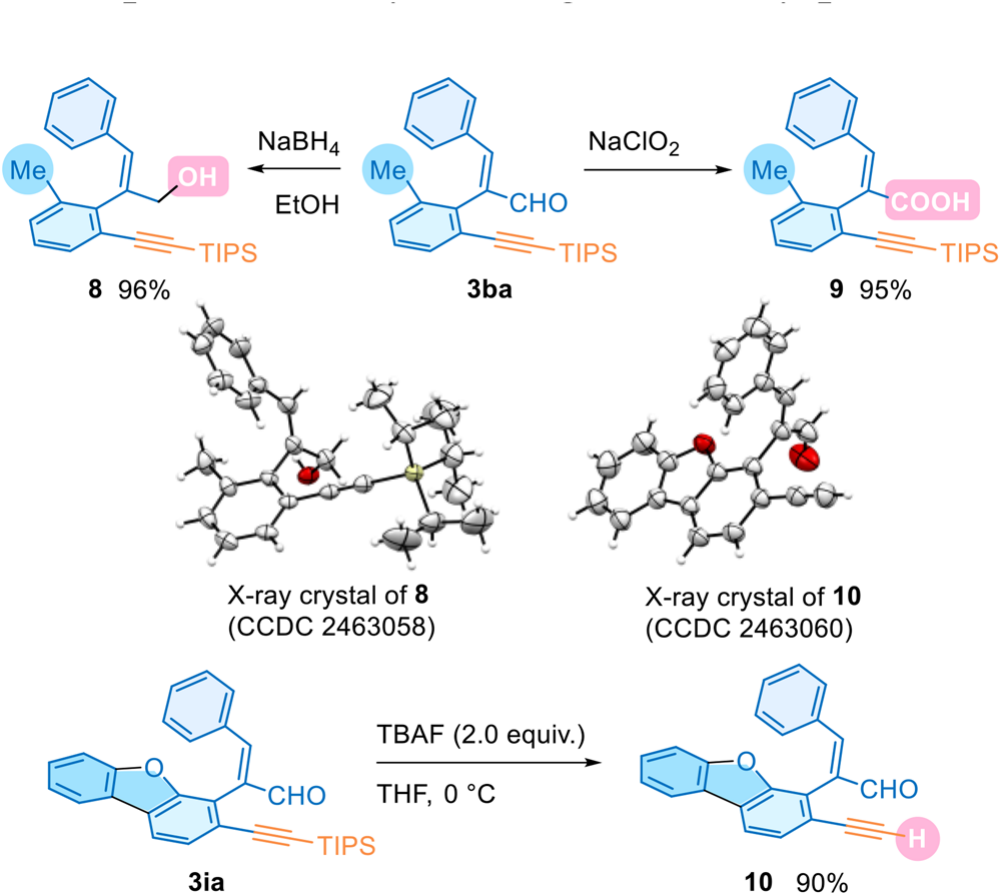
Further transformation of alkynylated diaryl acryladehdyes.

In addition to substrate scope exploration and mechanistic studies, the synthetic utility of this electrochemical alkynylation was investigated (Scheme 5). The alkynylated diaryl acrylaldehydes were readily derivatized through either reduction or oxidation to afford the resulting compounds **8**–**10** in excellent yields. The structures of products **8** and **10** were also unambiguously confirmed by X‐ray crystallographic analysis (CCDC 2463058 and 2463060, see SI for details). These structurally diverse alkynyl stilbenes are versatile scaffolds for further development of a bioactive compound library in the drug discovery process.

## 3 Conclusion

In conclusion, while the nucleophilic Sonogashira cross‐coupling is a well‐established synthetic tool for constructing C(sp)‐C(sp^2^) bonds, the development of “inverse Sonogashira coupling” as a valuable synthetic complement remains in great demand. Herein, we have developed a pallada‐electrocatalytic system for the *ortho*‐aryl C─H alkynylation of 2,3‐diaryl acrylaldehydes. Under modulated AC‐promoted conditions, the reaction proceeds via a transient, unstable alkynyl bromide radical anion intermediate, which demonstrates favorable efficacy in electrophilic alkynylation for delivering alkynylated stilbene frameworks in good‐to‐excellent yields. Notably, this method achieves exclusive C─H site‐selectivity at the aldehyde‐bearing geminal arene. Mechanistic studies, including control experiments, EPR spectroscopy, and DFT calculations, support an intriguing Pd(II)/Pd(III) catalytic cycle involving a key alkynyl bromide radical anion species. We anticipate that this new catalyst system will serve as a highly useful tool for tackling other versatile arene alkynylation in relevance.

## 4 Experimental Section/Methods

### 4.1 General Procedures for Electrochemical Pd‐Catalyzed Alkynylation Reaction

The electrocatalysis reaction was carried out in an undivided cell, with a platinum anode (30 mm × 5 mm × 0.1 mm) and a platinum cathode (30 mm × 5 mm × 0.1 mm). Stilbene **1** (1.0 equiv.), alkynyl bromide **2** (2.0‐4.0 equiv.), Pd(TFA)_2_ (10 mol%), *D, L‐tert‐*Leucine (40 mol%), N(*n*‐Bu)_4_BF_4_ (0.04 M), KO*t*‐Bu (1.2 equiv.) and 5‐chloro‐3‐nitropyridin‐2(1*H*)‐one (**4**) (1.2 equiv.) were placed in a 7 mL cell and dissolved in acetic acid (5 mL). Electrocatalysis was performed at 60°C with a constant current of 0.6 mA under an air atmosphere for 32 h. The reaction was then allowed to cool to room temperature. Ethyl acetate (EtOAc) was added, and the electrodes were washed with EtOAc (3 × 1 mL). The organic layer was collected and filtered through a Celite pad and concentrated in vacuo. The mixture was then purified by flash column chromatography with ethyl acetate and hexane as the elution solvent to give the desired product **3**.

### 4.2 General Procedures for Gram‐Scale Synthesis of Compound **3ba**

The electrocatalysis reaction was carried out in an undivided cell, with a platinum anode (15 mm × 15 mm × 0.1 mm) and a platinum cathode (15 mm × 15 mm × 0.1 mm). Stilbene **1b** (5.0 mmol, 1.0 equiv.), alkynyl bromide **2a** (10.0 mmol, 2.0 equiv.), Pd(TFA)_2_ (0.5 mmol, 10 mol%), *D, L‐tert‐*Leucine (2.0 mmol, 40 mol%), N(*n*‐Bu)_4_BF_4_ (0.8 mmol, 0.04 M), KO*t*‐Bu (6.0 mmol, 1.2 equiv.) and 5‐chloro‐3‐nitropyridin‐2(1*H*)‐one (**4**) (6.0 mmol, 1.2 equiv.) were placed in a cell and dissolved in acetic acid (20 mL). Electrocatalysis was performed at 60°C with a constant current of 2.0 mA under an air atmosphere for 7 days. The reaction was then allowed to cool to room temperature. Ethyl acetate was added, and the electrodes were washed with EtOAc (3 × 1 mL). The organic layer was collected and filtered through a Celite pad and concentrated in vacuo. The mixture was then purified by flash column chromatography with ethyl acetate and hexane as the elution solvent to give the desired product **3ba** in 56% (1.13 g).

### 4.3 General Procedures for Synthesis of Product **8**

Compound **3ba** (2 mmol, 1.0 equiv.) was dissolved in ethanol (EtOH) (2 mL) and dichloromethane (DCM) (2 mL), and then NaBH_4_ (2.0 equiv.) were added at 0 °C. The reaction mixture was stirred at room temperature for 3 h. After the completion of the reaction (monitored by TLC), saturated NH_4_Cl was added, and the crude products were extracted with EtOAc. The organic layers were combined, dried over Na_2_SO_4_, filtered, and the solvent was then evaporated under vacuum. The mixture was purified by flash column chromatography with silica gel (200–300 mesh), using ethyl acetate and hexane (1:10, v/v, R*_f_* = 0.12) as the elution solvent to give the desired product **8** in 96% yield (0.78 g, white solid).

### 4.4 General Procedures for Synthesis of Product **9**

Compound **3ba** (2 mmol, 1.0 equiv.) was dissolved in *t*‐BuOH (1 M), and then a solution of NaH_2_PO_4_ (10 mmol, 5.0 equiv.) and NaClO_2_ (7.4 mmol, 3.7 equiv.) in water (10 mL) were added followed by 2‐methyl‐2‐ butene (18 mmol, 9.0 equiv.). The reaction mixture was stirred at 40 °C for 4 h. After the completion of the reaction (monitored by TLC), saturated NH_4_Cl was added, and the crude products were extracted with EtOAc. The organic layers were combined, dried over Na_2_SO_4_, filtered, and the solvent was then evaporated under vacuum to obtain crude acid. The mixture was purified by flash column chromatography with silica gel (200‐300 mesh), using methanol, ethyl acetate, and hexane (1:5:15, v/v, R*_f_* = 0.12) as the elution solvent to give the desired product **9** in 95% yield (0.79 g, colorless oil).

### 4.5 General Procedures for Synthesis of Product **10**

Compound **3ha**  (0.5 mmol, 1.0 equiv.) was dissolved in dry THF (5 mL), and cooled to 0 °C. TBAF (2.0 M in THF) (1.0 mmol, 2 equiv.) was added to the mixture dropwise. The reaction mixture was stirred at 0 °C for 30 min. After the completion of the reaction (monitored by TLC), the crude products were extracted with EtOAc and brine. The organic layers were combined, dried over Na_2_SO_4_, filtered, and the solvent was then evaporated under vacuum. The mixture was purified by flash column chromatography with silica gel (200‐300 mesh), using ethyl acetate and hexane (1:10, v/v, R*_f_* = 0.55) as the elution solvent to give the desired product **10** in 90% yield (0.145 g).

#### 4.5.1 (*E*)‐3‐Phenyl‐2‐(2‐((triisopropylsilyl)ethynyl)naphthalen‐1‐yl)acrylaldehyde (Scheme 2, Product 3aa)

This compound was prepared according to General Procedures using stilbene **1a** (0.2 mmol, 1.0 equiv.) and alkynyl bromide **2a** (0.4 mmol, 2.0 equiv.) as substrates, and the reaction was performed under constant current 0.6 mA (1/80 Hz. D = 50%). The crude product was purified by column chromatography (EtOAc/Hexane = 1:10, R*_f_* = 0.42) to afford a sticky yellow oil in 68% yield (59.6 mg). **^1^H NMR (500 MHz, CDCl_3_)** δ 9.12 (s, 1H), 7.87 (dd, *J* = 8.0, 3 Hz, 2H), 7.78 (s, 1H), 7.65 (d, *J* = 8.5 Hz, 1H), 7.61 (d, *J* = 8.5 Hz, 1H), 7.48 (t, *J* = 7.5 Hz, 1H), 7.39 (t, *J* = 7.5 Hz, 1H), 7.23 (t, *J* = 7.5 Hz, 1H), 7.14 (t, *J* = 7.5 Hz, 1H), 7.10 (t, *J* = 8.0 Hz, 1H), 1.01 (s, 21H). **^13^C NMR (125 MHz, CDCl_3_)** δ 193.4, 151.6, 139.3, 135.7, 134.0, 133.2, 131.1, 130.7, 129.3, 128.7, 128.6, 128.6, 127.3, 126.9, 125.3, 121.1, 105.7, 95.4, 18.7, 11.3. **HRMS (HESI‐II)**: m/z [M+Na]^+^ calcd for C_30_H_34_NaOSi: 461.2271; found 461.2270.

#### 4.5.2 (*E*)‐2‐(2‐Methyl‐6‐((triisopropylsilyl)ethynyl)phenyl)‐3‐phenylacrylaldehyde (Scheme 2, Product 3ba)

This compound was prepared according to General Procedures using stilbene **1b** (0.25 mmol, 1.0 equiv.) and alkynyl bromide **2a** (0.5 mmol, 2.0 equiv.) as substrates, and the reaction was performed under constant current 0.8 mA (1/80 Hz. D = 50%). The crude product was purified by column chromatography (EtOAc/Hexane = 1:10, R*_f_* = 0.45) to afford a sticky colorless oil in 62% yield (62.4 mg). **^1^H NMR (500 MHz, CDCl_3_)** δ 9.76 (s, 1H), 7.51 (s, 1H), 7.45 (d, *J* = 7.5 Hz, 1H), 7.30‐7.21 (m, 6H), 7.13 (d, *J* = 8.0 Hz, 1H), 2.04 (s, 3H), 0.95 (s, 21H).**^13^C NMR (125 MHz, CDCl_3_)** δ 193.4, 150.6, 140.6, 136.7, 136.6, 134.4, 130.8, 130.6, 130.5, 130.4, 128.8, 128.3, 123.5, 105.4, 93.8, 19.9, 18.7, 11.3. **HRMS (HESI‐II)**: m/z [M+Na]^+^ calcd for C_27_H_34_NaOSi: 425.2271; found 425.2264.

#### 4.5.3 (*E*)‐2‐(2‐Ethyl‐6‐((triisopropylsilyl)ethynyl)phenyl)‐3‐phenylacrylaldehyde (Scheme 2, Product 3ca)

This compound was prepared according to General Procedures using stilbene **1c** (0.25 mmol, 1.0 equiv.) and alkynyl bromide **2a** (0.5 mmol, 2.0 equiv.) as substrates, and the reaction was performed under constant current 0.8 mA(1/80 Hz. D = 50%). The crude product was purified by column chromatography (EtOAc/Hexane = 1:10, R*_f_* = 0.46) to afford a white solid in 66% yield (68.7 mg). **^1^H NMR (500 MHz, CDCl_3_)** δ 9.78 (s, 1H), 7.53 (s, 1H), 7.47 (d, *J* = 7.3 Hz, 1H), 7.34‐7.28 (m, 3H), 7.22 (t, *J* = 7.8 Hz, 2H), 7.14 (d, *J* = 7.6 Hz, 2H), 2.26‐2.29 (m, 2H), 1.00 (t, *J* = 7.5 Hz, 3H), 0.96 (s, 21H). **^13^C NMR (125 MHz, CDCl_3_)** δ 193.7, 150.8, 142.5, 142.4, 136.1, 134.4, 130.9, 130.7, 130.6, 128.9, 128.8, 128.5, 123.5, 105.5, 93.7, 26.6, 18.6, 14.8, 11.3. **HRMS (HESI‐II)**: m/z [M+Na]^+^ calcd for C_28_H_36_NaOSi: 439.2428; found 439.2424. **Melting point** 74.3‐75.2°C.

#### 4.5.4 (*E*)‐2‐(2‐Isopropyl‐6‐((triisopropylsilyl)ethynyl)phenyl)‐3‐phenylacrylaldehyde (Scheme 2, Product 3da)

This compound was prepared according to General Procedures using stilbene **1d** (0.25 mmol, 1.0 equiv.) and alkynyl bromide **2a** (0.5 mmol, 2.0 equiv.) as substrates, and the reaction was performed under constant current 0.8 mA(1/80 Hz. D = 50%). The crude product was purified by column chromatography (EtOAc/Hexane = 1:10, R*_f_* = 0.55) to afford a sticky colorless oil in 67% yield (72.0 mg). **^1^H NMR (500 MHz, CDCl_3_)** δ 9.79 (s, 1H), 7.53 (s, 1H), 7.46 (t, *J* = 4.1 Hz, 1H), 7.36 (d, *J* = 4.1 Hz, 1H), 7.28 (t, *J* = 7.2 Hz, 1H), 7.21 (t, *J* = 7.6 Hz, 2H), 7.12 (d, *J* = 7.9 Hz, 2H), 2.70 (septet, *J* = 6.5 Hz, 1H), 1.12 (d, *J* = 6.5 Hz, 3H), 0.95 (s, 21H), 0.90 (d, *J* = 6.8 Hz, 3H). **^13^C NMR (125 MHz, CDCl_3_)** δ 193.7, 150.9, 147.3, 140.5, 135.3, 134.4, 130.9, 130.7, 130.6, 128.7, 128.7, 126.2, 123.3, 105.6, 93.5, 31.2, 24.1, 23.7, 18.6, 11.3. **HRMS (HESI‐II)**: m/z [M+Na]^+^ calcd for C_29_H_38_NaOSi: 453.2584; found 453.2578.

#### 4.5.5 (*E*)‐2‐(2‐Methoxy‐6‐((triisopropylsilyl)ethynyl)phenyl)‐3‐phenylacrylaldehyde (Scheme 2, Product 3ea)

This compound was prepared according to General Procedures using stilbene **1e** (0.25 mmol, 1.0 equiv.) and alkynyl bromide **2a** (0.5 mmol, 2.0 equiv.) as substrates, and the reaction was performed under constant current 0.8 mA(1/80 Hz. D = 50%). The crude product was purified by column chromatography (EtOAc/Hexane = 1:10, R*_f_* = 0.28) to afford a sticky colorless oil in 65% yield (63.0 mg). **^1^H NMR (500 MHz, CDCl_3_)** δ 9.73 (s, 1H), 7.52 (s, 1H), 7.33 (t, *J* = 8.0 Hz, 1H), 7.30‐ 7.26 (m, 1H), 7.24‐7.19 (m, 5H), 7.96 (dd, *J* = 8.0, 0.6 Hz, 1H), 3.69 (s, 3H), 0.94(s, 21H). **^13^C NMR (125 MHz, CDCl_3_)** δ 193.3, 157.1, 150.7, 137.8, 134.6, 130.4, 130.3, 129.5, 128.6, 126.1, 125.6, 124.7, 111.6, 104.7, 94.2, 56.1, 18.6, 11.3. **HRMS (HESI‐II)**: m/z [M+Na]^+^ calcd for C_27_H_34_NaO_2_Si: 411.2220; found 411.2214.

#### 4.5.6 (*E*)‐2‐(2‐Chloro‐6‐((triisopropylsilyl)ethynyl)phenyl)‐3‐phenylacrylaldehyde (Scheme 2, Product 3fa)

This compound was prepared according to General Procedures using stilbene **1f** (0.25 mmol, 1.0 equiv.) and alkynyl bromide **2a** (0.5 mmol, 2.0 equiv.) as substrates, and the reaction was performed under constant current 0.8 mA(1/80 Hz. D = 50%). The crude product was purified by column chromatography (EtOAc/Hexane = 1:10, R*_f_* = 0.40) to afford a sticky colorless oil in 55% yield (58.0 mg). **^1^H NMR (500 MHz, CDCl_3_)** δ 9.74 (s, 1H), 7.57 (s, 1H), 7.50 (dd, *J* = 7.5, 1.0 Hz, 1H), 7.45 (dd, *J* = 8.0, 1.0 Hz, 1H), 7.33‐7.29 (m, 2H), 7.26‐7.23 (m, 2H), 7.17–7.15 (m, 2H), 0.95 (s, 21H). **^13^C NMR (125 MHz, CDCl_3_)** δ 192.2, 151.3, 138.6, 136.1, 134.0, 133.9, 131.6, 130.8, 130.4, 129.8, 129.4, 128.9, 125.6, 103.9, 95.6, 18.6, 11.2. **HRMS (HESI‐II)**: m/z [M+Na]^+^ calcd for C_26_H_31_ClNaOSi: 445.1725; found 445.1723.

#### 4.5.7 (*E*)‐2‐(2‐Fluoro‐6‐((triisopropylsilyl)ethynyl)phenyl)‐3‐phenylacrylaldehyde (Scheme 2, Product 3ga)

This compound was prepared according to General Procedures using stilbene **1** **g** (0.25 mmol, 1.0 equiv.) and alkynyl bromide **2a** (0.5 mmol, 2.0 equiv.) as substrates, and the reaction was performed under constant current 0.8 mA (1/80 Hz. D = 50%). The crude product was purified by column chromatography (EtOAc/Hexane = 1:10, R*_f_* = 0.40) to afford a sticky colorless oil in 31% yield (31.4 mg). **^1^H NMR (500 MHz, CDCl_3_)** δ 9.75 (s, 1H), 7.60 (s, 1H), 7.40 (dd, *J* = 7.8, 1.1 Hz, 1H), 7.37–7.31 (m, 2H), 7.27‐7.21 (m, 4H), 7.12 (td, *J* = 8.6, 1.1 Hz, 1H), 0.95 (s, 21H). **^13^C NMR (125 MHz, CDCl_3_)** δ 192.4, 159.7 (d, *J_F_* = 245.4 Hz), 152.0, 135.1, 134.0, 130.8, 130.4, 130.0 (d, *J_F_* = 8.9 Hz), 129.0 (d, *J_F_* = 2.9 Hz), 128.8, 125.7 (d, *J_F_* = 4.7 Hz), 124.7 (d, *J_F_* = 18.3 Hz), 116.3 (d, *J_F_* = 22.3 Hz), 103.6 (d, *J_F_* = 4.1 Hz), 95.7, 18.6, 11.2. **^19^F NMR (470 MHz, CDCl_3_)** δ ‐112.5(m). **HRMS (HESI‐II)**: m/z [M+Na]^+^ calcd for C_26_H_31_FNaOSi: 429.2020; found 429.2015.

#### 4.5.8 (*E*)‐3‐Phenyl‐2‐(3‐((triisopropylsilyl)ethynyl)‐[1,1'‐biphenyl]‐2‐yl)acrylaldehyde (Scheme 2, Product 3ha)

This compound was prepared according to General Procedures using stilbene **1** **h** (0.25 mmol, 1.0 equiv.) and alkynyl bromide **2a** (0.5 mmol, 2.0 equiv.) as substrates, and the reaction was performed under constant current 0.8 mA (1/80 Hz. D = 50%). The crude product was purified by column chromatography (EtOAc/Hexane = 1:10, R*_f_* = 0.62) to afford a sticky colorless oil in 60% yield (69.7 mg). **^1^H NMR (500 MHz, CDCl_3_)** δ 9.53 (s, 1H), 7.63 (dd, *J* = 7.8, 1.3 Hz, 1H), 7.42 (t, *J* = 7.8 Hz, 1H), 7.32‐7.28 (m, 2H), 7.27 (s, 1H), 7.23‐7.20 (m, 2H), 7.19‐7.17 (m, 1H), 7.16‐7.12 (m, 2H), 7.07 (d, *J* = 7.4 Hz, 2H), 6.97 (d, *J* = 6.9 Hz, 2H), 0.97 (s, 21H). **^13^C NMR (125 MHz, CDCl_3_)** δ 193.4, 151.0, 142.4, 140.8, 140.7, 135.6, 134.5, 132.4, 130.5, 130.4, 130.3, 128.6, 128.5, 128.4, 127.8, 127.2, 124.1, 105.3, 94.4, 18.7, 11.3. **HRMS (HESI‐II)**: m/z [M+Na]^+^ calcd for C_32_H_36_NaOSi: 487.2428; found 487.2425.

#### 4.5.9 (*E*)‐3‐Phenyl‐2‐(3‐((triisopropylsilyl)ethynyl)dibenzo[*b,d*]furan‐4‐yl)acrylaldehyde (Scheme 2, Product 3ia)

This compound was prepared according to General Procedures using stilbene **1i** (0.2 mmol, 1.0 equiv.) and alkynyl bromide **2a** (0.4 mmol, 2.0 equiv.) as substrates, and the reaction was performed under constant current 0.6 mA (1/80 Hz. D = 50%). The crude product was purified by column chromatography (EtOAc/Hexane = 1:10, R*_f_* = 0.38) to afford a sticky yellow oil in 70% yield (67.0 mg). **^1^H NMR (500 MHz, CDCl_3_)** δ 9.88 (s, 1H), 7.94 (t, *J* = 8.0 Hz, 2H), 7.74 (s, 1H), 7.60 (d, *J* = 8.0 Hz, 1H), 7.45‐7.41 (m, 2H), 7.36‐7.32 (m, 1H), 7.26‐7.24 (m, 1H), 7.21‐7.19 (m, 2H), 7.18‐7.15 (m, 2H), 0.98 (s, 21H). **^13^C NMR (125 MHz, CDCl_3_)** δ 192.6, 156.9, 153.6, 152.0, 135.6, 134.1, 130.8, 130.6, 128.8, 128.0, 127.8, 124.9, 124.0, 123.2, 122.2, 121.5, 121.0, 120.6, 112.1, 104.9, 95.2, 18.7, 11.3. **HRMS (HESI‐II)**: m/z [M+Na]^+^ calcd for C_32_H_34_NaO_2_Si: 501.2220; found 501.2216.

#### 4.5.10 (*E*)‐3‐Phenyl‐2‐(3‐((triisopropylsilyl)ethynyl)dibenzo[*b*,*d*]thiophen‐4‐yl)acrylaldehyde (Scheme 2, Product 3ja)

This compound was prepared according to General Procedures using stilbene **1i** (0.2 mmol, 1.0 equiv.) and alkynyl bromide **2a** (0.4 mmol, 2.0 equiv.) as substrates, and the reaction was performed under constant current 0.6 mA (1/80 Hz. D = 50%). The crude product was purified by column chromatography (EtOAc/Hexane = 1:10, R*_f_* = 0.40) to afford a yellow solid in 78% yield (77.1 mg). **^1^H NMR (500 MHz, CDCl_3_)** δ 9.86 (s, 1H), 8.15 (t, *J* = 7.5 Hz, 2H), 7.74‐7.70 (m, 3H), 7.46 (t, *J* = 7.5 Hz, 1H), 7.42 (t, *J* = 7.4 Hz, 1H), 7.27‐7.24 (m, 1H), 7.21‐7.15 (m, 4H), 1.00 (s, 21H). **^13^C NMR (125 MHz, CDCl_3_)** δ 192.2, 151.2, 140.3, 139.7, 139.0, 135.8, 135.7, 133.8, 132.2, 130.9, 130.5, 129.4, 128.8, 127.2, 124.7, 123.0, 122.1, 121.9, 121.3, 105.0, 95.5, 18.7, 11.3. **HRMS (HESI‐II)**: m/z [M+Na]^+^ calcd for C_32_H_34_NaOSSi: 517.1992; found 517.1986. **Melting point** 114.8‐116.2°C

#### 4.5.11 Methyl (*E*)‐3‐(3‐oxo‐1‐phenylprop‐1‐en‐2‐yl)‐4‐((triisopropylsilyl)ethynyl)benzoate (Scheme 2, Product 3ka)

This compound was prepared according to General Procedures using stibene **1k** (0.20 mmol, 1.0 equiv.) and alkynyl bromide **2a** (0.4 mmol, 2.0 equiv.) as substrates, and the reaction was performed under constant current 0.6 mA (1/80 Hz. D = 50%). The crude product was purified by column chromatography (EtOAc/Hexane = 1:10, R*_f_* = 0.18) to afford a sticky colorless oil in 30% yield (33.5 mg). **^1^H NMR (500 MHz, CDCl_3_)** δ 9.76 (s, 1H), 8.04 (dd, *J* = 8.5 Hz, 2H), 7.81(d, *J* = 8.5 Hz, 1H), 7.67(d, *J* = 8.5 Hz, 1H), 7.51 (s, 1H), 7.29 (t, *J* = 8.5 Hz, 1H), 7.22 (t, *J* = 8.3 Hz, 2H), 7.14 (d, *J* = 8.1 Hz, 2H), 3.88 (s, 3H), 0.96 (s, 21H). **^13^C NMR (100 MHz, CDCl_3_)** δ 192.9, 166.3, 150.9, 140.3, 137.4, 133.8, 133.3, 130.7, 130.6, 130.2, 129.3, 128.8, 128.2, 104.1, 98.2, 52.4, 18.6, 11.2. **HRMS (HESI‐II)**: m/z [M+H]^+^ calcd for C_28_H_35_O_3_Si: 447.2350; found 447.2352.

#### 4.5.12 (*E*)‐3‐Phenyl‐2‐(3‐((triisopropylsilyl)ethynyl)naphthalen‐2‐yl)acrylaldehyde (Scheme 2, Product 3la)

This compound was prepared according to General Procedures using stilbene **1l** (0.2 mmol, 1.0 equiv.) and alkynyl bromide **2a** (0.4 mmol, 2.0 equiv.) as substrates, and the reaction was performed under constant current 0.6 mA (1/80 Hz. D = 50%). The crude product was purified by column chromatography (EtOAc/Hexane = 1:10, R*_f_* = 0.44) to afford a sticky colorless oil in 52% yield (45.6 mg). **^1^H NMR (500 MHz, CDCl_3_)** δ 9.86 (s, 1H), 8.19 (s, 1H), 7.82 (d, *J* = 8.0 Hz, 1H), 7.82 (d, *J* = 7.9 Hz, 1H),7.64 (s, 1H), 7.58 (s, 1H), 7.57‐7.50 (m, 2H), 7.30‐7.27 (m, 1H), 7.23‐7.19 (m, 4H), 1.03 (s, 21H). **^13^C NMR (125 MHz, CDCl_3_)** δ 193.6, 150.6, 141.0, 134.2, 133.6, 133.5, 133.1, 132.8, 130.9, 130.5, 128.7, 128.1, 127.7, 127.1, 127.0, 121.1, 105.2, 94.2, 18.7, 11.3. **HRMS (HESI‐II)**: m/z [M+Na]^+^ calcd for C_30_H_34_NaOSi: 461.2271; found 461.2265.

#### 4.5.13 (*E*)‐3‐Phenyl‐2‐(2‐((triisopropylsilyl)ethynyl)pyren‐1‐yl)acrylaldehyde (Scheme 2, Product 3ma)

This compound was prepared according to General Procedures using stilbene **1m** (0.2 mmol, 1.0 equiv.) and alkynyl bromide **2a** (0.4 mmol, 2.0 equiv.) as substrates, and the reaction was performed under constant current 0.6 mA (1/80 Hz. D = 50%). The crude product was purified by column chromatography (EtOAc/Hexane = 1:10, R*_f_* = 0.45) to afford a sticky colorless oil in 50% yield (51.3 mg). **^1^H NMR (500 MHz, CDCl_3_)** δ 10.01 (s, 1H), 8.42 (s, 1H), 8.20 (d, *J* = 7.6 Hz, 1H), 8.15‐8.11 (m, 2H), 8.06 (d, *J* = 9.0 Hz, 1H), 8.02‐7.97 (m, 2H), 7.88 (s, 1H), 7.81 (d, *J* = 9.0 Hz, 1H), 7.19‐7.16 (m, 1H), 7.06‐7.04 (m, 4H), 1.03 (s, 21H). **^13^C NMR (125 MHz, CDCl_3_)** δ 193.7, 151.9, 139.7, 134.2, 131.8, 131.5, 131.3, 131.2, 130.7, 130.7, 129.2, 128.9, 128.8, 128.5, 127.1, 126.7, 125.8, 125.7, 124.7, 124.7, 124.5, 121.1, 105.8, 94.9, 18.8, 11.4. **HRMS (HESI‐II)**: m/z [M+Na]^+^ calcd for C_36_H_36_NaOSi: 535.2428; found 535.2422.

#### 4.5.14 (*E*)‐3‐Phenyl‐2‐(2‐((triisopropylsilyl)ethynyl)benzo[*b*]thiophen‐3‐yl)acrylaldehyde (Scheme 2, Product 3na)

This compound was prepared according to General Procedures using stilbene **1n** (0.2 mmol, 1.0 equiv.) and alkynyl bromide **2a** (0.4 mmol, 2.0 equiv.) as substrates. The crude product was purified by column chromatography (EtOAc/Hexane = 1:10, R*_f_* = 0.42) to afford a sticky yellow oil in 57% yield (63.3 mg). **^1^H NMR (500 MHz, CDCl_3_)** δ 9.85 (s, 1H), 7.80 (d, *J* = 8.1 Hz, 1H), 7.74 (s, 1H), 7.38 (t, *J* = 7.5 Hz, 1H), 7.32 (d, *J* = 7.6 Hz, 1H), 7.28‐7.25 (m, 2H), 7.22‐7.19 (m, 4H), 0.98 (s, 21H). **^13^C NMR (125 MHz, CDCl_3_)** δ 192.6, 152.2, 139.7, 137.3, 134.7, 134.7, 133.9, 130.9, 130.7, 128.8, 126.1, 125.0, 123.3, 122.4, 122.3, 101.6, 98.5, 18.6, 11.2. **HRMS (HESI‐II)**: m/z [M+H]^+^ calcd for C_28_H_33_OSSi: 444.2016; found 444.2009. m/z [M+Na]^+^ calcd for C_28_H_32_NaOSSi: 467.1835; found 467.1829.

#### 4.5.15 (*E*)‐3‐Phenyl‐2‐(2‐((triisopropylsilyl)ethynyl)benzo[*b*]furan‐3‐yl)acrylaldehyde (Scheme 2, Product 3oa)

This compound was prepared according to General Procedures using stilbene **1o** (0.2 mmol, 1.0 equiv.) and alkynyl bromide **2a** (0.4 mmol, 2.0 equiv.) as substrates, and the reaction was performed under constant current 0.6 mA (1/80 Hz. D = 50%). The crude product was purified by column chromatography (EtOAc/Hexane = 1:10, R*_f_* = 0.48) to afford a sticky colorless oil in 42% yield (45.0 mg). **^1^H NMR (500 MHz, CDCl_3_)** δ 9.83 (s, 1H), 7.69 (s, 1H), 7.49 (d, *J* = 8.4 Hz, 1H), 7.37‐7.33 (m, 3H), 7.30 (t, *J* = 7.4 Hz, 1H), 7.23 (t, *J* = 7.5 Hz, 2H), 7.18‐7.13 (m, 2H), 1.00 (s, 21H). **^13^C NMR (125 MHz, CDCl_3_)** δ 192.4, 154.7, 151.7, 137.1, 134.0, 131.2, 130.9, 130.8, 128.8, 126.8, 126.3, 123.6, 121.1, 119.0, 111.7, 102.7, 95.3, 18.6, 11.2. **HRMS (HESI‐II)**: m/z [M+H]^+^ calcd for C_28_H_33_O_2_Si: 429.2244; found 429.2237. [M+Na]^+^ calcd for C_28_H_32_NaO_2_Si: 451.2064; found 451.2055.

#### 4.5.16 (*E*)‐2‐(2,6‐Bis((triisopropylsilyl)ethynyl)phenyl)‐3‐phenylacrylaldehyde (Scheme 2, Product 3ra)

This compound was prepared according to General Procedures using stilbene **1r** (0.2 mmol, 1.0 equiv.) and alkynyl bromide **2a** (0.8 mmol, 4.0 equiv.) as substrates, and the reaction was performed under constant current 0.6 mA (1/80 Hz. D = 50%). The crude product was purified by column chromatography (EtOAc/Hexane = 1:10, R*_f_* = 0.42) to afford a white solid in 52% yield (59.1 mg). **^1^H NMR (500 MHz, CDCl_3_)** δ 9.72 (s, 1H), 7.54 (d, *J* = 8.0 Hz, 2H), 7.51 (s, 1H), 7.31 (t, *J* = 7.5 Hz, 1H), 7.27 (d, *J* = 7.5 Hz, 1H), 7.21 (t, *J* = 7.5 Hz, 2H), 7.16 (t, *J* = 8.0 Hz, 2H), 0.95 (s, 42H). **^13^C NMR (125 MHz, CDCl_3_)** δ 192.6, 150.6, 140.2, 134.3, 132.9, 130.5, 130.5, 128.7, 128.2, 124.1, 104.4, 94.9, 18.6, 11.3. **HRMS (HESI‐II)**: m/z [M+Na]^+^ calcd for C_37_H_52_NaOSi_2_: 591.3449; found 591.3441. **Melting point** 76.5‐77.9°C

#### 4.5.17 (*E*)‐2‐(4‐Methoxy‐2,6‐bis((triisopropylsilyl)ethynyl)phenyl)‐3‐phenylacrylaldehyde (Scheme 2, Product 3sa)

This compound was prepared according to General Procedures using stilbene **1s** (0.2 mmol, 1.0 equiv.) and alkynyl bromide **2a** (0.8 mmol, 4.0 equiv.) as substrates, and the reaction was performed under constant current 0.6 mA (1/80 Hz. D = 50%). The crude product was purified by column chromatography (EtOAc/Hexane = 1:10, R*_f_* = 0.32) to afford a sticky colorless oil in 56% yield (67.0 mg). **^1^H NMR (500 MHz, CDCl_3_)** δ 9.71 (s, 1H), 7.49 (s, 1H), 7.30‐7.27 (m, 1H), 7.23 (d, *J* = 7.5 Hz, 2H), 7.18 (d, *J* = 7.0 Hz, 2H), 7.08 (s, 2H), 3.85 (s, 3H), 0.95 (s, 42H). **^13^C NMR (125 MHz, CDCl_3_)** δ 193.00, 158.9, 150.9, 140.0, 134.4, 132.6, 130.5, 130.4, 128.6, 124.9, 118.7, 104.4, 94.6, 55.7, 18.6, 11.3. **HRMS (HESI‐II)**: m/z [M+Na]^+^ calcd for C_27_H_34_NaO_2_Si: 621.3555; found 621.3547.

#### 4.5.18 (*E*)‐2‐(3‐Methoxy‐2,6‐bis((triisopropylsilyl)ethynyl)phenyl)‐3‐phenylacrylaldehyde (Scheme 2, Product 3ta)

This compound was prepared according to General Procedures using stilbene **1t** (0.2 mmol, 1.0 equiv.) and alkynyl bromide **2a** (0.8 mmol, 4.0 equiv.) as substrates, and the reaction was performed under constant current 0.6 mA (1/80 Hz. D = 50%). The crude product was purified by column chromatography (EtOAc/Hexane = 1:10, R*_f_* = 0.30) to afford a white solid in 46% yield (55.1 mg). **^1^H NMR (500 MHz, CDCl_3_)** δ 9.70 (s, 1H), 7.51‐7.49 (m, 2H), 7.28‐7.26 (m, 1H), 7.23‐7.18 (m, 4H), 3.90 (s, 3H), 0.97 (s, 21H), 0.94 (s, 21H). **^13^C NMR (125 MHz, CDCl_3_)** δ 192.5, 161.1, 150.4, 142.2, 140.1, 134.3, 134.0, 130.6, 130.4, 128.6, 116.0, 113.5, 110.6, 104.5, 100.3, 99.6, 92.5, 56.2, 18.7, 11.4, 11.3. **HRMS (HESI‐II)**: m/z [M+Na]^+^ calcd for C_27_H_34_NaO_2_Si: 621.3555; found 621.3547. **Melting point** 79.1‐80.5°C

#### 4.5.19 (*E*)‐3‐(*p*‐Tolyl)‐2‐(2‐((triisopropylsilyl)ethynyl)naphthalen‐1‐yl)acrylaldehyde (Scheme 2, Product 3ua)

This compound was prepared according to General Procedures using stilbene **1u** (0.2 mmol, 1.0 equiv.) and alkynyl bromide **2a** (0.4 mmol, 2.0 equiv.) as substrates, and the reaction was performed under constant current 0.6 mA (1/80 Hz. D = 50%). The crude product was purified by column chromatography (EtOAc/Hexane = 1:10, R*_f_* = 0.44) to afford a sticky yellow oil in 58% yield (52.5 mg). **^1^H NMR (500 MHz, CDCl_3_)** δ 9.88 (s, 1H), 7.86 (d, *J* = 8.5 Hz, 1H), 7.86 (d, *J* = 8.5 Hz, 1H), 7.73 (s, 1H), 7.64 (d, *J* = 8.5 Hz, 1H), 7.60 (d, *J* = 8.5 Hz, 1H), 7.48‐7.45 (m,1H), 7.40‐7.38 (m, 1H), 6.96‐6.91(m, 4H), 2.23 (s, 3H), 0.99 (s, 21H). **^13^C NMR (125 MHz, CDCl_3_)** δ 193.4, 151.8, 141.3, 138.4, 136.0, 133.2, 131.4, 131.1, 130.8, 129.5, 129.3, 128.5, 128.5, 127.3, 126.9, 125.4, 121.1, 105.8, 95.3, 21.6, 18.7, 11.3. **HRMS (HESI‐II)**: m/z [M+Na]^+^ calcd for C_31_H_36_NaOSi: 475.2428; found 475.2423.

#### 4.5.20 (*E*)‐3‐(4‐Methoxyphenyl)‐2‐(2‐((triisopropylsilyl)ethynyl)naphthalen‐1‐yl)acrylaldehyde (Scheme 2, Product 3va)

This compound was prepared according to General Procedures using stilbene **1v** (0.2 mmol, 1.0 equiv.) and alkynyl bromide **2a** (0.4 mmol, 2.0 equiv.) as substrates, and the reaction was performed under constant current 0.6 mA (1/80 Hz. D = 50%). The crude product was purified by column chromatography (EtOAc/Hexane = 1:10, R*_f_* = 0.40) to afford a sticky yellow oil in 48% yield (45.0 mg). **^1^H NMR (500 MHz, CDCl_3_)** δ 9.85 (s, 1H), 7.86 (t, *J* = 8.1 Hz, 1H), 7.85 (d, *J* = 8.1 Hz, 1H), 7.70 (s, 1H), 7.64 (d, *J* = 8.5 Hz, 1H), 7.61 (d, *J* = 8.4 Hz, 1H), 7.49‐7.45 (m, 1H), 7.40‐7.37 (m, 1H), 7.02‐7.00 (m, 2H), 6.65‐6.52 (m, 2H), 3.71 (s, 1H), 7.02 (d, J = 7.8 Hz, 2H), 6.46 (d, J = 15.9 Hz, 1H), 3.73 (s, 3H), 0.99 (s, 21H). **^13^C NMR (125 MHz, CDCl_3_)** δ 193.3, 161.6, 151.5, 137.0, 136.1, 133.3, 132.7, 131.2, 129.3, 128.5, 128.5, 127.3, 127.0, 126.9, 125.4, 121.1, 114.3, 105.8, 95.1, 55.4, 18.7, 11.3. **HRMS (HESI‐II)**: m/z [M+Na]^+^ calcd for C_31_H_36_NaO_2_Si: 491.2377; found 491.2372.

#### 4.5.21 (*E*)‐3‐(4‐Fluorophenyl)‐2‐(2‐((triisopropylsilyl)ethynyl)naphthalen‐1‐yl)acrylaldehyde (Scheme 2, Product 3wa)

This compound was prepared according to General Procedures using stilbene **1w** (0.2 mmol, 1.0 equiv.) and alkynyl bromide **2a** (0.4 mmol, 2.0 equiv.) as substrates, and the reaction was performed under constant current 0.6 mA (1/80 Hz. D = 50%). The crude product was purified by column chromatography (EtOAc/Hexane = 1:10, R*_f_* = 0.33) to afford a sticky colorless oil in 55% yield (50.2 mg). **^1^H NMR (500 MHz, CDCl_3_)** δ 9.87 (s, 1H), 7.87 (d, *J* = 8.1 Hz, 1H), 7.86 (d, *J* = 8.5 Hz, 1H), 7.72 (s, 1H), 7.64 (d, *J* = 8.5 Hz, 1H), 7.57 (d, *J* = 8.4 Hz, 1H), 7.50‐7.47(m, 1H), 7.41‐7.38 (m, 1H), 7.08‐7.05 (m, 2H), 6.83‐6.80 (m, 2H), 0.99 (s, 21H). **^13^C NMR (125 MHz, CDCl_3_)** δ 193.2, 163.9 (d, *J_F_* = 252 Hz), 149.9, 138.9 (d, *J_F_* = 2.0 Hz), 135.3, 133.3, 132.7 (d, *J_F_* = 8.8 Hz), 131.0, 130.4 (d, *J_F_* = 3.0 Hz), 129.3, 128.7, 128.4, 127.4, 127.1, 125.1, 121.2, 116.0 (d, *J_F_* = 21.7 Hz), 105.6, 95.6, 18.7, 11.3. **^19^F NMR (470 MHz, CDCl_3_)** δ ‐108.4. **HRMS (HESI‐II)**: m/z [M+Na]^+^ calcd for C_30_H_33_FNaOSi: 479.21769; found 479.21755. **Melting point** 136.1‐137.3°C.

#### 4.5.22 (*E*)‐3‐(4‐Chlorophenyl)‐2‐(2‐((triisopropylsilyl)ethynyl)naphthalen‐1‐yl)acrylaldehyde (Scheme 2, Product 3xa)

This compound was prepared according to General Procedures using stilbene **1x** (0.2 mmol, 1.0 equiv.) and alkynyl bromide **2a** (0.4 mmol, 2.0 equiv.) as substrates, and the reaction was performed under constant current 0.6 mA (1/80 Hz. D = 50%). The crude product was purified by column chromatography (EtOAc/Hexane = 1:10, R*_f_* = 0.38) to afford a sticky colorless oil in 80% yield (75.5 mg). **^1^H NMR (500 MHz, CDCl_3_)** δ 9.88 (s, 1H), 7.87 (d, *J* = 8.1 Hz, 1H), 7.86 (d, *J* = 8.5 Hz, 1H), 7.71 (s, 1H), 7.63 (d, *J* = 8.5 Hz, 1H), 7.56 (d, *J* = 8.4 Hz, 1H), 7.50‐7.47(m, 1H), 7.41‐7.38 (m, 1H), 7.10‐7.08 (m, 2H), 7.01‐6.98 (m, 2H), 1.00 (s, 21H). **^13^C NMR (125 MHz, CDCl_3_)** δ 193.0, 149.6, 139.7, 136.7, 135.2, 133.2, 132.6, 131.7, 130.9, 129.3, 129.1, 128.8, 128.7, 127.5, 127.1, 125.1, 121.1, 105.5, 95.8, 18.7, 11.3. **HRMS (HESI‐II)**: m/z [M+Na]^+^ calcd for C_30_H_33_ClNaOSi: 495.1881; found 495.1876. **Melting point** 116.1‐117.5°C

#### 4.5.23 (*E*)‐3‐(3‐(Trifluoromethyl)phenyl‐2‐(2‐((triisopropylsilyl)ethynyl)naphthalen‐1‐yl)acrylaldehyde (Scheme 2, Product 3ya)

This compound was prepared according to General Procedures using stibene **1y** (0.2 mmol, 1.0 equiv.) and alkynyl bromide **2a** (0.5 mmol, 2.0 equiv.) as substrates, and the reaction was performed under constant current 0.6 mA (1/80 Hz. D = 50%). The crude product was purified by column chromatography (EtOAc/Hexane = 1:10, R*_f_* = 0.30) to afford a sticky colorless oil in 42% yield (47.0 mg). **^1^H NMR (500 MHz, CDCl_3_)** δ 9.92 (s, 1H), 7.88 (d, *J* = 8.5 Hz, 2H), 7.78 (s, 1H), 7.63 (d, *J* = 8.5 Hz, 1H), 7.56 (d, *J* = 8.3 Hz, 1H), 7.51‐7.45 (m, 2H), 7.44 (s, 1H), 7.42‐7.39 (m, 1H), 7.18 (t, *J* = 7.8 Hz, 1H), 7.12 (d, *J* = 8.1 Hz, 1H), 0.98 (s, 21H). **^13^C NMR (125 MHz, CDCl_3_)** δ 192.9, 148.9, 140.9, 133.2, 132.7, 131.2 (d, *J_F_* = 32.5 Hz), 131.0, 129.3, 129.2, 129.0, 128.7, 127.7 (d, *J_F_* = 3.8 Hz), 127.5, 127.1, 126.9 (d, *J_F_* = 3.6 Hz), 125.0, 123.4 (d, *J_F_* = 270.8 Hz), 121.1, 105.4, 96.0, 18.6, 11.3. **^19^F NMR (470 MHz, CDCl_3_)** δ ‐63.3. **HRMS (HESI‐II)**: m/z [M+H]^+^ calcd for C_31_H_33_F_3_OSi: 507.2326; found 507.2336.

#### 4.5.24 (*E*)‐4‐Methyl‐2‐(2‐((triisopropylsilyl)ethynyl)naphthalen‐1‐yl)pent‐2‐enal (Scheme 2, Product 3za)

This compound was prepared according to General Procedures using stibene **1z** (0.20 mmol, 1.0 equiv.) and alkynyl bromide **2a** (0.4 mmol, 2.0 equiv.) as substrates, and the reaction was performed under constant current 0.6 mA (1/80 Hz. D = 50%). The crude product was purified by column chromatography (EtOAc/Hexane = 1:10, R*_f_* = 0.62) to afford a light‐yellow oil in 15% yield (11.7 mg). **^1^H NMR (400 MHz, CDCl_3_)** δ 10.32 (s, 1H), 7.82 (dd, *J* = 7.4, 1.6 Hz, 1H), 7.76 (d, *J* = 8.5 Hz, 1H), 7.58(d, *J* = 8.5 Hz, 1H), 7.54 (dd, *J* = 7.5, 1.4 Hz, 1H), 7.44 (quintd, *J* = 7.4, 2.0 Hz, 2H), 2.47 (s, 3H), 1.69 (s, 3H), 1.10 (s, 21H). **^13^C NMR (100 MHz, CDCl_3_)** δ 189.6, 158.7, 138.1, 136.2, 133.2, 131.9, 129.1, 128.5, 127.9, 126.9, 126.6, 125.4, 121.4, 106.4, 94.2, 29.9, 24.7, 18.8, 11.4. **HRMS (HESI‐II)**: m/z [M+H]^+^ calcd for C_26_H_35_OSi: 391.2452; found 391.2452; m/z [M+Na]^+^ calcd for C_26_H_34_NaOSi: 413.2271; found 413.2271.

#### 4.5.25 (*E*)‐2‐(2‐((*tert*‐Butyldimethylsilyl)ethynyl)naphthalen‐1‐yl)‐3‐phenylacrylaldehyde (Scheme 2, Product 3ab)

This compound was prepared according to General Procedures using stilbene **1a** (0.2 mmol, 1.0 equiv.) and TBS‐protected alkynyl bromide **2b** (0.4 mmol, 2.0 equiv.) as substrates, and the reaction was performed under constant current 0.6 mA (1/80 Hz. D = 50%). The crude product was purified by column chromatography (EtOAc/Hexane = 1:10, R*_f_* = 0.56) to afford a sticky colorless oil in 67% yield (53.0 mg). **^1^H NMR (500 MHz, CDCl_3_)** δ 9.89 (s, 1H), 7.86 (d, *J* = 8.0 Hz, 1H), 7.85 (d, *J* = 8.1 Hz, 1H), 7.77 (s, 1H), 7.62 (d, *J* = 8.5 Hz, 1H), 7.59 (d, *J* = 8.5 Hz, 1H), 7.49‐7.46(m, 1H), 7.40‐7.37 (m, 1H), 7.23 (t, *J* = 7.3 Hz, 1H), 7.12 (t, *J* = 7.5 Hz, 2H), 7.07 (d, *J* = 7.9 Hz, 2H), 0.58 (s, 9H), 0.06 (s, 3H), 0.03(s, 3H). **^13^C NMR (125 MHz, CDCl_3_)** δ 193.3, 151.5, 139.2, 135.9, 134.1, 133.3, 131.1, 130.7, 130.6, 129.0, 128.8, 128.6, 128.6, 127.3, 127.0, 125.3, 120.8, 104.6, 97.4, 26.1, 16.8, ‐4.6. **HRMS (HESI‐II)**: m/z [M+Na]^+^ calcd for C_27_H_28_NaOSi: 419.1802; found 419.1795.

#### 4.5.26 (*E*)‐3‐Phenyl‐2‐(2‐((triethylsilyl)ethynyl)naphthalen‐1‐yl)acrylaldehyde (Scheme 2, Product 3ac)

This compound was prepared according to General Procedures using stilbene **1a** (0.2 mmol, 1.0 equiv.) and TES‐procted alkynyl bromide **2c** (0.4 mmol, 2.0 equiv.) as substrates, and the reaction was performed under constant current 0.6 mA (1/80 Hz. D = 50%). The crude product was purified by column chromatography (EtOAc/Hexane = 1:10, R*_f_* = 0.55) to afford a sticky colorless oil in 62% yield (49.1 mg). **^1^H NMR (500 MHz, CDCl_3_)** δ 9.89 (s, 1H), 7.87‐7.84 (m, 2H), 7.77 (s, 1H), 7.62 (d, *J* = 8.5 Hz, 1H), 7.59 (d, *J* = 8.5 Hz, 1H), 7.49‐7.46(m, 1H), 7.40‐7.37 (m, 1H), 7.23 (t, *J* = 7.3 Hz, 1H), 7.12 (t, *J* = 7.5 Hz, 1H), 7.07 (d, *J* = 7.9 Hz, 1H), 0.90 (t, *J* = 8.0 Hz, 9H), 0.53 (q, *J* = 8.0 Hz, 6H). **^13^C NMR (125 MHz, CDCl_3_)** δ 193.3, 151.4, 139.2, 136.0, 134.1, 133.3, 131.1, 130.6, 130.6, 129.0, 128.8, 128.6, 128.6, 127.3, 127.0, 125.4, 120.9, 105.2, 96.6, 7.6, 4.5. **HRMS (HESI‐II)**: m/z [M+Na]^+^ calcd for C_27_H_28_NaOSi: 419.1802; found 419.1797.

#### 4.5.27 (*E*)‐2‐(2‐Methyl‐6‐((triisopropylsilyl)ethynyl)phenyl)‐3‐phenylprop‐2‐en‐1‐ol (Scheme 5, Compound 8)

This compound was prepared according to General Procedures using compound **3ba** (2.0 mmol, 1.0 equiv.) as substrate. The crude product was purified by column chromatography (EtOAc/Hexane = 1:10, R*_f_* = 0.12) to afford a white solid in 96% yield (0.77 g). **^1^H NMR (500 MHz, CDCl_3_)** δ 7.43 (d, *J* = 7.1 Hz, 1H), 7.20‐7.15 (m, 2H), 7.11‐7.10 (m, 3H), 6.95 (d, *J* = 3.3, 2H), 6.94 (s, 1H), 6.79 (s, 1H), 4.47 (s, 2H), 2.11 (s, 3H), 4.47 (s, 2H), 1.04 (s, 21H). **^13^C NMR (125 MHz, CDCl_3_)** δ 140.9, 139.5, 136.7, 136.4, 130.8, 130.7, 128.4, 128.2, 127.8, 127.4, 127.1, 123.4, 106.3, 93.5, 68.2, 19.8, 18.7, 18.7, 11.4. **HRMS (HESI‐II)**: m/z [M+Na]^+^ calcd for C_27_H_36_NaOSi: 427.2428; found 427.2420. **Melting point**: 102.3‐104.5 °C

#### 4.5.28 (*E*)‐2‐(2‐Methyl‐6‐((triisopropylsilyl)ethynyl)phenyl)‐3‐phenylacrylic acid (Scheme 5, Compound 9)

This compound was prepared according to General Procedures using compound **3ba** (2.0 mmol, 1.0 equiv.) as substrate. The crude product was purified by column chromatography (MeOH/EtOAc/Hexane = 1:5:10, R*_f_* = 0.12) to afford a colorless oil in 95% yield (0.79 g). **^1^H NMR (500 MHz, CDCl_3_)** δ 7.43 (d, *J* = 8.0 Hz, 1H), 7.26‐7.16 (m, 5H), 7.05 (d, *J* = 8.0 Hz, 2H), 2.10 (s, 3H), 0.98 (s, 21H). **^13^C NMR (125 MHz, CDCl_3_)** δ 172.3, 143.2, 138.1, 136.9, 134.7, 130.5, 130.4, 130.3, 129.8, 129.7, 128.6, 128.0, 124.0, 105.4, 94.2, 19.9, 18.7, 11.3. **HRMS (HESI‐II)**: m/z [M‐H]^−^ calcd for C_27_H_34_O_2_Si: 417.2255; found 417.2256.

#### 4.5.29 (*E*)‐2‐(3‐Ethynyldibenzo[*b,d*]furan‐4‐yl)‐3‐phenylacrylaldehyde (Scheme 5, Compound 10)

This compound was prepared according to General Procedures using compound **3ia** (0.5 mmol, 1.0 equiv.) as substrate. The crude product was purified by column chromatography (EtOAc/Hexane = 1:10, R*_f_* = 0.55) to afford a white solid in 90% yield (0.145 g). **^1^H NMR (500 MHz, CDCl_3_)** δ 9.91 (s, 1H), 7.96 (d, *J* = 8.0 Hz, 2H), 7.80 (s, 1H), 7.63 (d, *J* = 8.0 Hz, 1H), 7.44‐7.42 (m, 2H), 7.36‐7.33 (m, 1H), 7.27‐7.24 (m, 1H), 7.26‐7.10 (m, 4H), 3.06 (s, 3H). **^13^C NMR (125 MHz, CDCl_3_)** δ 192.5, 156.9, 153.6, 152.2, 135.2, 134.2, 130.8, 130.4, 128.8, 128.1, 128.0, 125.4, 123.9, 123.3, 121.8, 121.1, 120.8, 112.1, 82.0, 81.1. **HRMS (HESI‐II)**: m/z [M+Na]^+^ calcd for C_23_H_14_NaO_2_: 345.0886; found 345.0878. **Melting point** 217.6‐219.2 °C.

[CCDC 2463153, CCDC 2463058, and CCDC 2463060 contain the supplementary crystallographic data for this paper. These data can be obtained free of charge from The Cambridge Crystallographic Data Centre via www.ccdc.cam.ac.uk/data_request/cif.] (Supporting Information).

## Author Contributions

**Junya Wang**: methodology, investigation, writing – original draft, data curation, formal analysis. **Xiaozhou Wang**: methodology, investigation, data curation. **Pui Ying Choy**: writing – original draft, investigation, methodology, writing – review and editing. **Chi‐Wai Cheung**: writing – original draft. **Li‐Wen Xu**: funding acquisition, project administration. **Hung Kay Lee**: data curation, formal analysis. **Gui‐Juan Cheng**: supervision, writing – review and editing, funding acquisition, project administration, conceptualization. **Fuk Yee Kwong**: funding acquisition, writing – review and editing, project administration, supervision, resources, conceptualization.

## Conflicts of Interest

The authors declare no conflicts of interest.

## Supporting information




**Supporting File**: advs76881‐sup‐0001‐SuppMat.pdf.

## Data Availability

The data that support the findings of this study are available in the Supporting Information of this article. Copies of the NMR spectra are also provided. The X‐ray crystallography coordinates for structures of **3ja** (CCDC 2463153), **8** (CCDC 2463058) and **10** (CCDC 2463060) have been deposited at the Cambridge Crystallographic Data Centre (CCDC).
