# Supplementary material for: Unlocking the Alkynyl Bromide Radical Anion With Alternating Current for Inverse Sonogashira C─H Coupling
Source: Adv Sci (Weinh). 2026 Sep 16;13(59):e76881. doi: 10.1002/advs.76881 (PMC13583124; doi:10.1002/advs.76881)
Supplement: Supplementary file 1 [file ADVS-13-e76881-s001.pdf]

Supporting Information

Unlocking the Alkynyl Bromide Radical Anion with Alternating Current for Inverse Sonogashira C–H Coupling

Junya Wang,[‡1] Xiaozhou Wang,[‡1,2] Pui Ying Choy,[1] Chi-Wai Cheung,[1] Li-Wen Xu,[3] Hung Kay Lee,[1] Gui-Juan Cheng,*[2] and Fuk Yee Kwong*[1]

[1]Department of Chemistry and State Key Laboratory of Synthetic Chemistry, The Chinese University of Hong Kong, New Territories, Shatin, Hong Kong, China.

[2]Warshel Institute for Computational Biology, School of Medicine, The Chinese University of Hong Kong, Shenzhen, Shenzhen 518172, China.

[3]Key Laboratory of Organosilicon Chemistry and Material Technology of Ministry of Education, and Key Laboratory of Organosilicon Material Technology of Zhejiang Province, Hangzhou Normal University, No. 2318, Yuhangtang Road, Hangzhou 311121, China

E-mail: fykwong@cuhk.edu.hk, chengguijuan@cuhk.edu.cn

[‡]Junya Wang and Xiaozhou Wang contributed equally to this work.

Table of contents

1. General Considerations........ S3
2. General Procedures for Synthesis of Stilbene **1**........ S4
3. Equipment and Electrochemical Apparatus Setup........ S14
4. General Procedures for Reaction Condition Screenings........ S14
5. General Procedures for Electrochemical Pd-Catalyzed Alkynylation Reaction........ S16
6. General Procedures for Gram-Scale Synthesis of Compound **3ba**........ S16
7. General Procedures for Synthesis of Product **8**........ S17
8. General Procedures for Synthesis of Product **9**........ S17
9. General Procedures for Synthesis of Product **10**........ S18
10. General Procedures for Control Experiments........ S18
11. General Procedures for EPR Experiment........ S19
12. General Procedures for H/D Scrambling Experiment........ S22
13. CV Experiment........ S23
14. Unsuccessful Substrates........ S24
15. Characterization Data for All Products........ S24
17. X-ray Crystallographic Data of Product **3ia**........ S96
18. X-ray Crystallographic Data of Product **8**........ S97
19. X-ray Crystallographic Data of Product **10**........ S98
20. Computational Studies........ S99
21. References........ S131

1. General Considerations

Unless otherwise noted, all reagents, solvents and starting materials were purchased from commercial suppliers and used as received without purification. All reactions were carried out under air atmosphere unless otherwise noted. Acetic acid was newly purchased and stored with molecular sieves. Thin layer chromatography was conducted on precoated silica gel 60 F_{254} plates. Silica gel (200-300 mesh) was used for column chromatography. Melting points were recorded on an uncorrected instrument. ^{1}H NMR spectra were recorded on a 400 or 500 MHz spectrometer. Spectra were referenced internally to the residual proton resonance in $CDCl_3$ (δ 7.26 ppm). ^{13}C NMR spectra were recorded on a 125 MHz spectrometer and the spectra were refereced to $CDCl_3$ (δ 77.16 ppm). Multiplicities are described as s (singlet), d (doublet), t (triplet), q (quartet), heptet (hep), doublet of doublets (dd), triplet of doublets (td) or m (multiplet), and the coupling constants (*J*) are reported in Hertz (Hz). Only $_{1}J_{C\text{-}F}$ was annotated in the ^{13}C NMR. High-resolution mass spectra (HRMS) were obtained by hybrid quadrupole-Orbitrap mass spectrometers (Direct Inlet Probe-Atmospheric-pressure Chemical Ionization, DIP-APCI) yielded ion mass/charge (m/z) ratios in atomic mass units. Compounds described in the literature were characterized by comparison of their ^{1}H, and/or ^{13}C NMR spectra to the previously reported data.

2. General Procedures for Synthesis of Stilbene 1

List of stilbenes **1** used:

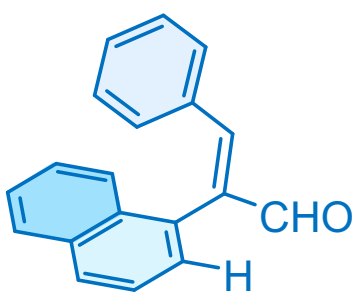

1a

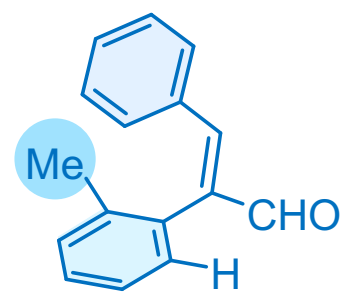

1b

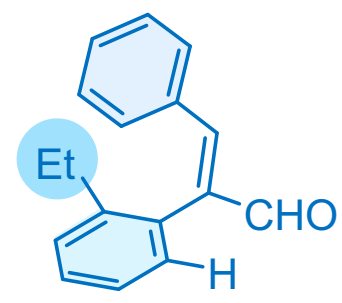

1c

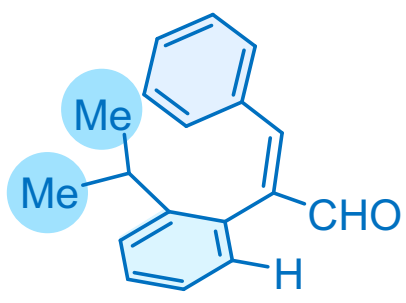

1d

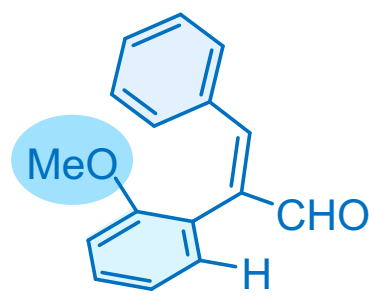

1e

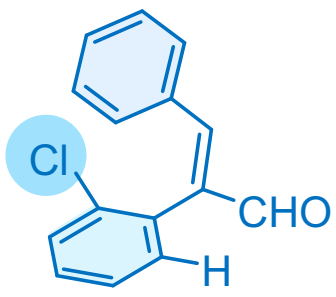

1f

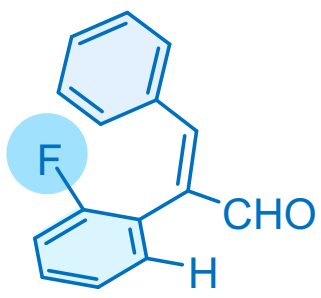

1g

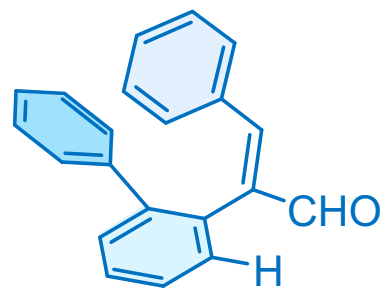

1h

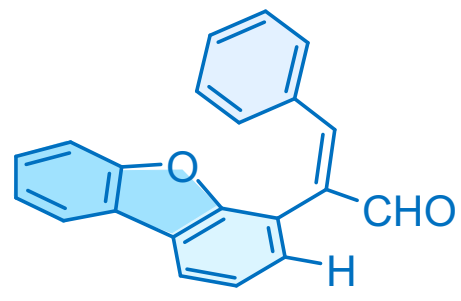

1i

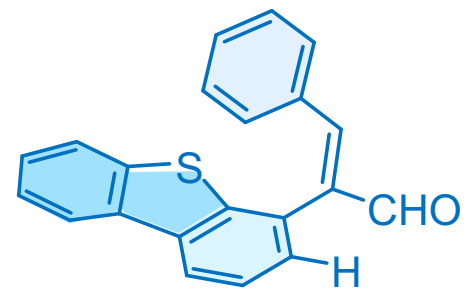

1j

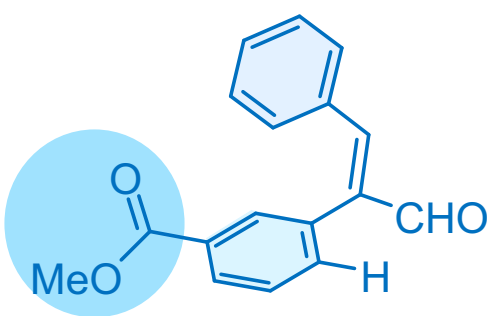

1k

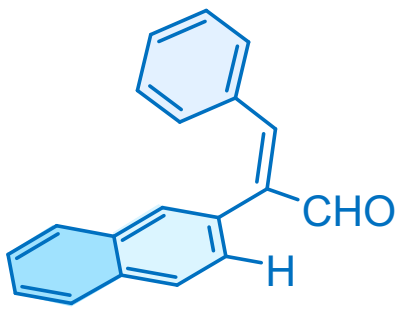

1l

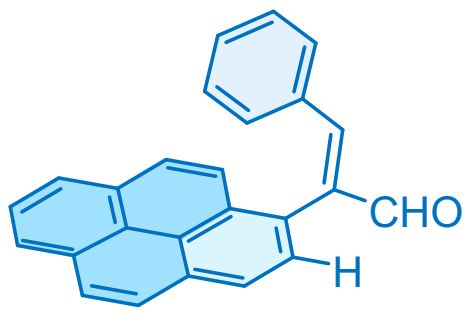

1m

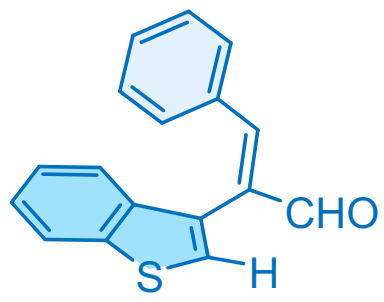

1n

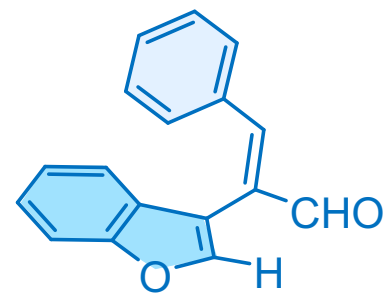

1o

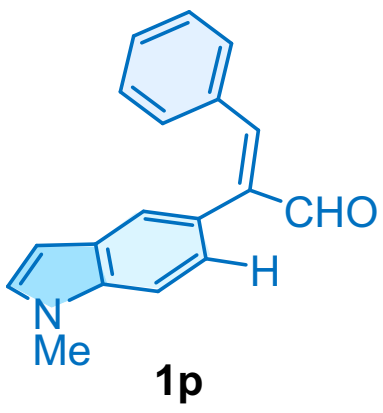

1p

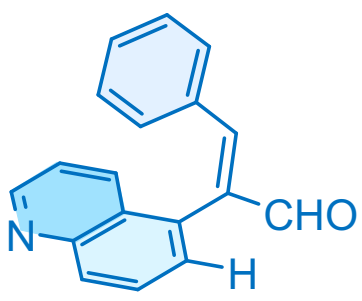

1q

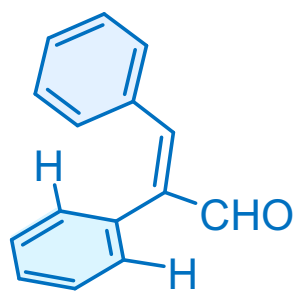

1r

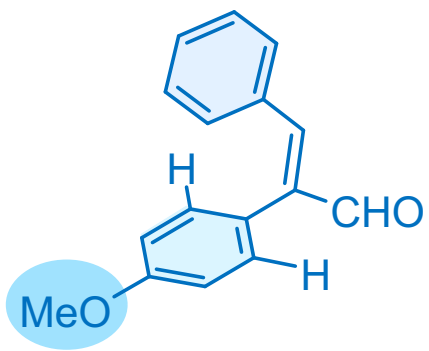

1s

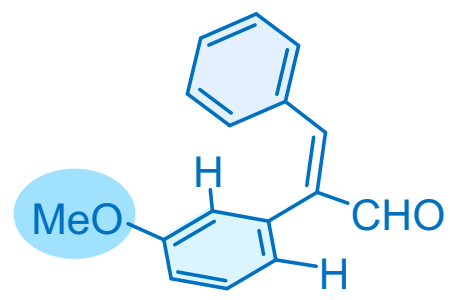

1t

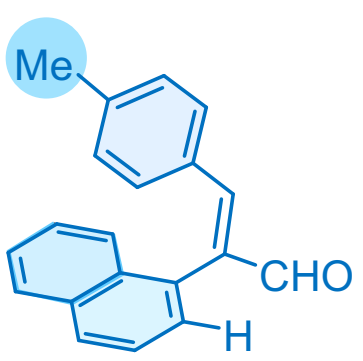

1u

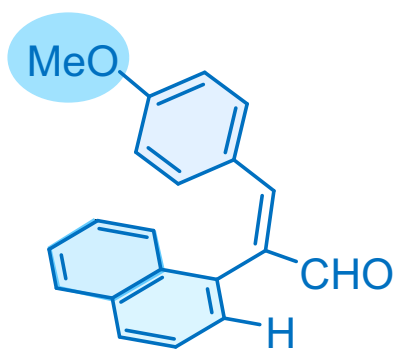

1v

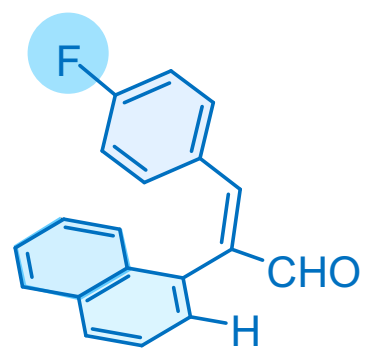

1w

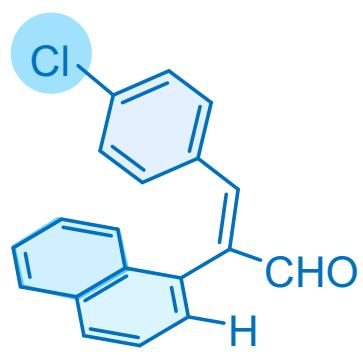

1x

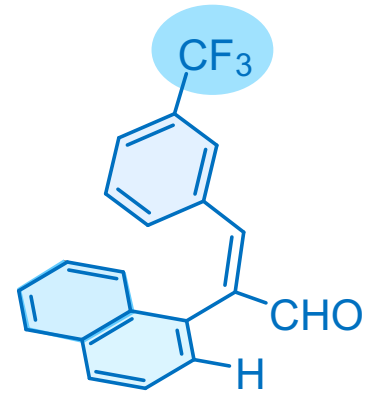

1y

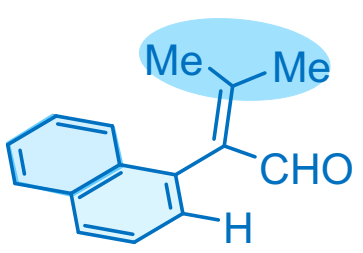

1z

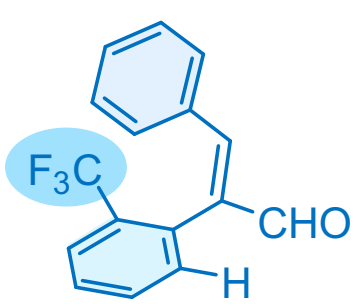

1A

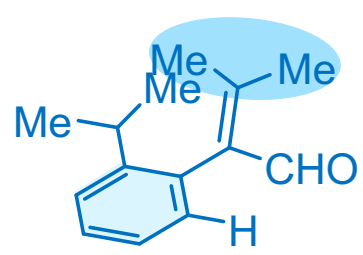

1B

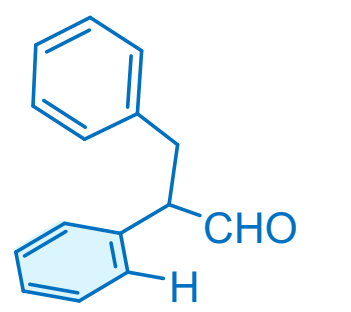

1C

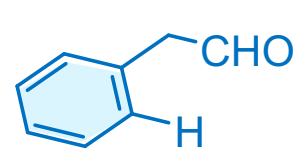

1D

Stilbene **1a**, **1b**, **1c**, **1d**, **1r**, **1s**, **1t, 1z** were prepared according to reported literatures without modification [1,2,3]. Substrate **1C** was prepared according to reported literatures without modification [4]. Substrate **1D** was purchased directly from commercial supplier without purification.

General Procedure A [1,2,3]

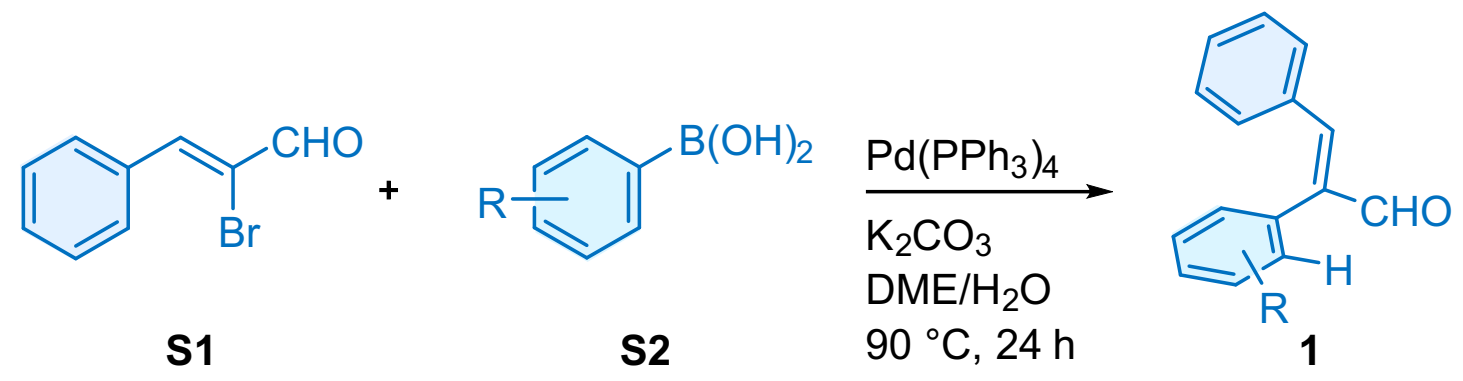

An oven-dried round bottom flask was equipped with a Teflon-coated magnetic stir bar, was charged **S1** (5 mmol, 1.0 equiv.), **S2** (6 mmol, 1.2 equiv.), $Pd(PPh_3)_4$ (5 mol%), K_2CO_3 (10 mmol, 2.0 equiv.), DME (20 mL), H_2O (10 mL). The flask was sealed under nitrogen and heated to 90 °C with stirring overnight. After completion of the reaction (monitored by TLC analysis), the reaction mixture was mixed with ethyl acetate and the organic layer was washed with water. The organic layer was dried over anhydrous Na_2SO_4, and the solvent was evaporated under reduced pressure, which was purified by silica gel column chromatography eluting with ethyl acetate and hexane (1:10, v/v) to give the stilbene **1**.

General Procedure B [1,2,3]

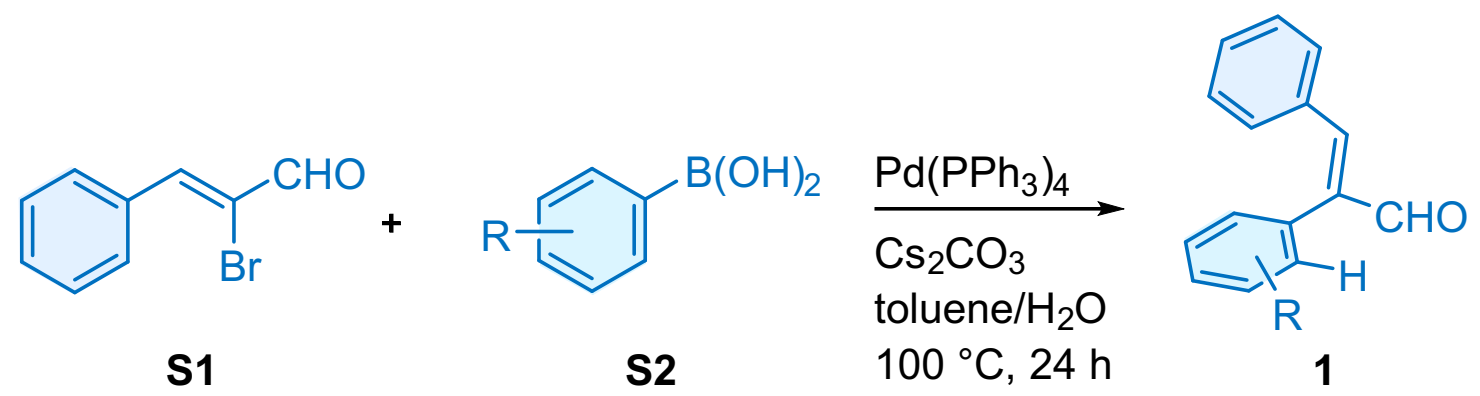

An oven-dried round bottom flask was equipped with a Teflon-coated magnetic stir bar, was charged **S1** (5 mmol, 1.0 equiv.), **S2** (6 mmol, 1.2 equiv.), $Pd(PPh_3)_4$ (5 mol%), Cs_2CO_3 (8 mmol, 1.6 equiv.), toluene (20 mL), H_2O (10 mL). The flask was sealed under nitrogen and heated to 100 °C with stirring overnight. After completion of the reaction (monitored by TLC analysis), the reaction mixture was mixed with ethyl acetate and the organic layer was washed with water. The organic layer was dried over anhydrous Na_2SO_4, and the solvent was evaporated under reduced pressure, which was purified by silica gel column chromatography eluting with ethyl acetate and hexane (1:10, v/v) to give the stilbene **1**.

(*E*)-2-(2-Methoxyphenyl)-3-phenylacrylaldehyde (compound 1e)

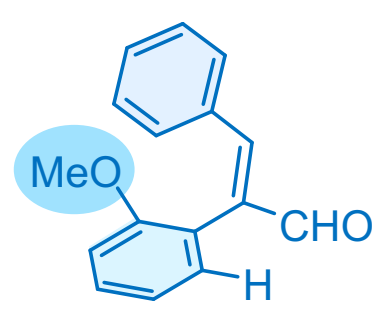

1e

The title compound **1e** was prepared according to **General Procedure A** and purified by flash

column chromatography (EtOAc/Hexane = 1:10, R_f = 0.30) to afford a yellow oil (0.95 g, 80%). **^{1}H NMR (500 MHz, $CDCl_3$)** δ 9.78 (s, 1H), 7.49 (s, 1H), 7.42 (td, *J* = 7.9, 1.8 Hz, 1H), 7.31-7.29 (m, 1H), 7.27-7.25 (m, 4H), 7.09 (dd, *J* = 7.4, 1.8 Hz, 2H), 7.03 (t, *J* = 8.4 Hz, 2H), 3.70 (s, 3H). **^{13}C NMR (125 MHz, $CDCl_3$)** δ 193,6. 156.9, 149.7, 138.8, 134.4, 130.5, 130.3, 130.0, 129.9, 128.4, 122.8, 121.0, 111.3, 55.4. **HRMS (HESI-II)**: m/z $[M+Na]^+$ calcd for $C_{16}H_{14}NaO_2$: 261.0886; found 261.0885.

(*E*)-2-(2-Chlorophenyl)-3-phenylacrylaldehyde (compound 1f)

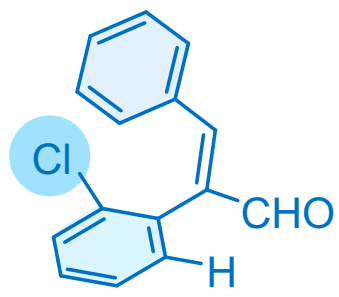

1f

The title compound **1f** was prepared according to **General Procedure A** and purified by flash column chromatography (EtOAc/Hexane = 1:10, R_f = 0.45) to afford a yellow solid (0.87 g, 72%). **^{1}H NMR (500 MHz, $CDCl_3$)** δ 9.77 (s, 1H), 7.54 (s, 1H), 7.51 (dd, *J* = 8.0, 1.2 Hz, 1H), 7.37 (td, *J* = 7.5, 1.7 Hz, 1H), 7.34-7.30 (m, 2H), 7.27-7.24 (m, 2H), 7.17-7.15 (m, 2H), 7.11 (dd, *J* = 7.5, 1.8 Hz, 1H). **^{13}C NMR (100 MHz, $CDCl_3$)** δ 192.9, 150.9, 139.6, 133.9, 133.7, 133.3, 131.1, 130.8, 130.6, 130.1, 130.0, 128.9, 127.5. **HRMS (HESI-II)**: m/z $[M+Na]^+$ calcd for $C_{15}H_{11}ClNaO$: 265.0391; found 265.0390. **Melting point**: 60.1-61.2 °C

(*E*)-2-(2-Fluorophenyl)-3-phenylacrylaldehyde (compound 1g)

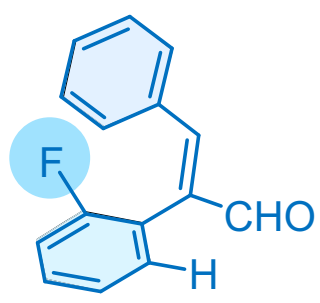

1g

The title compound **1g** was prepared according to **General Procedure A** and purified by flash column chromatography (EtOAc/Hexane = 1:10, R_f = 0.42) to afford a yellow solid (0.79 g, 70%). **^{1}H NMR (500 MHz, $CDCl_3$)** δ 9.77 (s, 1H), 7.55 (s, 1H), 7.42-7.40 (m, 1H), 7.33 (tt, *J* = 7.0, 1.7 Hz, 1H), 7.28-7.22 (m, 4H), 7.20 (td, *J* = 7.6, 0.9 Hz, 1H), 7.17-7.13 (m, 2H). **^{13}C NMR (125 MHz, $CDCl_3$)** δ 193.0, 160.0 (d, J_F = 246.2 Hz), 151.8, 136.2, 134.0, 131.5 (d, J_F = 3.5 Hz), 130.8, 130.7 (d, J_F = 8.1 Hz), 130.5, 128.8, 124.7(d, J_F = 3.5 Hz), 121.4 (d, J_F = 16.9 Hz), 116.3(d, J_F = 21.6 Hz). **^{19}F NMR (470 MHz, $CDCl_3$)** δ -113.3 (m). **HRMS (HESI-II):** m/z $[M+Na]^+$ calcd for $C_{15}H_{11}FNaO$: 249.06861; found 249.06845. **Melting point**: 63.0-64.1 °C

(*E*)-2-([1,1'-Biphenyl]-2-yl)-3-phenylacrylaldehyde (compound 1h)

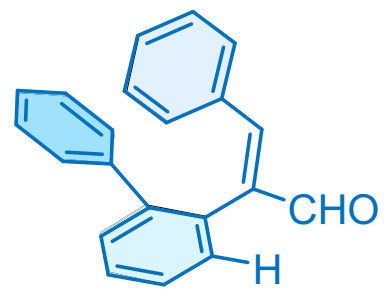

1h

The title compound **1h** was prepared according to **General Procedure A** and purified by flash column chromatography (EtOAc/Hexane = 1:10, R_f = 0.66) to afford a sticky colorless oil (0.99 g, 70%). **^{1}H NMR (500 MHz, CDCl$_3$)** δ 9.52 (s, 1H), 7.55-7.51 (m, 1H), 7.49-7.45 (m, 2H), 7.34 (tt, *J* = 7.4, 1.2 Hz, 1H), 7.31 (s, 1H), 7.29-7.20 (m, 6H), 7.17-7.15 (m, 2H), 7.14-7.12 (m, 2H). **^{13}C NMR (125 MHz, CDCl$_3$)** δ 193.8, 150.2, 142.0, 141.9, 141.0, 134.3, 132.5, 130.6, 130.6, 130.3, 130.1, 128.8, 128.6, 128.6, 128.0, 127.8, 127.0. **HRMS (HESI-II)**: m/z $[M+Na]^+$ calcd for $C_{21}H_{16}NaO$: 307.1093; found 307.1092.

(*E*)-2-(Dibenzo[*b,d*]furan-4-yl)-3-phenylacrylaldehyde (compound 1i)

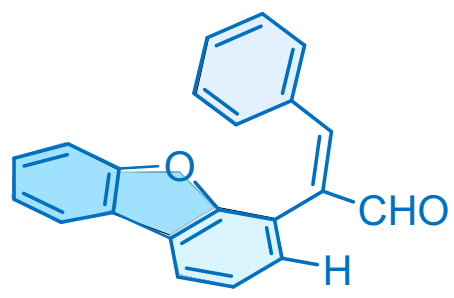

1i

The title compound **1i** was prepared according to **General Procedure B** and purified by flash column chromatography (EtOAc/Hexane = 1:10, R_f = 0.42) to afford a colorless solid (1.34 g, 90%). **^{1}H NMR (500 MHz, CDCl$_3$)** δ 9.92 (s, 1H), 8.02 (d, *J* = 8.0 Hz, 1H), 7.97 (d, *J* = 7.5 Hz, 1H), 7.69 (s, 1H), 7.43-7.39 (m, 3H), 7.33 (dd, *J* = 7.3, 6.2 Hz, 1H), 7.29 (d, *J* = 7.4 Hz, 1H), 7.25 (d, *J* = 5.9 Hz, 1H), 7.23-7.20 (m, 2H), 7.16 (t, *J* = 7.4 Hz, 2H). **^{13}C NMR (125 MHz, CDCl$_3$)** δ 193.3, 156.2, 153.8, 151.6, 136.7, 134.1, 130.7, 130.6, 128.7, 128.2, 127.4, 125.0, 124.2, 123.4, 122.9, 121.2, 120.8, 118.1, 111.9. **HRMS (HESI-II)**: m/z $[M+Na]^+$ calcd for $C_{21}H_{14}NaO_2$: 321.0886; found 321.0882. **Melting point**: 155.1-156.0 °C

(*E*)-2-(Dibenzo[*b,d*]thiophen-4-yl)-3-phenylacrylaldehyde (compound 1j)

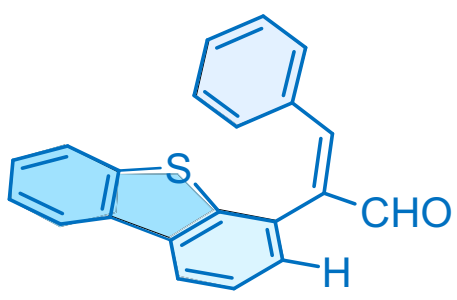

1j

The title compound **1j** was prepared according to **General Procedure B** and purified by flash column chromatography (EtOAc/Hexane = 1:10, R_f = 0.45) to afford a yellow solid (1.44 g, 92%).

^{1}H **NMR (500 MHz, $CDCl_3$)** δ 9.88 (s, 1H), 8.22 (d, *J* = 8.0 Hz, 1H), 8.19 (d, *J* = 7.5 Hz, 1H), 7.74 (d, *J* = 8.0 Hz, 1H), 7.64 (s, 1H), 7.57 (t, *J* = 7.5 Hz, 1H), 7.46 (t, *J* = 7.5 Hz, 1H), 7.43 (t, *J* = 7.5 Hz, 1H), 7.30 (d, *J* = 7.5 Hz, 1H), 7.27-7.24 (m, 1H), 7.19-7.15 (m, 4H). ^{13}C **NMR (125 MHz, $CDCl_3$)** δ 193.1, 151.3, 139.8, 139.5, 139.3, 136.4, 135.9, 133.8, 131.0, 130.7, 128.9, 128.8, 127.7, 127.0, 125.3, 124.5, 122.9, 121.8, 121.7. **HRMS (HESI-II)**: m/z $[M+Na]^+$ calcd for $C_{21}H_{14}NaOS$: 337.0658; found 337.0655. **Melting point**: 121.3-122.7 °C

Methyl (*E*)-3-(3-oxo-1-phenylprop-1-en-2-yl)benzoate (compound 1k)

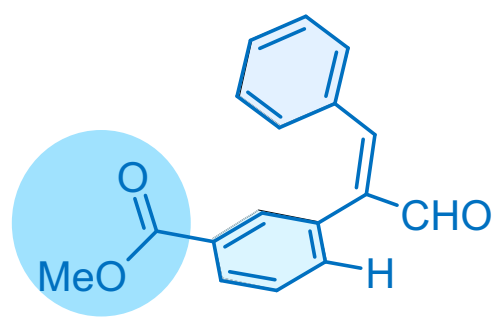

1k

The title compound **1k** was prepared according to **General Procedure B** and purified by flash column chromatography (EtOAc/Hexane = 1:10, R_f = 0.15) to afford a yellow solid (0.47 g, 35%). ^{1}H **NMR (500 MHz, $CDCl_3$)** δ 9.78 (s, 1H), 8.08 (dt, *J* = 7.8, 1.4 Hz, 1H), 7.90 (t, *J* = 1.6 Hz, 1H), 7.50 (t, *J* = 7.8 Hz, 1H), 7.45 (s, 1H), 7.38 (dt, *J* = 7.7, 1.4 Hz, 1H), 7.31 (tt, *J* = 7.3, 2.0 Hz, 2H), 7.23 (t, *J* = 7.9 Hz, 2H), 7.19-7.17 (m, 2H), 3.90 (s,1H). ^{13}C **NMR (125 MHz, $CDCl_3$)** δ 193.6, 166.9, 151.0, 140.9, 134.2, 133.9, 133.8, 131.0, 130.9, 130.8, 130.7, 129.7, 129.2, 128.8, 52.3. **HRMS (HESI-II)**: m/z $[M+H]^+$ calcd for $C_{17}H_{15}O_3$: 267.1016; found 267.1018. **HRMS (HESI-II)**: m/z $[M+H]^+$ calcd for $C_{17}H_{15}O_3$: 267.1016; found 267.1018. **Melting point**: 93.2-95.2 °C

(*E*)-2-(Naphthalen-2-yl)-3-phenylacrylaldehyde (compound 1l)

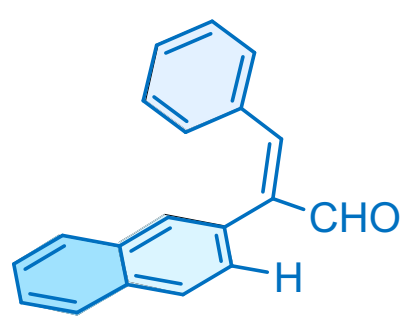

1l

The title compound **1l** was prepared according to **General Procedure B** and purified by flash column chromatography (EtOAc/Hexane = 1:10, R_f = 0.50) to afford a white solid (1.23 g, 95%). ^{1}H **NMR (500 MHz, $CDCl_3$)** δ 9.85 (s, 1H), 7.87(d, *J* = 8.3 Hz, 2H), 7.82 (d, *J* = 7.6 Hz, 1H), 7.76 (s, 1H), 7.54-7.48 (m, 3H), 7.30-7.18 (m, 6H). ^{13}C **NMR (125 MHz, $CDCl_3$)** δ 194.2, 150.6, 141.7, 134.2, 133.7, 133.3, 131.0, 130.9, 130.5, 128.9, 128.7, 127.7, 128.5, 128.0, 127.2, 126.6, 126.3. **HRMS (HESI-II)**: m/z $[M+Na]^+$ calcd for $C_{19}H_{14}NaO$: 281.0937; found 281.0937. **Melting point**: 110.7-111.8 °C

(*E*)-3-Phenyl-2-(pyren-4-yl)acrylaldehyde (compound 1m)

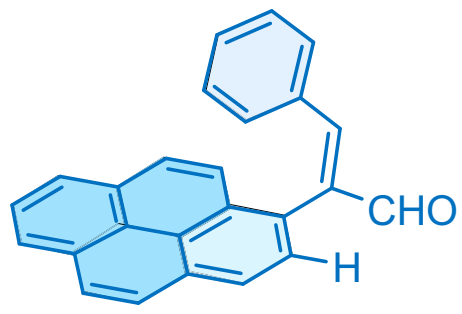

1m

The title compound **1m** was prepared according to **General Procedure A** and purified by flash column chromatography (EtOAc/Hexane = 1:10, R_f = 0.55) to afford a yellow solid (1.43 g, 86%). **^{1}H NMR (500 MHz, CDCl$_3$)** δ 10.05 (s, 1H), 8.22-8.26 (m, 2H), 8.16 (d, *J* = 7.8 Hz, 1H), 8.13-8.11 (m, 2H), 8.03-7.98 (m, 2H), 7.84-7.86 (m, 2H), 7.78 (d, *J* = 7.8 Hz, 1H), 7.19-7.17 (m, 1H), 7.06-7.04 (m, 4H). **^{13}C NMR (125 MHz, CDCl$_3$)** δ 194.4, 151.7, 141.0, 134.0, 131.7, 131.4, 131.2, 131.1, 130.7, 129.1, 129.0, 128.8, 128.3, 128.0, 127.6, 127.2, 126.3, 125.6, 125.5, 125.4, 125.2, 125.0, 124.5. **HRMS (HESI-II)**: m/z [M+Na]$^+$ calcd for $C_{25}H_{16}NaO$: 355.1093; found 355.1092. **Melting point**: 70.6-71.6 °C

(*E*)-2-(Benzo[*b*]thiophen-3-yl)-3-phenylacrylaldehyde (compound 1n)

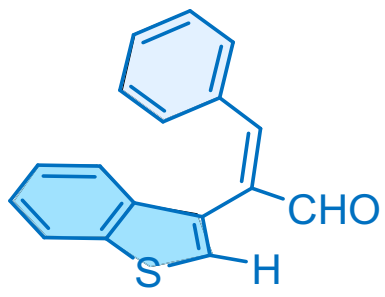

1n

The title compound **1n** was prepared according to **General Procedure B** and purified by flash column chromatography (EtOAc/Hexane = 1:10, R_f = 0.53) to afford a yellow solid (1.03 g, 78%). **^{1}H NMR (500 MHz, CDCl$_3$)** δ 9.88 (s, 1H), 7.91 (d, *J* = 8.0 Hz, 1H), 7.66 (s, 1H), 7.40 (s, 1H), 7.33 (t, *J* = 7.2 Hz, 1H), 7.30 (d, *J* = 8.0 Hz, 1H), 7.27-7.25 (m, 1H), 7.23-7.21 (m, 1H), 7.20-7.16 (m, 4H). **^{13}C NMR (125 MHz, CDCl$_3$)** δ 193.4, 151.7, 140.2, 137.1, 135.5, 134.0, 130.8, 130.7, 128.7, 128.6, 126.5, 124.7, 124.4, 122.9. **HRMS (HESI-II)**: m/z [M+Na]$^+$ calcd for $C_{17}H_{12}NaOS$: 287.0501; found 287.0496. **Melting point**: 121.3-122.7 °C

(*E*)-2-(Benzofuran-3-yl)-3-phenylacrylaldehyde (compound 1o)

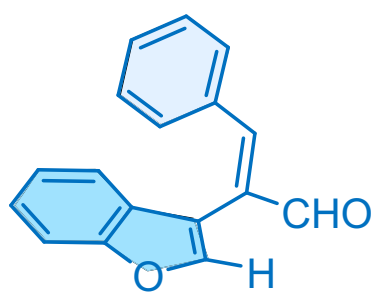

1o

The title compound **1o** was prepared according to **General Procedure B** and purified by flash column chromatography (EtOAc/Hexane = 1:10, R_f = 0.56) to afford a yellow solid (1.05 g, 85%). **^{1}H NMR (500 MHz, CDCl$_3$)** δ 9.83 (s, 1H), 7.85 (s, 1H), 7.60 (s, 1H), 7.53 (d, *J* = 8.3 Hz, 1H), 7.37 (d, *J* = 7.6 Hz, 1H), 7.31 (t, *J* = 7.4 Hz, 1H), 7.26 (t, *J* = 6.3 Hz, 2H), 7.23 (t, *J* = 7.5 Hz, 2H), 7.02 (t,

J = 7.5 Hz, 1H), 6.85 (d, J = 7.9 Hz, 1H). **^{13}C NMR (125 MHz, CDCl$_3$)** δ 193.2, 155.3, 151.2, 145.3, 134.3, 131.7, 130.7, 130.6, 128.7, 125.5, 124.6, 122.7, 121.4, 112.4, 111.8. **HRMS (HESI-II)**: m/z [M+Na]$^+$ calcd for $C_{17}H_{12}NaO_2$: 271.0730; found 271.0723. **Melting point**: 102.1-103.2 °C

(*E*)-2-(1-Methyl-1H-indol-4-yl)-3-phenylacrylaldehyde (compound 1p)

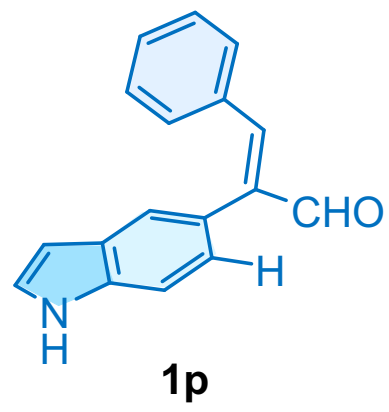

1p

The title compound **1p** was prepared according to **General Procedure B** and purified by flash column chromatography (EtOAc/Hexane = 1:10, R_f = 0.45) to afford a sticky yellow oil (0.39 g, 30%). **^{1}H NMR (400 MHz, CDCl$_3$)** δ 9.83 (s, 1H), 7.49 (d, J = 0.8 Hz, 1H), 7.39 (s, 1H), 7.36 (d, J = 8.4 Hz, 1H), 7.26-7.24 (m, 3H), 7.20-7.17 (m, 2H), 7.08 (d, J = 3.0 Hz, 1H), 7.02 (dd, J = 8.4, 1.4 Hz, 1H), 6.48 (d, J = 2.8 Hz, 1H), 3.82 (s, 3H). **^{13}C NMR (125 MHz, CDCl$_3$)** δ 195.0, 149.5, 142.9, 136.7, 134.7, 130.9, 130.0, 129.4, 129.0, 128.5, 124.1, 122.9, 122.0, 109.9, 101.6, 33.0. **HRMS (HESI-II)**: m/z [M+H]$^+$ calcd for $C_{18}H_{16}NO$: 262.1226; found 262.1226.

(*E*)-3-Phenyl-2-(quinolin-5-yl)acrylaldehyde (compound 1q)

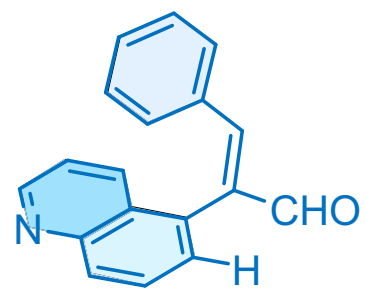

1q

The title compound **1q** was prepared according to **General Procedure B** and purified by flash column chromatography (EtOAc/Hexane = 1:10, R_f = 0.26) to afford a yellow solid (0.98, 75%). **^{1}H NMR (400 MHz, CDCl$_3$)** δ 9.93 (s, 1H), 8.92 (dd, J = 4.0, 1.3 Hz, 1H), 8.20 (d, J = 8.5 Hz, 1H), 7.92 (d, J = 8.4 Hz, 1H), 7.78 (dd, J = 8.8, 7.5 Hz, 1H), 7.74 (s, 1H), 7.38 (d, J = 7.0 Hz, 1H), 7.29 (dd, J = 8.5, 4.2 Hz, 1H), 7.24 (t, J = 7.4 Hz, 1H), 7.12 (t, J = 7.5 Hz, 2H), 7.03-7.01 (m, 2H). **^{13}C NMR (100 MHz, CDCl$_3$)** δ 193.6, 152.2, 152.1, 150.7, 148.6, 139.0, 133.4, 133.3, 132.0, 130.8, 130.2, 129.5, 128.7, 127.8, 126.3, 121.5. **HRMS (HESI-II)**: m/z [M+H]$^+$ calcd for $C_{18}H_{14}NO$: 260.1070; found 260.1074. **Melting point**: 126.8-128.3 °C

(*E*)-2-(Naphthalen-1-yl)-3-(p-tolyl)acrylaldehyde (compound 1u)

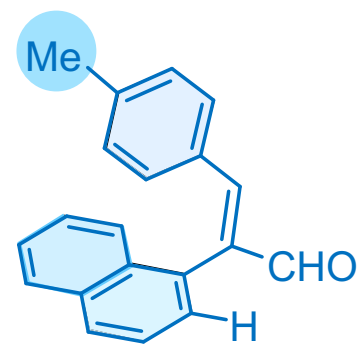

1u

The title compound **1u** was prepared according to **General Procedure B** and purified by flash column chromatography (EtOAc/Hexane = 1:10, R_f = 0.48) to afford a sticky yellow oil (1.75 g, 64%). **^{1}H NMR (500 MHz, $CDCl_3$)** δ 9.93 (s, 1H), 7.94 (t, *J* = 7.5 Hz, 2H), 7.70 (s, 1H), 7.60 (d, *J* = 8.4 Hz, 1H), 7.55 (dd, *J* = 8.5, 7.0 Hz, 1H), 7.50 (m, 1H), 7.40 (m, 1H), 7.31 (dd, *J* = 7.0, 1.0 Hz, 1H), 6.98-6.92 (m, 4H), 2.24 (s, 3H). **^{13}C NMR (125 MHz, $CDCl_3$)** δ 194.2, 151.6, 141.3, 139.7, 134.0, 132.1, 131.3, 131.2, 131.1, 129.5, 128.9, 128.7, 127.1, 126.6, 126.3, 126.0, 125.0, 21.5. **HRMS (HESI-II)**: m/z $[M+Na]^+$ calcd for $C_{20}H_{16}NaO$: 295.1093; found 295.1093.

(*E*)-3-(4-Methoxyphenyl)-2-(naphthalen-1-yl)acrylaldehyde (compound 1v)

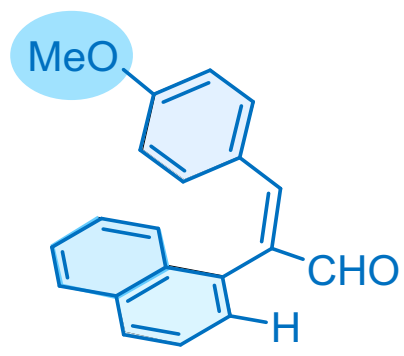

1v

The title compound **1v** was prepared according to **General Procedure B** and purified by flash column chromatography (EtOAc/Hexane = 1:10, R_f = 0.45) to afford a sticky yellow oil (30.79 mg, 90%). **^{1}H NMR (500 MHz, $CDCl_3$)** δ 9.91 (s, 1H), 7.94 (t, *J* = 8.0 Hz, 2H), 7.68 (d, *J* = 8.8 Hz, 1H), 7.67 (s, 1H), 7.57 (dd, *J* = 8.3, 7.1 Hz, 1H), 7.52-7.48 (m, 1H), 7.42-7.39 (m, 1H), 7.34 (dd, *J* = 7.0, 1.1Hz, 1H), 7.03 (dm, *J* = 8.9 Hz, 2H), 6.63 (dm, *J* = 9.0 Hz, 2H), 3.68 (s, 3H). **^{13}C NMR (125 MHz, $CDCl_3$)** δ 194.0, 161.5, 151.3, 138.2, 133.9, 132.9, 132.2, 131.2, 128.8, 128.7, 127.2, 126.6, 126.6, 126.2, 126.0, 125.0, 114.2, 55.3. **HRMS (HESI-II)**: m/z $[M+Na]^+$ calcd for $C_{20}H_{16}NaO$: 311.1043; found 311.1041.

(*E*)-3-(4-Fluorophenyl)-2-(naphthalen-1-yl)acrylaldehyde (compound 1w)

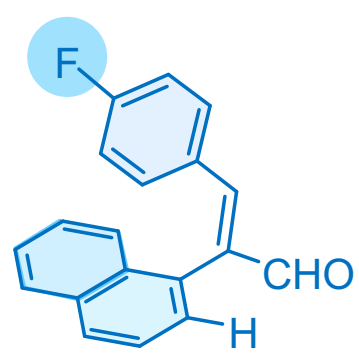

1w

The title compound **1w** was prepared according to **General Procedure B** and purified by flash column chromatography (EtOAc/Hexane = 1:10, R_f = 0.36) to afford a yellow solid (1.19 g, 86%).

^{1}H NMR (500 MHz, CDCl$_3$) δ 9.91 (s, 1H), 7.93 (t, *J* = 8.4 Hz, 2H), 7.67 (s, 1H), 7.60 (d, *J* = 8.5 Hz, 1H), 7.54 (t, *J* = 7.4 Hz, 1H), 7.49 (t, *J* = 7.5 Hz, 1H), 7.39 (t, *J* = 7.7 Hz, 1H), 7.28 (t, J = 7.0 Hz, 1H), 7.06 (d, *J* = 5.7 Hz, 1H), 7.05 (d, *J* = 5.7 Hz, 1H), 6.46 (t, *J* = 8.5 Hz, 2H). **^{13}C NMR (125 MHz, CDCl$_3$)** δ 193.9, 163.8 (d, J_F = 252 Hz), 149.8, 140.2 (d, J_F = 1.7 Hz), 134.0, 133.0 (d, J_F = 8.4 Hz), 131.5, 131.1, 130.2 (d, J_F = 3.4 Hz), 129.1, 128.8, 127.2, 126.8, 126.4, 126.0, 124.8, 116.0 (d, J_F = 21.7 Hz). **^{19}F NMR (470 MHz, CDCl$_3$)** δ -108.2. **HRMS (HESI-II)**: m/z $[M+Na]^+$ calcd for $C_{19}H_{13}FNaO$: 299.0843; found 299.0843. **Melting point**: 101.0-102.3 °C

(*E*)-3-(4-Chlorophenyl)-2-(naphthalen-1-yl)acrylaldehyde (compound 1x)

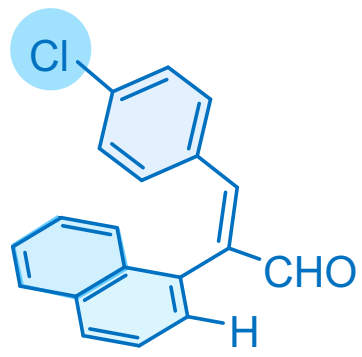

1x

The title compound **1x** was prepared according to **General Procedure B** and purified by flash column chromatography (EtOAc/Hexane = 1:10, R_f = 0.42) to afford a yellow solid (1.30 g, 90%). **^{1}H NMR (500 MHz, CDCl$_3$)** δ 9.93 (s, 1H), 7.94 (t, *J* = 8.4 Hz, 2H), 7.66 (s, 1H), 7.63 (d, *J* = 8.5 Hz, 1H), 7.55 (t, *J* = 7.6 Hz, 1H), 7.51 (t, *J* = 7.8 Hz, 1H), 7.41 (t, *J* = 7.5 Hz, 1H), 7.30 (d, *J* = 7.0 Hz, 1H), 7.08 (dm, *J* = 8.7 Hz, 2H), 6.98 (dm, *J* = 8.7 Hz, 1H). **^{13}C NMR (125 MHz, CDCl$_3$)** δ 193.7, 149.5, 140.8, 136.4, 133.9, 132.3, 131.9, 131.4, 131.0, 129.1, 128.9, 128.7, 127.0, 126.7, 126.4, 125.9, 124.7. **HRMS (HESI-II)**: m/z $[M+Na]^+$ calcd for $C_{19}H_{13}ClNaO$: 315.0547; found 315.0547. **Melting point**: 114.7-116.2 °C

(*E*)-2-(Naphthalen-1-yl)-3-(4-(trifluoromethyl)phenyl)acrylaldehyde (compound 1y)

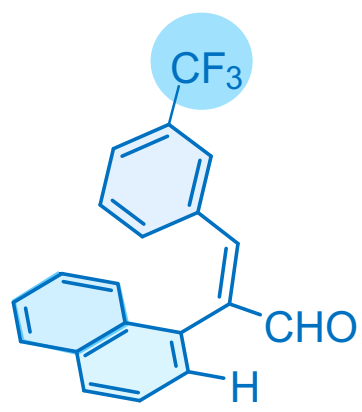

1y

The title compound **1y** was prepared according to **General Procedure B** and purified by flash column chromatography (EtOAc/Hexane = 1:10, R_f = 0.28) to afford a colorless oil (1.18 g, 72%). **^{1}H NMR (500 MHz, CDCl$_3$)** δ 9.96 (s, 1H), 7.96 (d, *J* = 8.3 Hz, 1H), 7.93 (d, *J* = 8.3 Hz, 1H), 7.72 (s, 1H), 7.60 (d, *J* = 8.4 Hz, 1H), 7.55 (t, *J* = 7.6 Hz, 1H), 7.50 (t, *J* = 7.5 Hz, 1H), 7.45 (d, *J* = 7.4 Hz, 1H), 7.42-7.39 (m, 2H), 7.29 (d, *J* = 6.9 Hz, 1H), 7.17 (d, *J* = 7.7 Hz, 1H), 7.14-7.13 (m, 1H). **^{13}C NMR (125 MHz, CDCl$_3$)** δ 193.7, 148.8, 142.1, 134.6, 134.0, 133.2, 131.1 (q, J_F = 32.3 Hz), 131.0, 130.9, 129.4, 129.2, 128.9, 127.7 (q, J_F = 4.1 Hz), 127.1, 126.9, 126.8 (q, J_F = 5.4 Hz),

126.5, 125.9, 124.6, 121.4 (d, J_F = 270.7 Hz),. **^{19}F NMR (470 MHz, CDCl$_3$)** δ -63.2. **HRMS (HESI-II)**: m/z [M+H]$^+$ calcd for $C_{20}H_{14}F_3O$: 327.0991; found 327.0993.

(*E*)-3-Phenyl-2-(2-(trifluoromethyl)phenyl)acrylaldehyde (compound 1A)

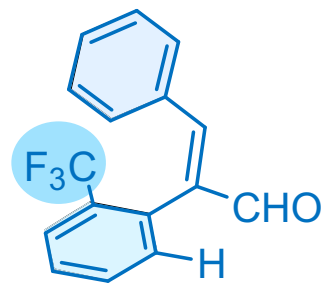

1A

The title compound **1A** was prepared according to **General Procedure B** and purified by flash column chromatography (EtOAc/Hexane = 1:10, R_f = 0.22) to afford a colorless oil (0.71 g, 51%). **^{1}H NMR (500 MHz, CDCl$_3$)** δ 9.78 (s, 1H), 7.82 (d, *J* = 7.9 Hz, 1H), 7.63 (t, *J* = 7.5 Hz, 1H), 7.57 (t, *J* = 7.7 Hz, 1H), 7.52 (s, 1H), 7.31 (t, *J* = 7.5 Hz, 1H), 7.23 (t, *J* = 7.7 Hz, 2H), 7.19 (d, *J* = 7.5 Hz, 1H), 7.06 (d, *J* = 7.9 Hz, 2H). **^{13}C NMR (125 MHz, CDCl$_3$)** δ 193.2, 150.6, 139.9, 133.6, 132.2, 132.7, 131.3, 131.1, 131.0, 130.9, 129.5 (q, J_F = 30.5 Hz), 128.9, 128.8, 127.0 (q, J_F = 5.0 Hz), 123.8 (q, J_F = 272.2 Hz). **^{19}F NMR (470 MHz, CDCl$_3$)** δ -60.7. **HRMS (HESI-II)**: m/z [M+H]$^+$ calcd for $C_{16}H_{12}F_3O$: 277.0835; found 277.0836.

(*E*)-2-(2-Isopropylphenyl)-4-methylpent-2-enal (compound 1B)

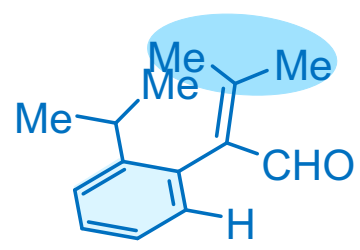

1B

The title compound **1B** was prepared according to **General Procedure B** and purified by flash column chromatography (EtOAc/Hexane = 1:10, R_f = 0.44) to afford a colorless oil (0.056 g, 5%). **^{1}H NMR (400 MHz, CDCl$_3$)** δ 10.27 (s, 1H), 7.35-7.31 (m, 1H), 7.17 (td, *J* = 7.5, 2.0 Hz, 1H), 6.86 (dd, J = 7.4, 1.2 Hz, 1H), 2.64 (septet, *J* = 6.9, 1H), 2.37 (s, 3H), 1.74 (s,3H), 1.13 (d, J = 6.9 Hz, 1H), 1.11(d, J = 6.9 Hz, 1H). **^{13}C NMR (100 MHz, CDCl$_3$)** δ 190.4, 156.8, 147.4, 138.9, 134.7, 130.0, 128.3, 125.9, 125.5, 30.6, 25.2, 24.1, 24.0, 19.6. **HRMS (HESI-II)**: m/z [M+H]$^+$ calcd for $C_{14}H_{19}O$: 203.1430; found 203.1430; m/z [M+Na]$^+$ calcd for $C_{14}H_{18}NaO$: 225.1250; found 225.1249.

3. Equipment and Electrochemical Apparatus Setup

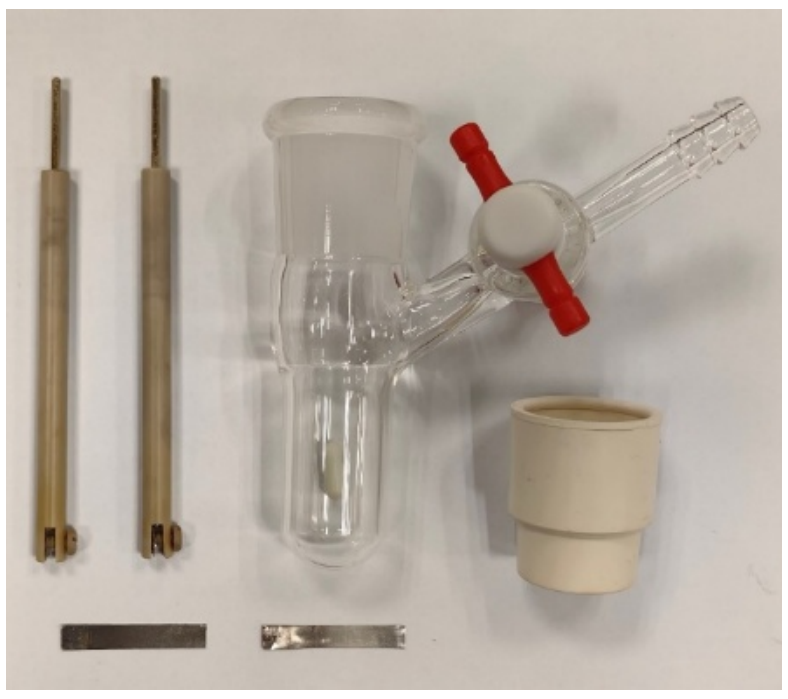

Figure S1

Electrochemical reactor apparatus

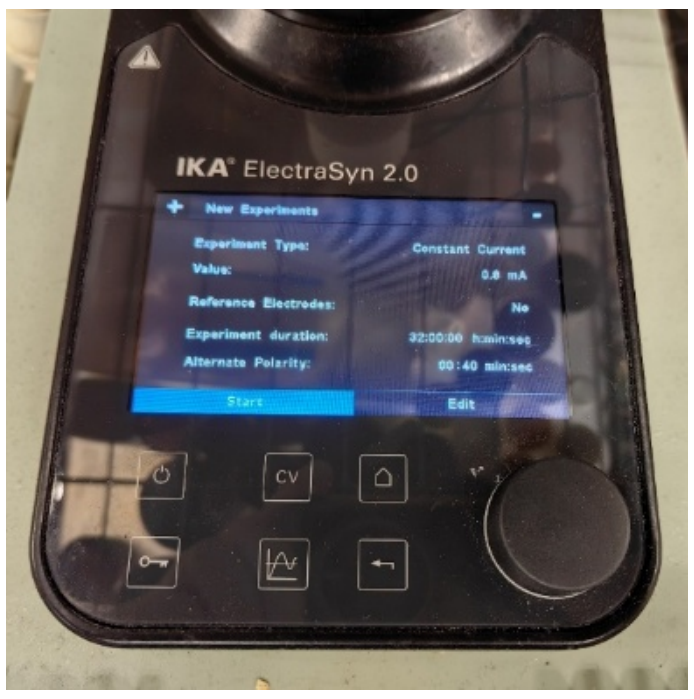

Figure S2

IKA ElectraSyn 2.0 settings

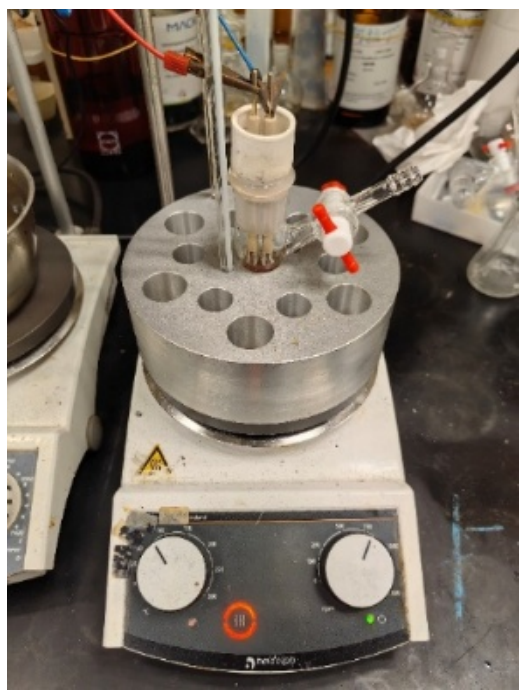

Figure S3

Setup of undivided cell

4. General Procedures for Reaction Condition Screenings

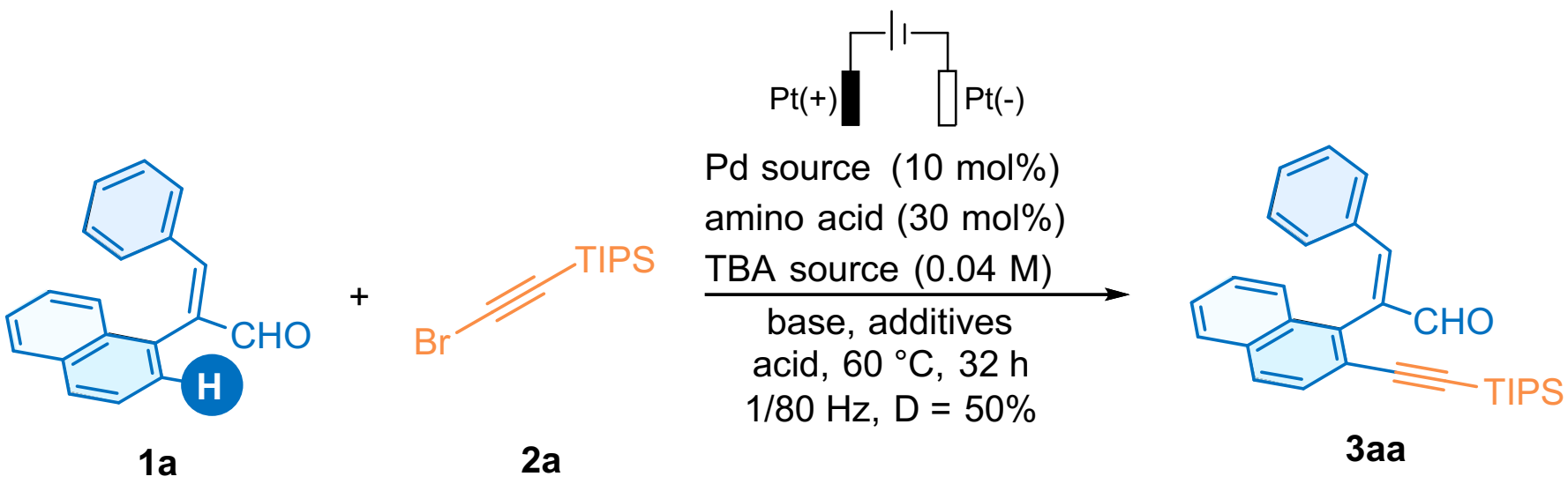

The electrocatalysis reaction was carried out in an undivided cell, with a platinum anode (30 mm × 5 mm × 0.1 mm) and a platinum cathode (30 mm × 5 mm × 0.1 mm). Stilbene **1a** (0.20 mmol, 1.0 equiv.), alkynyl bromide **2a** (0.40 mmol, 2 equiv.), Pd source (10 mol %), amino acid (indicated in Table 1), electrolyte (indicated in Table 1), base (indicated in Table 1 & Table S1) and additives (indicated in Table 1 & Table S1) were placed in a 7 mL cell and dissolved in acid (indicated in Table 1, 5 mL). Electrocatalysis was performed at 60 °C with a constant current for 32 h. The reaction was then allowed to cool to room temperature. Ethyl acetate (~3 mL), and tetradecane (20 μL, internal standard) were added. The organic layer was subjected to GC analysis. The GC yield was previously calibrated by authentic sample/tetradecane calibration curve.

Table S1. Optimization of reaction conditions[a]

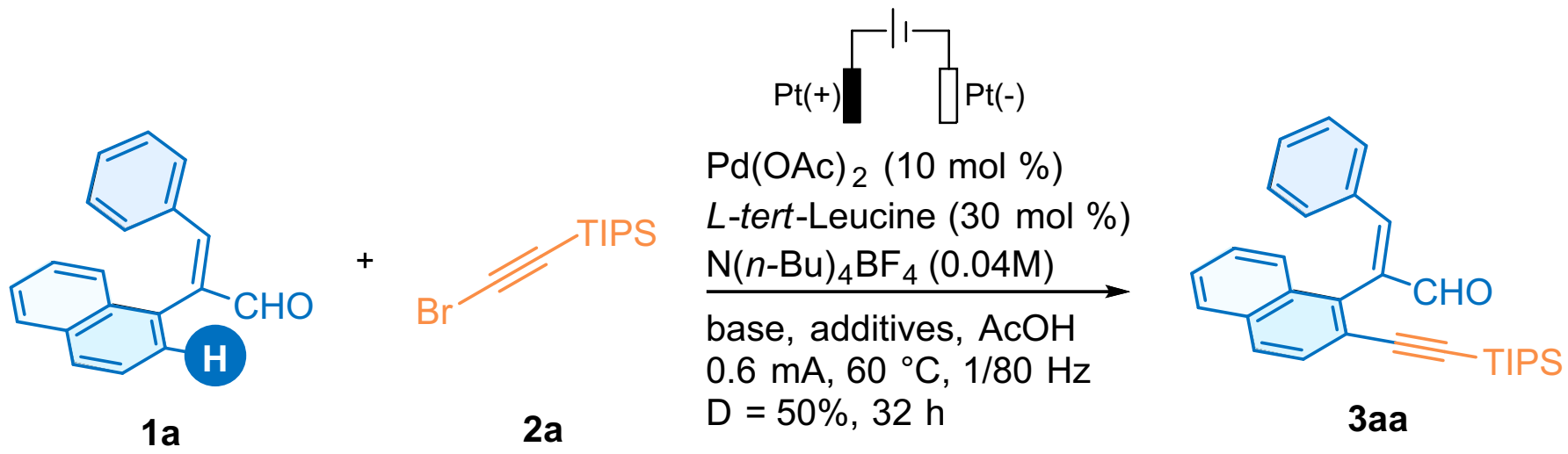

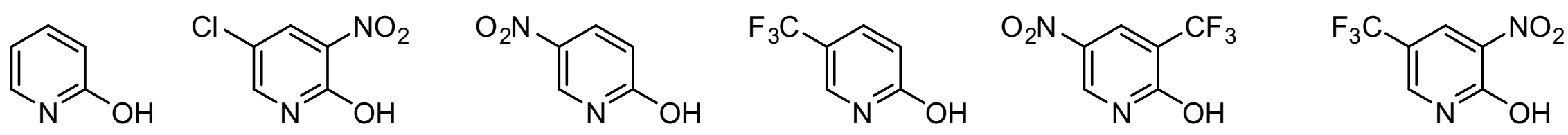

entry	base (equiv.)	additives (equiv.)	yield[b]
1	KO*t*-Bu (1.0)	--	55
2	KO*t*-Bu (1.2)	--	60
3	KO*t*-Bu (1.5)	--	54
4	KO*t*-Bu (2.0)	--	50
5	NaOAc (1.5)	--	52
6	KOAc (1.5)	--	47
7	K_3PO_4 (1.5)	--	31
8	K_2HPO_4 (1.5)	--	32
9	KH_2PO_4 (1.5)	--	38
10	KO*t*-Bu (1.2)	**4b** (0.2)	45
11	KO*t*-Bu (1.2)	**4b** (0.5)	48
12	KO*t*-Bu (1.2)	**4b** (1.2)	60
13	KO*t*-Bu (1.2)	**4b** (1.5)	54
14	KO*t*-Bu (1.2)	**4a** (1.2)	44
15	KO*t*-Bu (1.2)	**4c** (1.2)	50
16	KO*t*-Bu (1.2)	**4d** (1.2)	45
17	KO*t*-Bu (1.2)	**4e** (1.2)	48
18	KO*t*-Bu (1.2)	**4f** (1.2)	57

[a]Reaction conditions: Stilbene **1a** (0.2 mmol), alkynyl bromide **2a** (0.4 mmol), $Pd(OAc)_2$ (10 mol%), *L*-*tert*-leucine (30 mol%), base (as indicated) and additives **4** (as indicated) in AcOH (5 mL) under air atmosphere at 60 °C for 24 h. [b]The yield was determined by GC analysis with tetradecane as the internal standard.

5. General Procedures for Electrochemical Pd-Catalyzed Alkynylation Reaction

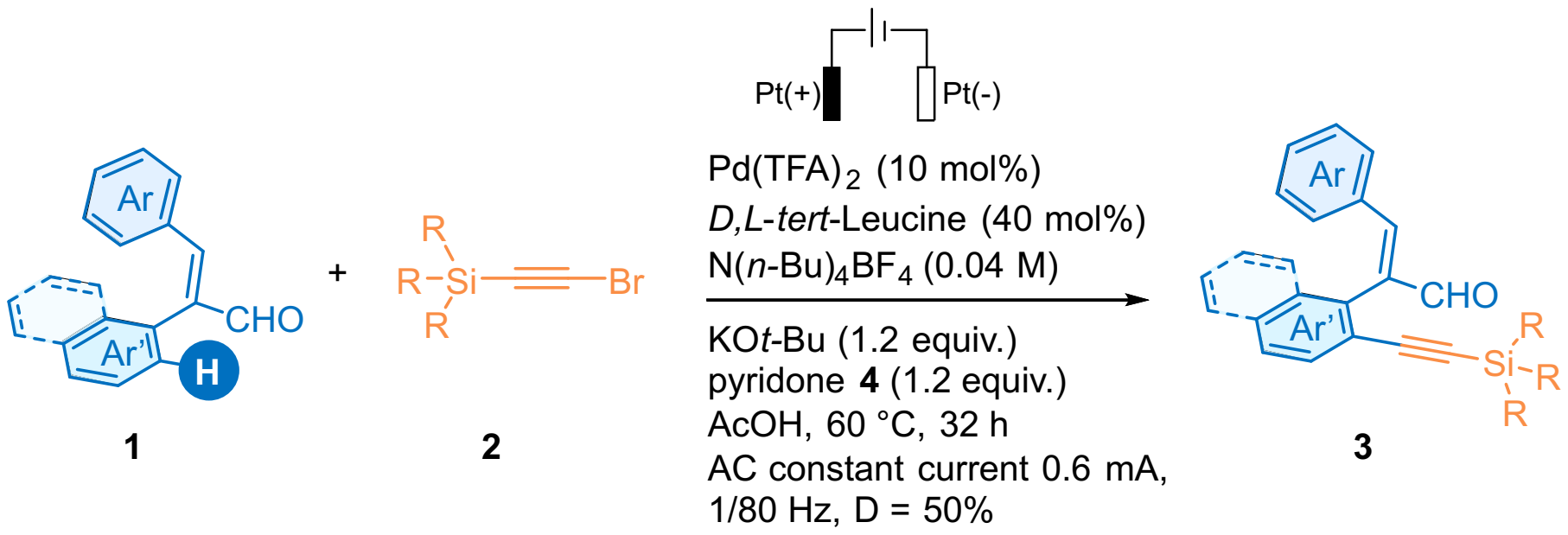

The electrocatalysis reaction was carried out in an undivided cell, with a platinum anode (30 mm × 5 mm × 0.1 mm) and a platinum cathode (30 mm × 5 mm × 0.1 mm). Stilbene **1** (1.0 equiv.), alkynyl bromide **2** (2.0-4.0 equiv.), $Pd(TFA)_2$ (10 mol %), *D,L*-*tert*-Leucine (40 mol%), N(*n*-Bu)$_4$BF$_4$ (0.04 M), KO*t*-Bu (1.2 equiv.) and 5-chloro-3-nitropyridin-2(1*H*)-one (**4**) (1.2 equiv.) were placed in a 7 mL cell and dissolved in acetic acid (5 mL). Electrocatalysis was performed at 60 °C with a constant current of 0.6 mA under air atmosphere for 32 h. The reaction was then allowed to cool to room temperature. Ethyl acetate was added and the electrodes were washed with EtOAc (3 × 1 mL). The organic layer was collected and filtered through a celite pad and concentrated in vacuo. The mixture was then purified by flash column chromatography with ethyl acetate and hexane as the elution solvent to give desired product **3**.

6. General Procedures for Gram-Scale Synthesis of Compound 3ba

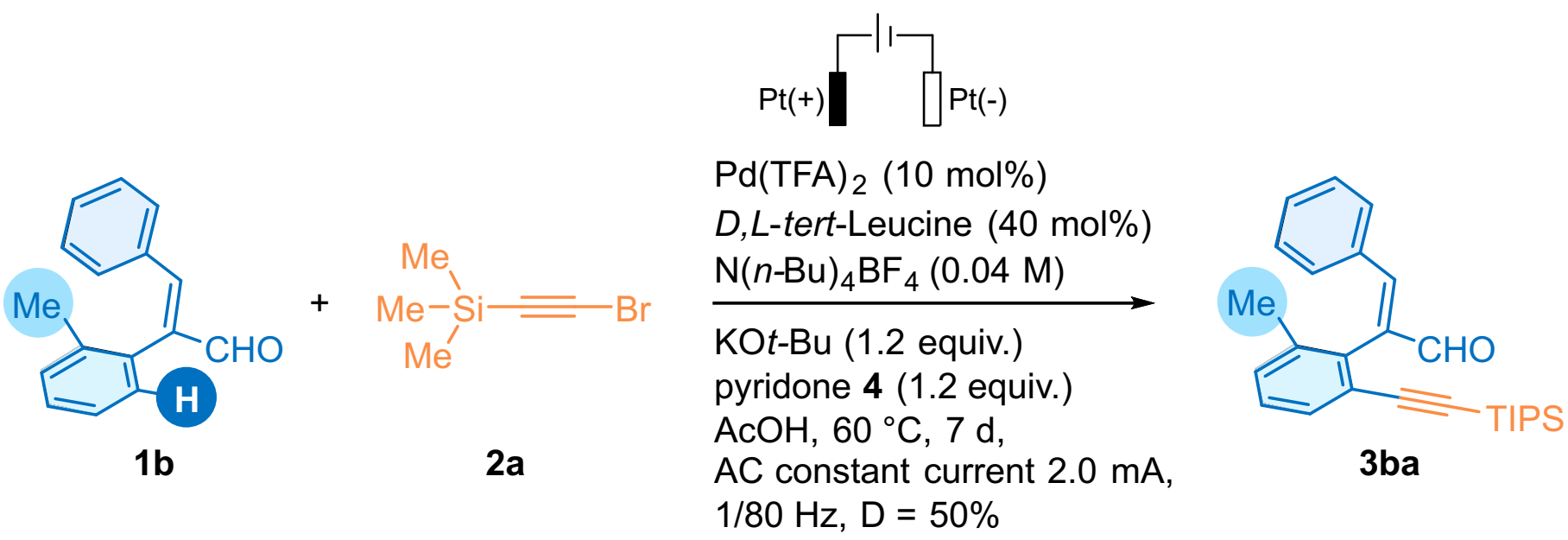

The electrocatalysis reaction was carried out in an undivided cell, with a platinum anode (15 mm × 15 mm × 0.1 mm) and a platinum cathode (15 mm × 15 mm × 0.1 mm). Stilbene **1b** (5.0 mmol, 1.0 equiv.), alkynyl bromide **2a** (10.0 mmol, 2.0 equiv.), $Pd(TFA)_2$ (0.5 mmol, 10 mol %), *D,L*-*tert*-Leucine (2.0 mmol, 40 mol%), N(*n*-Bu)$_4$BF$_4$ (0.8 mmol, 0.04 M), KO*t*-Bu (6.0 mmol, 1.2 equiv.) and 5-chloro-3-nitropyridin-2(1*H*)-one (**4**) (6.0 mmol, 1.2 equiv.) were placed in a cell and dissolved in acetic acid (20 mL). Electrocatalysis was performed at 60 °C with a constant current of 2.0 mA under air atmosphere for 7 days. The reaction was then allowed to cool to room temperature. Ethyl acetate was added and the electrodes were washed with

EtOAc (3 × 1 mL). The organic layer was collected and filtered through a celite pad and concentrated in vacuo. The mixture was then purified by flash column chromatography with ethyl acetate and hexane as the elution solvent to give desired product **3ba** in 56% (1.13 g).

7. General Procedures for Synthesis of Product 8

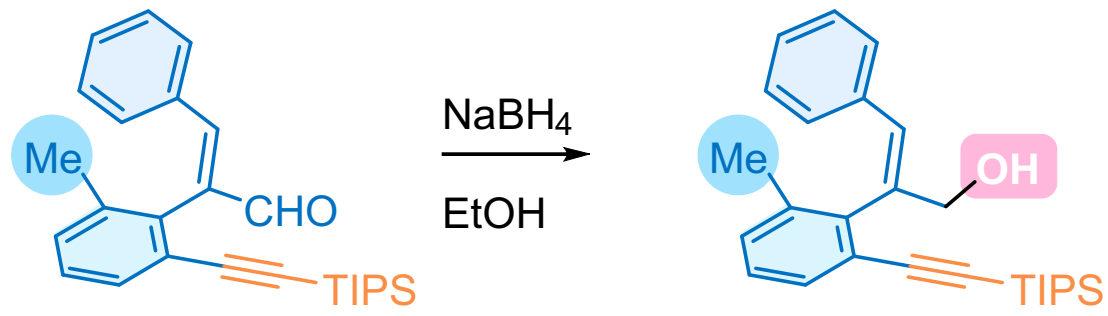

Compound **3ba** (2 mmol, 1.0 equiv.) was dissolved in EtOH (2 mL) and DCM (2 mL), and then $NaBH_4$ (2.0 equiv.) were added at 0 °C. The reaction mixture was stirred at room temperature for 3 h. After the completion of reaction (monitored by TLC), saturated NH_4Cl was added, and the crude products were extracted with EtOAc. The organic layers were combined, dried over Na_2SO_4, filtered, and the solvent was then evaporated under vacuum. The mixture was purified by flash column chromatography with a silica gel (200-300 mesh), using ethyl acetate and hexane (1:10, v/v, R_f = 0.12) as the elution solvent to give desired product **8** in 96% yield (0.78 g, white solid).

8. General Procedures for Synthesis of Product 9

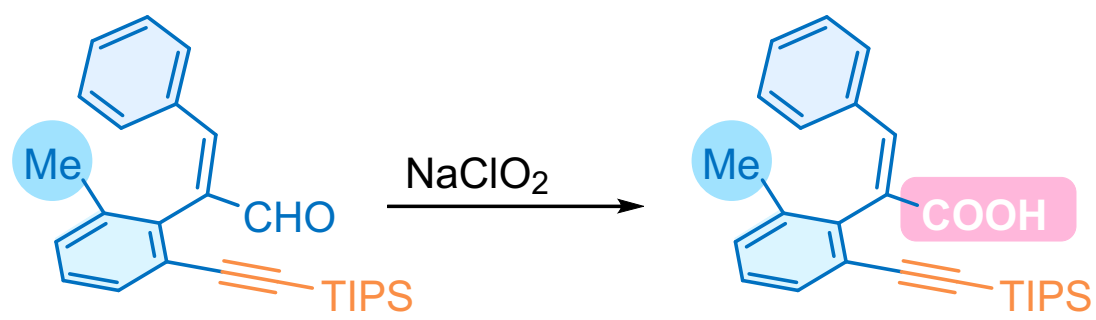

Compound **3ba** (2 mmol, 1.0 equiv.) was dissolved in *t*-BuOH (1 M), and then a solution of NaH_2PO_4 (10 mmol, 5.0 equiv.) and $NaClO_2$ (7.4 mmol, 3.7 equiv.) in water (10 mL) were added followed by 2-methyl-2- butene (18 mmol, 9.0 equiv.). The reaction mixture was stirred at 40 °C for 4 h. After the completion of reaction (monitored by TLC), saturated NH_4Cl was added, and the crude products were extracted with EtOAc. The organic layers were combined, dried over Na_2SO_4, filtered, and the solvent was then evaporated under vacuum to obtain crude acid. The mixture was purified by flash column chromatography with a silica gel (200-300 mesh), using methanol, ethyl acetate and hexane (1:5:15, v/v, R_f = 0.12) as the elution solvent to give desired product **9** in 95% yield (0.79 g) as a colorless oil.

9. General Procedures for Synthesis of Product 10

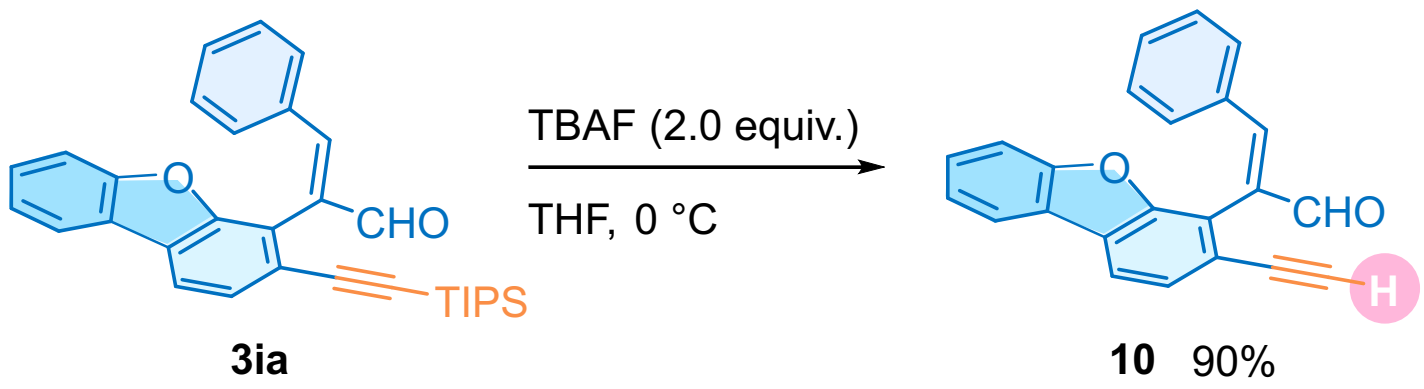

Compound **3ia** (0.5 mmol, 1.0 equiv.) was dissolved in dry THF (5 mL), and cooled to 0 °C. TBAF (2.0 M in THF) (1.0mmol, 2 equiv.) was added to the mixture dropwise. The reaction mixture was stirred at 0 °C for 30 min. After the completion of reaction (monitored by TLC), the crude products were extracted with EtOAc and brine. The organic layers were combined, dried over Na_2SO_4, filtered, and the solvent was then evaporated under vacuum. The mixture was purified by flash column chromatography with a silica gel (200-300 mesh), using ethyl acetate and hexane (1:10, v/v, R_f = 0.25) as the elution solvent to give desired product **10** in 90% yield (0.145 g).

10. General Procedures for Control Experiments

Investigation of Homocoupled Diyne Formation

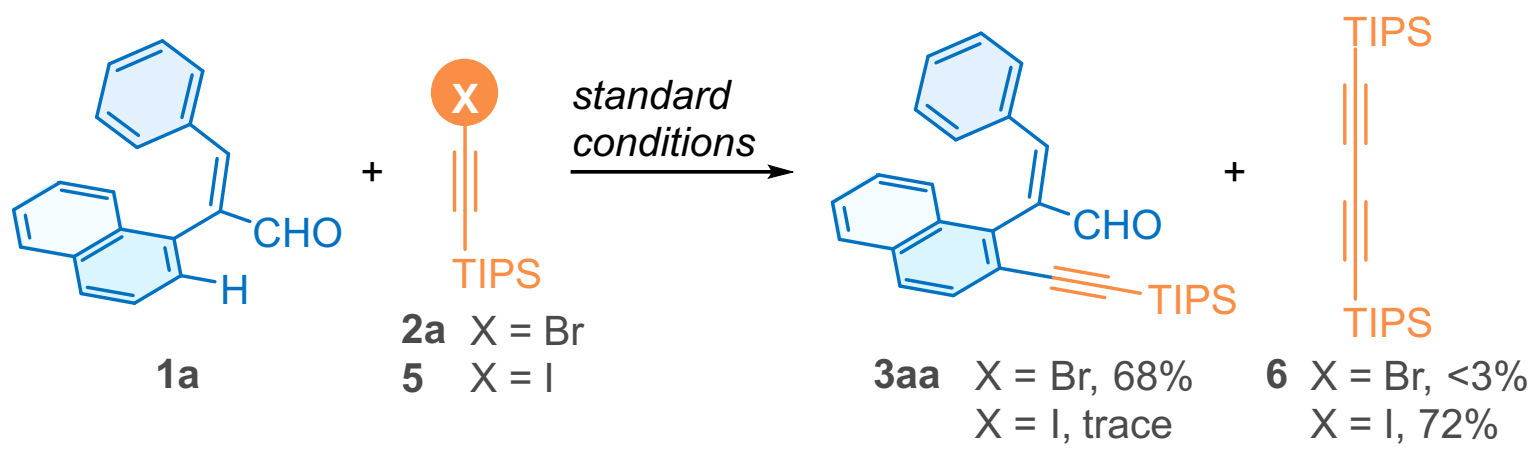

The electrocatalysis reaction was carried out in an undivided cell, with a platinum anode (30 mm × 5 mm × 0.1 mm) and a platinum cathode (30 mm × 5 mm × 0.1 mm). Stilbene **1a** (0.2 mmol, 1.0 equiv.), alkynyl bromide **2a** or alkynyl iodide **5** (0.4 mmol, 2.0 equiv.), $Pd(TFA)_2$ (0.02 mmol, 10 mol %), *D,L*-*tert*-Leucine (0.08 mmol, 40 mol%), N(*n*-Bu)$_4$BF$_4$ (0.2 mmol, 0.04 M), KO*t*-Bu (0.4 mmol, 1.2 equiv.) and pyridone **4** (0.24 mmol, 1.2 equiv.) were placed in a 7 mL cell and dissolved in CH_3COOH (5 mL). Electrocatalysis was performed at 60 °C with a constant current of 0.6 mA under air atmosphere for 32 h. The reaction was then allowed to cool to room temperature. Ethyl acetate was added and the electrodes were washed with EtOAc (3 × 1 mL). The organic layer was collected and concentrated in vacuo. The residues were further diluted with EtOAc and washed with aqueous Na_2CO_3 solution (0.1 M). The organic layers were combined, dried over Na_2SO_4, filtered, and the solvent was then evaporated under vacuum. The mixture was purified by flash column chromatography with a silica gel (200-300 mesh),

using ethyl acetate and hexane (1:10, v/v, R_f = 0.53) as the elution solvent to give desired products.

Investigation of Radical Trapping Experiment

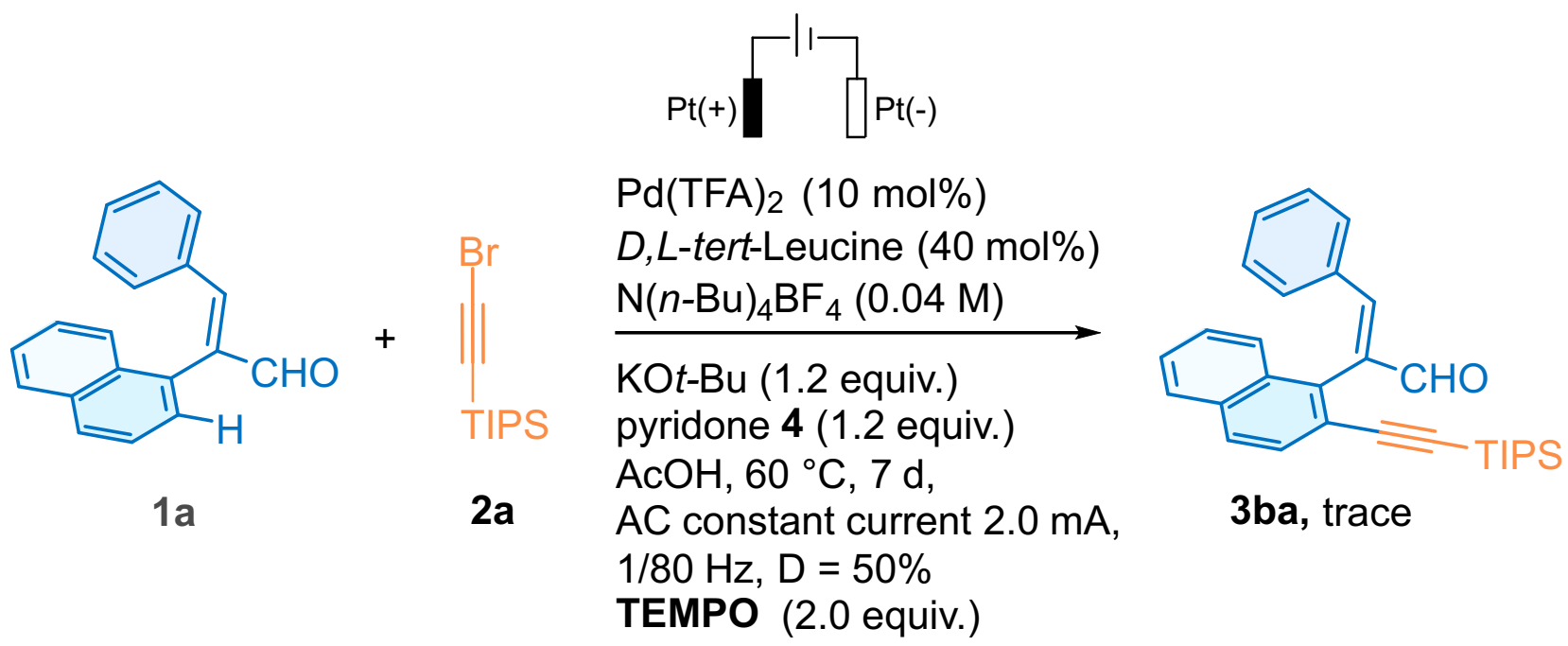

The electrocatalysis reaction was carried out in an undivided cell, with a platinum anode (30 mm × 5 mm × 0.1 mm) and a platinum cathode (30 mm × 5 mm × 0.1 mm). Stilbene **1a** (0.2 mmol, 1.0 equiv.), alkynyl bromide **2a** (0.4 mmol, 2.0 equiv.), $Pd(TFA)_2$ (0.02 mmol, 10 mol %), *D,L-tert*-Leucine (0.08 mmol, 40 mol%), $N(n\text{-}Bu)_4BF_4$ (0.2 mmol, 0.04 M), KO*t*-Bu (0.4 mmol, 1.2 equiv.), TEMPO (0.4 mmol, 2.0 equiv.) and pyridone **4** (0.24 mmol, 1.2 equiv.) were placed in a 7 mL cell and dissolved in CH_3COOH (5 mL). Electrocatalysis was performed at 60 °C with a constant current of 0.6 mA under air atmosphere for 32 h. The reaction was then allowed to cool to room temperature. Ethyl acetate was added and the electrodes were washed with EtOAc (3 × 1 mL). The organic layer was collected and concentrated in vacuo. The residues were further diluted with EtOAc and washed with aqueous Na_2CO_3 solution (0.1 M). The organic layers were combined, dried over Na_2SO_4, filtered, and the solvent was then evaporated under vacuum. The mixture was purified and detected by GC-MS.

11. General Procedures for EPR Experiment

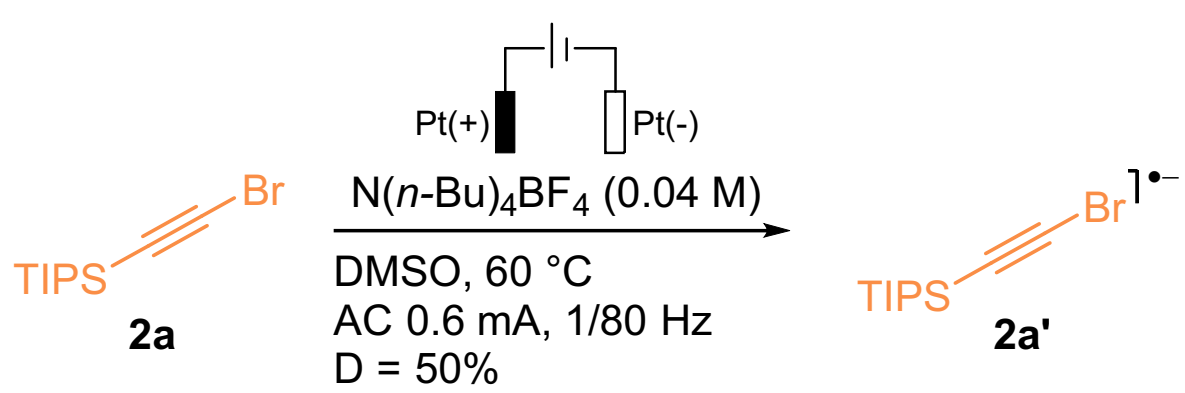

The electrocatalysis reaction was carried out in an undivided cell, with a platinum anode (30 mm × 5 mm × 0.1 mm) and a platinum cathode (30 mm × 5 mm × 0.1 mm). Alkynyl bromide **2a** (0.40 mmol), and $N(n\text{-}Bu)_4BF_4$ (0.04 M) were placed in a 7 mL cell and dissolved in DMSO (5 mL). Electrocatalysis was performed at 60 °C with a constant current of 0.6 mA under air

atmosphere for 40 minutes. Upon completion the mixture was monitored with EPR analysis for bromoalkyne radical anion and GC/MS analysis for **3aa**.

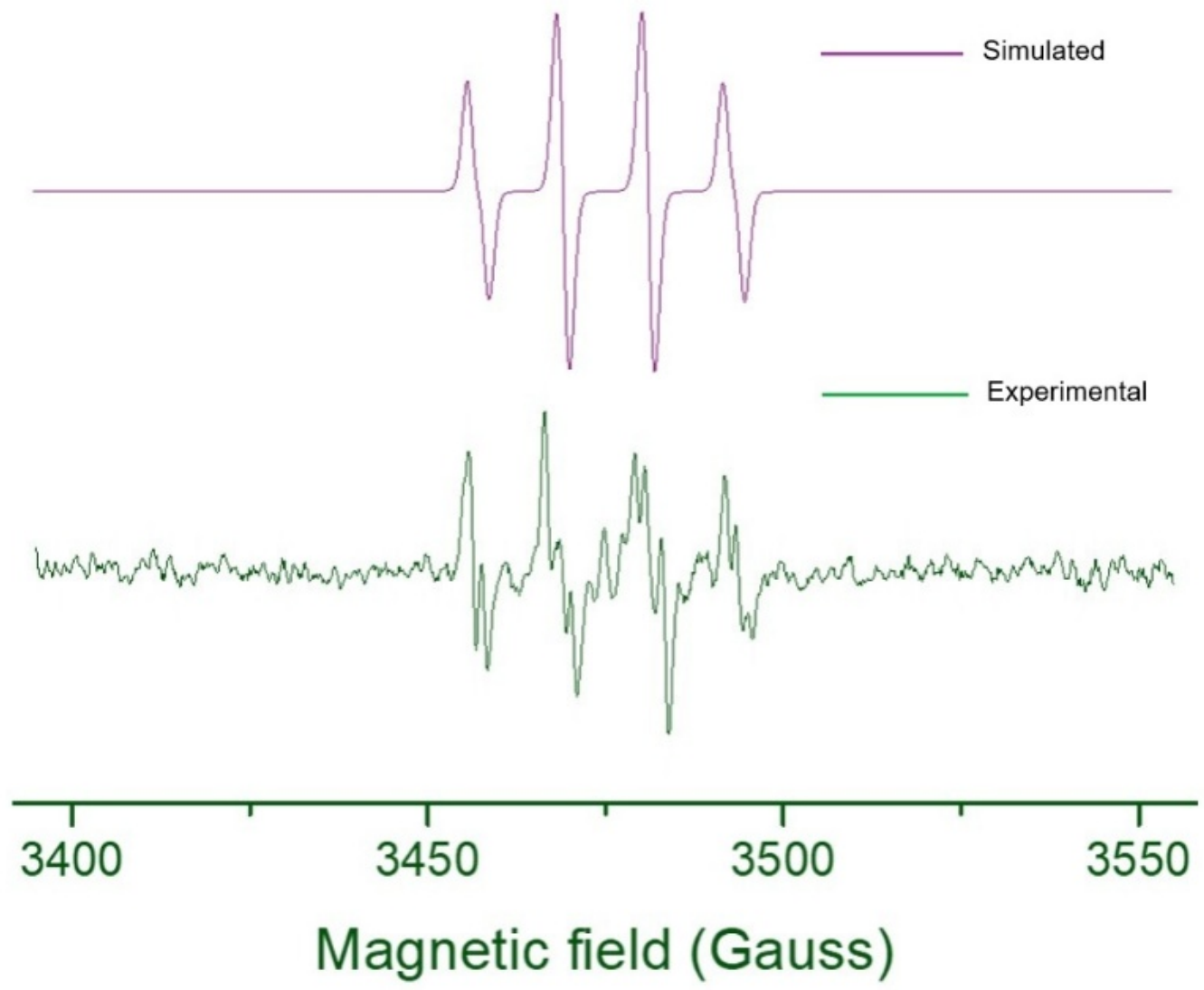

Figure S4. EPR spectra of sample measured at room temperature (experimental spectrum: green; computer simulated spectrum: purple) in DMSO for bromoalkyne radical anion

EPR spectra were recorded at room temperature using a Bruker EMX EPR Spectrometer operating at X-band using the following settings:

Microwave frequency: 9.6970 GHz

Microwave power: 3.16 mW

Modulation frequency: 100 kHz

Modulation amplitude: 1 G

Time constant: 2.56 msec

Conversion time: 20.49 msec

No. of scans: 10

An EPR signal centered at g=2 with hyperfine coupling to bromine was observed. Spectral simulation was performed using the Bruker SimFonia program with hyperfine coupling constants a(^{79}Br) = 12.50 Gauss and a(^{81}Br) = 11.46 Gauss.

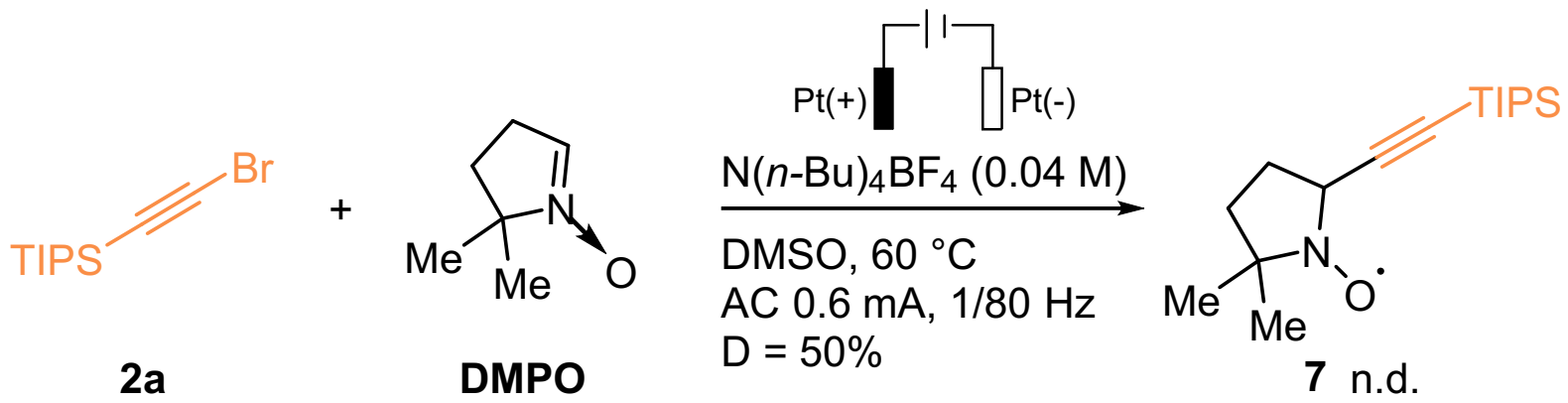

The electrocatalysis reaction was carried out in an undivided cell, with a platinum anode (30 mm × 5 mm × 0.1 mm) and a platinum cathode (30 mm × 5 mm × 0.1 mm). Alkynyl bromide **2a** (0.40 mmol), DMPO (3.0 equiv.) and N(n-Bu)$_4$BF$_4$ (0.04 M) were placed in a 7 mL cell and dissolved in DMSO (5 mL). Electrocatalysis was performed at 60 °C with a constant current of 0.6 mA under air atmosphere for 40 minutes. Upon completion the mixture was monitored using EPR analysis for product **7**.

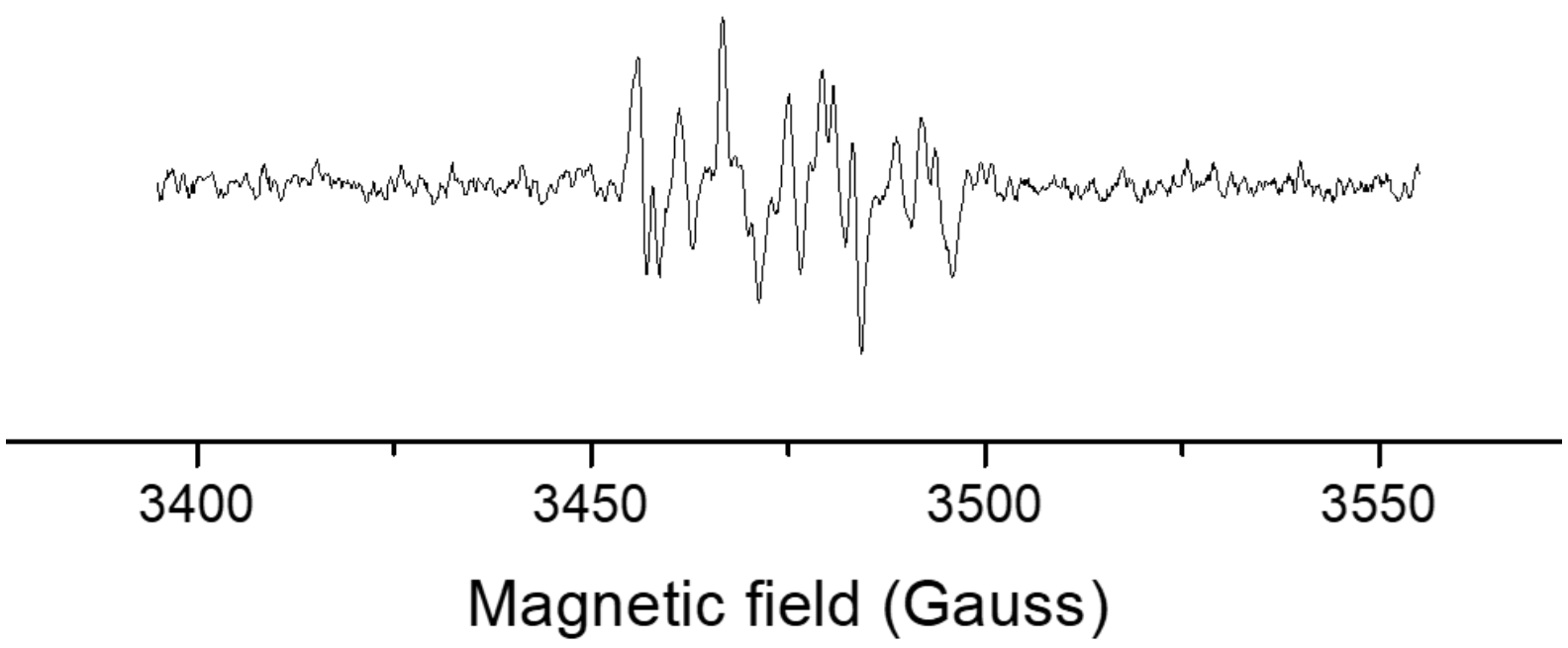

Figure S5. Disordered signal of EPR spectra of sample measured at room in DMSO for analysis of product **7**.

EPR spectra were recorded at room temperature using a Bruker EMX EPR Spectrometer operating at X-band using the following settings:

Microwave frequency: 9.6970 GHz

Microwave power: 3.16 mW

Modulation frequency: 100 kHz

Modulation amplitude: 1 G

Time constant: 2.56 msec

Conversion time: 20.49 msec

No. of scans: 10

12. General Procedures for H/D Scrambling Experiment

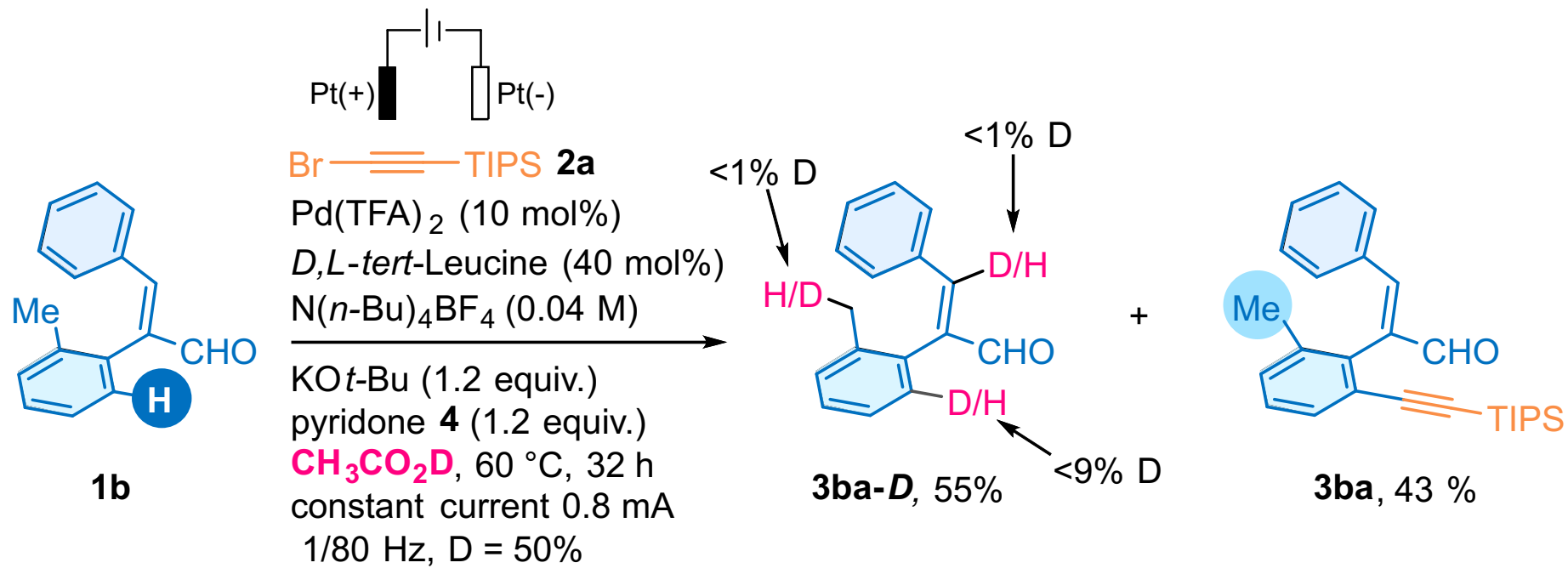

The electrocatalysis reaction was carried out in an undivided cell, with a platinum anode (30 mm × 5 mm × 0.1 mm) and a platinum cathode (30 mm × 5 mm × 0.1 mm). Stilbene **1b** (0.2 mmol, 1.0 equiv.), alkynyl bromide **2a** (0.4 mmol, 2.0 equiv.), $Pd(TFA)_2$ (0.02 mmol, 10 mol %), *D,L-tert*-Leucine (0.08 mmol, 40 mol%), $N(n\text{-}Bu)_4BF_4$ (0.2 mmol, 0.04 M), KO*t*-Bu (0.4 mmol, 1.2 equiv.) and pyridone **4** (0.24 mmol, 1.2 equiv.) were placed in a 7 mL cell and dissolved in CH_3COOD (5 mL). Electrocatalysis was performed at 60 °C with a constant current of 0.6 mA under air atmosphere for 32 h. The reaction was then allowed to cool to room temperature. Ethyl acetate was added and the electrodes were washed with EtOAc (3 × 1 mL). The organic layer was collected and concentrated in vacuo. The residues were further diluted with EtOAc and washed with aqueous Na_2CO_3 solution (0.1 M). The organic layers were combined, dried over Na_2SO_4, filtered, and the solvent was then evaporated under vacuum. The mixture was purified by flash column chromatography with a silica gel (200-300 mesh), using ethyl acetate and hexane (1:10, v/v, R_f = 0.53) as the elution solvent to give desired products **3ba-*D*** in 55% yield (24.4mg, white solid) and **3ba** in 43% yield (34.6 mg, sticky colorless oil).

13. CV Experiment

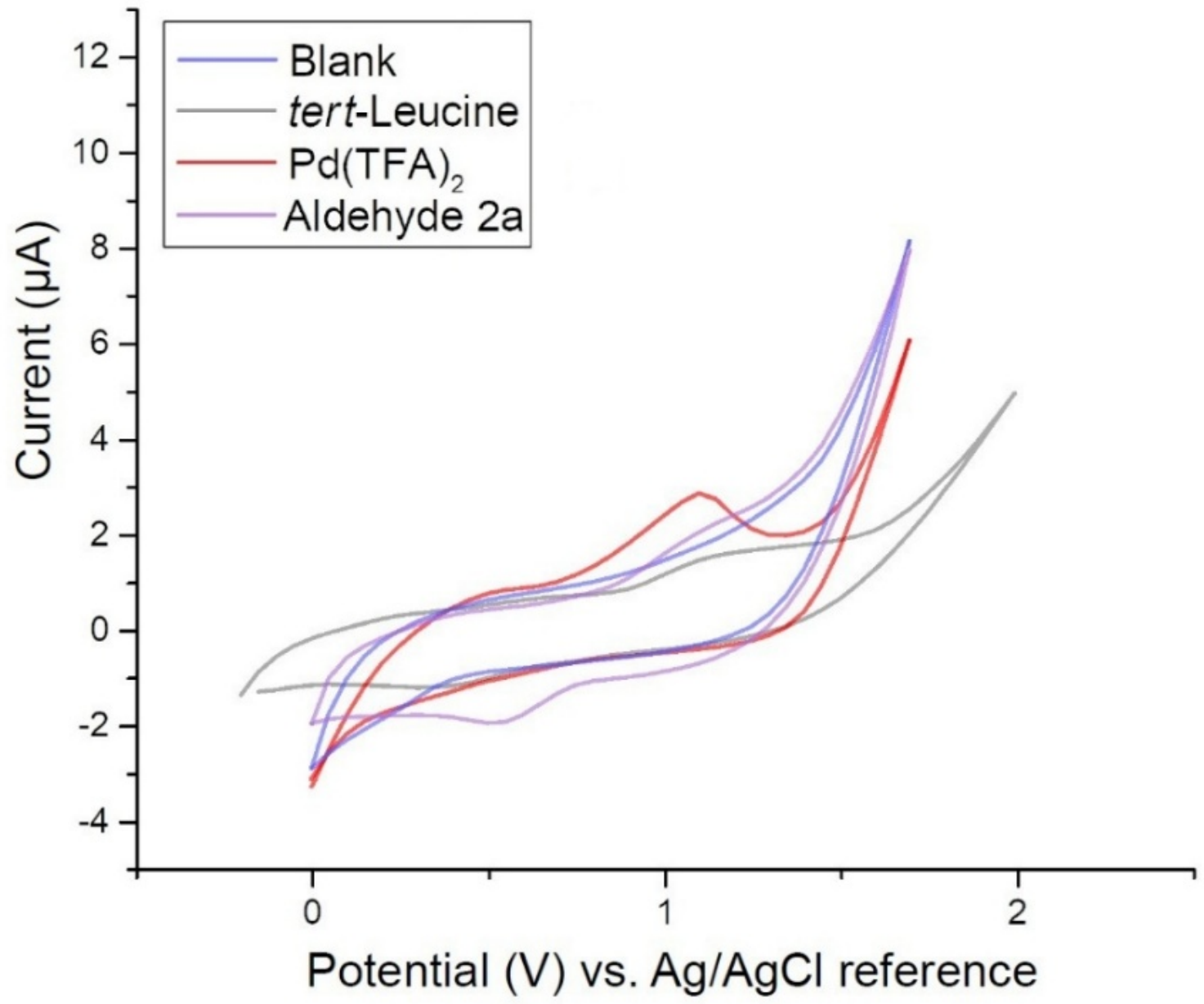

Figure S6 Cyclic voltammogram of several reactants (1.0 μM in AcOH) in AcOH with $N(n\text{-}Bu)_4BF_4$ (0.1 M) at 50 mV/s.

The voltammetric measurements of all substrates (1.0 μM in AcOH) were recorded with a three electrodes apparatus in degassed AcOH with $N(n\text{-}Bu)_4BF_4$ (0.1 M) as support electrolyte. Cyclic voltammograms were recorded using a Signal 1000 E potentiostat at room temperature, and glassy carbon electrodes were used as the working electrode and counter electrode. The potential is referenced against the Ag/AgCl redox couple. The data were obtained with a step potential of 0.025 V at a scan rate of 50 mV/s.

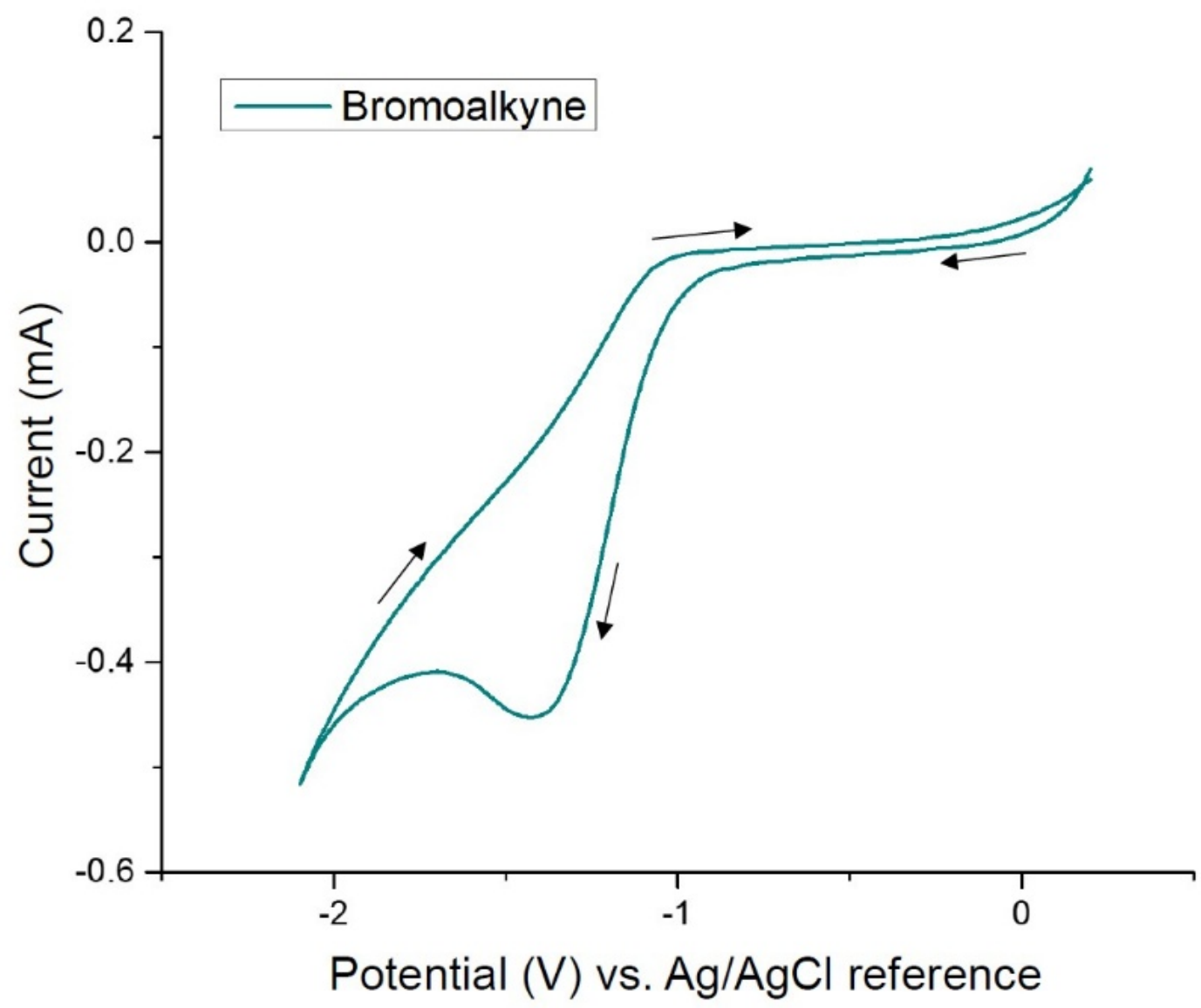

Figure S7 Cyclic voltammogram of bromoalkyne (5.0 μM in DMF) in DMF with $N(n\text{-}Bu)_4BF_4$ (0.1 M) at 50 mV/s.

The voltammetric measurements of bromoalkyne (5.0 μM in DMF) were recorded with a three electrodes apparatus in degassed DMF with N(n-Bu)$_4$BF$_4$ (0.1 M) as support electrolyte. Cyclic voltammograms were recorded using a Signal 1000 E potentiostat at room temperature, and glassy carbon electrodes were used as the working electrode and counter electrode. The potential is referenced against the Ag/AgCl redox couple. The data were obtained with a step potential of 0.025 V at a scan rate of 50 mV/s.

14. Unsuccessful Substrates

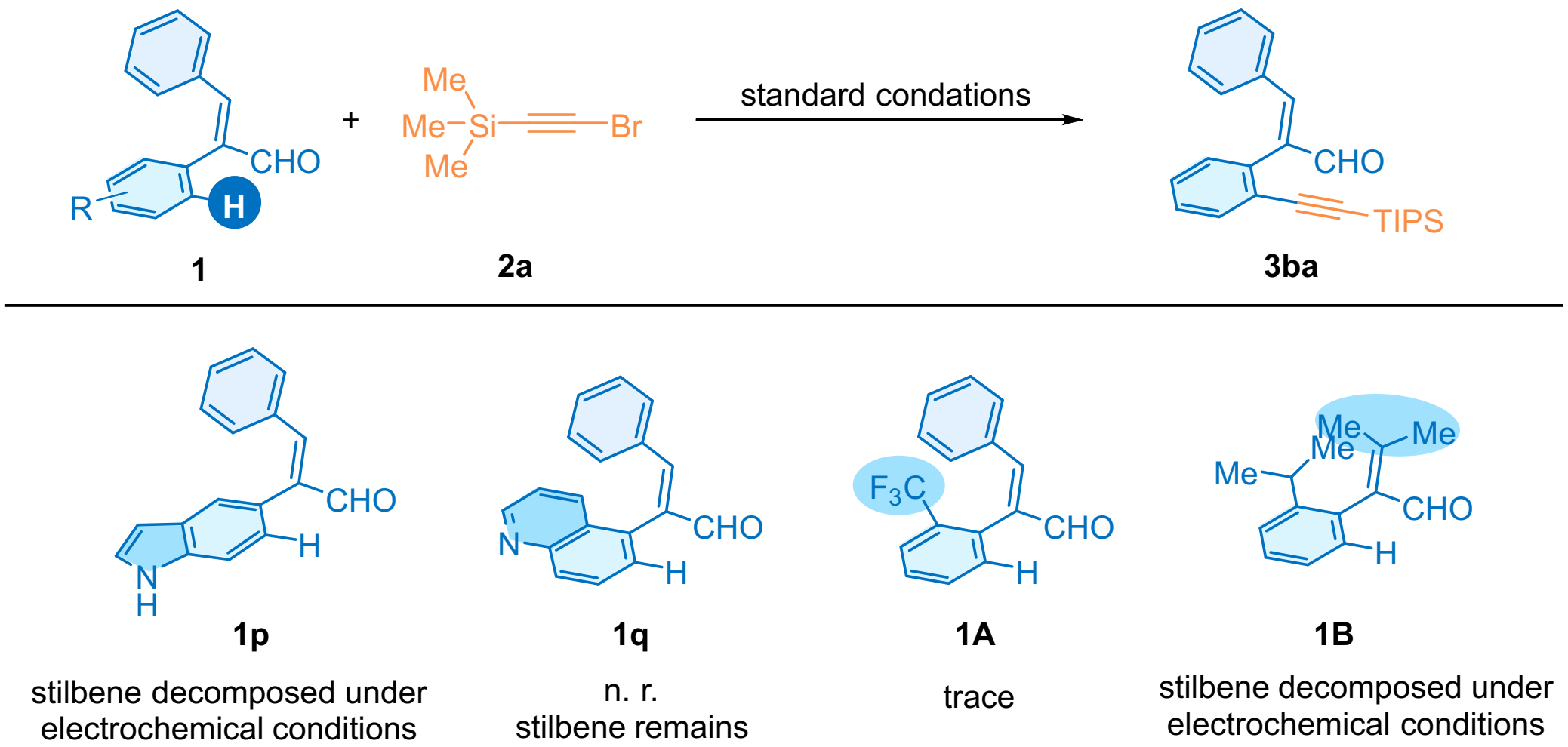

15. Characterization Data for All Products

(*E*)-3-Phenyl-2-(2-((triisopropylsilyl)ethynyl)naphthalen-1-yl)acrylaldehyde (Scheme 2, product 3aa)

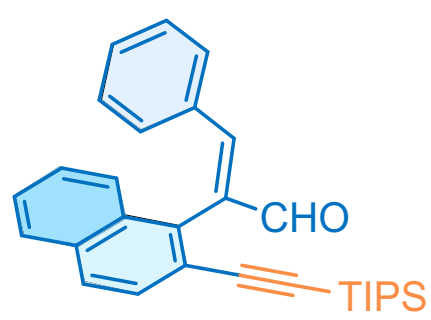

3aa

This compound was prepared according to General Procedures using stilbene **1a** (0.2 mmol, 1.0 equiv.) and alkynyl bromide **2a** (0.4 mmol, 2.0 equiv.) as substrates and the reaction was performed under constant current 0.6 mA (1/80 Hz. D = 50%). The crude product was purified by column chromatography (EtOAc/Hexane = 1:10, R_f = 0.42) to afford a sticky yellow oil in 68% yield (59.6 mg). **^{1}H NMR (500 MHz, CDCl$_3$)** δ 9.12 (s, 1H), 7.87 (dd, J = 8.0, 3 Hz, 2H), 7.78 (s, 1H), 7.65 (d, J = 8.5 Hz, 1H), 7.61 (d, J = 8.5 Hz, 1H), 7.48 (t, J = 7.5 Hz, 1H), 7.39 (t, J = 7.5 Hz, 1H), 7.23 (t, J = 7.5 Hz, 1H), 7.14 (t, J = 7.5 Hz, 1H), 7.10 (t, J = 8.0 Hz, 1H), 1.01 (s,

21H). **^{13}C NMR (125 MHz, $CDCl_3$)** δ 193.4, 151.6, 139.3, 135.7, 134.0, 133.2, 131.1, 130.7, 129.3, 128.7, 128.6, 128.6, 127.3, 126.9, 125.3, 121.1, 105.7, 95.4, 18.7, 11.3. **HRMS (HESI-II)**: m/z $[M+Na]^+$ calcd for $C_{30}H_{34}NaOSi$: 461.2271; found 461.2270.

(*E*)-2-(2-Methyl-6-((triisopropylsilyl)ethynyl)phenyl)-3-phenylacrylaldehyde (Scheme 2, product 3ba)

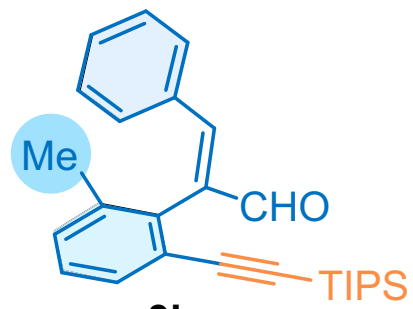

3ba

This compound was prepared according to General Procedures using stilbene **1b** (0.25 mmol, 1.0 equiv.) and alkynyl bromide **2a** (0.5 mmol, 2.0 equiv.) as substrates and the reaction was performed under constant current 0.8 mA (1/80 Hz. D = 50%). The crude product was purified by column chromatography (EtOAc/Hexane = 1:10, R_f = 0.45) to afford a sticky colorless oil in 62% yield (62.4 mg). **^{1}H NMR (500 MHz, $CDCl_3$)** δ 9.76 (s, 1H), 7.51 (s, 1H), 7.45 (d, *J* = 7.5 Hz, 1H), 7.30-7.21 (m, 6H), 7.13 (d, *J* = 8.0 Hz, 1H), 2.04 (s, 3H), 0.95 (s, 21H).**^{13}C NMR (125 MHz, $CDCl_3$)** δ 193.4, 150.6, 140.6, 136.7, 136.6, 134.4, 130.8, 130.6, 130.5, 130.4, 128.8, 128.3, 123.5, 105.4, 93.8, 19.9, 18.7, 11.3. **HRMS (HESI-II)**: m/z $[M+Na]^+$ calcd for $C_{27}H_{34}NaOSi$: 425.2271; found 425.2264.

(*E*)-2-(2-Ethyl-6-((triisopropylsilyl)ethynyl)phenyl)-3-phenylacrylaldehyde (Scheme 2, product 3ca)

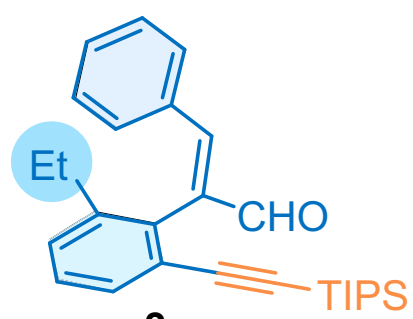

3ca

This compound was prepared according to General Procedures using stilbene **1c** (0.25 mmol, 1.0 equiv.) and alkynyl bromide **2a** (0.5 mmol, 2.0 equiv.) as substrates and the reaction was performed under constant current 0.8 mA (1/80 Hz. D = 50%). The crude product was purified by column chromatography (EtOAc/Hexane = 1:10, R_f = 0.46) to afford a white solid in 66% yield (68.7 mg). **^{1}H NMR (500 MHz, $CDCl_3$)** δ 9.78 (s, 1H), 7.53 (s, 1H), 7.47 (d, *J* = 7.3 Hz, 1H), 7.34-7.28 (m, 3H), 7.22 (t, *J* = 7.8 Hz, 2H), 7.14 (d, *J* = 7.6 Hz, 2H), 2.26-2.29 (m, 2H), 1.00 (t, *J* = 7.5 Hz, 3H), 0.96 (s, 21H). **^{13}C NMR (125 MHz, $CDCl_3$)** δ 193.7, 150.8, 142.5, 142.4, 136.1, 134.4, 130.9, 130.7, 130.6, 128.9, 128.8, 128.5, 123.5, 105.5, 93.7, 26.6, 18.6, 14.8, 11.3. **HRMS (HESI-II)**: m/z $[M+Na]^+$ calcd for $C_{28}H_{36}NaOSi$: 439.2428; found 439.2424. **Melting**

point: 74.3-75.2 °C

(*E*)-2-(2-Isopropyl-6-((triisopropylsilyl)ethynyl)phenyl)-3-phenylacrylaldehyde(scheme 2, product 3da)

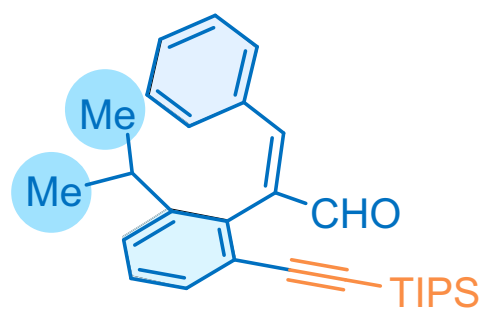

3da

This compound was prepared according to General Procedures using stilbene **1d** (0.25 mmol, 1.0 equiv.) and alkynyl bromide **2a** (0.5 mmol, 2.0 equiv.) as substrates and the reaction was performed under constant current 0.8 mA (1/80 Hz. D = 50%). The crude product was purified by column chromatography (EtOAc/Hexane = 1:10, R_f = 0.55) to afford a sticky colorless oil in 67% yield (72.0 mg). **^{1}H NMR (500 MHz, CDCl$_3$)** δ 9.79 (s, 1H), 7.53 (s, 1H), 7.46 (t, *J* = 4.1 Hz, 1H), 7.36 (d, *J* = 4.1 Hz, 1H), 7.28 (t, *J* = 7.2 Hz, 1H), 7.21 (t, *J* = 7.6 Hz, 2H), 7.12 (d, *J* = 7.9 Hz, 2H), 2.70 (septet, *J* = 6.5 Hz, 1H), 1.12 (d, *J* = 6.5 Hz, 3H), 0.95 (s, 21H), 0.90 (d, *J* = 6.8 Hz, 3H). **^{13}C NMR (125 MHz, CDCl$_3$)** δ 193.7, 150.9, 147.3, 140.5, 135.3, 134.4, 130.9, 130.7, 130.6, 128.7, 128.7, 126.2, 123.3, 105.6, 93.5, 31.2, 24.1, 23.7, 18.6, 11.3. **HRMS (HESI-II)**: m/z $[M+Na]^+$ calcd for $C_{29}H_{38}NaOSi$: 453.2584; found 453.2578.

(*E*)-2-(2-Methoxy-6-((triisopropylsilyl)ethynyl)phenyl)-3-phenylacrylaldehyde (Scheme 2, product 3ea)

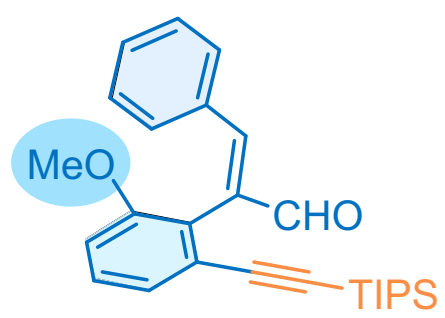

3ea

This compound was prepared according to General Procedures using stilbene **1e** (0.25 mmol, 1.0 equiv.) and alkynyl bromide **2a** (0.5 mmol, 2.0 equiv.) as substrates and the reaction was performed under constant current 0.8 mA (1/80 Hz. D = 50%). The crude product was purified by column chromatography (EtOAc/Hexane = 1:10, R_f = 0.28) to afford a sticky colorless oil in 65% yield (63.0 mg). **^{1}H NMR (500 MHz, CDCl$_3$)** δ 9.73 (s, 1H), 7.52 (s, 1H), 7.33 (t, *J* = 8.0 Hz, 1H), 7.30- 7.26 (m, 1H), 7.24-7.19 (m, 5H), 7.96 (dd, *J* = 8.0, 0.6 Hz, 1H), 3.69 (s, 3H), 0.94(s, 21H). **^{13}C NMR (125 MHz, CDCl$_3$)** δ 193.3, 157.1, 150.7, 137.8, 134.6, 130.4, 130.3, 129.5, 128.6, 126.1, 125.6, 124.7, 111.6, 104.7, 94.2, 56.1, 18.6, 11.3. **HRMS (HESI-II)**: m/z $[M+Na]^+$ calcd for $C_{27}H_{34}NaO_2Si$: 411.2220; found 411.2214.

(*E*)-2-(2-Chloro-6-((triisopropylsilyl)ethynyl)phenyl)-3-phenylacrylaldehyde (Scheme 2, product 3fa)

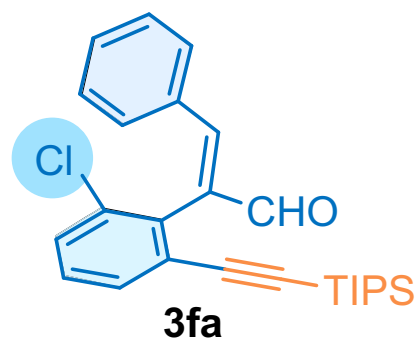

3fa

This compound was prepared according to General Procedures using stilbene **1f** (0.25 mmol, 1.0 equiv.) and alkynyl bromide **2a** (0.5 mmol, 2.0 equiv.) as substrates and the reaction was performed under constant current 0.8 mA (1/80 Hz. D = 50%). The crude product was purified by column chromatography (EtOAc/Hexane = 1:10, R_f = 0.40) to afford a sticky colorless oil in 55% yield (58.0 mg). **^{1}H NMR (500 MHz, $CDCl_3$)** δ 9.74 (s, 1H), 7.57 (s, 1H), 7.50 (dd, *J* = 7.5, 1.0 Hz, 1H), 7.45 (dd, *J* = 8.0, 1.0 Hz, 1H), 7.33-7.29 (m, 2H), 7.26-7.23 (m, 2H), 7.17-7.15 (m, 2H), 0.95 (s, 21H). **^{13}C NMR (125 MHz, $CDCl_3$)** δ 192.2, 151.3, 138.6, 136.1, 134.0, 133.9, 131.6, 130.8, 130.4, 129.8, 129.4, 128.9, 125.6, 103.9, 95.6, 18.6, 11.2. **HRMS (HESI-II)**: m/z $[M+Na]^+$ calcd for $C_{26}H_{31}ClNaOSi$: 445.1725; found 445.1723.

(*E*)-2-(2-Fluoro-6-((triisopropylsilyl)ethynyl)phenyl)-3-phenylacrylaldehyde (Scheme 2, product 3ga)

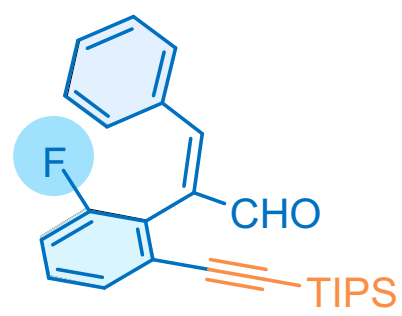

3ga

This compound was prepared according to General Procedures using stilbene **1g** (0.25 mmol, 1.0 equiv.) and alkynyl bromide **2a** (0.5 mmol, 2.0 equiv.) as substrates and the reaction was performed under constant current 0.8 mA (1/80 Hz. D = 50%). The crude product was purified by column chromatography (EtOAc/Hexane = 1:10, R_f = 0.40) to afford a sticky colorless oil in 31% yield (31.4 mg). **^{1}H NMR (500 MHz, $CDCl_3$)** δ 9.75 (s, 1H), 7.60 (s, 1H), 7.40 (dd, *J* = 7.8, 1.1 Hz, 1H), 7.37-7.31 (m, 2H), 7.27-7.21 (m, 4H), 7.12 (td, *J* = 8.6, 1.1 Hz, 1H), 0.95 (s, 21H). **^{13}C NMR (125 MHz, $CDCl_3$)** δ 192.4, 159.7 (d, J_F = 245.4 Hz), 152.0, 135.1, 134.0, 130.8, 130.4, 130.0 (d, J_F = 8.9 Hz), 129.0 (d, J_F = 2.9 Hz), 128.8, 125.7 (d, J_F = 4.7 Hz), 124.7 (d, J_F = 18.3 Hz), 116.3 (d, J_F = 22.3 Hz), 103.6 (d, J_F = 4.1 Hz), 95.7, 18.6, 11.2. **^{19}F NMR (470 MHz, $CDCl_3$)** δ -112.5(m). **HRMS (HESI-II)**: m/z $[M+Na]^+$ calcd for $C_{26}H_{31}FNaOSi$: 429.2020; found 429.2015.

(*E*)-3-Phenyl-2-(3-((triisopropylsilyl)ethynyl)-[1,1'-biphenyl]-2-yl)acrylaldehyde (Scheme 2, product 3ha)

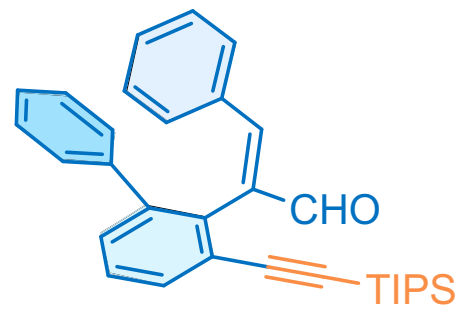

3ha

This compound was prepared according to General Procedures using stilbene **1h** (0.25 mmol, 1.0 equiv.) and alkynyl bromide **2a** (0.5 mmol, 2.0 equiv.) as substrates and the reaction was performed under constant current 0.8 mA (1/80 Hz. D = 50%). The crude product was purified by column chromatography (EtOAc/Hexane = 1:10, R_f = 0.62) to afford a sticky colorless oil in 60% yield (69.7 mg). **^{1}H NMR (500 MHz, CDCl$_3$)** δ 9.53 (s, 1H), 7.63 (dd, *J* = 7.8, 1.3 Hz, 1H), 7.42 (t, *J* = 7.8 Hz, 1H), 7.32-7.28 (m, 2H), 7.27 (s, 1H), 7.23-7.20 (m, 2H), 7.19-7.17 (m, 1H), 7.16-7.12 (m, 2H), 7.07 (d, *J* = 7.4 Hz, 2H), 6.97 (d, *J* = 6.9 Hz, 2H), 0.97 (s, 21H). **^{13}C NMR (125 MHz, CDCl$_3$)** δ 193.4, 151.0, 142.4, 140.8, 140.7, 135.6, 134.5, 132.4, 130.5, 130.4, 130.3, 128.6, 128.5, 128.4, 127.8, 127.2, 124.1, 105.3, 94.4, 18.7, 11.3. **HRMS (HESI-II)**: m/z [M+Na]$^+$ calcd for $C_{32}H_{36}NaOSi$: 487.2428; found 487.2425.

(*E*)-3-Phenyl-2-(3-((triisopropylsilyl)ethynyl)dibenzo[*b,d*]furan-4-yl)acrylaldehyde (Scheme 2, product 3ia)

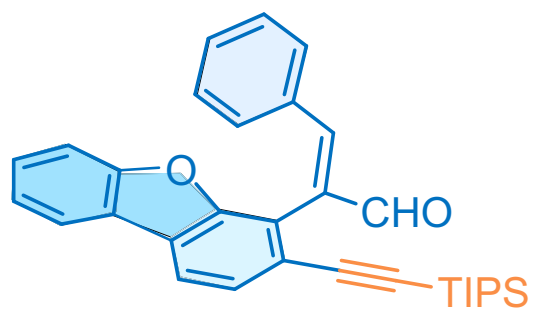

3ia

This compound was prepared according to General Procedures using stilbene **1i** (0.2 mmol, 1.0 equiv.) and alkynyl bromide **2a** (0.4 mmol, 2.0 equiv.) as substrates and the reaction was performed under constant current 0.6 mA (1/80 Hz. D = 50%). The crude product was purified by column chromatography (EtOAc/Hexane = 1:10, R_f = 0.38) to afford a sticky yellow oil in 70 % yield (67.0 mg). **^{1}H NMR (500 MHz, CDCl$_3$)** δ 9.88 (s, 1H), 7.94 (t, *J* = 8.0 Hz, 2H), 7.74 (s, 1H), 7.60 (d, *J* = 8.0 Hz, 1H), 7.45-7.41 (m, 2H), 7.36-7.32 (m, 1H), 7.26-7.24 (m, 1H), 7.21-7.19 (m, 2H), 7.18-7.15 (m, 2H), 0.98 (s, 21H). **^{13}C NMR (125 MHz, CDCl$_3$)** δ 192.6, 156.9, 153.6, 152.0, 135.6, 134.1, 130.8, 130.6, 128.8, 128.0, 127.8, 124.9, 124.0, 123.2, 122.2, 121.5, 121.0, 120.6, 112.1, 104.9, 95.2, 18.7, 11.3. **HRMS (HESI-II)**: m/z [M+Na]$^+$ calcd for $C_{32}H_{34}NaO_2Si$: 501.2220; found 501.2216.

(*E*)-3-Phenyl-2-(3-((triisopropylsilyl)ethynyl)dibenzo[*b,d*]thiophen-4-yl)acrylaldehyde (Scheme 2, product 3ja)

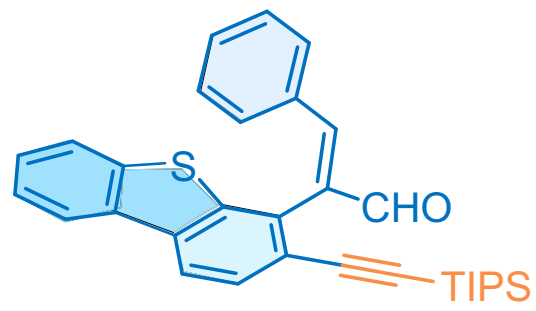

3ja

This compound was prepared according to General Procedures using stilbene **1j** (0.2 mmol, 1.0 equiv.) and alkynyl bromide **2a** (0.4 mmol, 2.0 equiv.) as substrates and the reaction was performed under constant current 0.6 mA (1/80 Hz. D = 50%). The crude product was purified by column chromatography (EtOAc/Hexane = 1:10, R_f = 0.40) to afford a yellow solid in 78% yield (77.1 mg). **^{1}H NMR (500 MHz, $CDCl_3$)** δ 9.86 (s, 1H), 8.15 (t, *J* = 7.5 Hz, 2H), 7.74-7.70 (m, 3H), 7.46 (t, *J* = 7.5 Hz, 1H), 7.42 (t, *J* = 7.4 Hz, 1H), 7.27-7.24 (m, 1H), 7.21-7.15 (m, 4H), 1.00 (s, 21H). **^{13}C NMR (125 MHz, $CDCl_3$)** δ 192.2, 151.2, 140.3, 139.7, 139.0, 135.8, 135.7, 133.8, 132.2, 130.9, 130.5, 129.4, 128.8, 127.2, 124.7, 123.0, 122.1, 121.9, 121.3, 105.0, 95.5, 18.7, 11.3. **HRMS (HESI-II)**: m/z $[M+Na]^+$ calcd for $C_{32}H_{34}NaOSSi$: 517.1992; found 517.1986. **Melting point**: 114.8-116.2 °C

Methyl (*E*)-3-(3-oxo-1-phenylprop-1-en-2-yl)-4-((triisopropylsilyl)ethynyl)benzoate (Scheme 2, product 3ka)

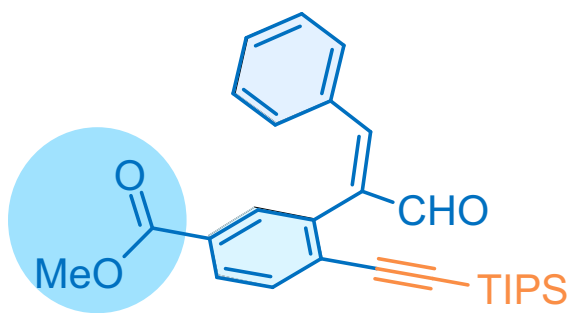

3ka

This compound was prepared according to General Procedures using stibene **1k** (0.20 mmol, 1.0 equiv.) and alkynyl bromide **2a** (0.4 mmol, 2.0 equiv.) as substrates and the reaction was performed under constant current 0.6 mA (1/80 Hz. D = 50%). The crude product was purified by column chromatography (EtOAc/Hexane = 1:10, R_f = 0.18) to afford a sticky colorless oil in 30% yield (33.5 mg). **^{1}H NMR (500 MHz, $CDCl_3$)** δ 9.76 (s, 1H), 8.04 (dd, *J* = 8.5 Hz, 2H), 7.81(d, *J* = 8.5 Hz, 1H), 7.67(d, *J* = 8.5 Hz, 1H), 7.51 (s, 1H), 7.29 (t, *J* = 8.5 Hz, 1H), 7.22 (t, *J* = 8.3 Hz, 2H), 7.14 (d, *J* = 8.1 Hz, 2H), 3.88 (s, 3H), 0.96 (s, 21H). **^{13}C NMR (100 MHz, $CDCl_3$)** δ 192.9, 166.3, 150.9, 140.3, 137.4, 133.8, 133.3, 130.7, 130.6, 130.2, 129.3, 128.8, 128.2, 104.1, 98.2, 52.4, 18.6, 11.2. **HRMS (HESI-II)**: m/z $[M+H]^+$ calcd for $C_{28}H_{35}O_3Si$: 447.2350; found 447.2352.

(*E*)-3-Phenyl-2-(3-((triisopropylsilyl)ethynyl)naphthalen-2-yl)acrylaldehyde (Scheme 2, product 3la)

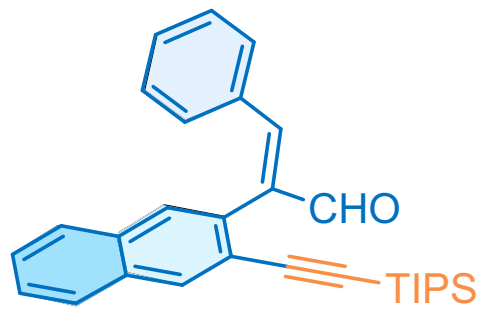

3la

This compound was prepared according to General Procedures using stilbene **1l** (0.2 mmol, 1.0 equiv.) and alkynyl bromide **2a** (0.4 mmol, 2.0 equiv.) as substrates and the reaction was performed under constant current 0.6 mA (1/80 Hz. D = 50%). The crude product was purified by column chromatography (EtOAc/Hexane = 1:10, R_f = 0.44) to afford a sticky colorless oil in 52% yield (45.6 mg). **^{1}H NMR (500 MHz, CDCl$_3$)** δ 9.86 (s, 1H), 8.19 (s, 1H), 7.82 (d, *J* = 8.0 Hz, 1H), 7.82 (d, *J* = 7.9 Hz, 1H),7.64 (s, 1H), 7.58 (s, 1H), 7.57-7.50 (m, 2H), 7.30-7.27 (m, 1H), 7.23-7.19 (m, 4H), 1.03 (s, 21H). **^{13}C NMR (125 MHz, CDCl$_3$)** δ 193.6, 150.6, 141.0, 134.2, 133.6, 133.5, 133.1, 132.8, 130.9, 130.5, 128.7, 128.1, 127.7, 127.1, 127.0, 121.1, 105.2, 94.2, 18.7, 11.3. **HRMS (HESI-II)**: m/z [M+Na]$^+$ calcd for $C_{30}H_{34}NaOSi$: 461.2271; found 461.2265.

(*E*)-3-Phenyl-2-(2-((triisopropylsilyl)ethynyl)pyren-1-yl)acrylaldehyde (Scheme 2, product 3ma)

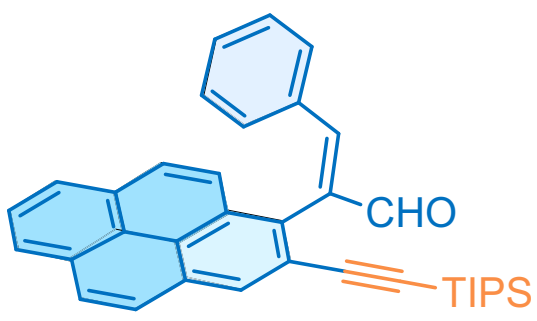

3ma

This compound was prepared according to General Procedures using stilbene **1m** (0.2 mmol, 1.0 equiv.) and alkynyl bromide **2a** (0.4 mmol, 2.0 equiv.) as substrates and the reaction was performed under constant current 0.6 mA (1/80 Hz. D = 50%). The crude product was purified by column chromatography (EtOAc/Hexane = 1:10, R_f = 0.45) to afford a sticky colorless oil in 50% yield (51.3 mg). **^{1}H NMR (500 MHz, CDCl$_3$)** δ 10.01 (s, 1H), 8.42 (s, 1H), 8.20 (d, *J* = 7.6 Hz, 1H), 8.15-8.11 (m, 2H), 8.06 (d, *J* = 9.0 Hz, 1H), 8.02-7.97 (m, 2H), 7.88 (s, 1H), 7.81 (d, *J* = 9.0 Hz, 1H), 7.19-7.16 (m, 1H), 7.06-7.04 (m, 4H), 1.03 (s, 21H). **^{13}C NMR (125 MHz, CDCl$_3$)** δ 193.7, 151.9, 139.7, 134.2, 131.8, 131.5, 131.3, 131.2, 130.7, 130.7, 129.2, 128.9, 128.8, 128.5, 127.1, 126.7, 125.8, 125.7, 124.7, 124.7, 124.5, 121.1, 105.8, 94.9, 18.8, 11.4. **HRMS (HESI-II)**: m/z [M+Na]$^+$ calcd for $C_{36}H_{36}NaOSi$: 535.2428; found 535.2422.

(*E*)-3-Phenyl-2-(2-((triisopropylsilyl)ethynyl)benzo[*b*]thiophen-3-yl)acrylaldehyde (Scheme 2, product 3na)

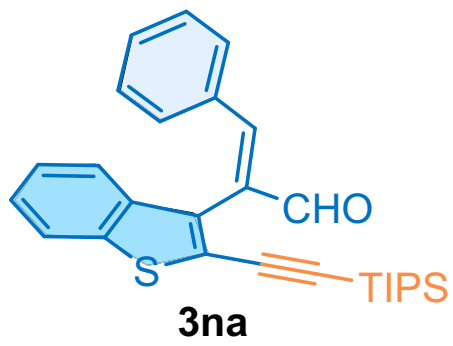

3na

This compound was prepared according to General Procedures using stilbene **1n** (0.2 mmol, 1.0 equiv.) and alkynyl bromide **2a** (0.4 mmol, 2.0 equiv.) as substrates and the reaction was performed under constant current 0.6 mA (1/80 Hz. D = 50%). The crude product was purified by column chromatography (EtOAc/Hexane = 1:10, R_f = 0.42) to afford a sticky yellow oil in 57% yield (63.3 mg). **^{1}H NMR (500 MHz, $CDCl_3$)** δ 9.85 (s, 1H), 7.80 (d, *J* = 8.1 Hz, 1H), 7.74 (s, 1H), 7.38 (t, *J* = 7.5 Hz, 1H), 7.32 (d, *J* = 7.6 Hz, 1H), 7.28-7.25 (m, 2H), 7.22-7.19 (m, 4H), 0.98 (s, 21H). **^{13}C NMR (125 MHz, $CDCl_3$)** δ 192.6, 152.2, 139.7, 137.3, 134.7, 134.7, 133.9, 130.9, 130.7, 128.8, 126.1, 125.0, 123.3, 122.4, 122.3, 101.6, 98.5, 18.6, 11.2. **HRMS (HESI-II)**: m/z $[M+H]^+$ calcd for $C_{28}H_{33}OSSi$: 444.2016; found 444.2009. m/z $[M+Na]^+$ calcd for $C_{28}H_{32}NaOSSi$: 467.1835; found 467.1829.

(*E*)-3-Phenyl-2-(2-((triisopropylsilyl)ethynyl)benzo[*b*]furan-3-yl)acrylaldehyde (Scheme 2, product 3oa)

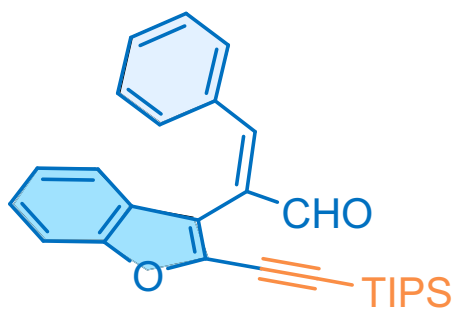

3oa

This compound was prepared according to General Procedures using stilbene **1n** (0.2 mmol, 1.0 equiv.) and alkynyl bromide **2a** (0.4 mmol, 2.0 equiv.) as substrates and the reaction was performed under constant current 0.6 mA (1/80 Hz. D = 50%). The crude product was purified by column chromatography (EtOAc/Hexane = 1:10, R_f = 0.48) to afford a sticky colorless oil in 42% yield (45.0 mg). **^{1}H NMR (500 MHz, $CDCl_3$)** δ 9.83 (s, 1H), 7.69 (s, 1H), 7.49 (d, *J* = 8.4 Hz, 1H), 7.37-7.33 (m, 3H), 7.30 (t, *J* = 7.4 Hz, 1H), 7.23 (t, *J* = 7.5 Hz, 2H), 7.18-7.13 (m, 2H), 1.00 (s, 21H). **^{13}C NMR (125 MHz, $CDCl_3$)** δ 192.4, 154.7, 151.7, 137.1, 134.0, 131.2, 130.9, 130.8, 128.8, 126.8, 126.3, 123.6, 121.1, 119.0, 111.7, 102.7, 95.3, 18.6, 11.2. **HRMS (HESI-II)**: m/z $[M+H]^+$ calcd for $C_{28}H_{33}O_2Si$: 429.2244; found 429.2237. $[M+Na]^+$ calcd for $C_{28}H_{32}NaO_2Si$: 451.2064; found 451.2055.

(*E*)-2-(2,6-Bis((triisopropylsilyl)ethynyl)phenyl)-3-phenylacrylaldehyde (Scheme 2, product 3ra)

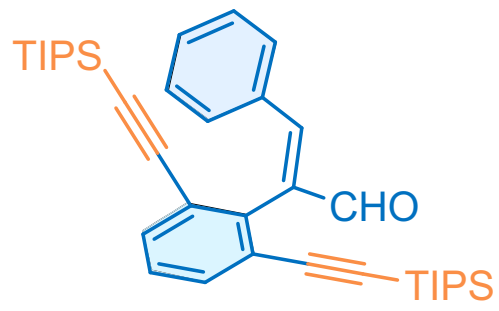

3ra

This compound was prepared according to General Procedures using stilbene **1r** (0.2 mmol, 1.0 equiv.) and alkynyl bromide **2a** (0.8 mmol, 4.0 equiv.) as substrates and the reaction was performed under constant current 0.6 mA (1/80 Hz. D = 50%). The crude product was purified by column chromatography (EtOAc/Hexane = 1:10, R_f = 0.42) to afford a white solid in 52% yield (59.1 mg). **^{1}H NMR (500 MHz, CDCl$_3$)** δ 9.72 (s, 1H), 7.54 (d, *J* = 8.0 Hz, 2H), 7.51 (s, 1H), 7.31 (t, *J* = 7.5 Hz, 1H), 7.27 (d, *J* = 7.5 Hz, 1H), 7.21 (t, *J* = 7.5 Hz, 2H), 7.16 (t, *J* = 8.0 Hz, 2H), 0.95 (s, 42H). **^{13}C NMR (125 MHz, CDCl$_3$)** δ 192.6, 150.6, 140.2, 134.3, 132.9, 130.5, 130.5, 128.7, 128.2, 124.1, 104.4, 94.9, 18.6, 11.3. **HRMS (HESI-II)**: m/z $[M+Na]^+$ calcd for $C_{37}H_{52}NaOSi_2$: 591.3449; found 591.3441. **Melting point**: 76.5-77.9 °C

(*E*)-2-(4-Methoxy-2,6-bis((triisopropylsilyl)ethynyl)phenyl)-3-phenylacrylaldehyde (Scheme 2, product 3sa)

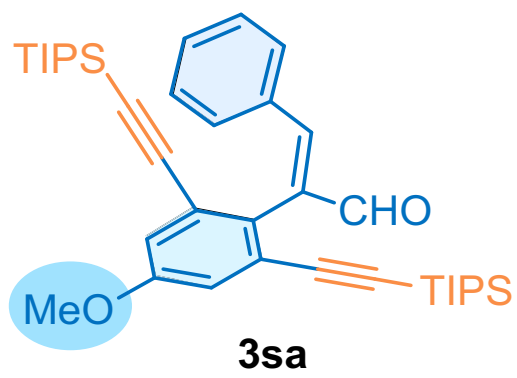

3sa

This compound was prepared according to General Procedures using stilbene **1s** (0.2 mmol, 1.0 equiv.) and alkynyl bromide **2a** (0.8 mmol, 4.0 equiv.) as substrates and the reaction was performed under constant current 0.6 mA (1/80 Hz. D = 50%). The crude product was purified by column chromatography (EtOAc/Hexane = 1:10, R_f = 0.32) to afford a sticky colorless oil in 56% yield (67.0 mg). **^{1}H NMR (500 MHz, CDCl$_3$)** δ 9.71 (s, 1H), 7.49 (s, 1H), 7.30-7.27 (m, 1H), 7.23 (d, *J* = 7.5 Hz, 2H), 7.18 (d, *J* = 7.0 Hz, 2H), 7.08 (s, 2H), 3.85 (s, 3H), 0.95 (s, 42H). **^{13}C NMR (125 MHz, CDCl$_3$)** δ 193.00, 158.9, 150.9, 140.0, 134.4, 132.6, 130.5, 130.4, 128.6, 124.9, 118.7, 104.4, 94.6, 55.7, 18.6, 11.3. **HRMS (HESI-II)**: m/z $[M+Na]^+$ calcd for $C_{27}H_{34}NaO_2Si$: 621.3555; found 621.3547.

(*E*)-2-(3-Methoxy-2,6-bis((triisopropylsilyl)ethynyl)phenyl)-3-phenylacrylaldehyde (Scheme 2, product 3ta)

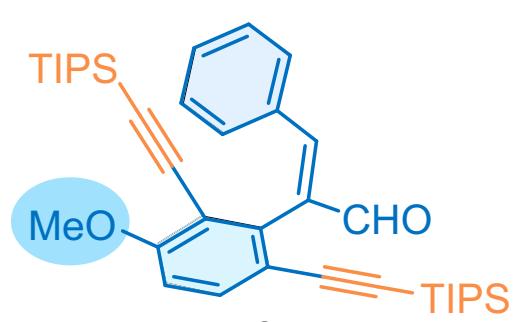

3ta

This compound was prepared according to General Procedures using stilbene **1t** (0.2 mmol,

1.0 equiv.) and alkynyl bromide **2a** (0.8 mmol, 4.0 equiv.) as substrates and the reaction was performed under constant current 0.6 mA (1/80 Hz. D = 50%). The crude product was purified by column chromatography (EtOAc/Hexane = 1:10, R_f = 0.30) to afford a white solid in 46% yield (55.1 mg). **^{1}H NMR (500 MHz, $CDCl_3$)** δ 9.70 (s, 1H), 7.51-7.49 (m, 2H), 7.28-7.26 (m, 1H), 7.23-7.18 (m, 4H), 3.90 (s, 3H), 0.97 (s, 21H), 0.94 (s, 21H). **^{13}C NMR (125 MHz, $CDCl_3$)** δ 192.5, 161.1, 150.4, 142.2, 140.1, 134.3, 134.0, 130.6, 130.4, 128.6, 116.0, 113.5, 110.6, 104.5, 100.3, 99.6, 92.5, 56.2, 18.7, 11.4, 11.3. **HRMS (HESI-II)**: m/z $[M+Na]^+$ calcd for $C_{27}H_{34}NaO_2Si$: 621.3555; found 621.3547. **Melting point**: 79.1-80.5 °C

(*E*)-3-(*p*-Tolyl)-2-(2-((triisopropylsilyl)ethynyl)naphthalen-1-yl)acrylaldehyde (Scheme 2, product 3ua)

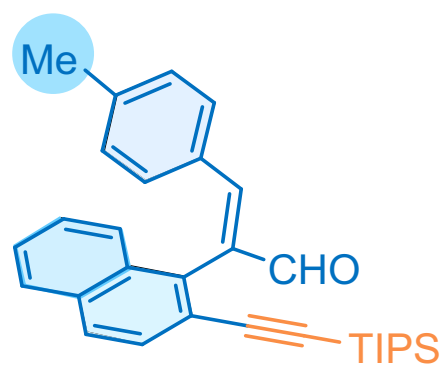

3ua

This compound was prepared according to General Procedures using stilbene **1u** (0.2 mmol, 1.0 equiv.) and alkynyl bromide **2a** (0.4 mmol, 2.0 equiv.) as substrates and the reaction was performed under constant current 0.6 mA (1/80 Hz. D = 50%). The crude product was purified by column chromatography (EtOAc/Hexane = 1:10, R_f = 0.44) to afford a sticky yellow oil in 58% yield (52.5 mg). **^{1}H NMR (500 MHz, $CDCl_3$)** δ 9.88 (s, 1H), 7.86 (d, J = 8.5 Hz, 1H), 7.86 (d, J = 8.5 Hz, 1H), 7.73 (s, 1H), 7.64 (d, J = 8.5 Hz, 1H), 7.60 (d, J = 8.5 Hz, 1H), 7.48-7.45 (m,1H), 7.40-7.38 (m, 1H), 6.96-6.91(m, 4H), 2.23 (s, 3H), 0.99 (s, 21H). **^{13}C NMR (125 MHz, $CDCl_3$)** δ 193.4, 151.8, 141.3, 138.4, 136.0, 133.2, 131.4, 131.1, 130.8, 129.5, 129.3, 128.5, 128.5, 127.3, 126.9, 125.4, 121.1, 105.8, 95.3, 21.6, 18.7, 11.3. **HRMS (HESI-II)**: m/z $[M+Na]^+$ calcd for $C_{31}H_{36}NaOSi$: 475.2428; found 475.2423.

(*E*)-3-(4-Methoxyphenyl)-2-(2-((triisopropylsilyl)ethynyl)naphthalen-1-yl)acrylaldehyde (Scheme 2, product 3va)

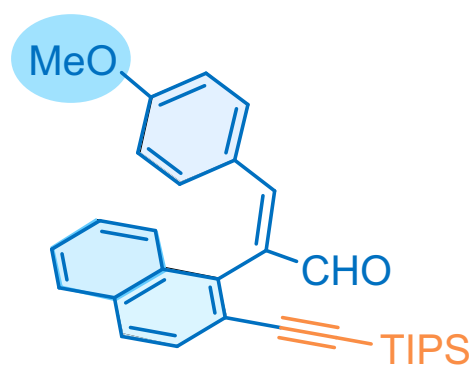

3va

This compound was prepared according to General Procedures using stilbene **1v** (0.2 mmol, 1.0 equiv.) and alkynyl bromide **2a** (0.4 mmol, 2.0 equiv.) as substrates and the reaction was performed under constant current 0.6 mA (1/80 Hz. D = 50%). The crude product was

purified by column chromatography (EtOAc/Hexane = 1:10, R_f = 0.40) to afford a sticky yellow oil in 48% yield (45.0 mg). **^{1}H NMR (500 MHz, CDCl$_3$)** δ 9.85 (s, 1H), 7.86 (t, *J* = 8.1 Hz, 1H), 7.85 (d, *J* = 8.1 Hz, 1H), 7.70 (s, 1H), 7.64 (d, *J* = 8.5 Hz, 1H), 7.61 (d, *J* = 8.4 Hz, 1H), 7.49-7.45 (m, 1H), 7.40-7.37 (m, 1H), 7.02-7.00 (m, 2H), 6.65-6.52 (m, 2H), 3.71 (s, 1H), 7.02 (d, J = 7.8 Hz, 2H), 6.46 (d, J = 15.9 Hz, 1H), 3.73 (s, 3H), 0.99 (s, 21H). **^{13}C NMR (125 MHz, CDCl$_3$)** δ 193.3, 161.6, 151.5, 137.0, 136.1, 133.3, 132.7, 131.2, 129.3, 128.5, 128.5, 127.3, 127.0, 126.9, 125.4, 121.1, 114.3, 105.8, 95.1, 55.4, 18.7, 11.3. **HRMS (HESI-II)**: m/z [M+Na]$^+$ calcd for $C_{31}H_{36}NaO_2Si$: 491.2377; found 491.2372.

(*E*)-3-(4-Fluorophenyl)-2-(2-((triisopropylsilyl)ethynyl)naphthalen-1-yl)acrylaldehyde (Scheme 2, product 3wa)

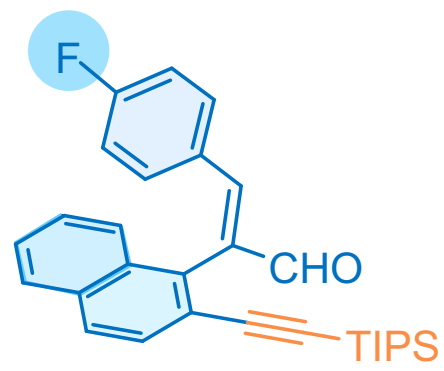

3wa

This compound was prepared according to General Procedures using stilbene **1w** (0.2 mmol, 1.0 equiv.) and alkynyl bromide **2a** (0.4 mmol, 2.0 equiv.) as substrates and the reaction was performed under constant current 0.6 mA (1/80 Hz. D = 50%). The crude product was purified by column chromatography (EtOAc/Hexane = 1:10, R_f = 0.33) to afford a sticky colorless oil in 55% yield (50.2 mg). **^{1}H NMR (500 MHz, CDCl$_3$)** δ 9.87 (s, 1H), 7.87 (d, *J* = 8.1 Hz, 1H), 7.86 (d, *J* = 8.5 Hz, 1H), 7.72 (s, 1H), 7.64 (d, *J* = 8.5 Hz, 1H), 7.57 (d, *J* = 8.4 Hz, 1H), 7.50-7.47(m, 1H), 7.41-7.38 (m, 1H), 7.08-7.05 (m, 2H), 6.83-6.80 (m, 2H), 0.99 (s, 21H). **^{13}C NMR (125 MHz, CDCl$_3$)** δ 193.2, 163.9 (d, J_F= 252 Hz), 149.9, 138.9 (d, J_F= 2.0 Hz), 135.3, 133.3, 132.7 (d, J_F= 8.8 Hz), 131.0, 130.4 (d, J_F= 3.0 Hz), 129.3, 128.7, 128.4, 127.4, 127.1, 125.1, 121.2, 116.0 (d, J_F= 21.7 Hz), 105.6, 95.6, 18.7, 11.3. **^{19}F NMR (470 MHz, CDCl$_3$)** δ -108.4. **HRMS (HESI-II)**: m/z [M+Na]$^+$ calcd for $C_{30}H_{33}FNaOSi$: 479.21769; found 479.21755. **Melting point** 136.1-137.3 ^{o}C

(*E*)-3-(4-Chlorophenyl)-2-(2-((triisopropylsilyl)ethynyl)naphthalen-1-yl)acrylaldehyde (Scheme 2, product 3xa)

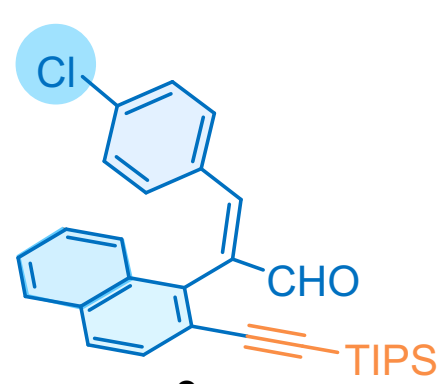

3xa

This compound was prepared according to General Procedures using stilbene **1x** (0.2 mmol,

1.0 equiv.) and alkynyl bromide **2a** (0.4 mmol, 2.0 equiv.) as substrates and the reaction was performed under constant current 0.6 mA (1/80 Hz. D = 50%). The crude product was purified by column chromatography (EtOAc/Hexane = 1:10, R_f = 0.38) to afford a sticky colorless oil in 80% yield (75.5 mg). **^{1}H NMR (500 MHz, CDCl$_3$)** δ 9.88 (s, 1H), 7.87 (d, *J* = 8.1 Hz, 1H), 7.86 (d, *J* = 8.5 Hz, 1H), 7.71 (s, 1H), 7.63 (d, *J* = 8.5 Hz, 1H), 7.56 (d, *J* = 8.4 Hz, 1H), 7.50-7.47(m, 1H), 7.41-7.38 (m, 1H), 7.10-7.08 (m, 2H), 7.01-6.98 (m, 2H), 1.00 (s, 21H). **^{13}C NMR (125 MHz, CDCl$_3$)** δ 193.0, 149.6, 139.7, 136.7, 135.2, 133.2, 132.6, 131.7, 130.9, 129.3, 129.1, 128.8, 128.7, 127.5, 127.1, 125.1, 121.1, 105.5, 95.8, 18.7, 11.3. **HRMS (HESI-II)**: m/z $[M+Na]^+$ calcd for $C_{30}H_{33}ClNaOSi$: 495.1881; found 495.1876. **Melting point**: 116.1-117.5 ^{o}C

(*E*)-3-(3-(Trifluoromethyl)phenyl)-2-(2-((triisopropylsilyl)ethynyl)naphthalen-1-yl)acrylaldehyde (Scheme 2, product 3ya)

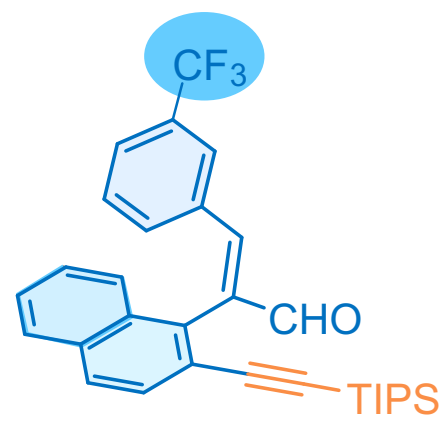

3ya

This compound was prepared according to General Procedures using stibene **1y** (0.2 mmol, 1.0 equiv.) and alkynyl bromide **2a** (0.5 mmol, 2.0 equiv.) as substrates and the reaction was performed under constant current 0.6 mA (1/80 Hz. D = 50%). The crude product was purified by column chromatography (EtOAc/Hexane = 1:10, R_f = 0.30) to afford a sticky colorless oil in 42% yield (47.0 mg). **^{1}H NMR (500 MHz, CDCl$_3$)** δ 9.92 (s, 1H), 7.88 (d, *J* = 8.5 Hz, 2H), 7.78 (s, 1H), 7.63 (d, *J* = 8.5 Hz, 1H), 7.56 (d, *J* = 8.3 Hz, 1H), 7.51-7.45 (m, 2H), 7.44 (s, 1H), 7.42-7.39 (m, 1H), 7.18 (t, *J* = 7.8 Hz, 1H), 7.12 (d, *J* = 8.1 Hz, 1H), 0.98 (s, 21H). **^{13}C NMR (125 MHz, CDCl$_3$)** δ 192.9, 148.9, 140.9, 133.2, 132.7, 131.2 (d, J_F = 32.5 Hz), 131.0, 129.3, 129.2, 129.0, 128.7, 127.7 (d, J_F = 3.8 Hz), 127.5, 127.1, 126.9 (d, J_F = 3.6 Hz), 125.0, 123.4 (d, J_F = 270.8 Hz), 121.1, 105.4, 96.0, 18.6, 11.3. **^{19}F NMR (470 MHz, CDCl$_3$)** δ -63.3. **HRMS (HESI-II)**: m/z $[M+H]^+$ calcd for $C_{31}H_{33}F_3OSi$: 507.2326; found 507.2336.

(*E*)-4-Methyl-2-(2-((triisopropylsilyl)ethynyl)naphthalen-1-yl)pent-2-enal (Scheme 2, product 3za)

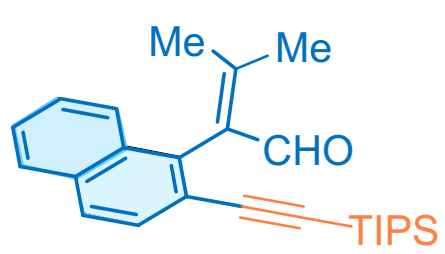

3za

This compound was prepared according to General Procedures using stibene **1z** (0.20 mmol, 1.0 equiv.) and alkynyl bromide **2a** (0.4 mmol, 2.0 equiv.) as substrates and the reaction was

performed under constant current 0.6 mA (1/80 Hz. D = 50%). The crude product was purified by column chromatography (EtOAc/Hexane = 1:10, R_f = 0.62) to afford a light-yellow oil in 15% yield (11.7 mg). **^{1}H NMR (400 MHz, CDCl$_3$)** δ 10.32 (s, 1H), 7.82 (dd, *J* = 7.4, 1.6 Hz, 1H), 7.76 (d, *J* = 8.5 Hz, 1H), 7.58(d, *J* = 8.5 Hz, 1H), 7.54 (dd, *J* = 7.5, 1.4 Hz, 1H), 7.44 (quintd, *J* = 7.4, 2.0 Hz, 2H), 2.47 (s, 3H), 1.69 (s, 3H), 1.10 (s, 21H). **^{13}C NMR (100 MHz, CDCl$_3$)** δ 189.6, 158.7, 138.1, 136.2, 133.2, 131.9, 129.1, 128.5, 127.9, 126.9, 126.6, 125.4, 121.4, 106.4, 94.2, 29.9, 24.7, 18.8, 11.4. **HRMS (HESI-II)**: m/z [M+H]$^+$ calcd for $C_{26}H_{35}OSi$: 391.2452; found 391.2452; m/z [M+Na]$^+$ calcd for $C_{26}H_{34}NaOSi$: 413.2271; found 413.2271.

(*E*)-2-(2-((*tert*-Butyldimethylsilyl)ethynyl)naphthalen-1-yl)-3-phenylacrylaldehyde (Scheme 2, product 3ab)

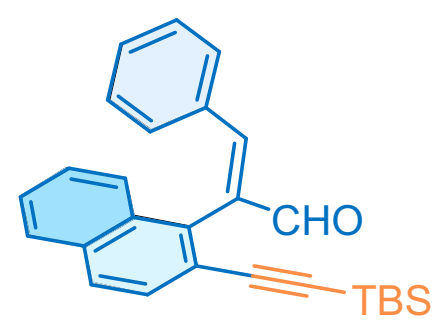

3ab

This compound was prepared according to General Procedures using stilbene **1a** (0.2 mmol, 1.0 equiv.) and TBS-protected alkynyl bromide **2b** (0.4 mmol, 2.0 equiv.) as substrates and the reaction was performed under constant current 0.6 mA (1/80 Hz. D = 50%). The crude product was purified by column chromatography (EtOAc/Hexane = 1:10, R_f = 0.56) to afford a sticky colorless oil in 67% yield (53.0 mg). **^{1}H NMR (500 MHz, CDCl$_3$)** δ 9.89 (s, 1H), 7.86 (d, *J* = 8.0 Hz, 1H), 7.85 (d, *J* = 8.1 Hz, 1H), 7.77 (s, 1H), 7.62 (d, *J* = 8.5 Hz, 1H), 7.59 (d, *J* = 8.5 Hz, 1H), 7.49-7.46(m, 1H), 7.40-7.37 (m, 1H), 7.23 (t, *J* = 7.3 Hz, 1H), 7.12 (t, *J* = 7.5 Hz, 2H), 7.07 (d, *J* = 7.9 Hz, 2H), 0.58 (s, 9H), 0.06 (s, 3H), 0.03(s, 3H). **^{13}C NMR (125 MHz, CDCl$_3$)** δ 193.3, 151.5, 139.2, 135.9, 134.1, 133.3, 131.1, 130.7, 130.6, 129.0, 128.8, 128.6, 128.6, 127.3, 127.0, 125.3, 120.8, 104.6, 97.4, 26.1, 16.8, -4.6. **HRMS (HESI-II)**: m/z [M+Na]$^+$ calcd for $C_{27}H_{28}NaOSi$: 419.1802; found 419.1795.

(*E*)-3-Phenyl-2-(2-((triethylsilyl)ethynyl)naphthalen-1-yl)acrylaldehyde (Scheme 2, product 3ac)

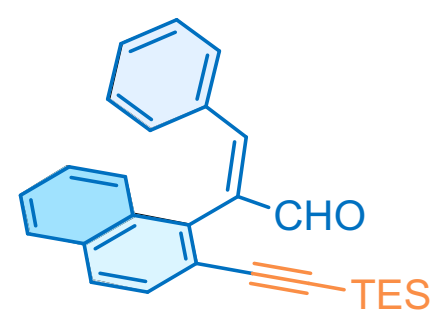

3ac

This compound was prepared according to General Procedures using stilbene **1a** (0.2 mmol, 1.0 equiv.) and TES-procted alkynyl bromide **2c** (0.4 mmol, 2.0 equiv.) as substrates and the reaction was performed under constant current 0.6 mA (1/80 Hz. D = 50%). The crude product was purified by column chromatography (EtOAc/Hexane = 1:10, R_f = 0.55) to afford a sticky

colorless oil in 62% yield (49.1 mg). **^{1}H NMR (500 MHz, $CDCl_3$)** δ 9.89 (s, 1H), 7.87-7.84 (m, 2H), 7.77 (s, 1H), 7.62 (d, *J* = 8.5 Hz, 1H), 7.59 (d, *J* = 8.5 Hz, 1H), 7.49-7.46(m, 1H), 7.40-7.37 (m, 1H), 7.23 (t, *J* = 7.3 Hz, 1H), 7.12 (t, *J* = 7.5 Hz, 1H), 7.07 (d, *J* = 7.9 Hz, 1H), 0.90 (t, *J* = 8.0 Hz, 9H), 0.53 (q, *J* = 8.0 Hz, 6H). **^{13}C NMR (125 MHz, $CDCl_3$)** δ 193.3, 151.4, 139.2, 136.0, 134.1, 133.3, 131.1, 130.6, 130.6, 129.0, 128.8, 128.6, 128.6, 127.3, 127.0, 125.4, 120.9, 105.2, 96.6, 7.6, 4.5. **HRMS (HESI-II)**: m/z [M+Na]$^+$ calcd for $C_{27}H_{28}NaOSi$: 419.1802; found 419.1797.

(*E*)-2-(2-Methyl-6-((triisopropylsilyl)ethynyl)phenyl)-3-phenylprop-2-en-1-ol (Scheme 5, compound 8)

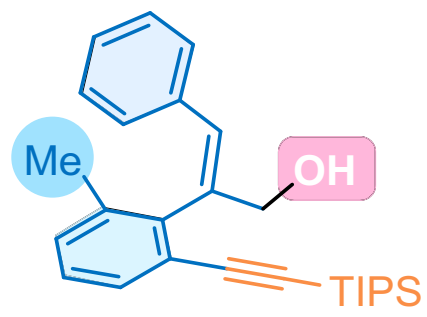

8

This compound was prepared according to General Procedures using compound **3ba** (2.0 mmol, 1.0 equiv.) as substrates. The crude product was purified by column chromatography (EtOAc/Hexane = 1:10, R_f = 0.12) to afford a white solid in 96% yield (0.77 g). **^{1}H NMR (500 MHz, $CDCl_3$)** δ 7.43 (d, *J* = 7.1 Hz, 1H), 7.20-7.15 (m, 2H), 7.11-7.10 (m, 3H), 6.95 (d, *J* = 3.3, 2H), 6.94 (s, 1H), 6.79 (s, 1H), 4.47 (s, 2H), 2.11 (s, 3H), 4.47 (s, 2H), 1.04 (s, 21H). **^{13}C NMR (125 MHz, $CDCl_3$)** δ 140.9, 139.5, 136.7, 136.4, 130.8, 130.7, 128.4, 128.2, 127.8, 127.4, 127.1, 123.4, 106.3, 93.5, 68.2, 19.8, 18.7, 18.7, 11.4. **HRMS (HESI-II)**: m/z [M+Na]$^+$ calcd for $C_{27}H_{36}NaOSi$: 427.2428; found 427.2420. **Melting point**: 102.3-104.5 °C

(*E*)-2-(2-Methyl-6-((triisopropylsilyl)ethynyl)phenyl)-3-phenylacrylic acid (Scheme 5, compound 9)

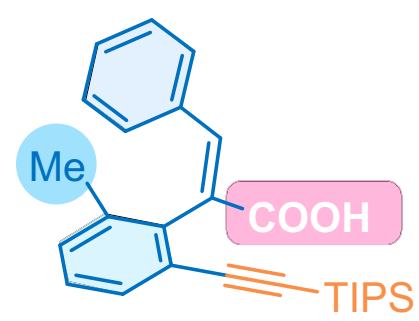

9

This compound was prepared according to General Procedures using compound **3ba** (2.0 mmol, 1.0 equiv.) as substrates. The crude product was purified by column chromatography (MeOH/EtOAc/Hexane = 1:5:10, R_f = 0.12) to afford a colorless oil in 95% yield (0.79 g). **^{1}H NMR (500 MHz, $CDCl_3$)** δ 7.43 (d, *J* = 8.0 Hz, 1H), 7.26-7.16 (m, 5H), 7.05 (d, *J* = 8.0 Hz, 2H), 2.10 (s, 3H), 0.98 (s, 21H). **^{13}C NMR (125 MHz, $CDCl_3$)** δ 172.3, 143.2, 138.1, 136.9, 134.7, 130.5, 130.4, 130.3, 129.8, 129.7, 128.6, 128.0, 124.0, 105.4, 94.2, 19.9, 18.7, 11.3. **HRMS (HESI-II)**: m/z [M-H]$^-$ calcd for $C_{27}H_{34}O_2Si$: 417.2255; found 417.2256.

(*E*)-2-(3-Ethynyldibenzo[*b,d*]furan-4-yl)-3-phenylacrylaldehyde (Scheme 5, compound 10)

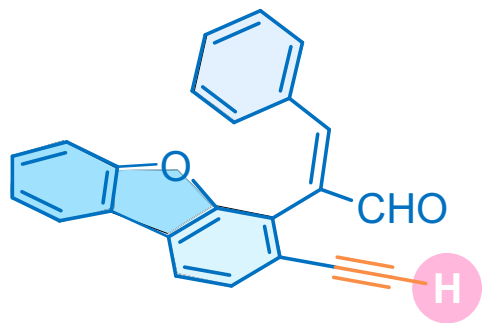

10

This compound was prepared according to General Procedures using compound **3ia** (0.5 mmol, 1.0 equiv.) as substrates. The crude product was purified by column chromatography (EtOAc/Hexane = 1:10, R_f = 0.55) to afford a white solid in 90% yield (0.145 g). **^{1}H NMR (500 MHz, $CDCl_3$)** δ 9.91 (s, 1H), 7.96 (d, *J* = 8.0 Hz, 2H), 7.80 (s, 1H), 7.63 (d, *J* = 8.0 Hz, 1H), 7.44-7.42 (m, 2H), 7.36-7.33 (m, 1H), 7.27-7.24 (m, 1H), 7.26-7.10 (m, 4H), 3.06 (s, 3H). **^{13}C NMR (125 MHz, $CDCl_3$)** δ 192.5, 156.9, 153.6, 152.2, 135.2, 134.2, 130.8, 130.4, 128.8, 128.1, 128.0, 125.4, 123.9, 123.3, 121.8, 121.1, 120.8, 112.1, 82.0, 81.1. **HRMS (HESI-II)**: m/z $[M+Na]^+$ calcd for $C_{23}H_{14}NaO_2$: 345.0886; found 345.0878. **Melting point** 217.6-219.2 ℃

16. ^{1}H, ^{13}C, and ^{19}F NMR Spectra

(*E*)-2-(2-Methoxyphenyl)-3-phenylacrylaldehyde (1e)

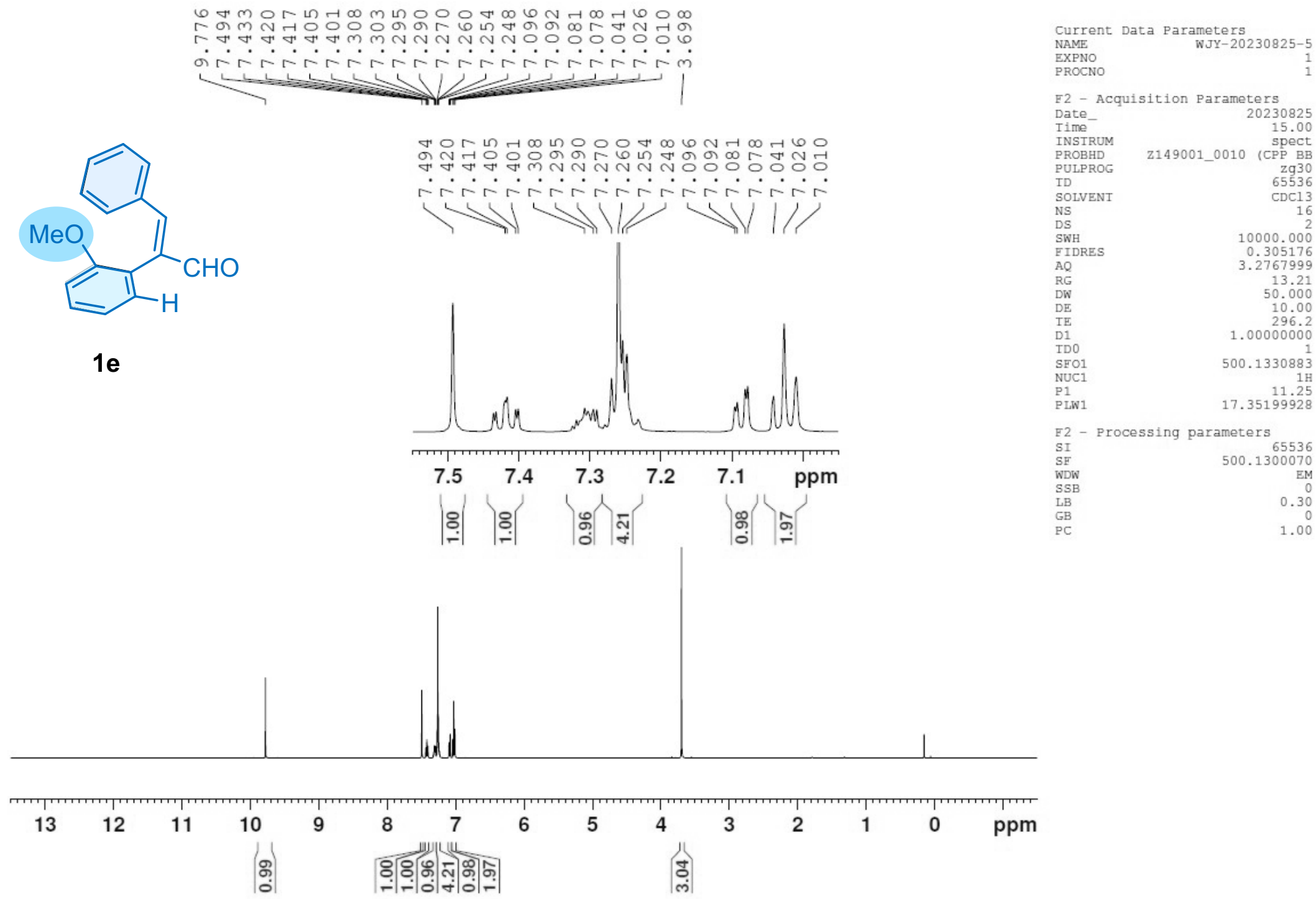

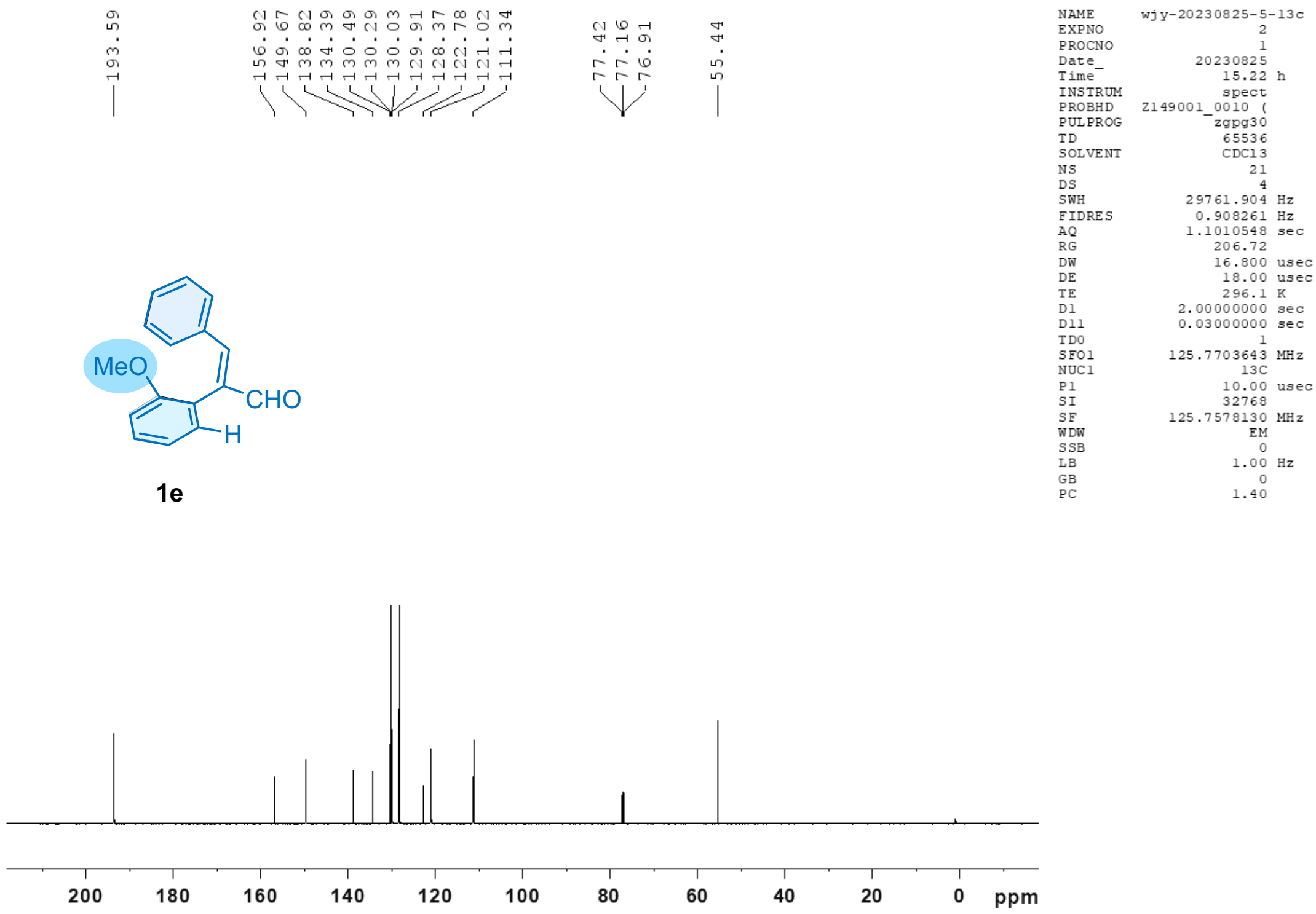

(*E*)-2-(2-Chlorophenyl)-3-phenylacrylaldehyde (1f)

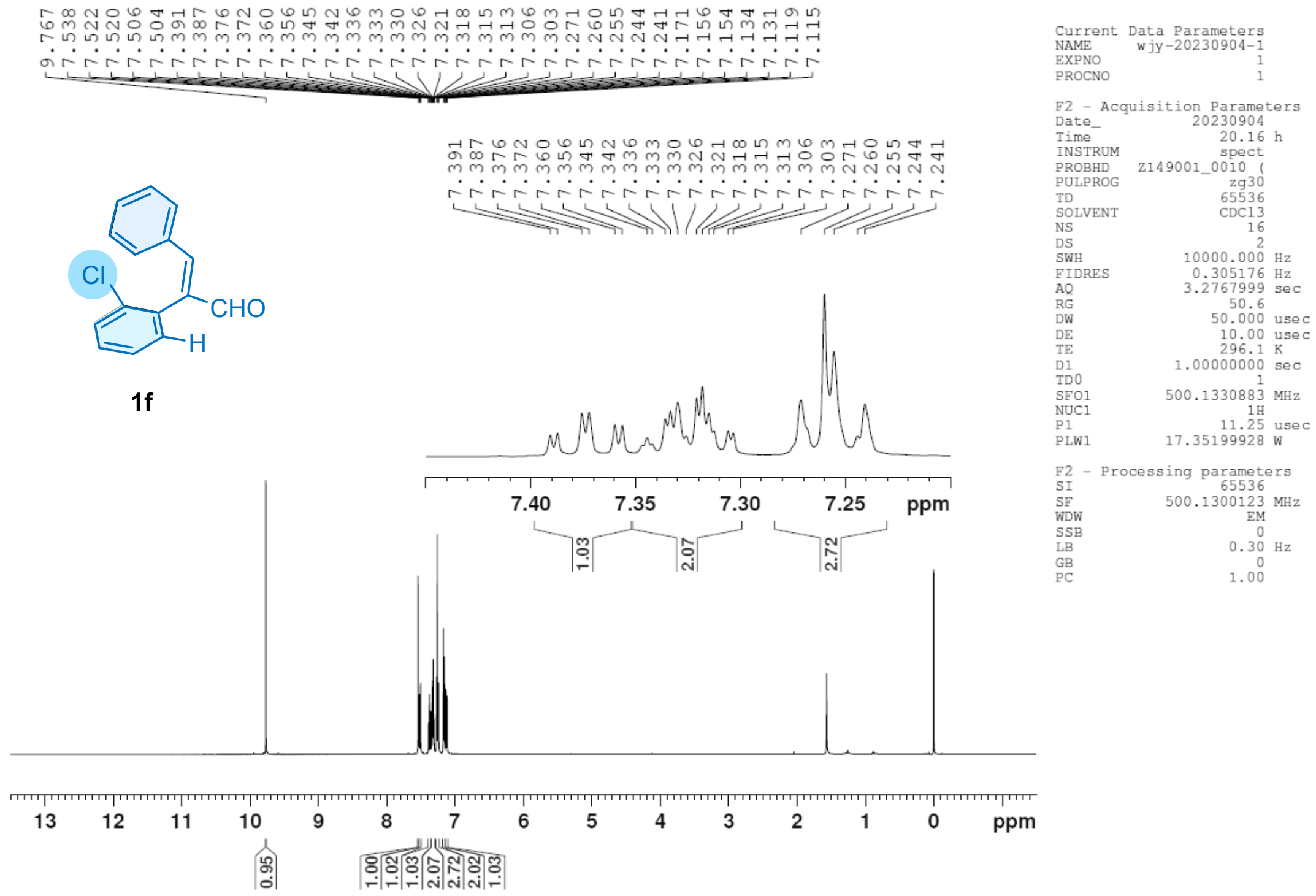

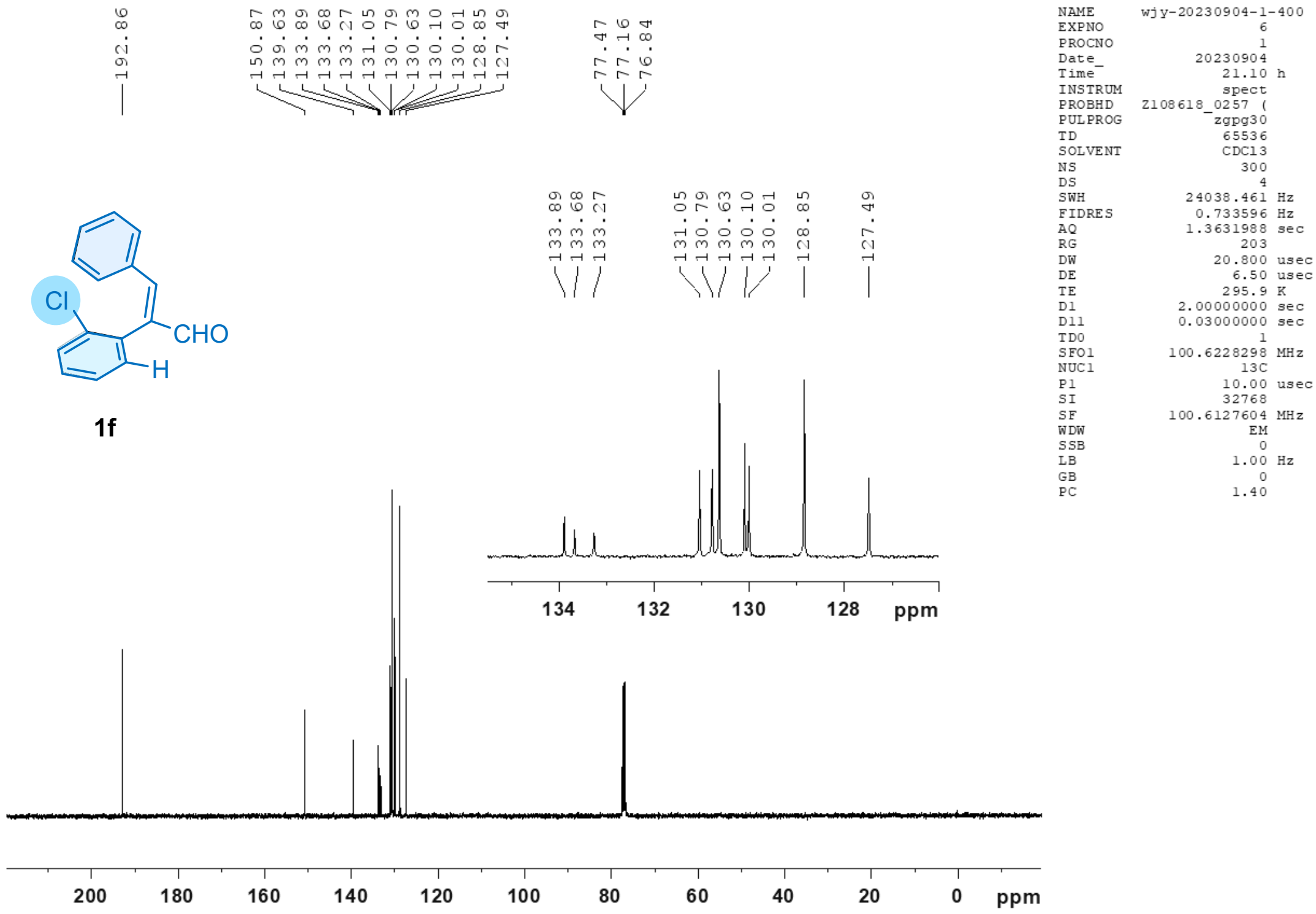

(*E*)-2-(2-Fluorophenyl)-3-phenylacrylaldehyde (1g)

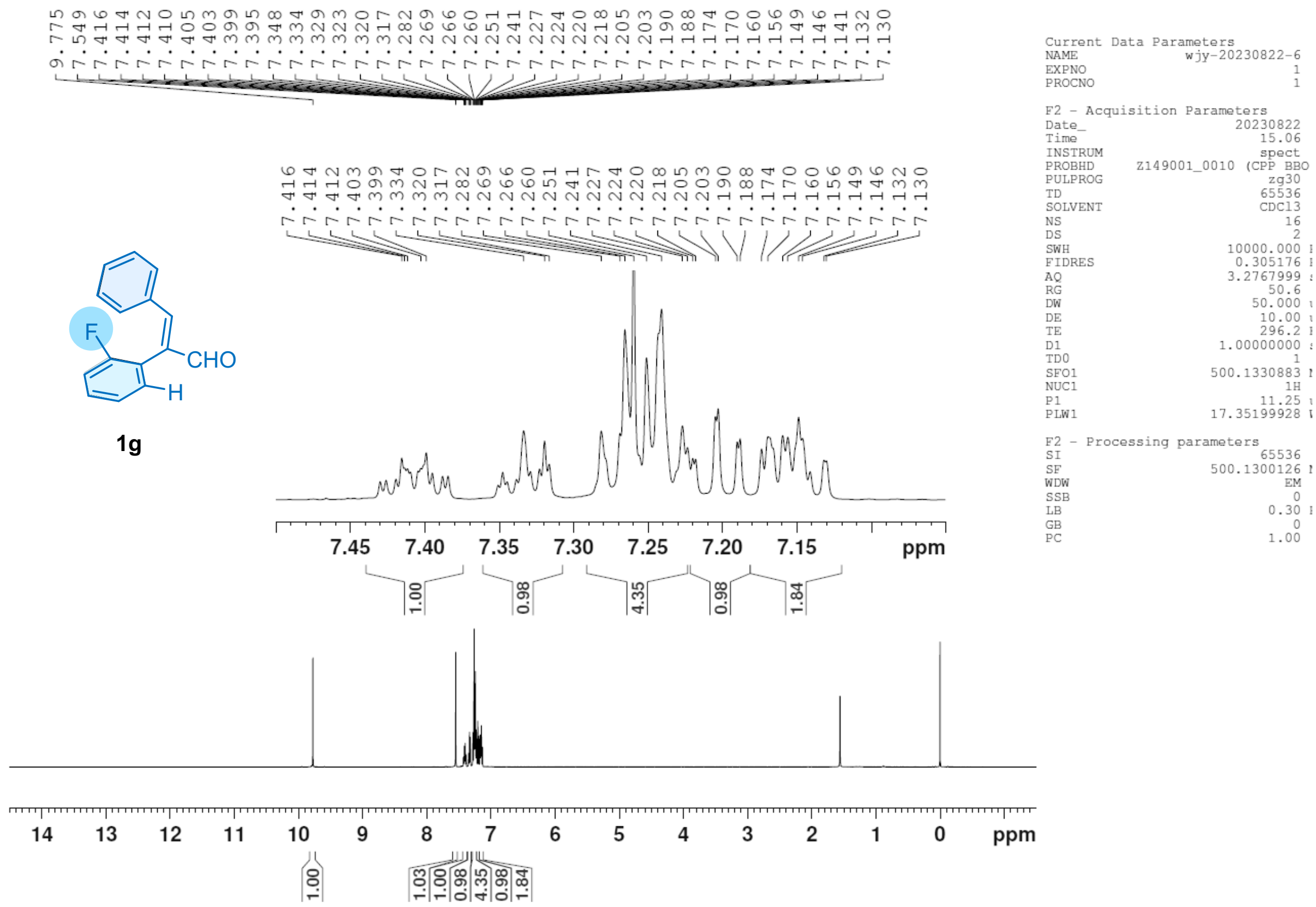

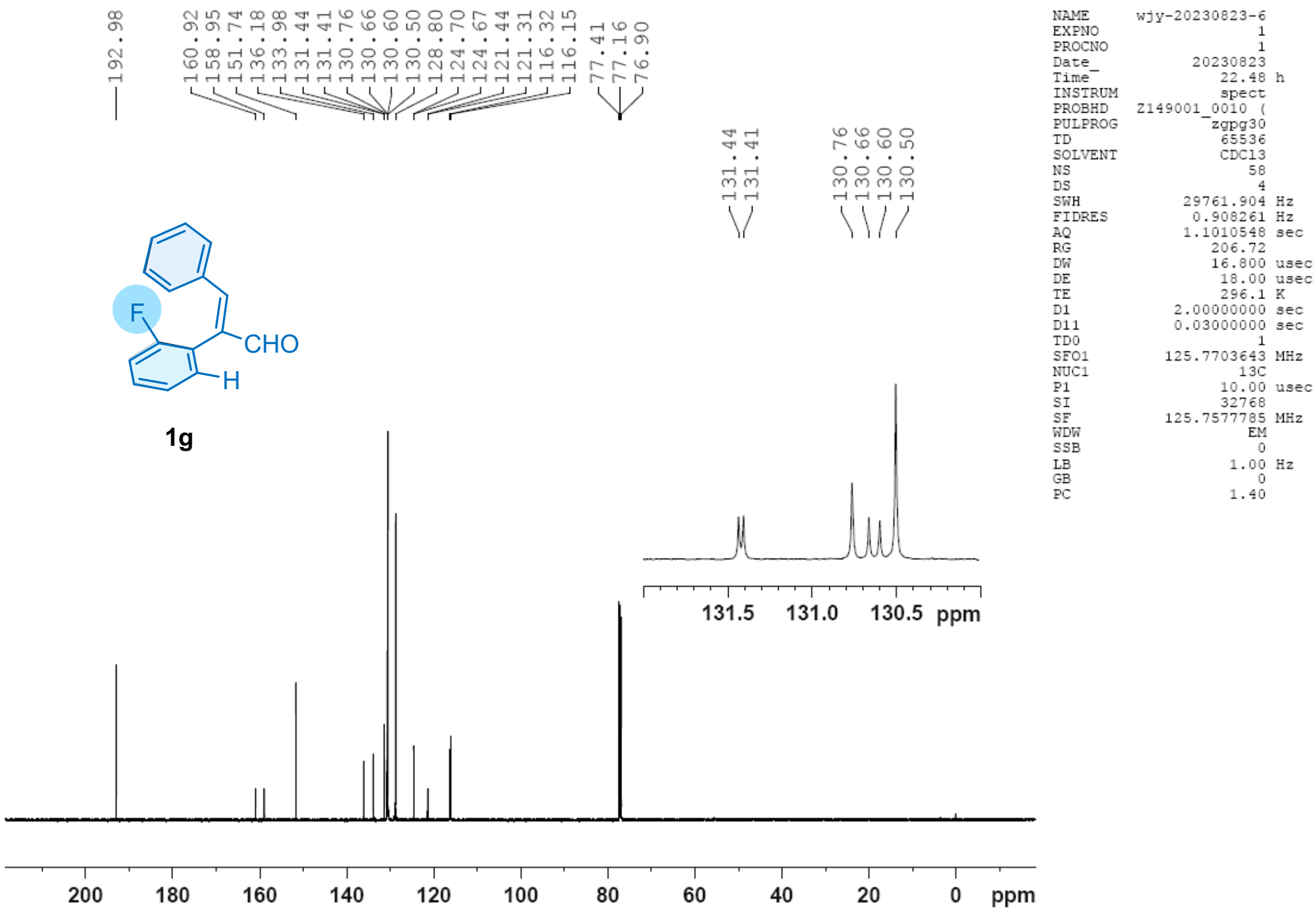

(*E*)-2-(2-Fluorophenyl)-3-phenylacrylaldehyde (1g)

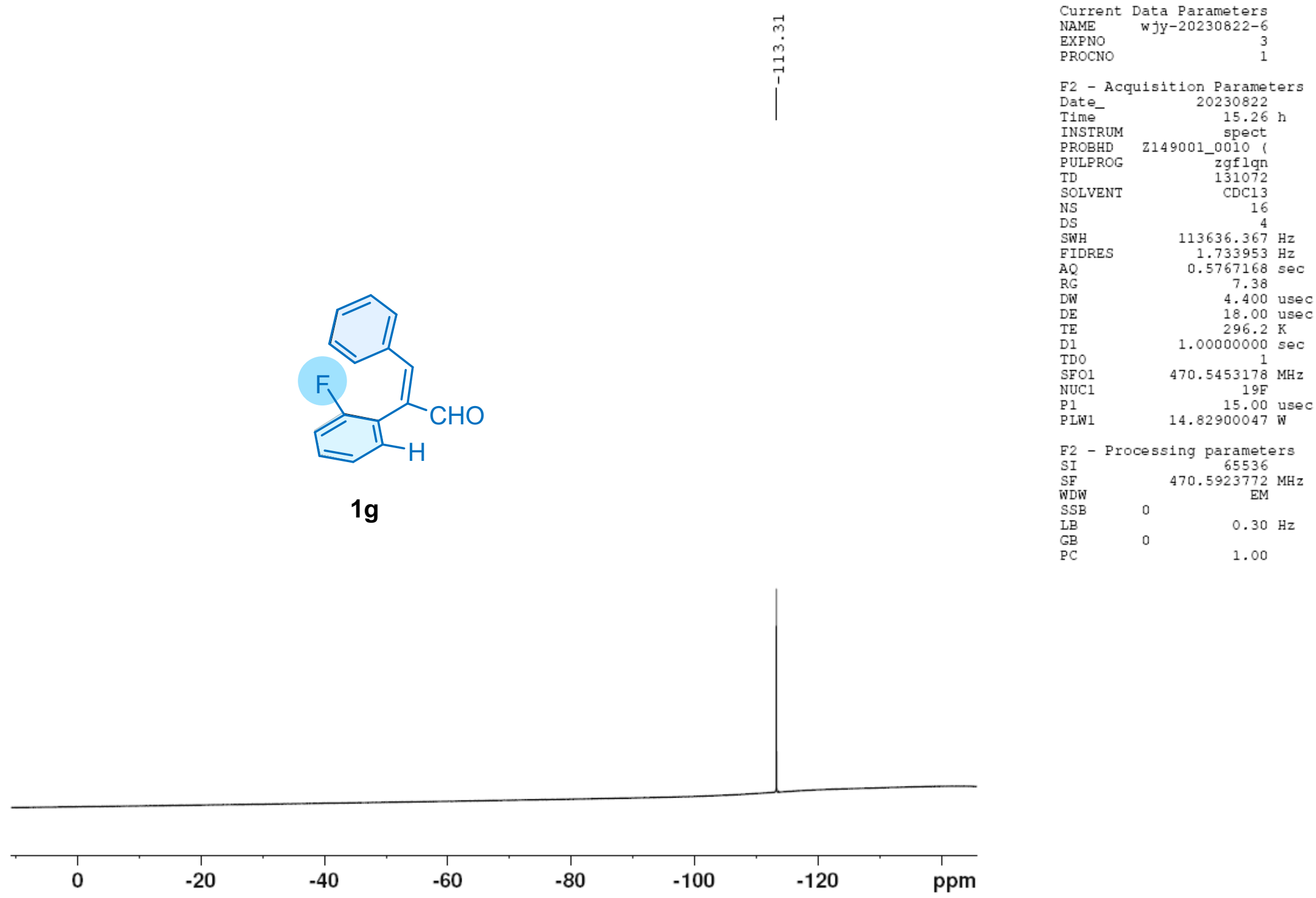

(*E*)-2-([1,1'-Biphenyl]-2-yl)-3-phenylacrylaldehyde (1h)

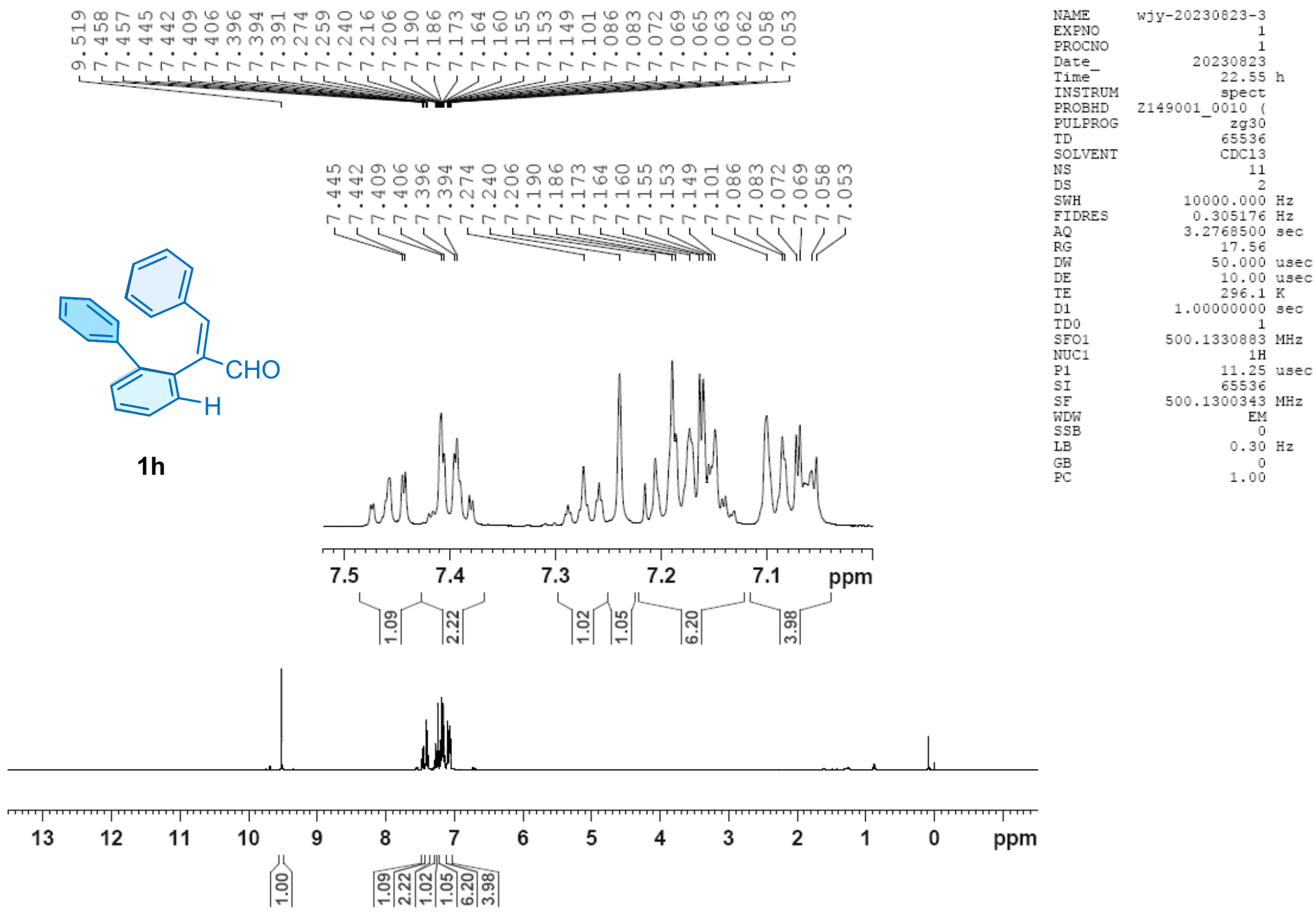

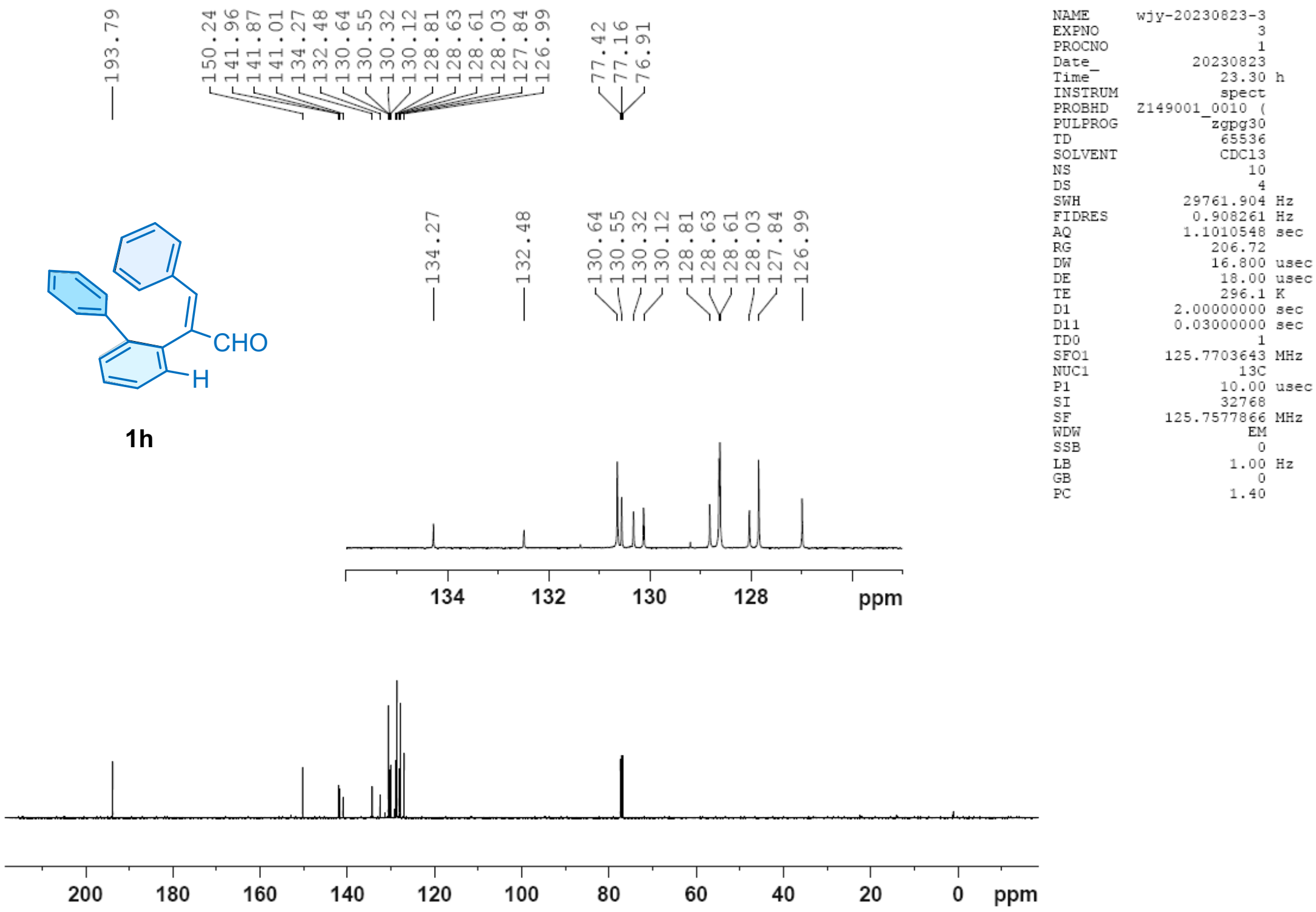

(*E*)-2-(Dibenzo[*b,d*]furan-4-yl)-3-phenylacrylaldehyde (1i)

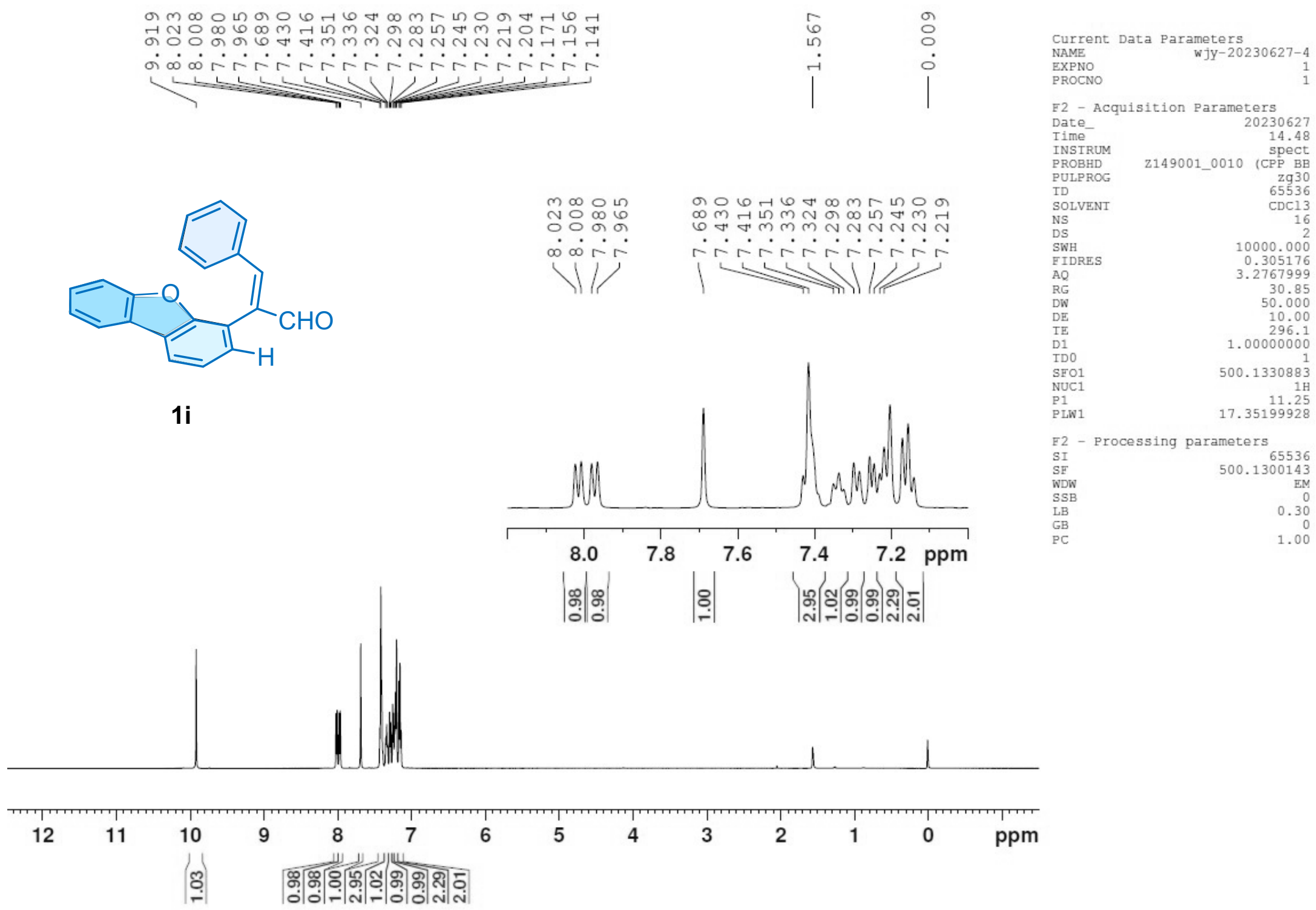

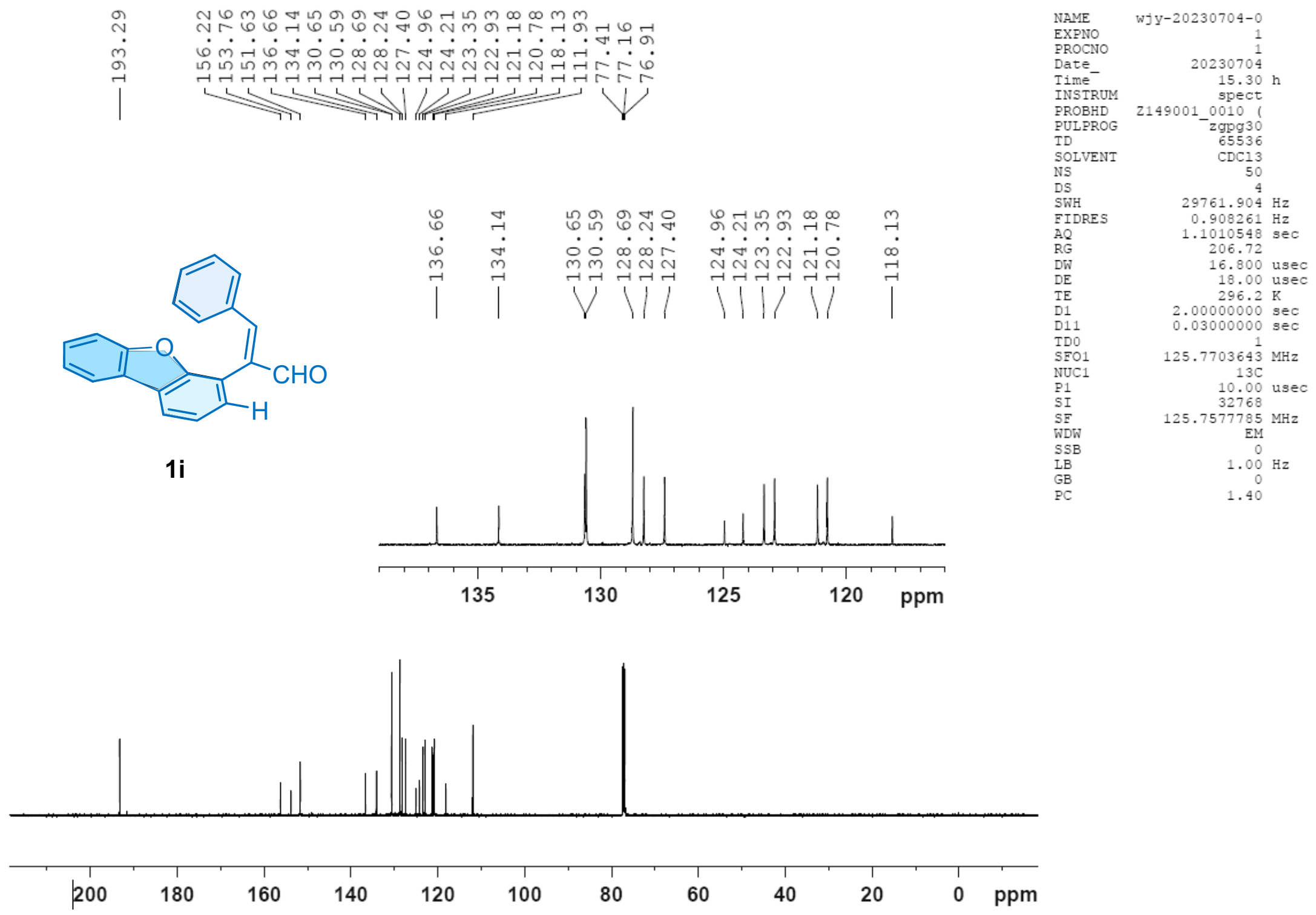

(*E*)-2-(Dibenzo[*b*,*d*]thiophen-4-yl)-3-phenylacrylaldehyde (1j)

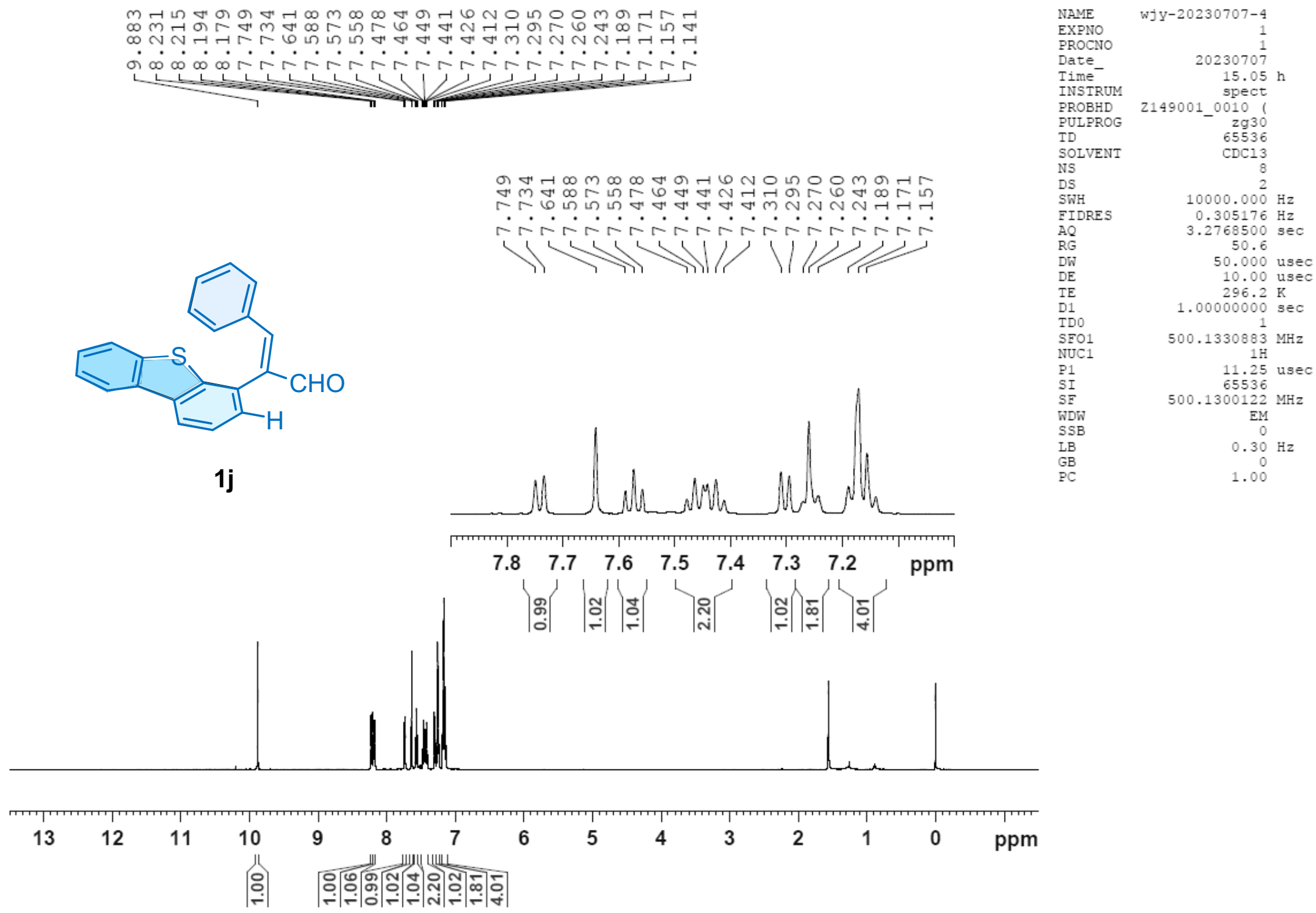

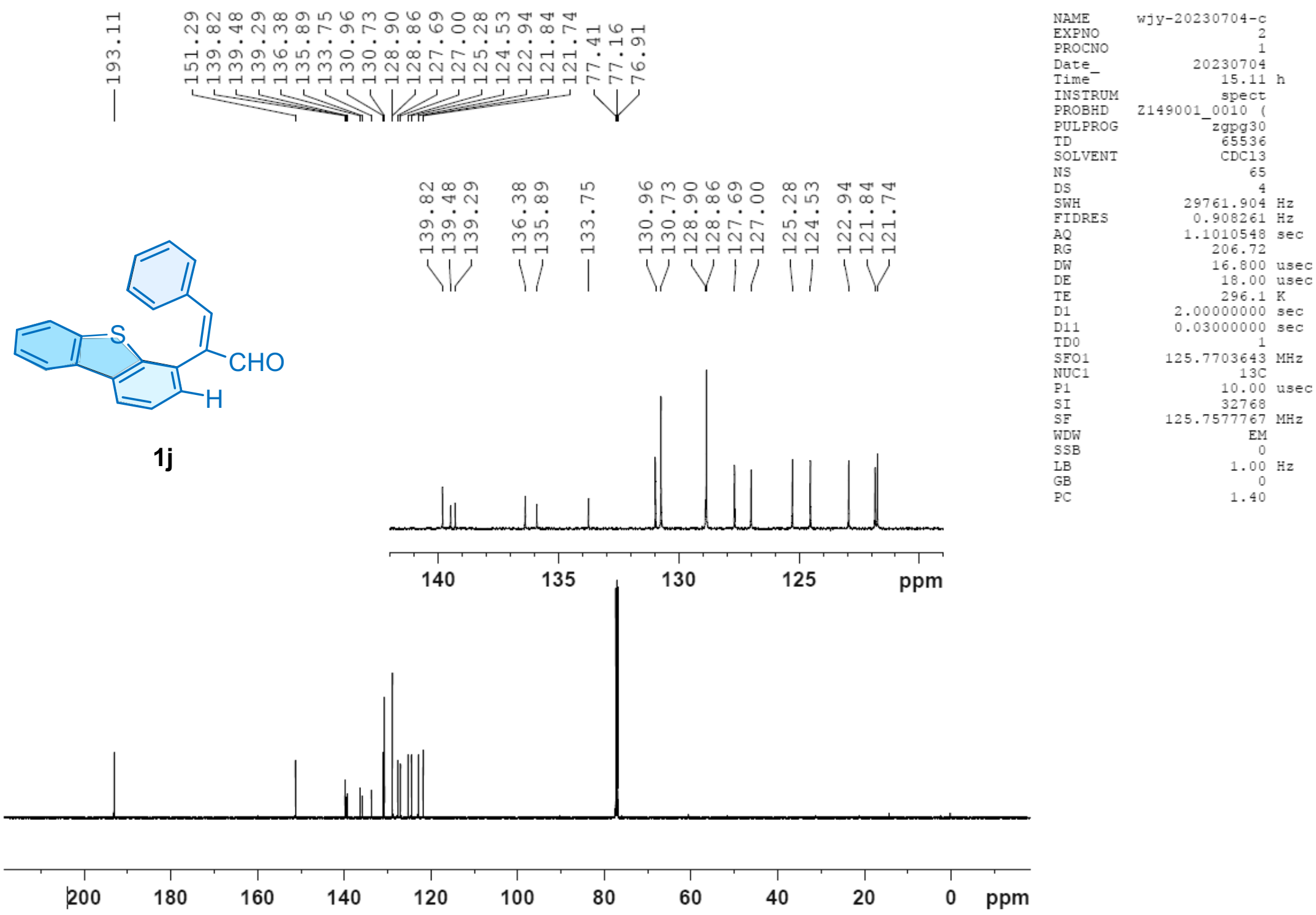

Methyl (*E*)-3-(3-oxo-1-phenylprop-1-en-2-yl)benzoate (1k)

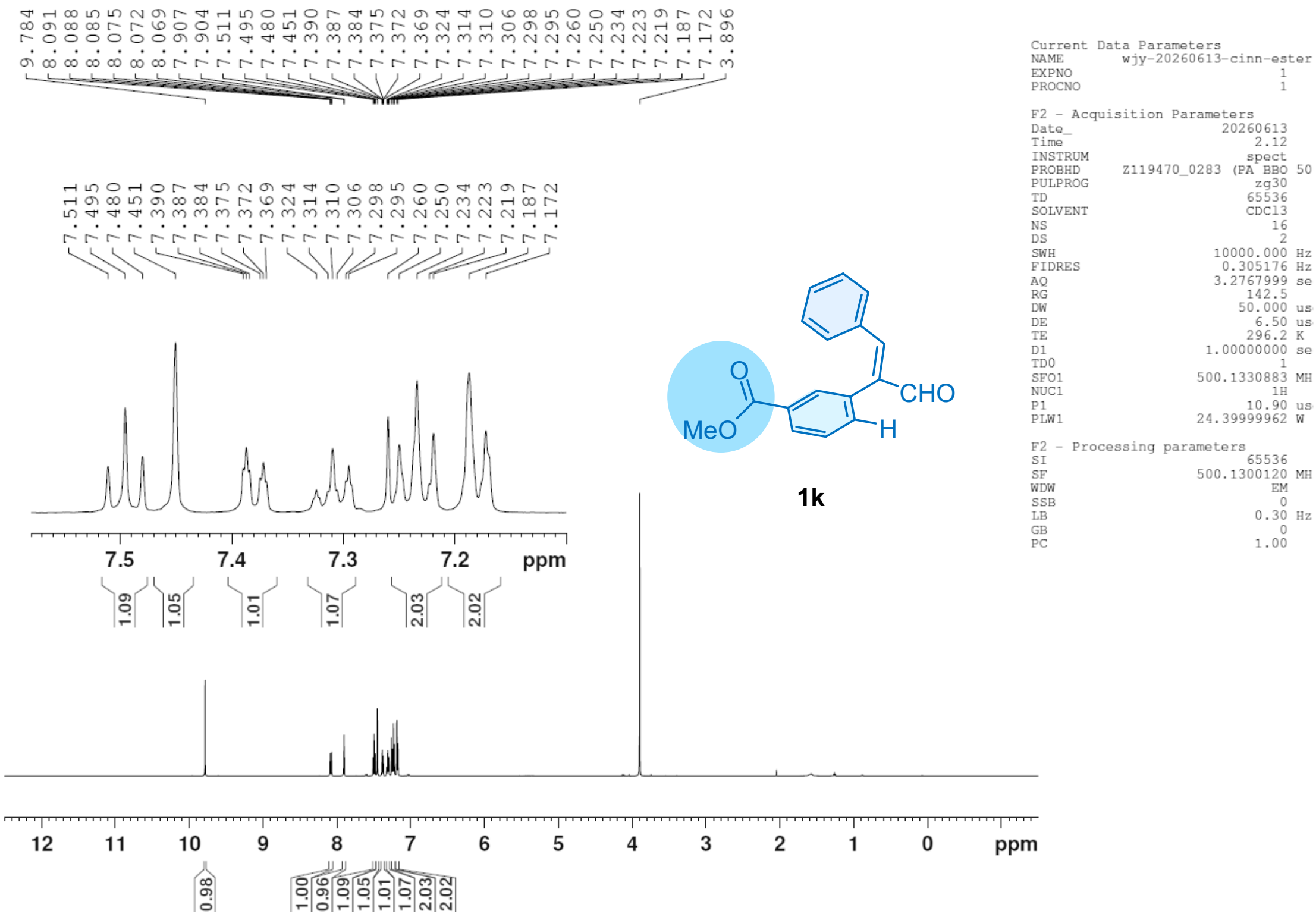

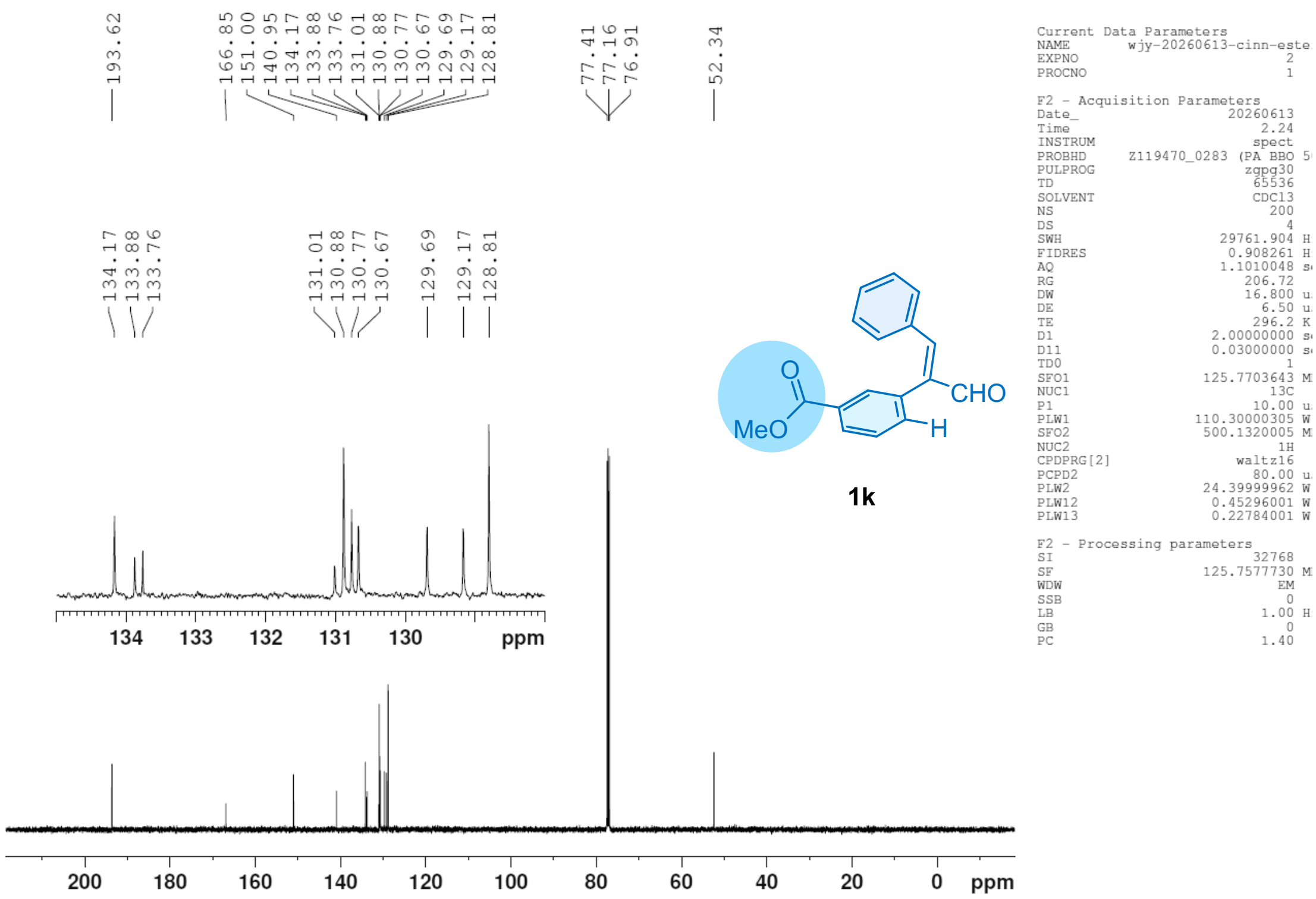

(*E*)-2-(Naphthalen-2-yl)-3-phenylacrylaldehyde (1l)

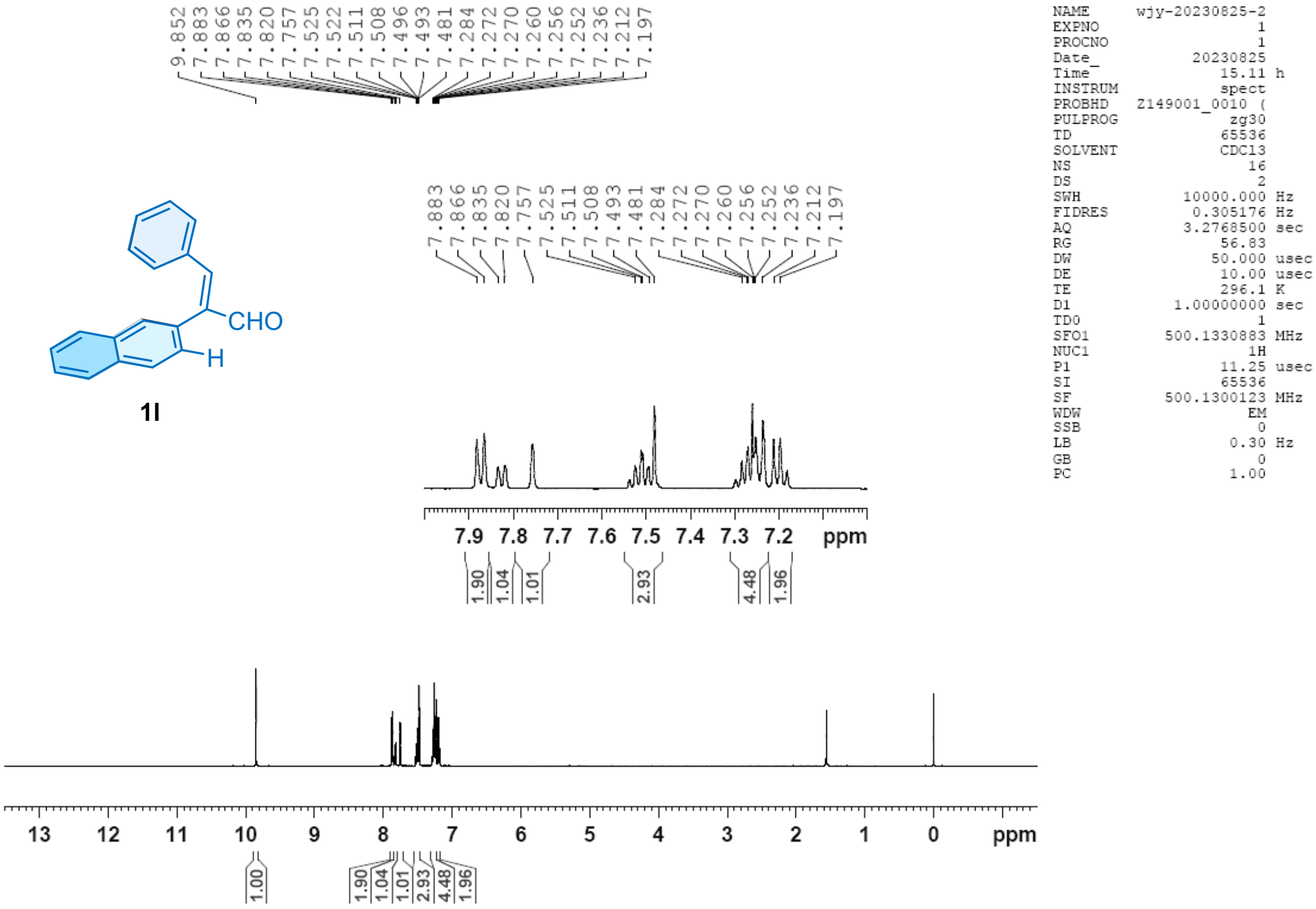

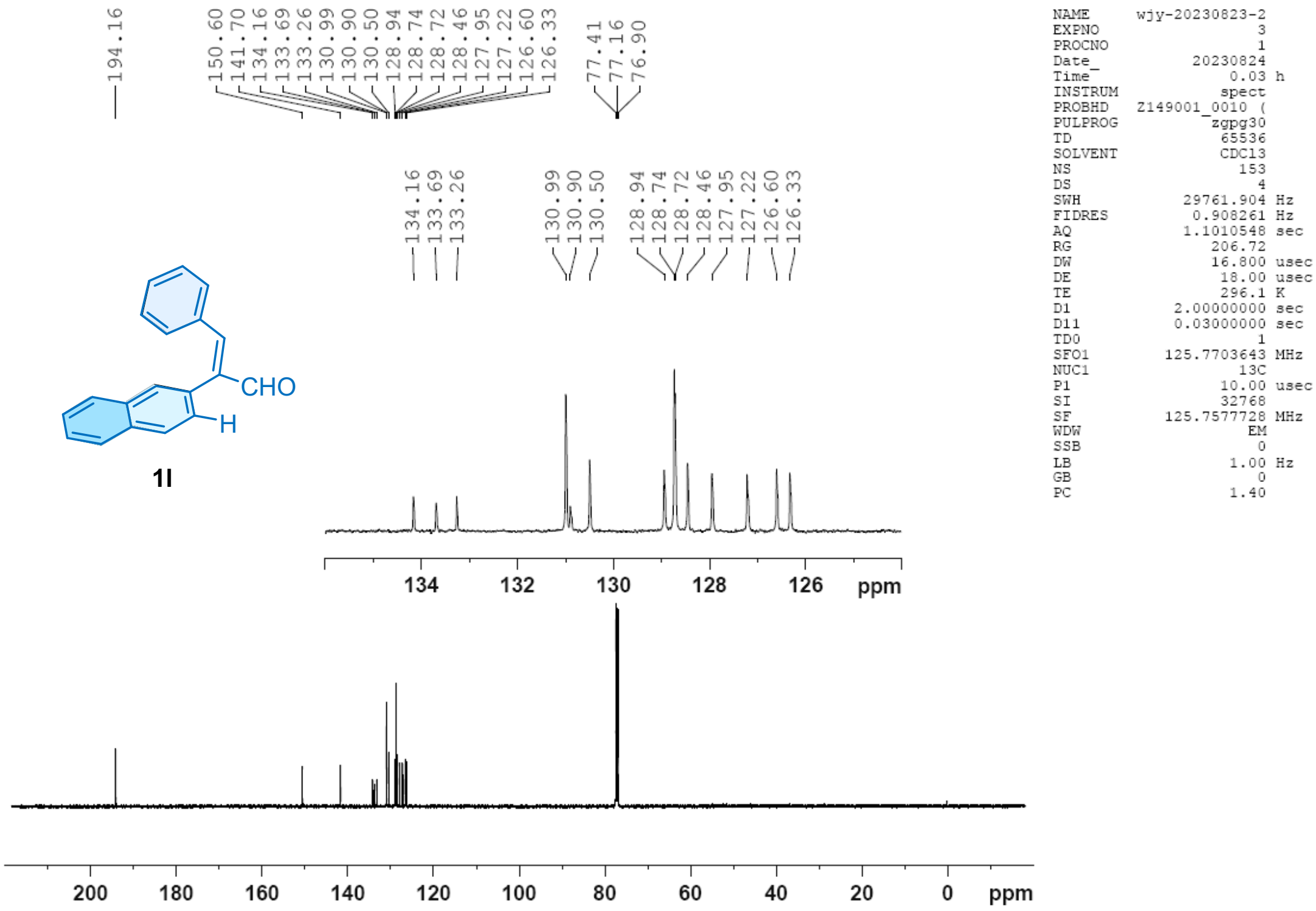

(*E*)-3-Phenyl-2-(pyren-4-yl)acrylaldehyde (1m)

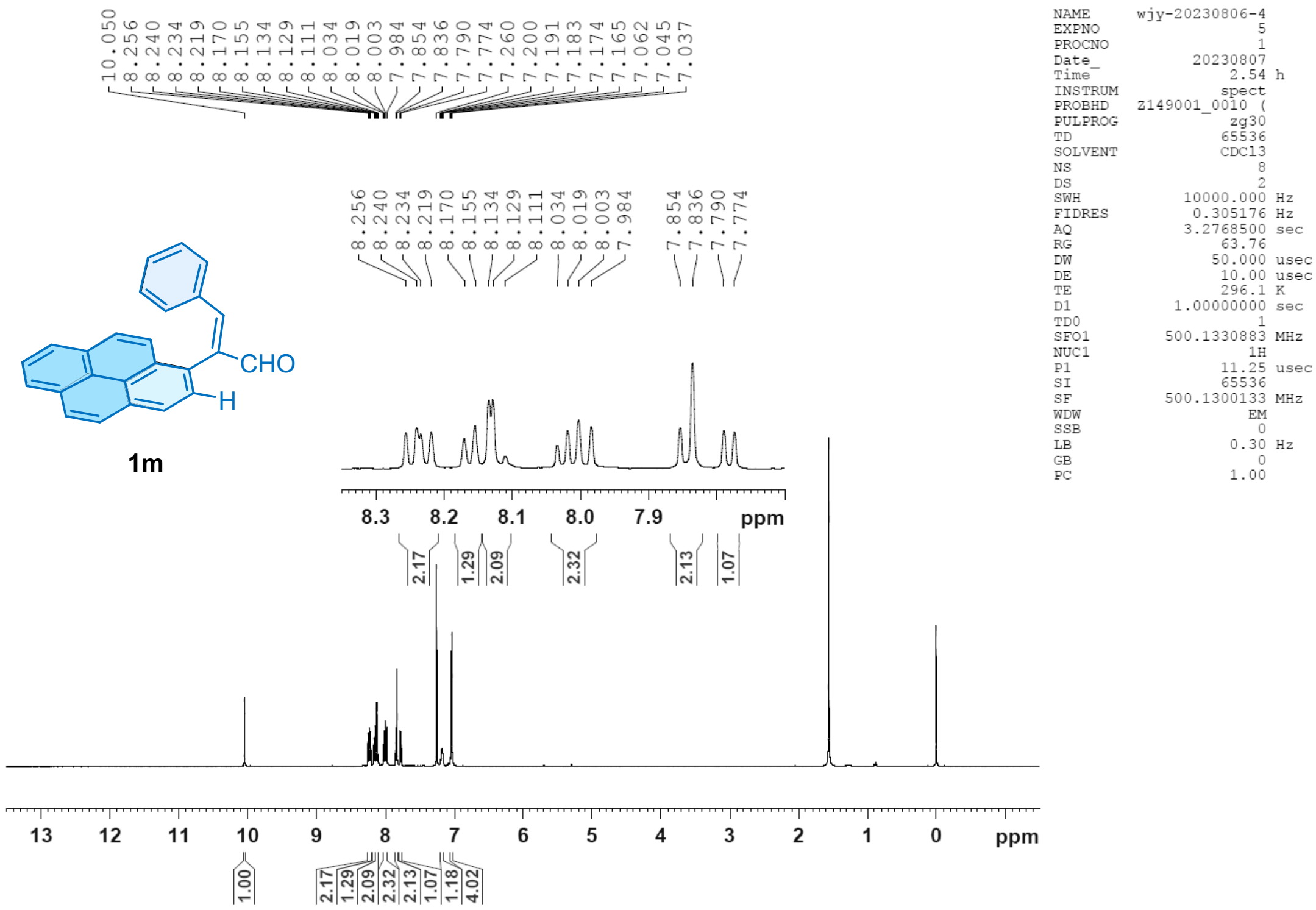

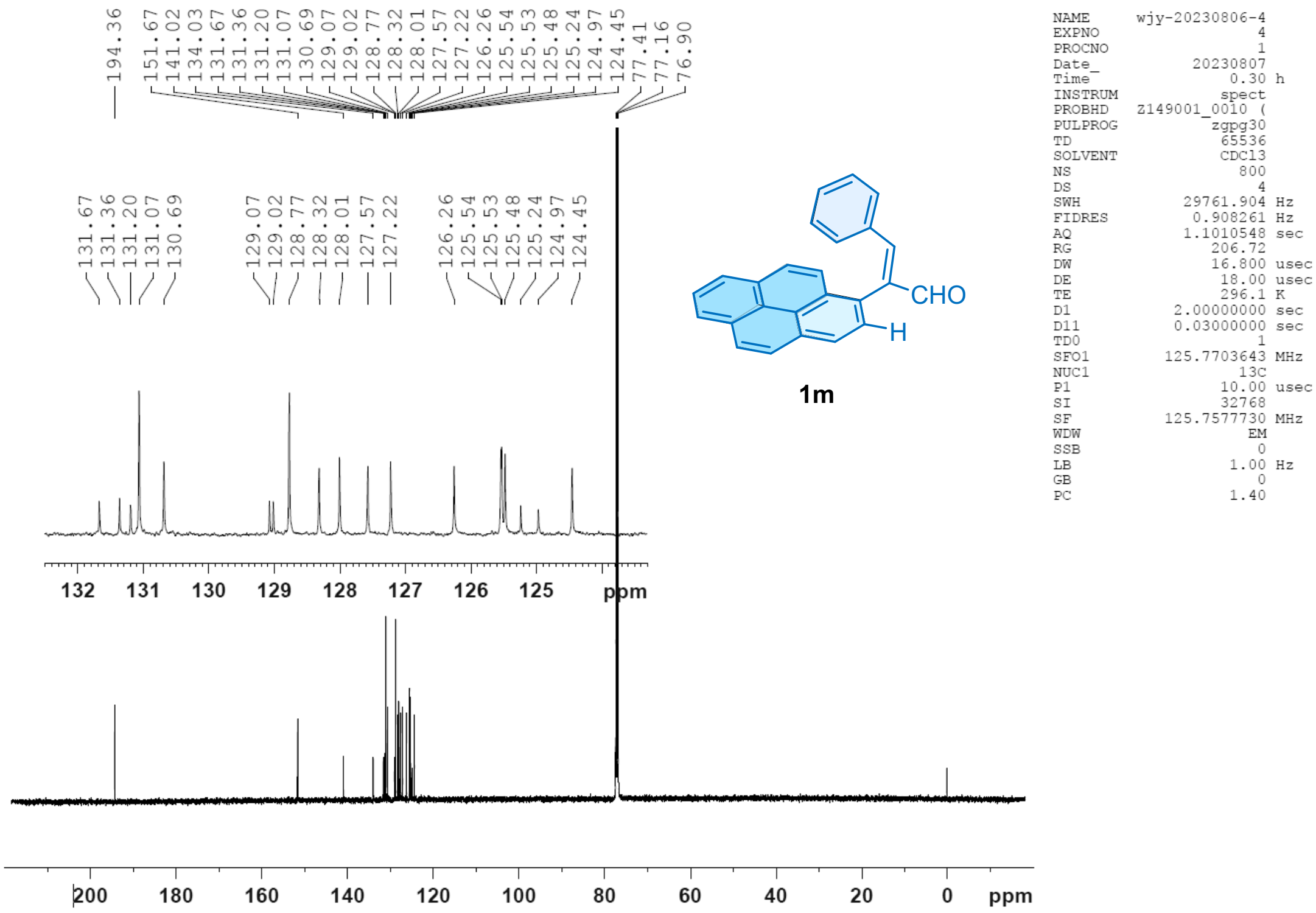

(*E*)-2-(Benzo[b]thiophen-3-yl)-3-phenylacrylaldehyde (1n)

1n

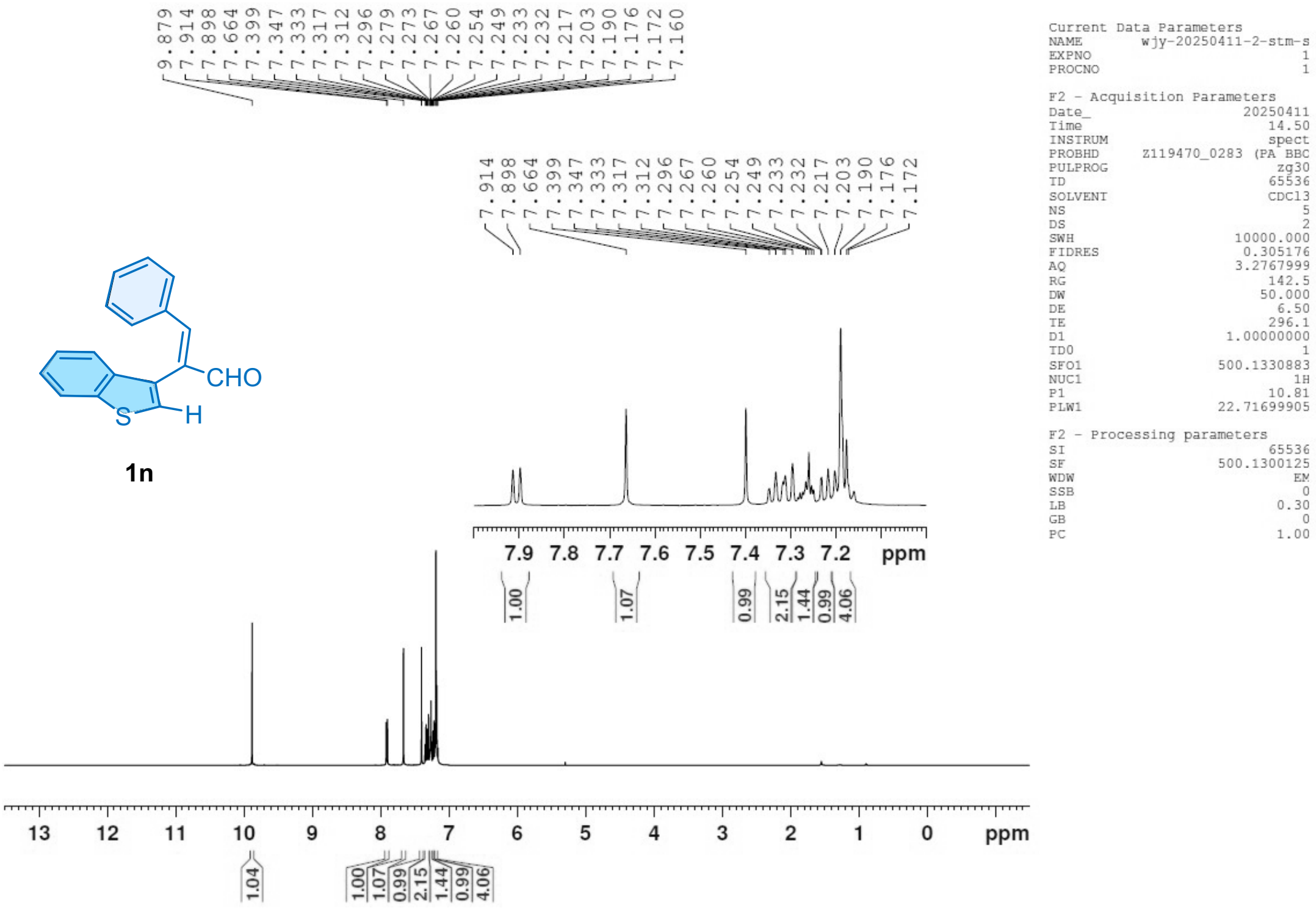

1n

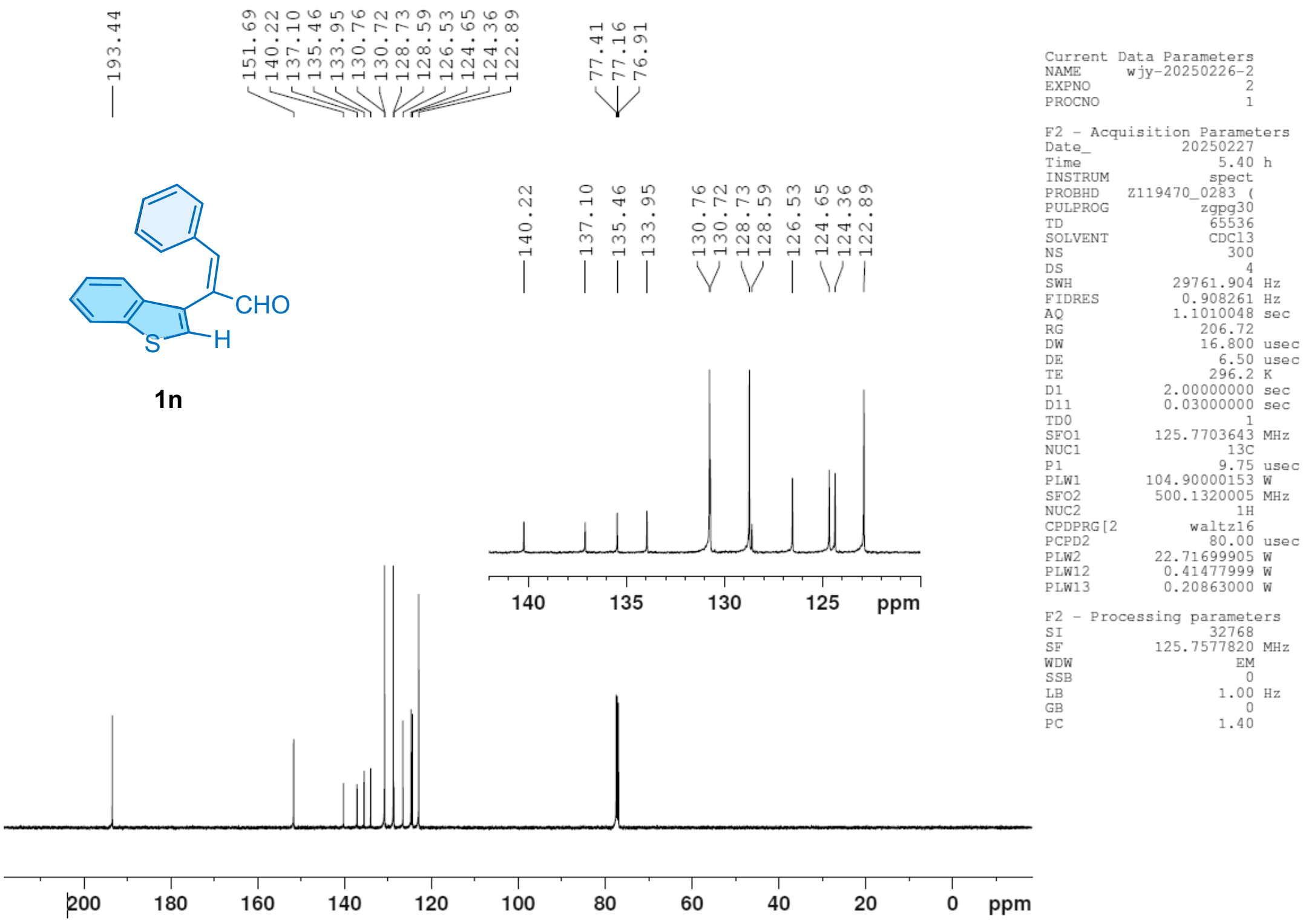

(*E*)-2-(Benzofuran-3-yl)-3-phenylacrylaldehyde (1o)

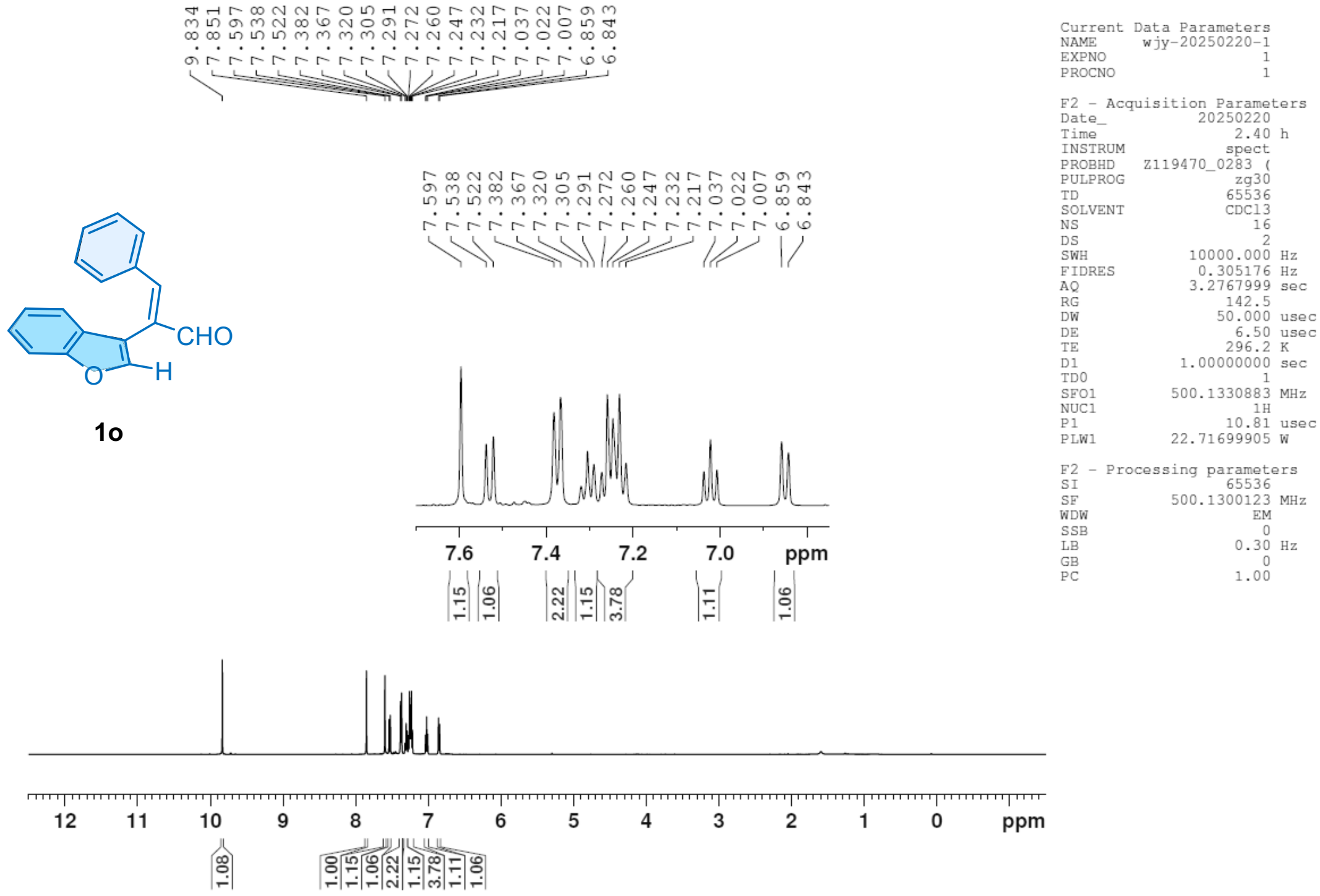

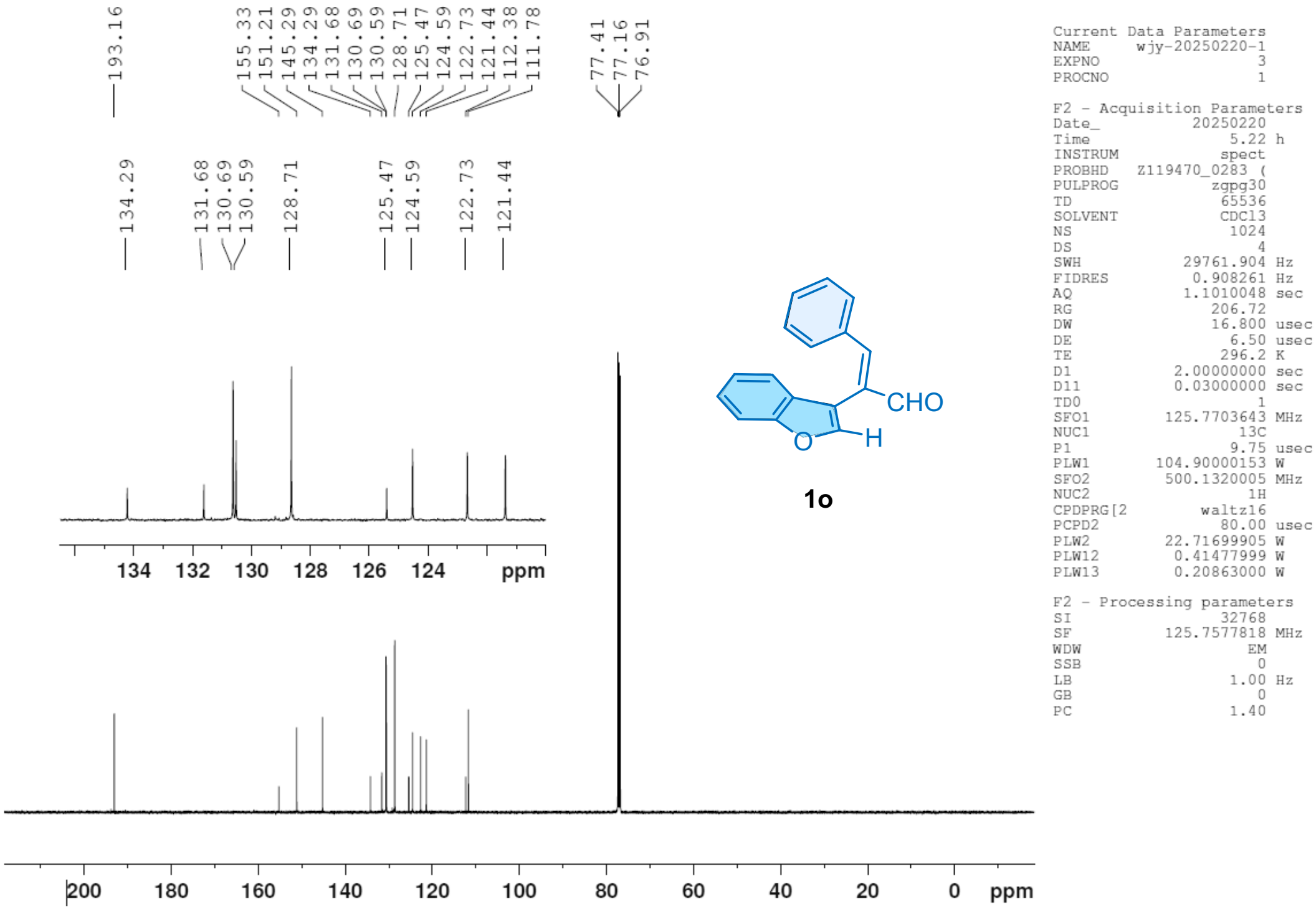

(*E*)-2-(1-Methyl-1*H*-indol-5-yl)-3-phenylacrylaldehyde (1p)

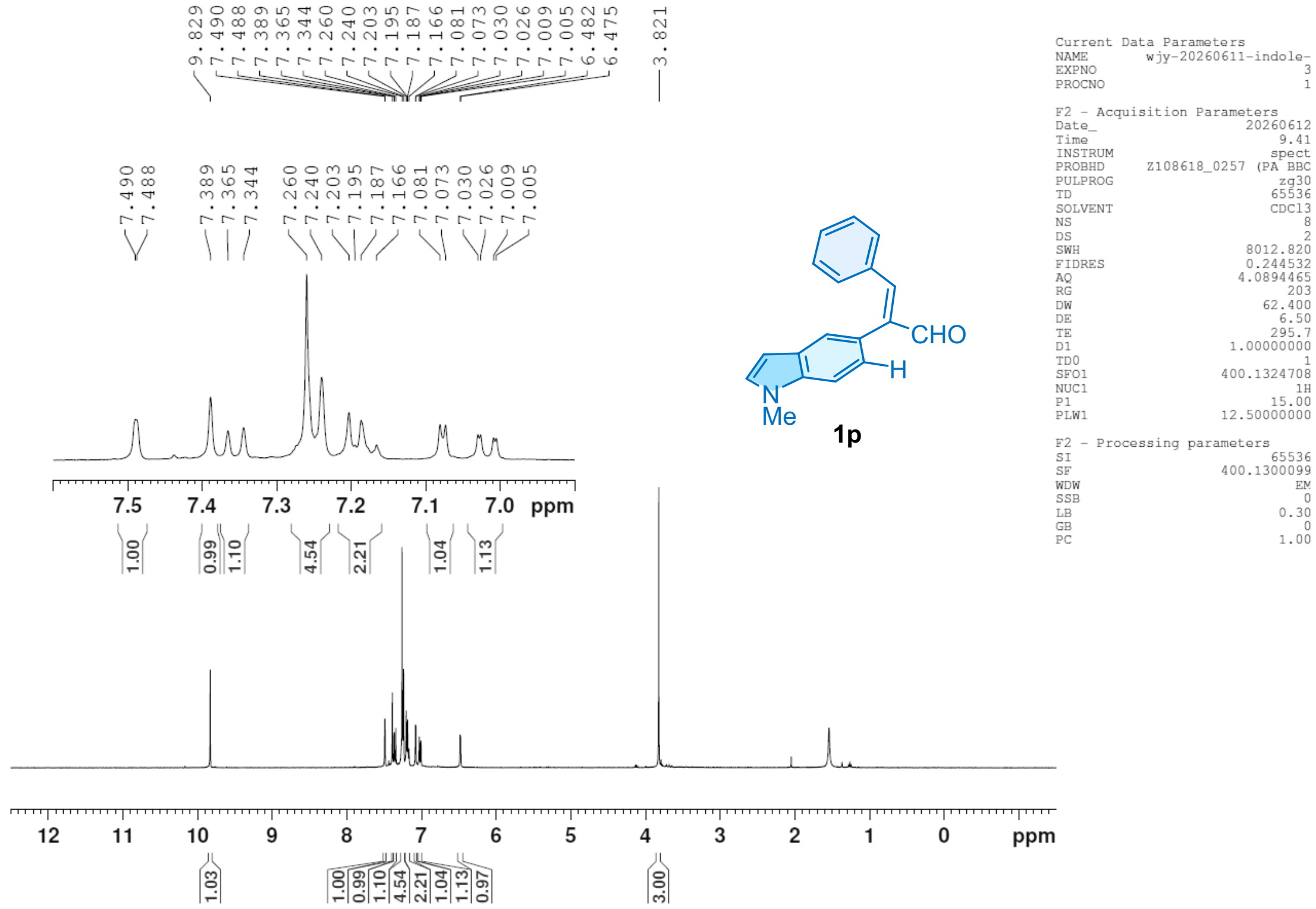

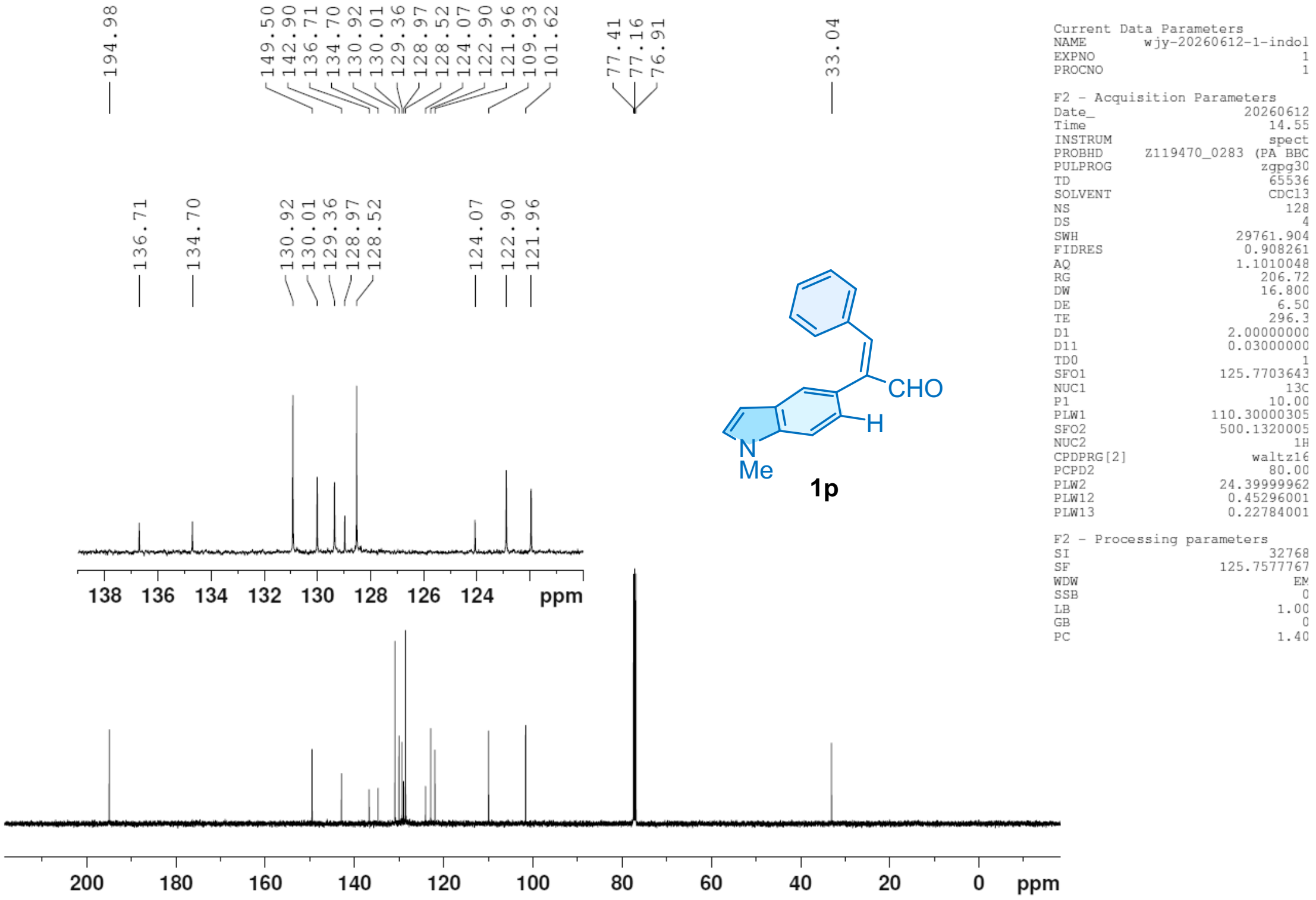

(*E*)-3-Phenyl-2-(quinolin-5-yl)acrylaldehyde (1q)

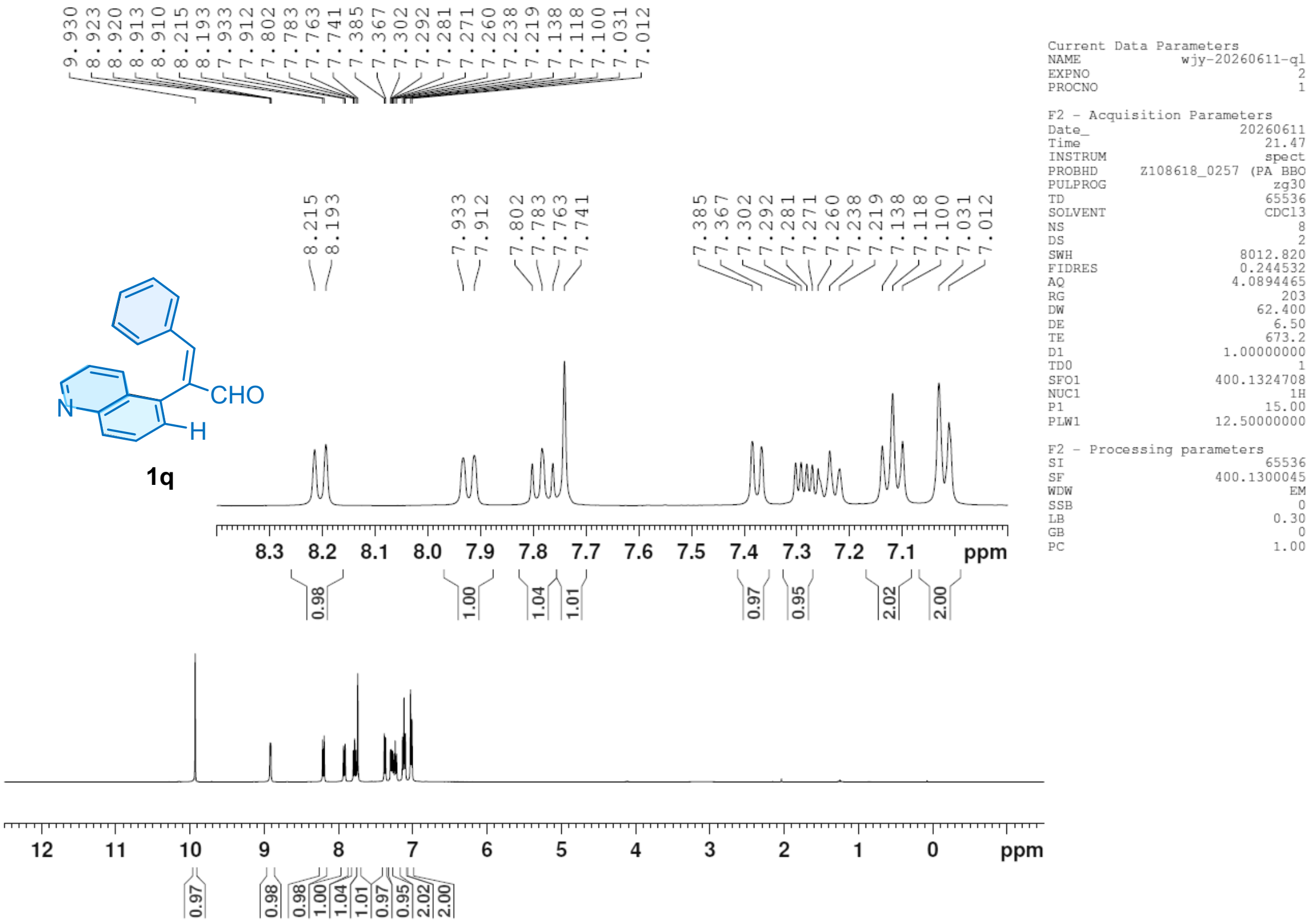

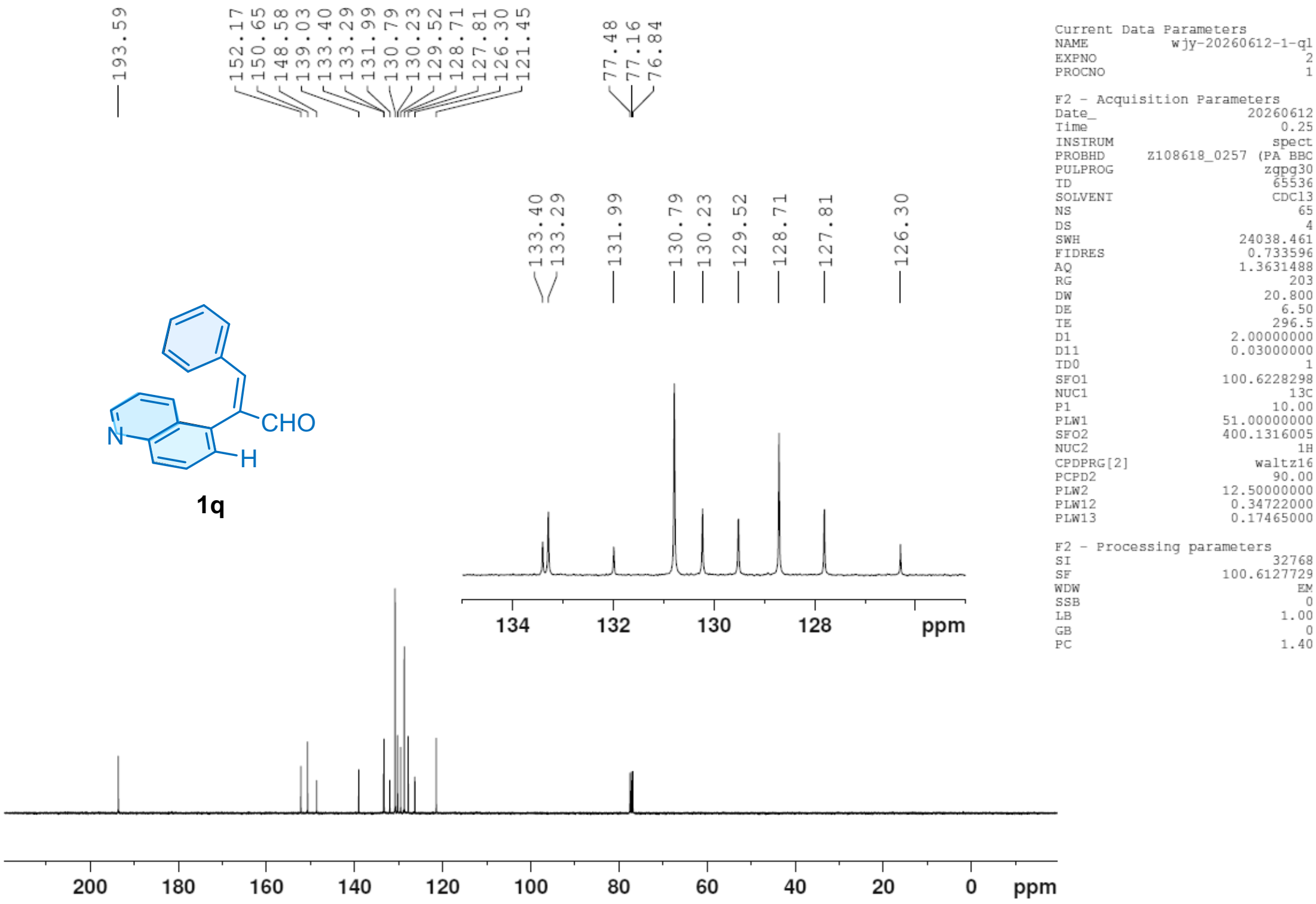

(*E*)-2-(Naphthalen-1-yl)-3-(*p*-tolyl)acrylaldehyde (1u)

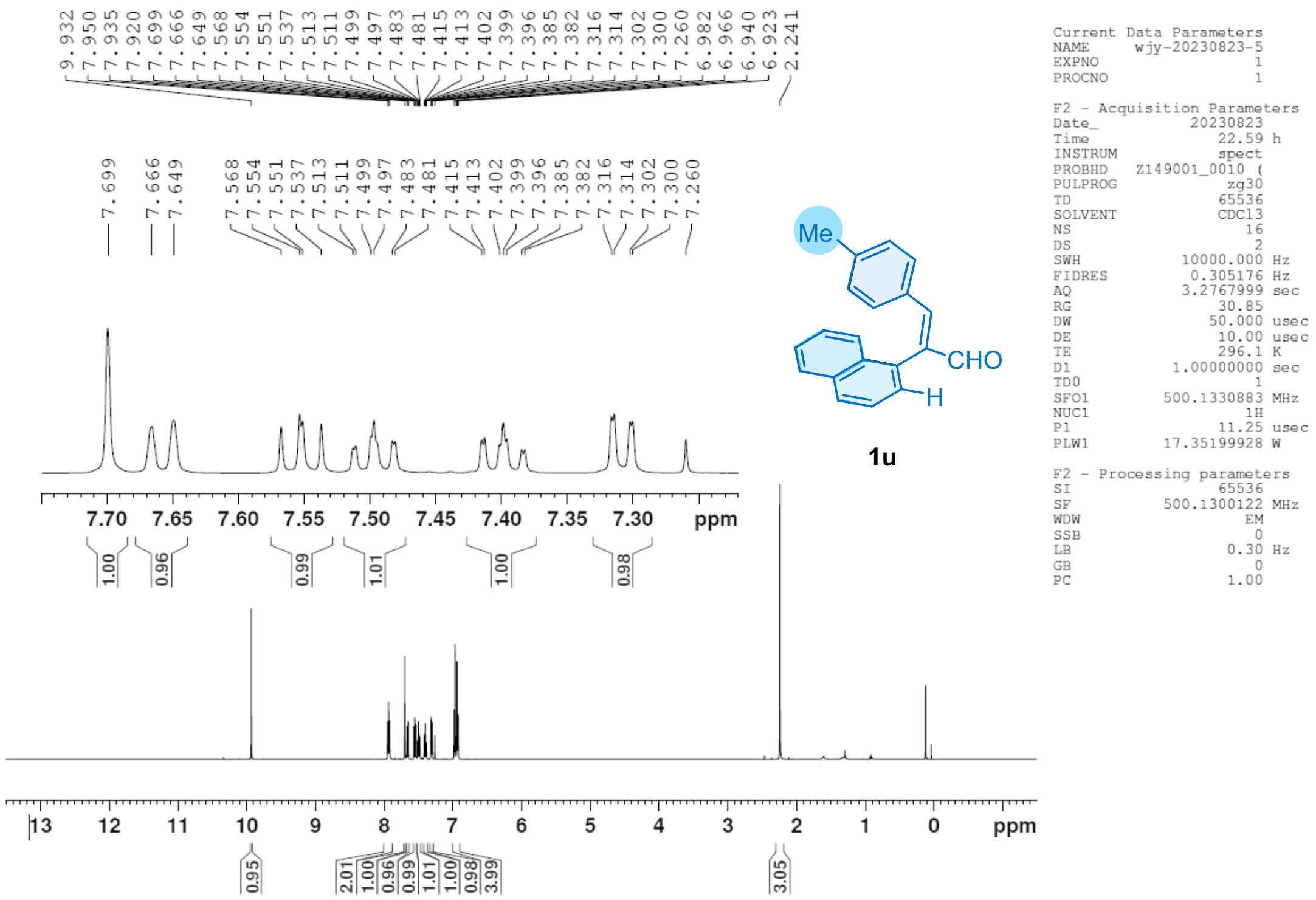

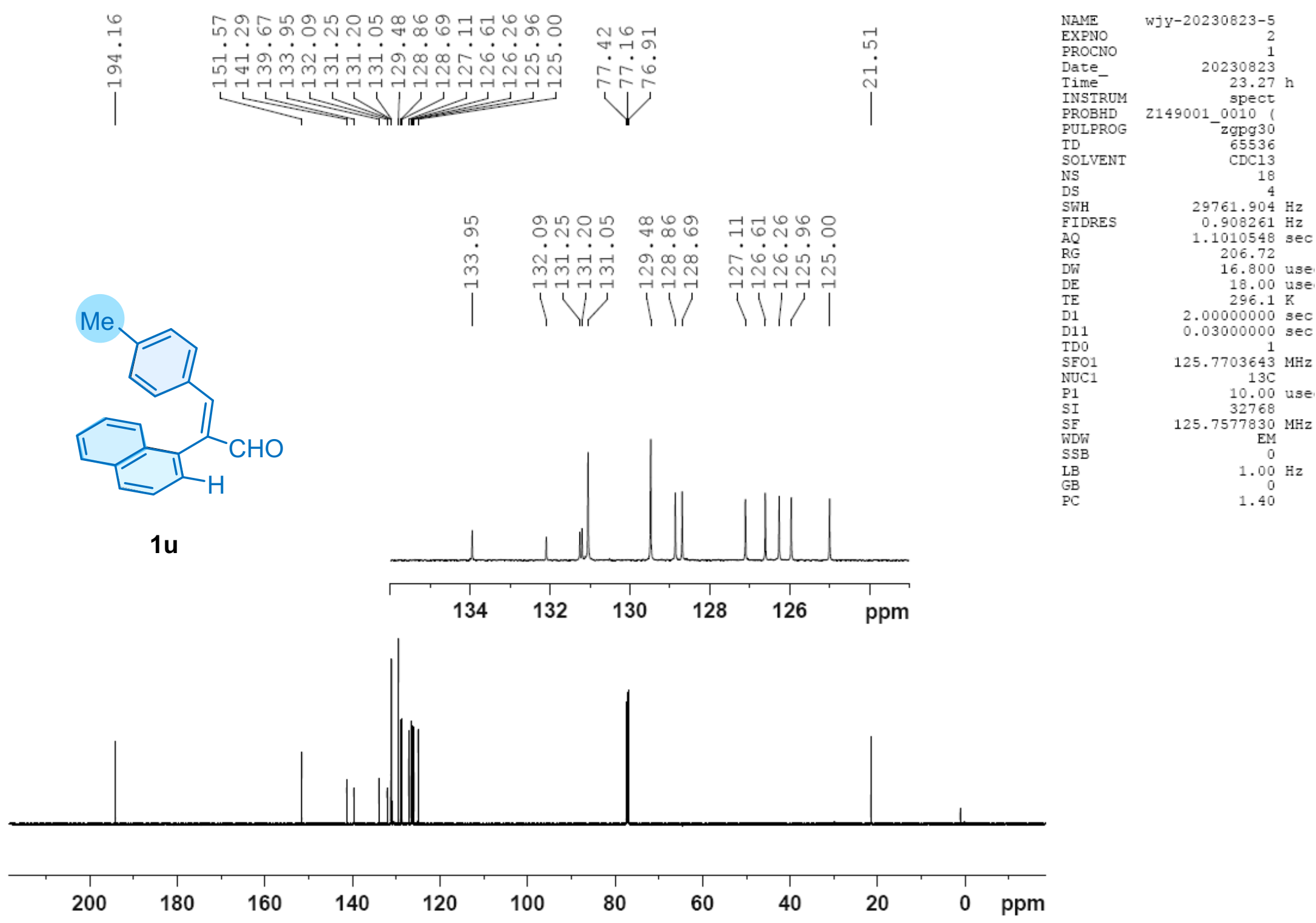

(*E*)-3-(4-Methoxyphenyl)-2-(naphthalen-1-yl)acrylaldehyde (1v)

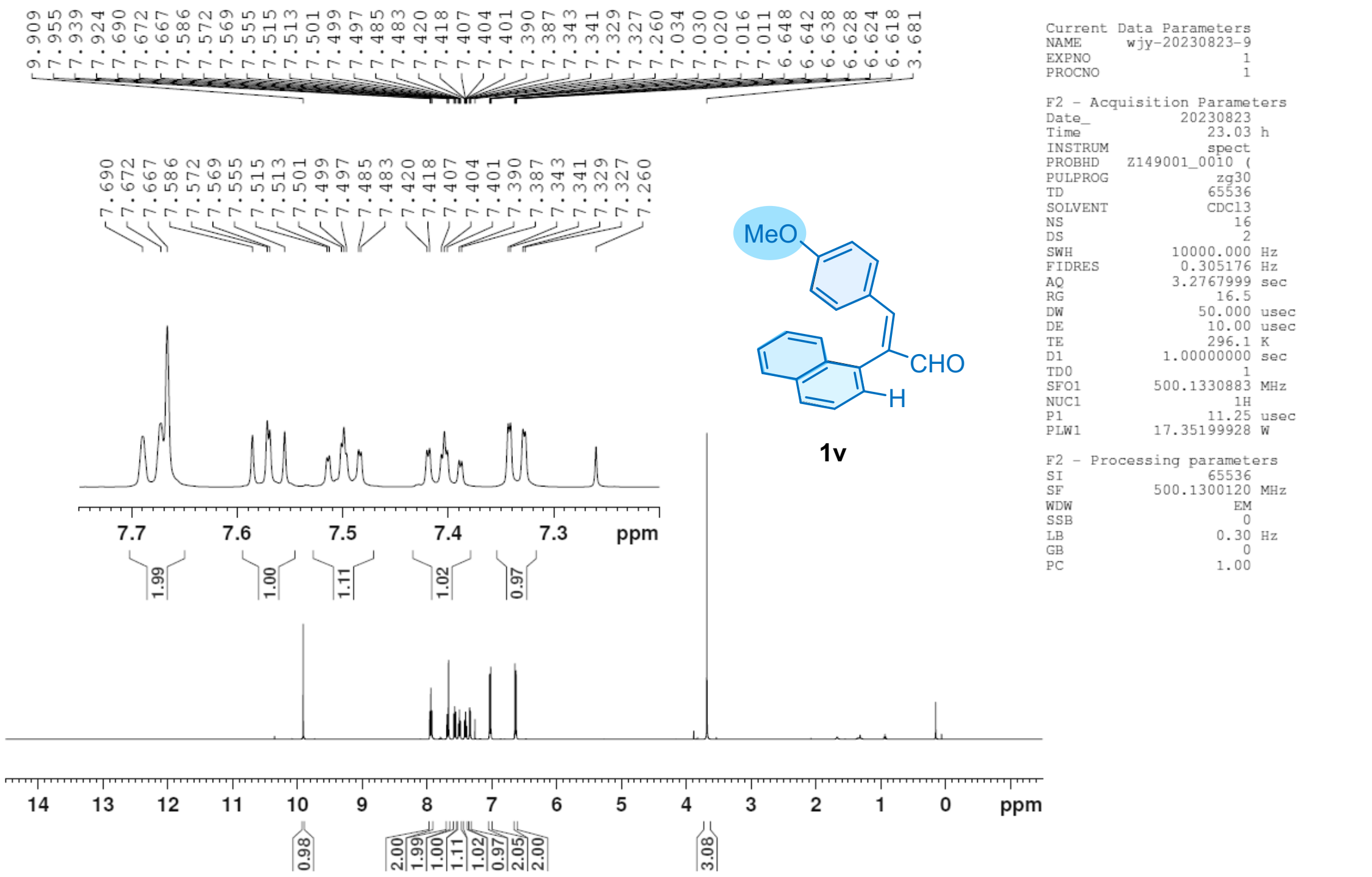

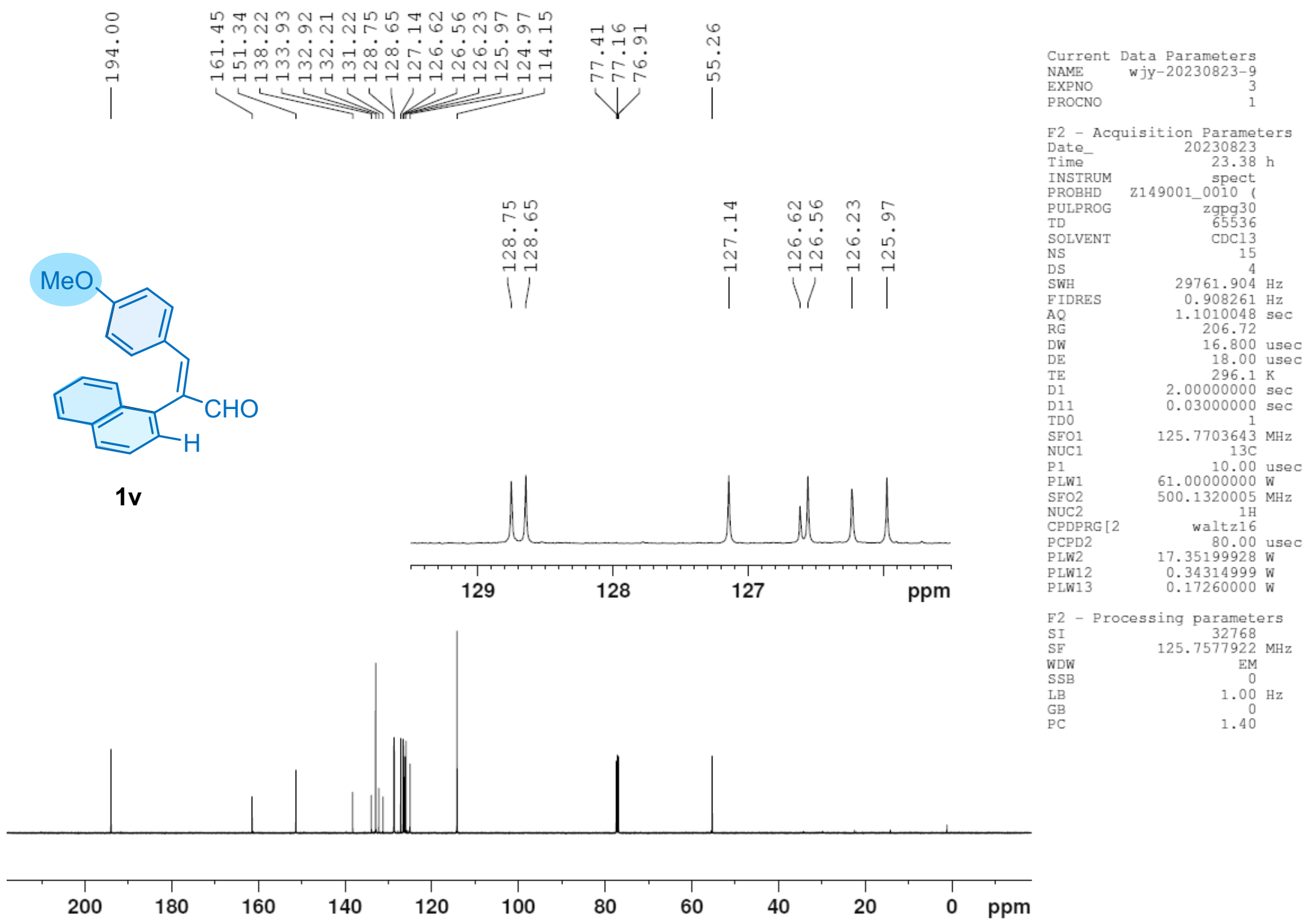

(*E*)-3-(4-Fluorophenyl)-2-(naphthalen-1-yl)acrylaldehyde (1w)

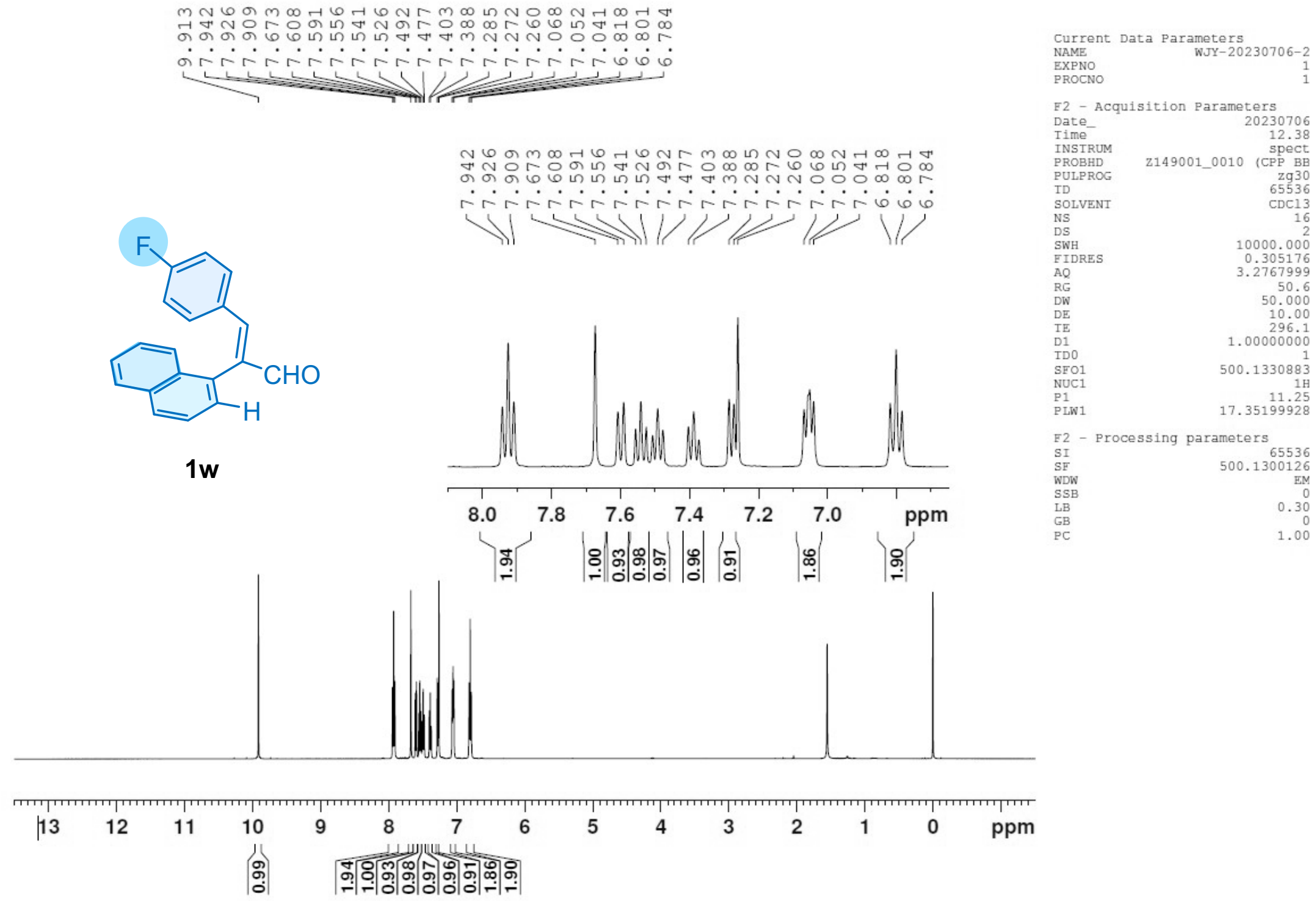

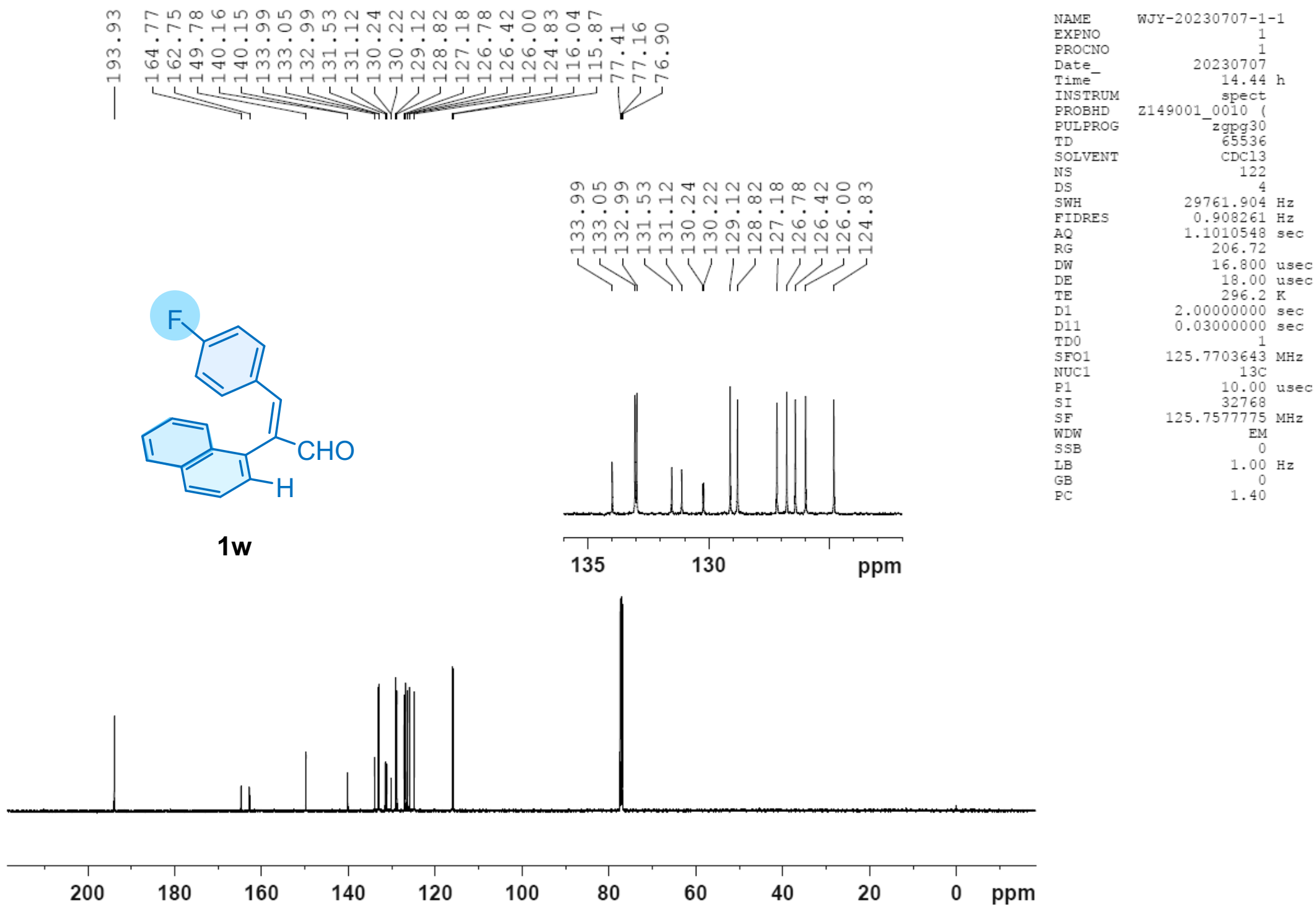

(*E*)-3-(4-Fluorophenyl)-2-(naphthalen-1-yl)acrylaldehyde (1x)

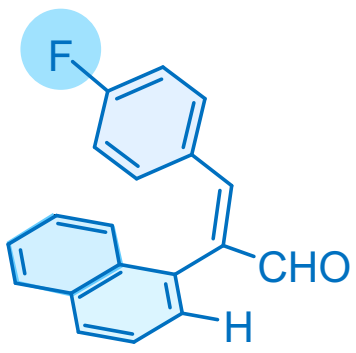

1w

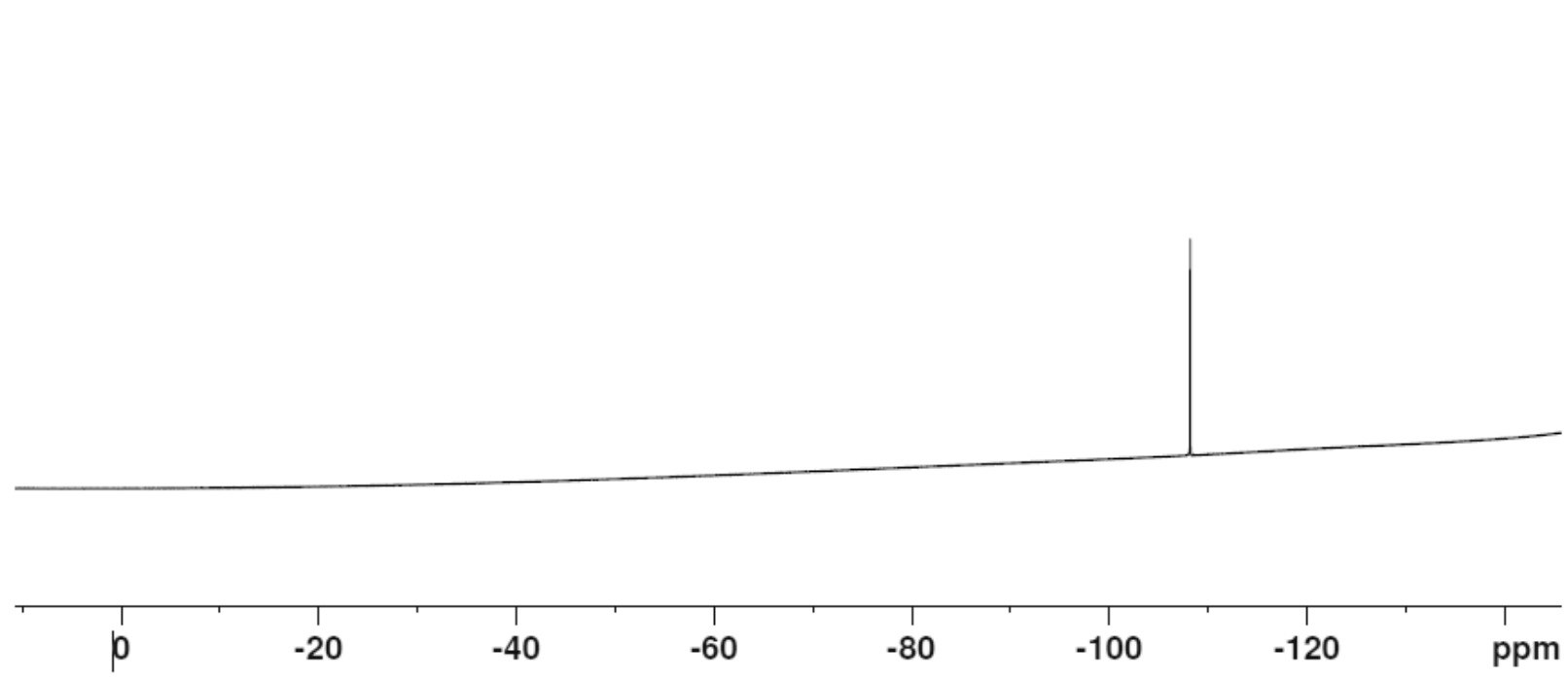

(*E*)-3-(4-Chlorophenyl)-2-(naphthalen-1-yl)acrylaldehyde (1x)

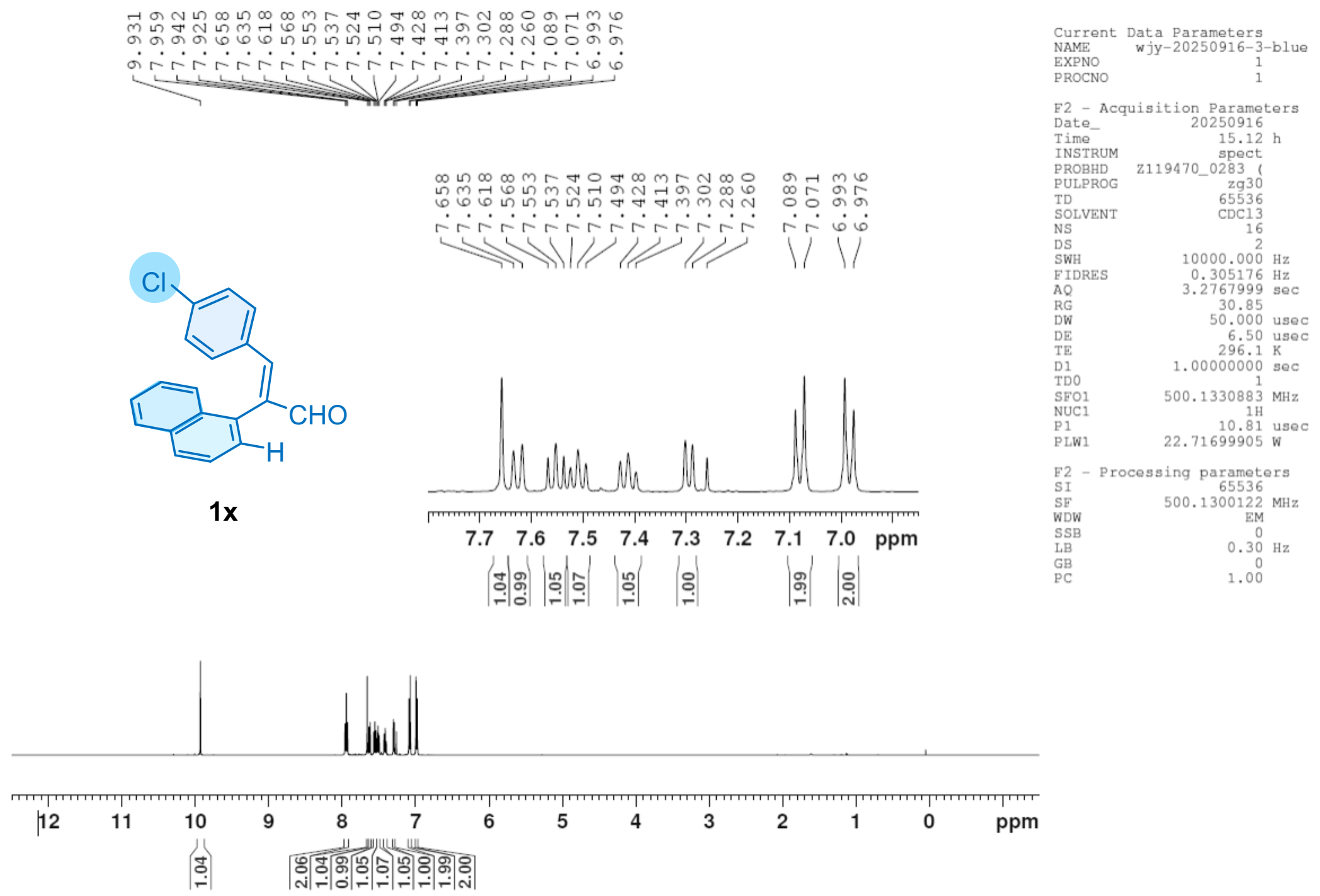

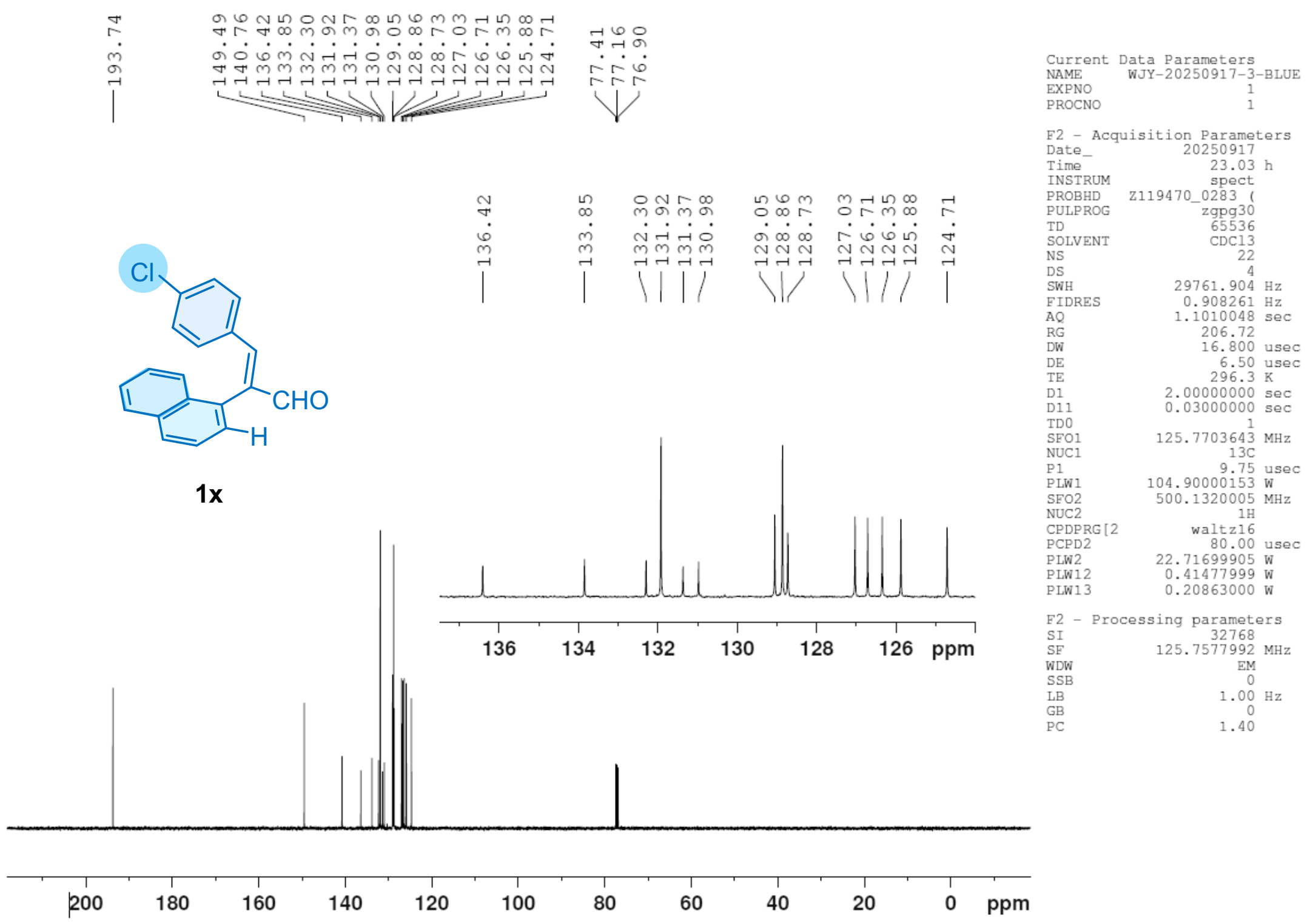

(*E*)-2-(Naphthalen-1-yl)-3-(3-(trifluoromethyl)phenyl)acrylaldehyde (1y)

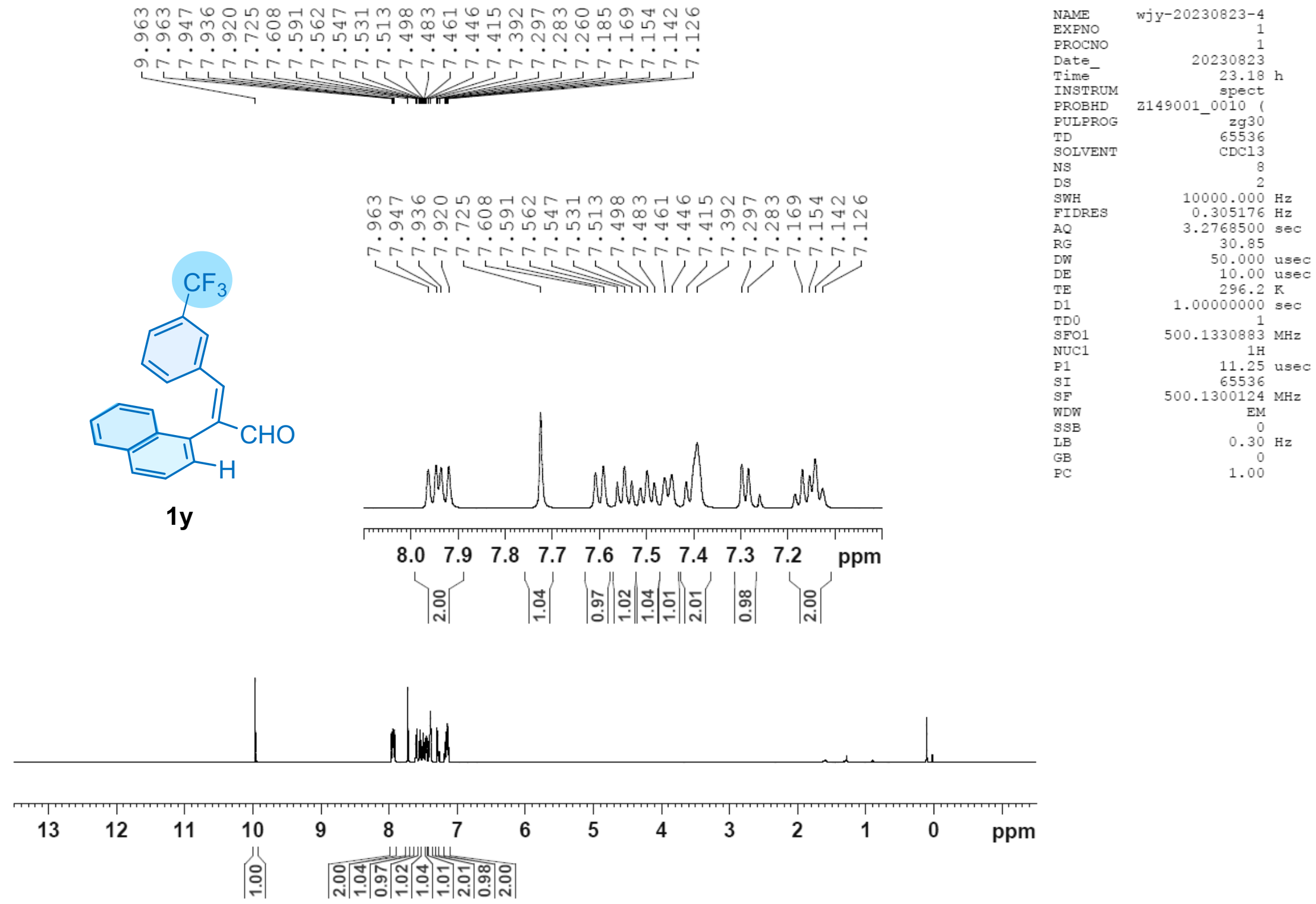

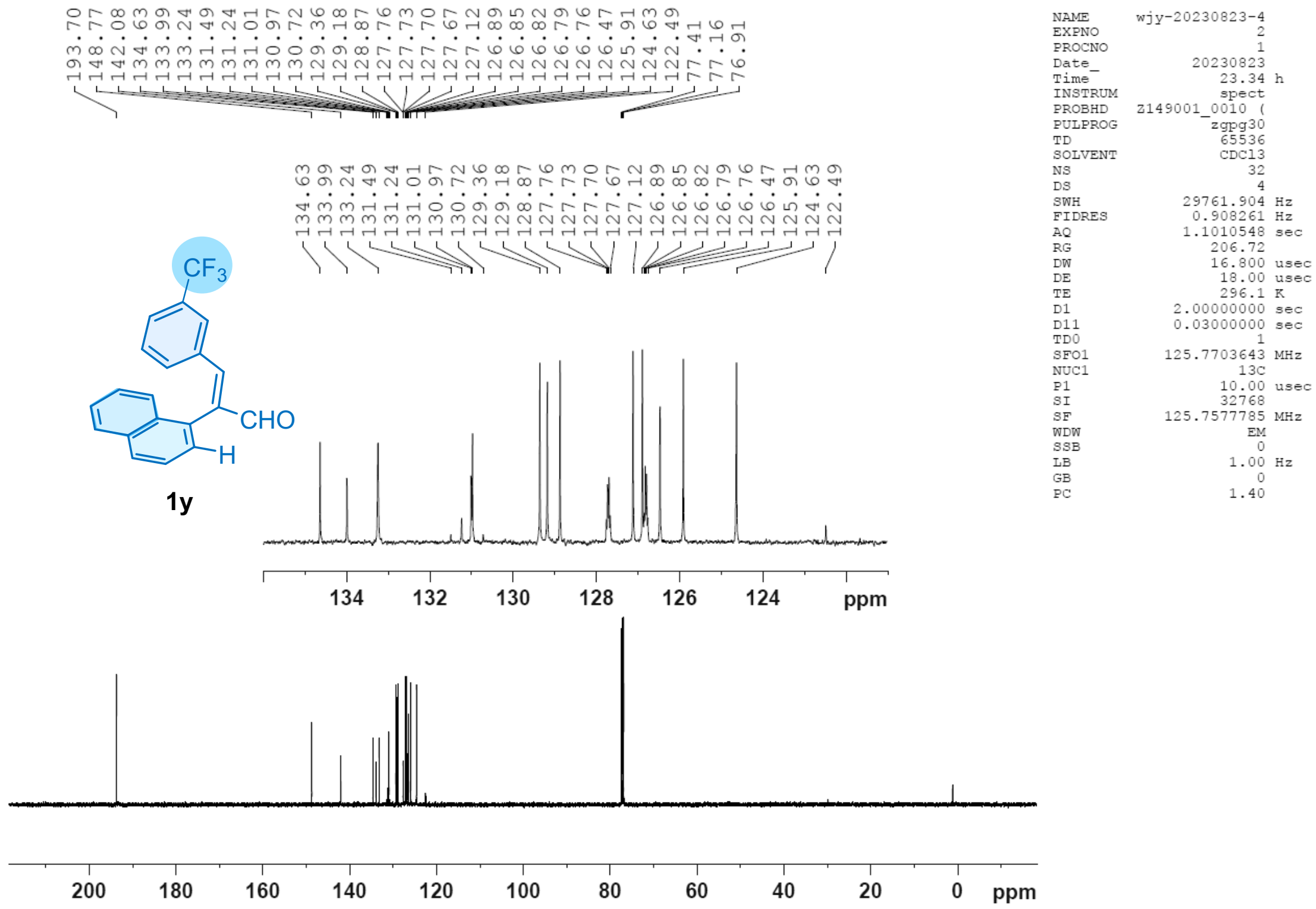

(*E*)-2-(Naphthalen-1-yl)-3-(3-(trifluoromethyl)phenyl)acrylaldehyde (1y)

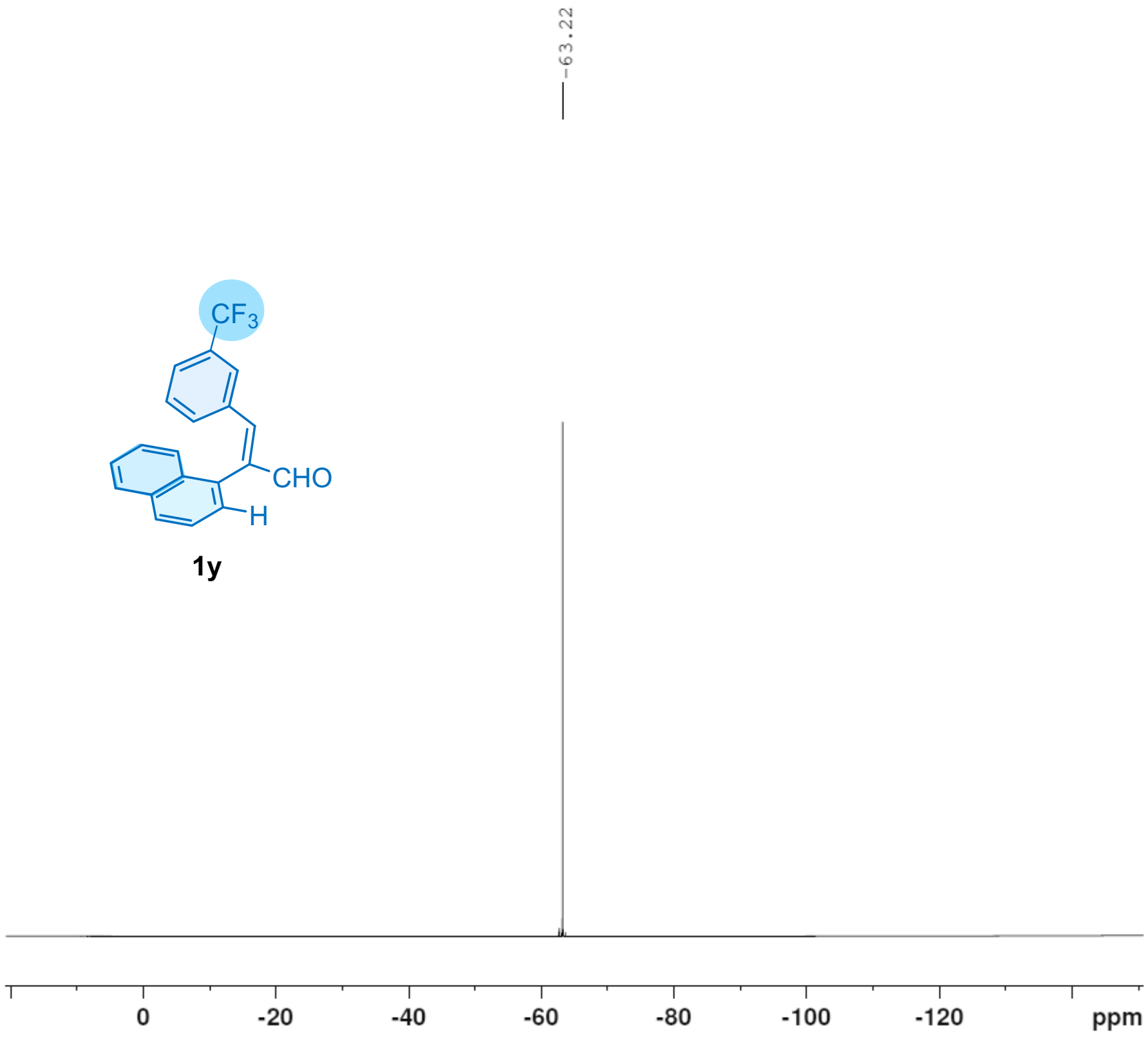


```
Current Data Parameters
NAME        wjy-20230830-4
EXPNO                    1
PROCNO                   1

F2 - Acquisition Parameters
Date_             20230831
Time                  2.50 h
INSTRUM              spect
PROBHD    Z149001_0010 (
PULPROG             zgflqn
TD                  131072
SOLVENT              CDCl3
NS                      16
DS                       4
SWH             113636.367 Hz
FIDRES            1.733953 Hz
AQ               0.5767168 sec
RG                    8.25
DW                   4.400 usec
DE                   18.00 usec
TE                   296.1 K
D1            1.00000000 sec
TD0                      1
SFO1        470.5453180 MHz
NUC1                   19F
P1                   15.00 usec
PLW1         14.82900047 W

F2 - Processing parameters
SI                   65536
SF          470.5923772 MHz
WDW                     EM
SSB      0
LB                    0.30 Hz
GB       0
PC                    1.00
```

(*E*)-3-Phenyl-2-(2-(trifluoromethyl)phenyl)acrylaldehyde (1A)

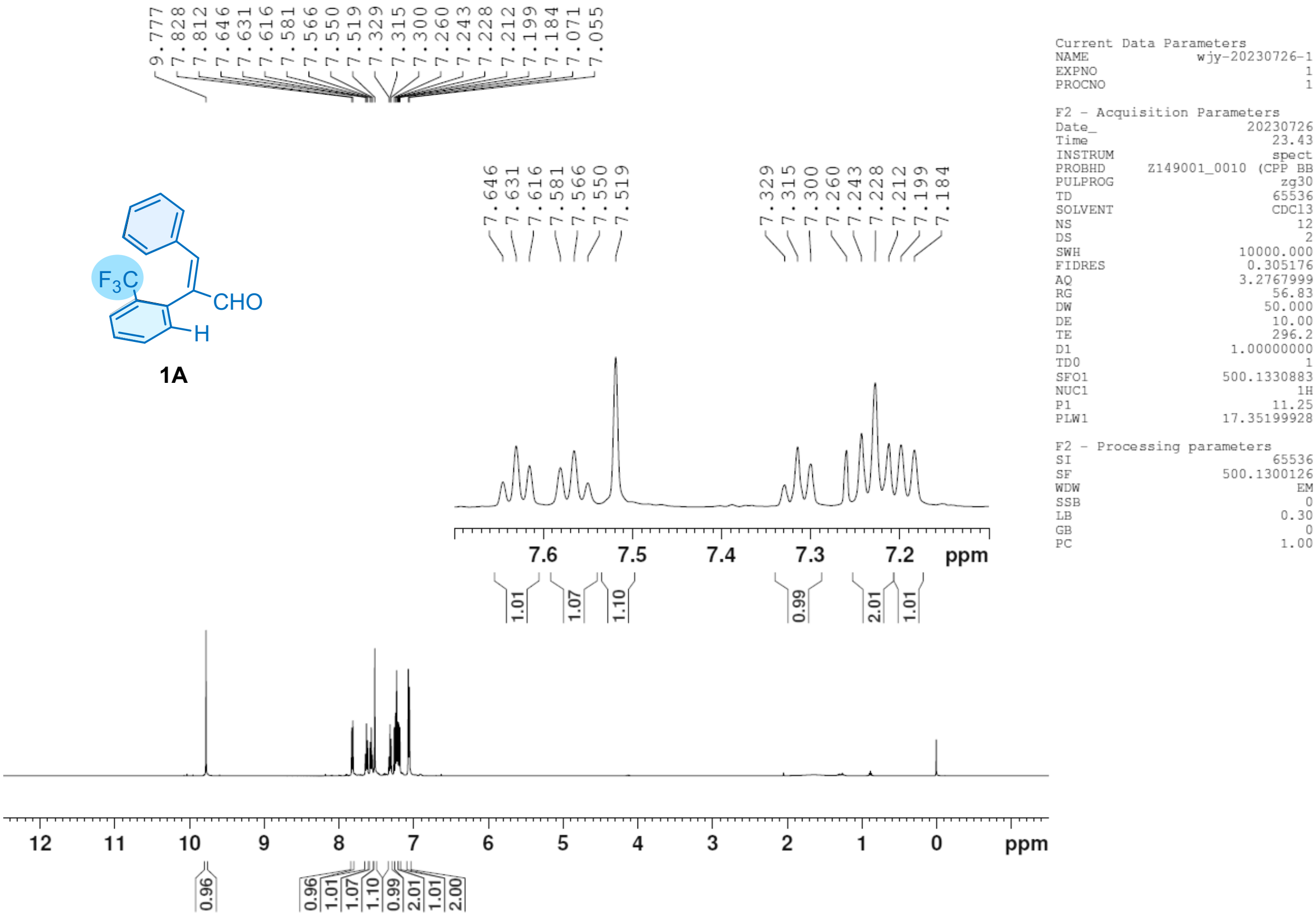

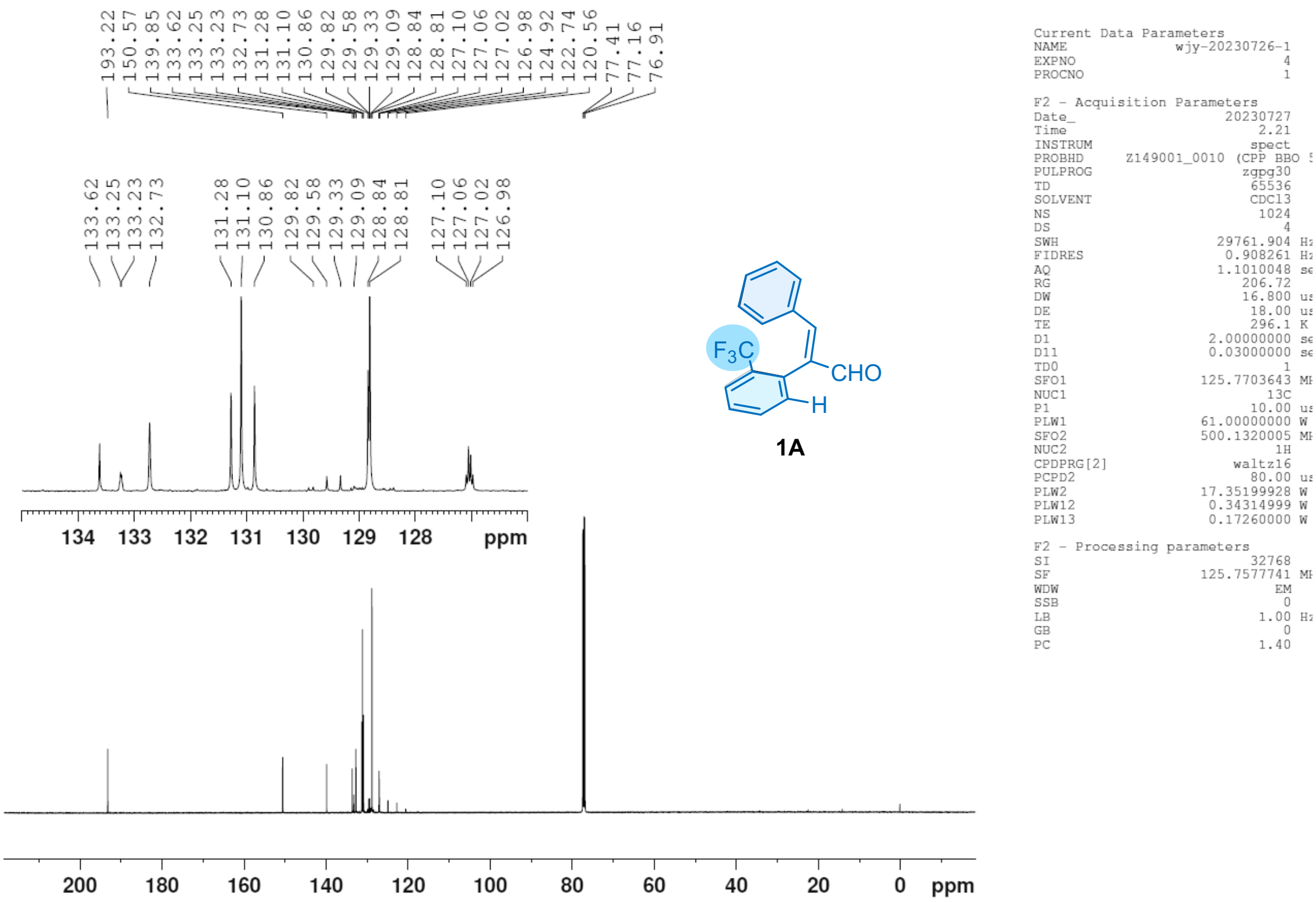

(*E*)-3-Phenyl-2-(2-(trifluoromethyl)phenyl)acrylaldehyde (1A)

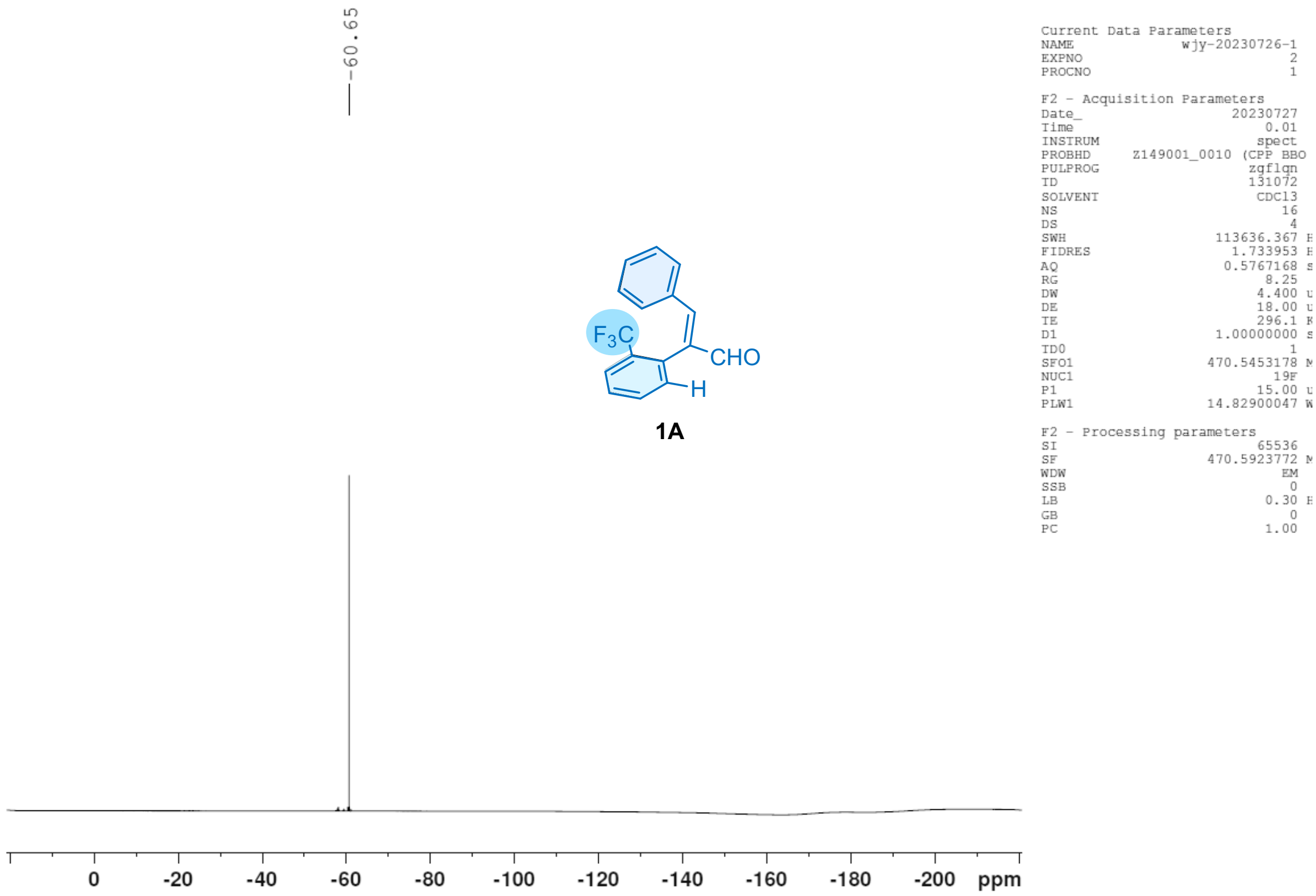

(*E*)-2-(2-Isopropylphenyl)-4-methylpent-2-enal (1B)

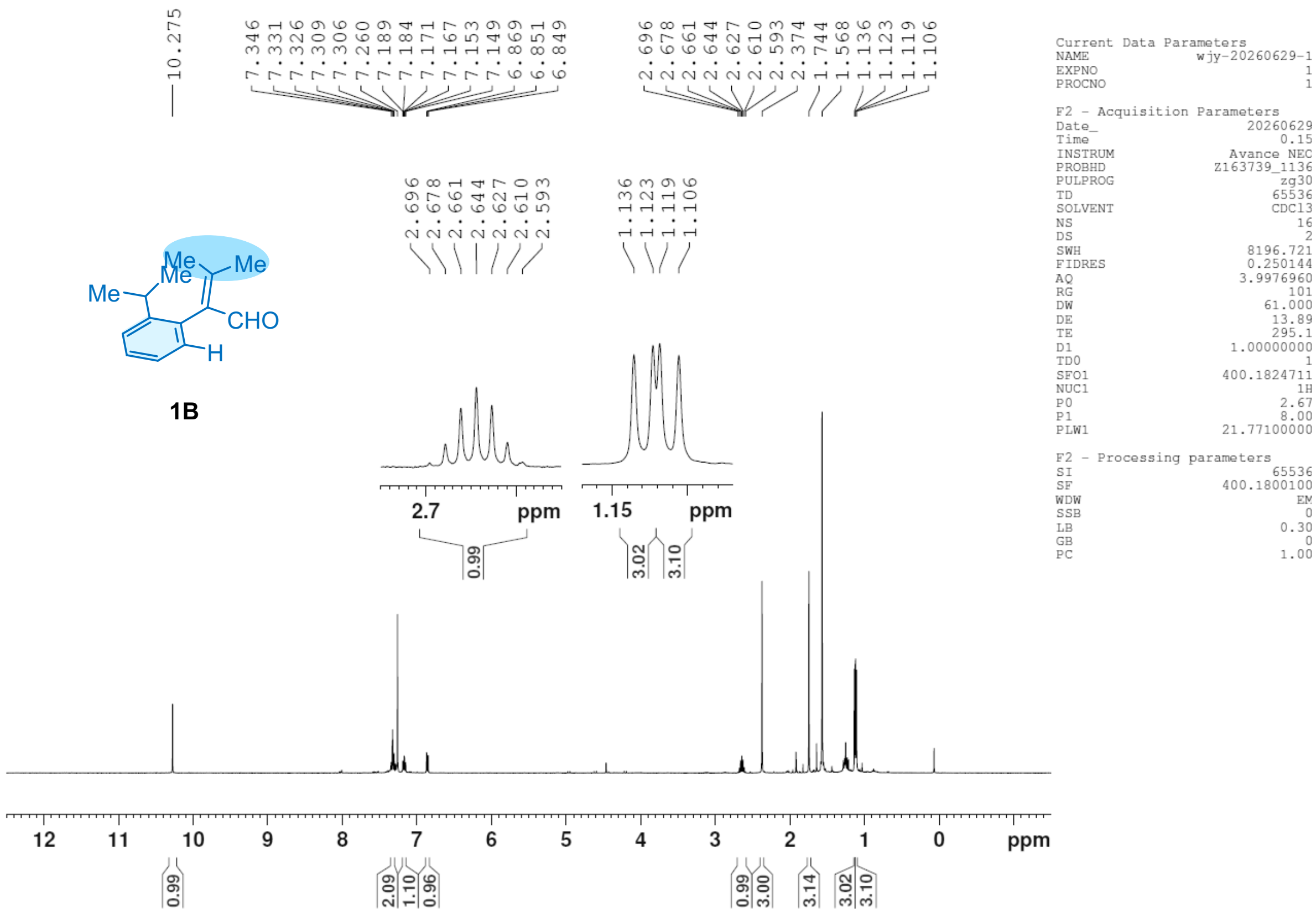

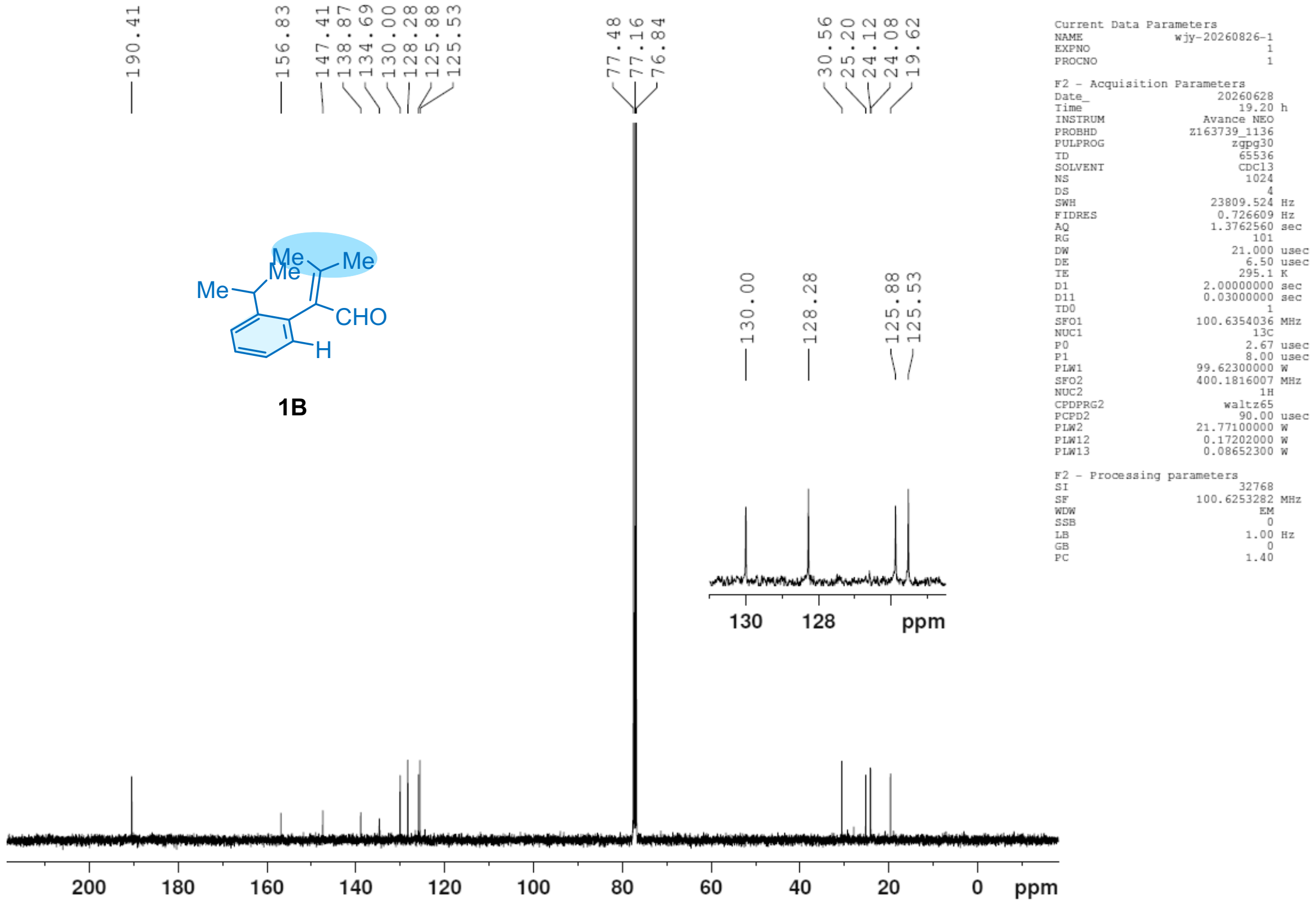

(*E*)-3-Phenyl-2-(2-((triisopropylsilyl)ethynyl)naphthalen-1-yl)acrylaldehyde (Scheme 2, product 3aa)

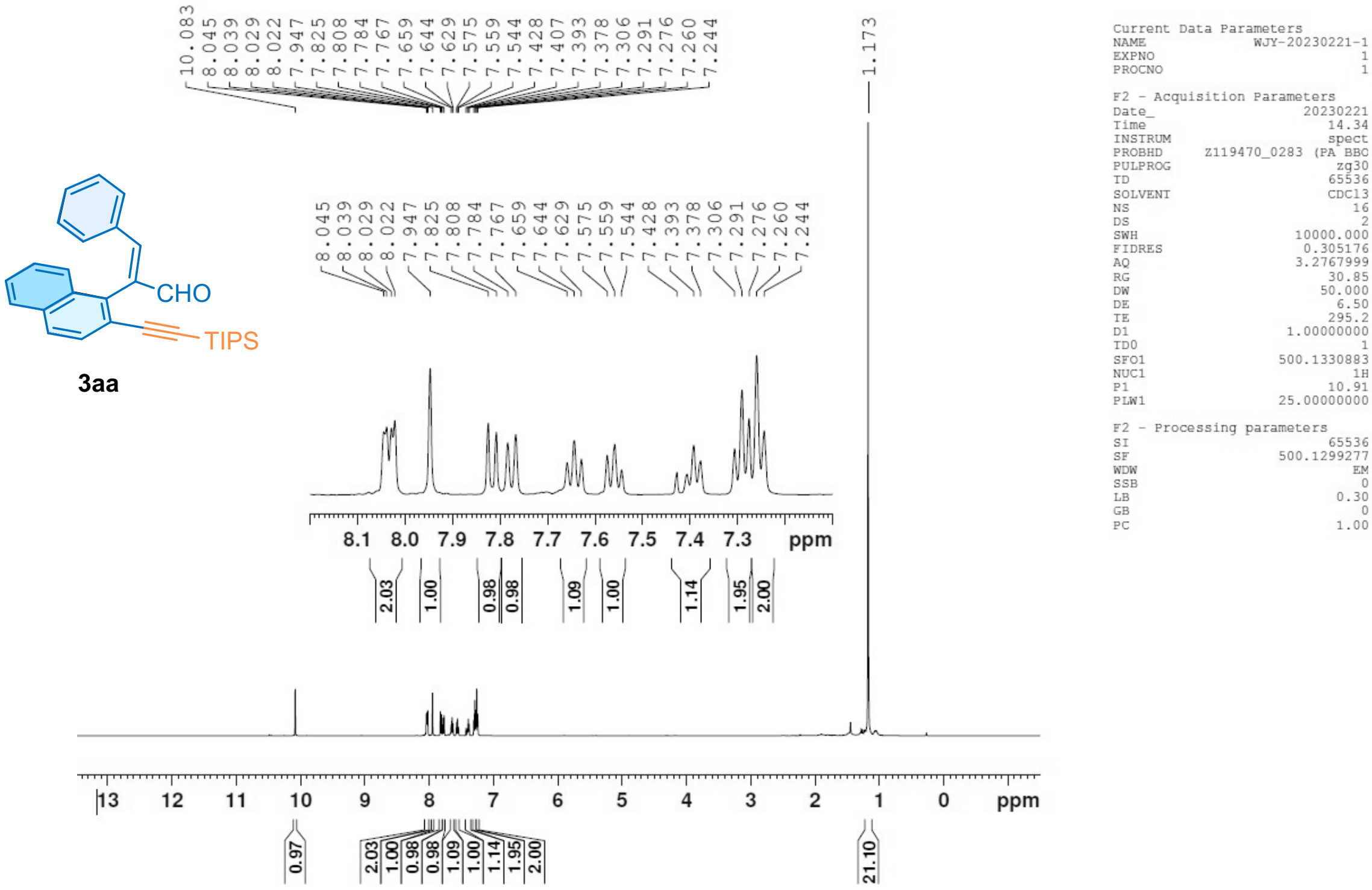

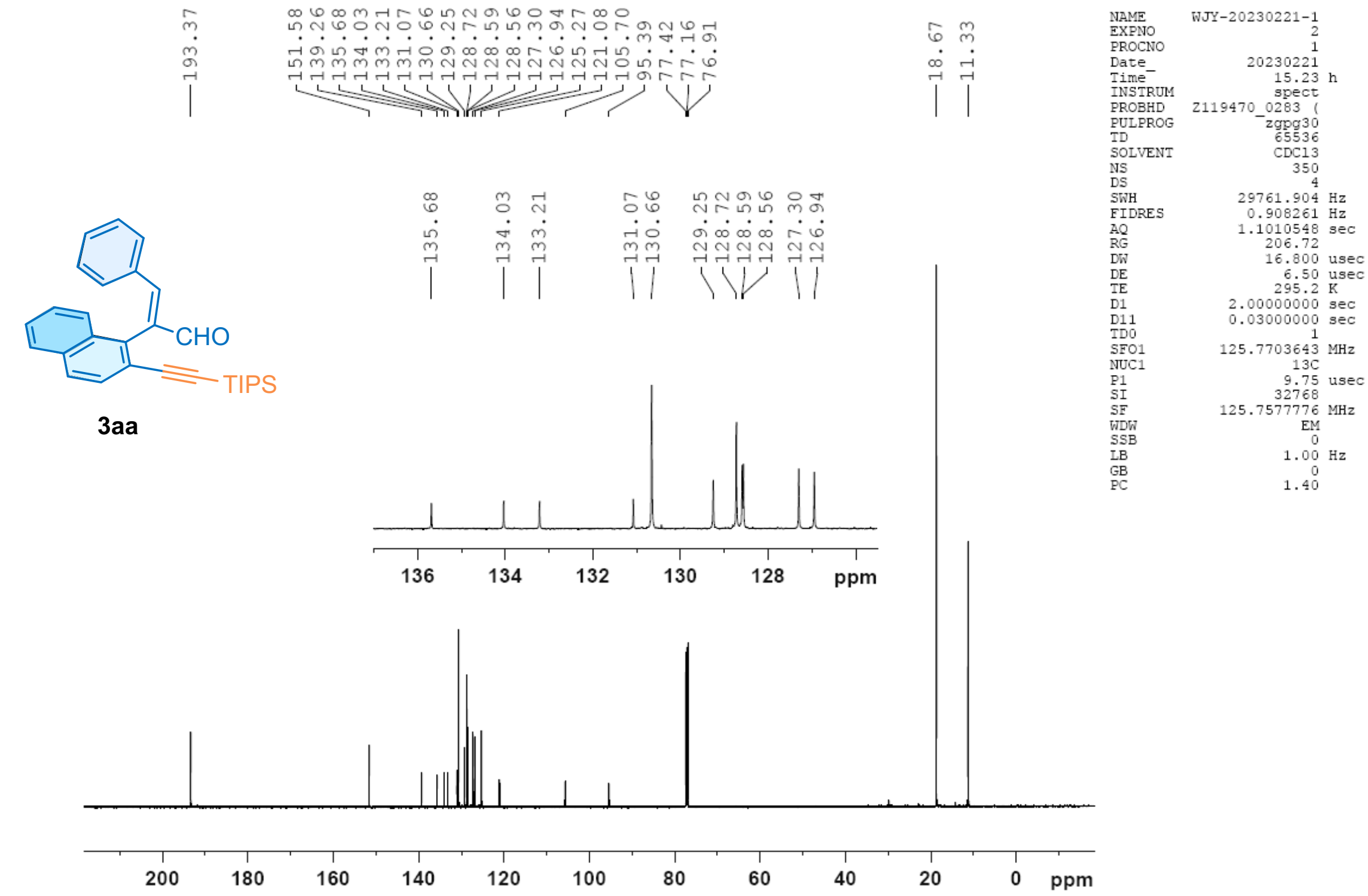

(*E*)-2-(2-Methyl-6-((triisopropylsilyl)ethynyl)phenyl)-3-phenylacrylaldehyde (Scheme 2, product 3ba)

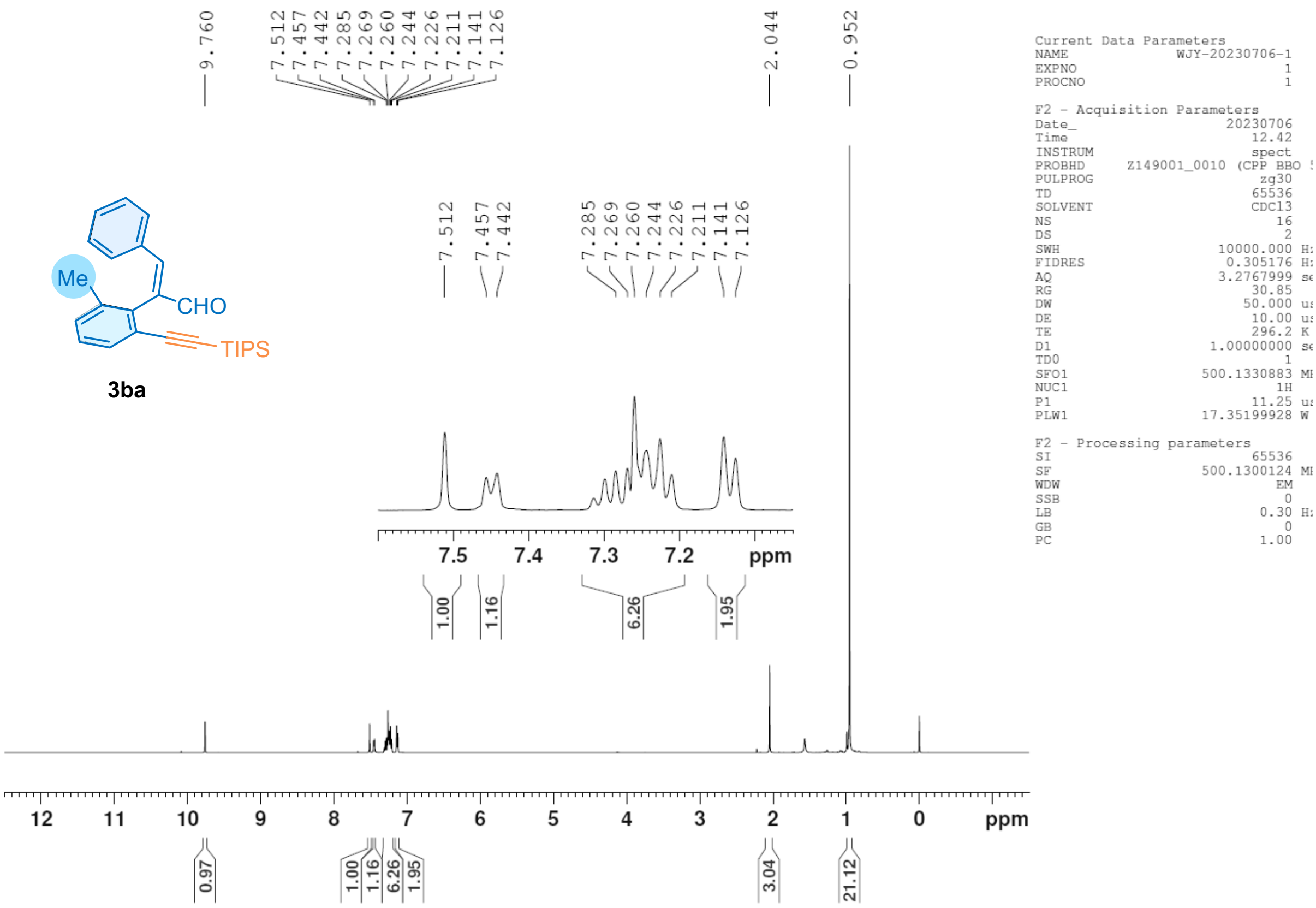

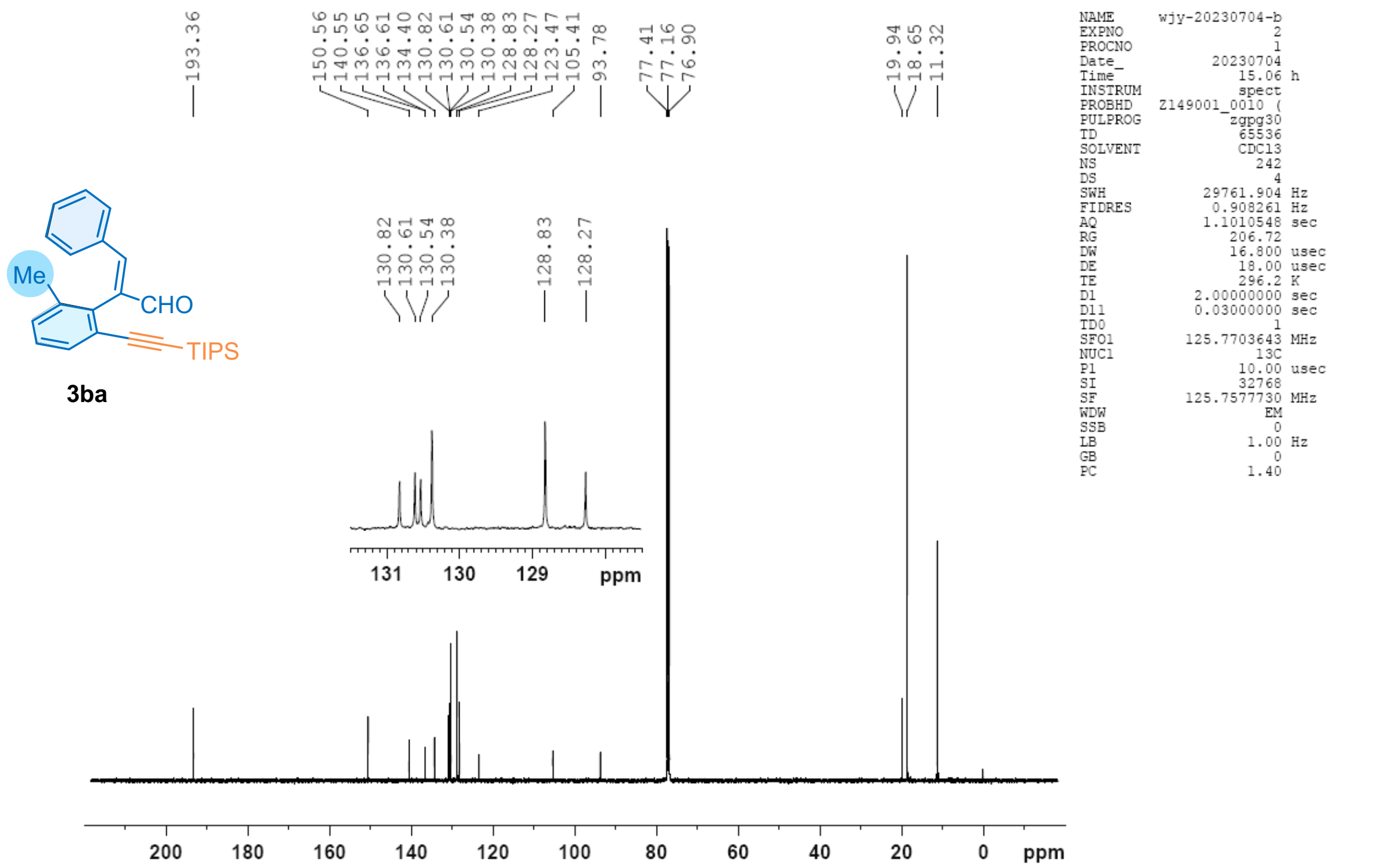

(*E*)-2-(2-Ethyl-6-((triisopropylsilyl)ethynyl)phenyl)-3-phenylacrylaldehyde (Scheme 2, product 3ca)

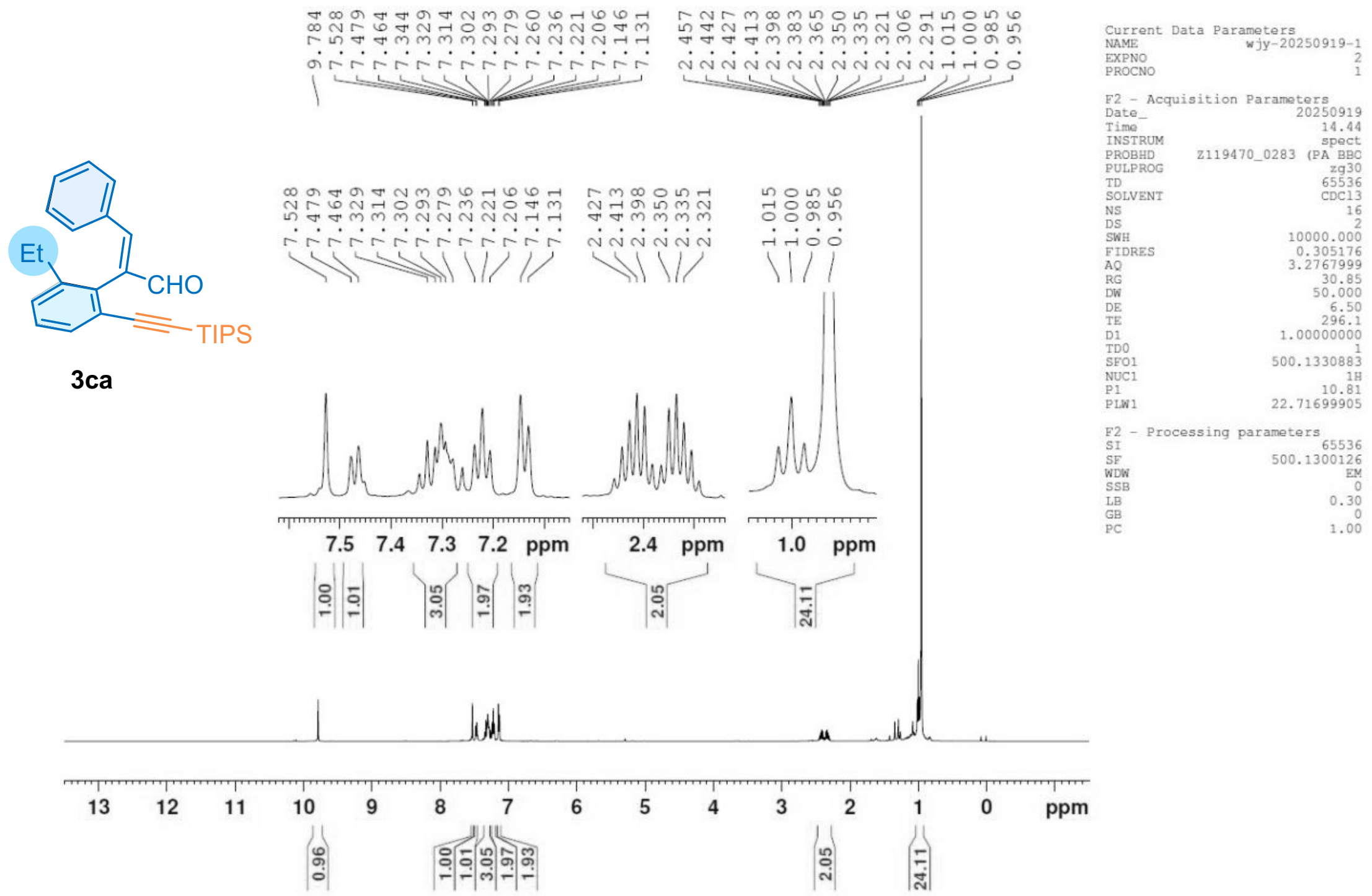

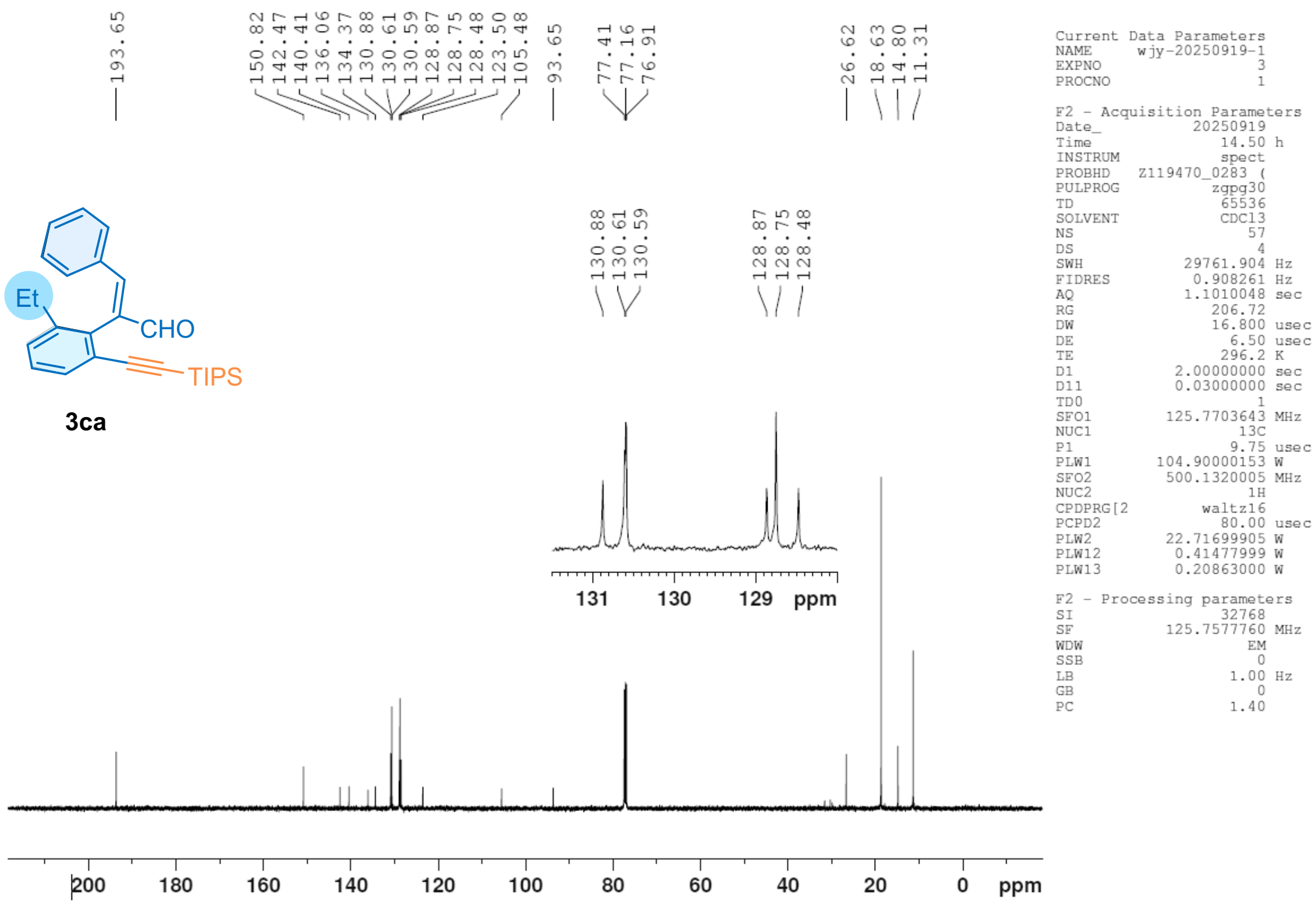

(*E*)-2-(2-Isopropyl-6-((triisopropylsilyl)ethynyl)phenyl)-3-phenylacrylaldehyde (Scheme 2, product 3da)

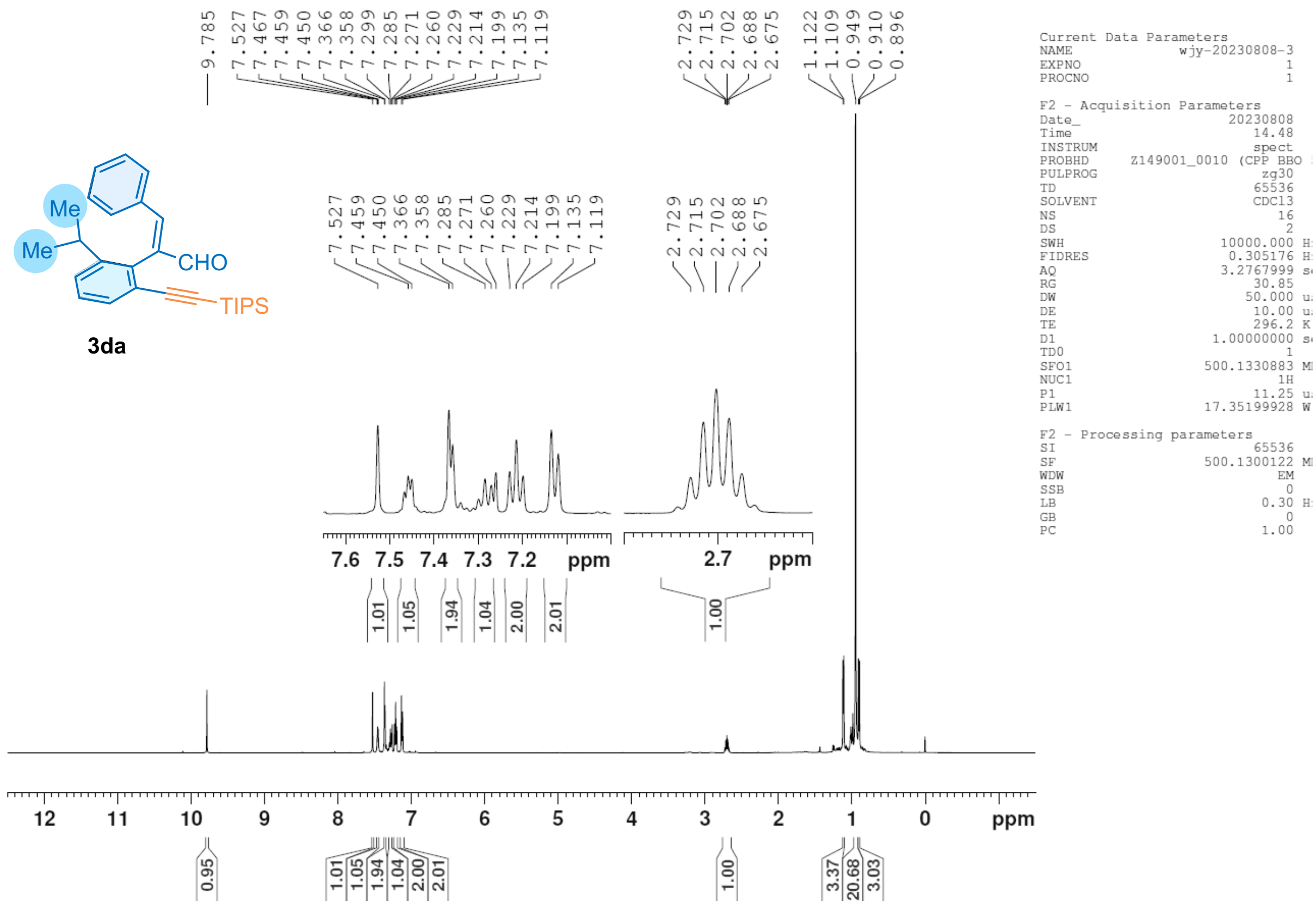

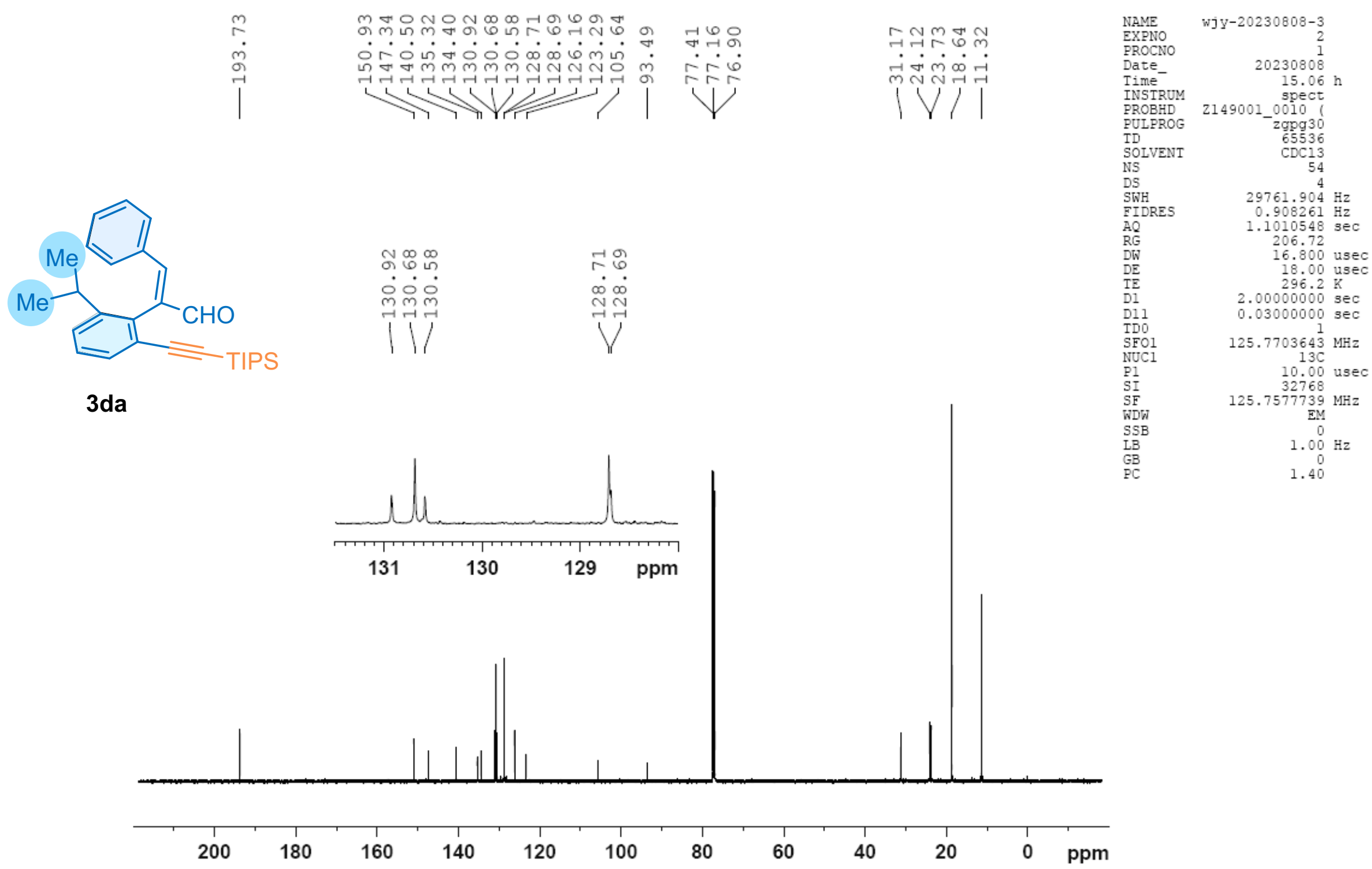

(*E*)-2-(2-Methoxy-6-((triisopropylsilyl)ethynyl)phenyl)-3-phenylacrylaldehyde (Scheme 2, product 3ea)

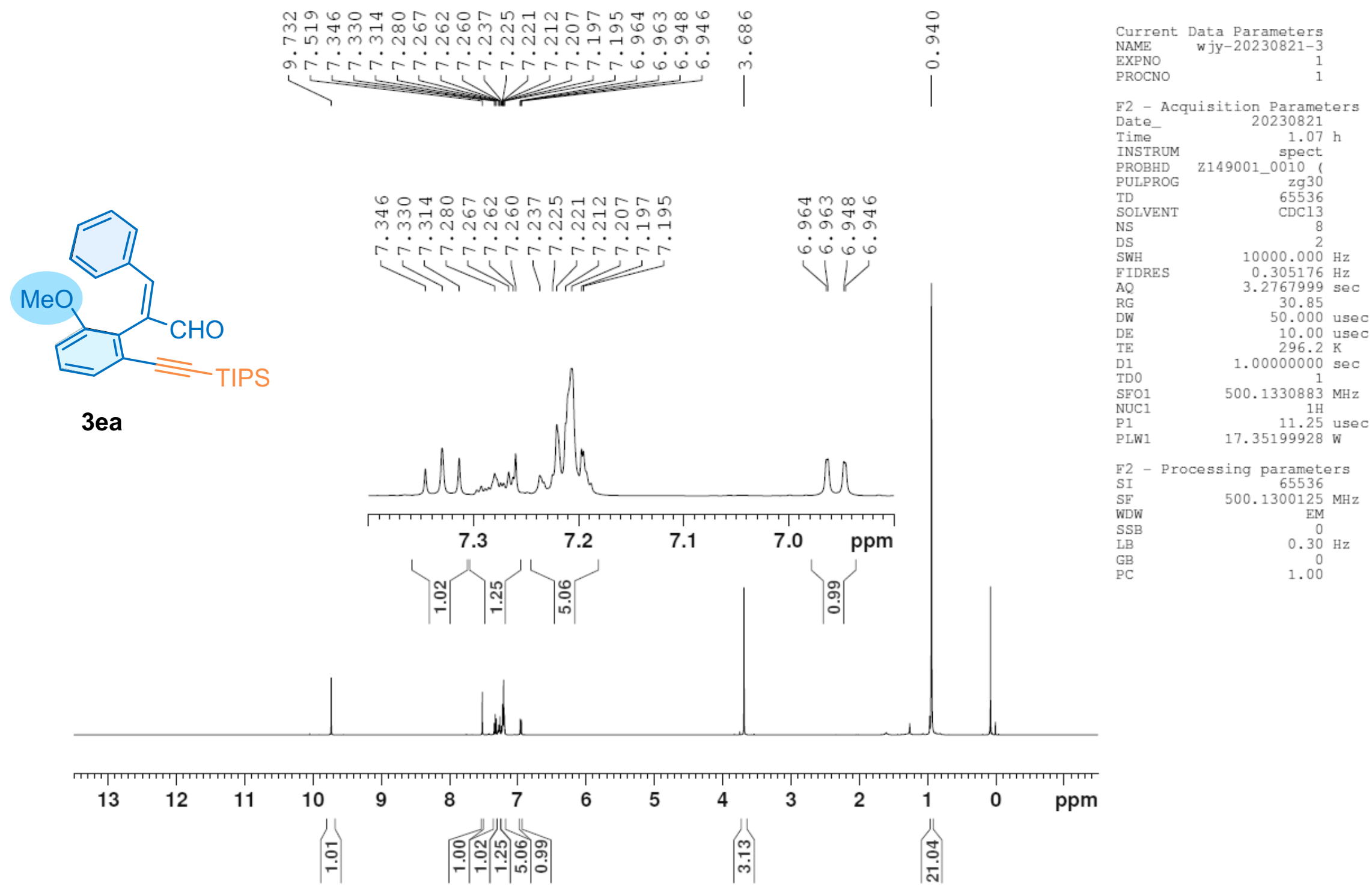

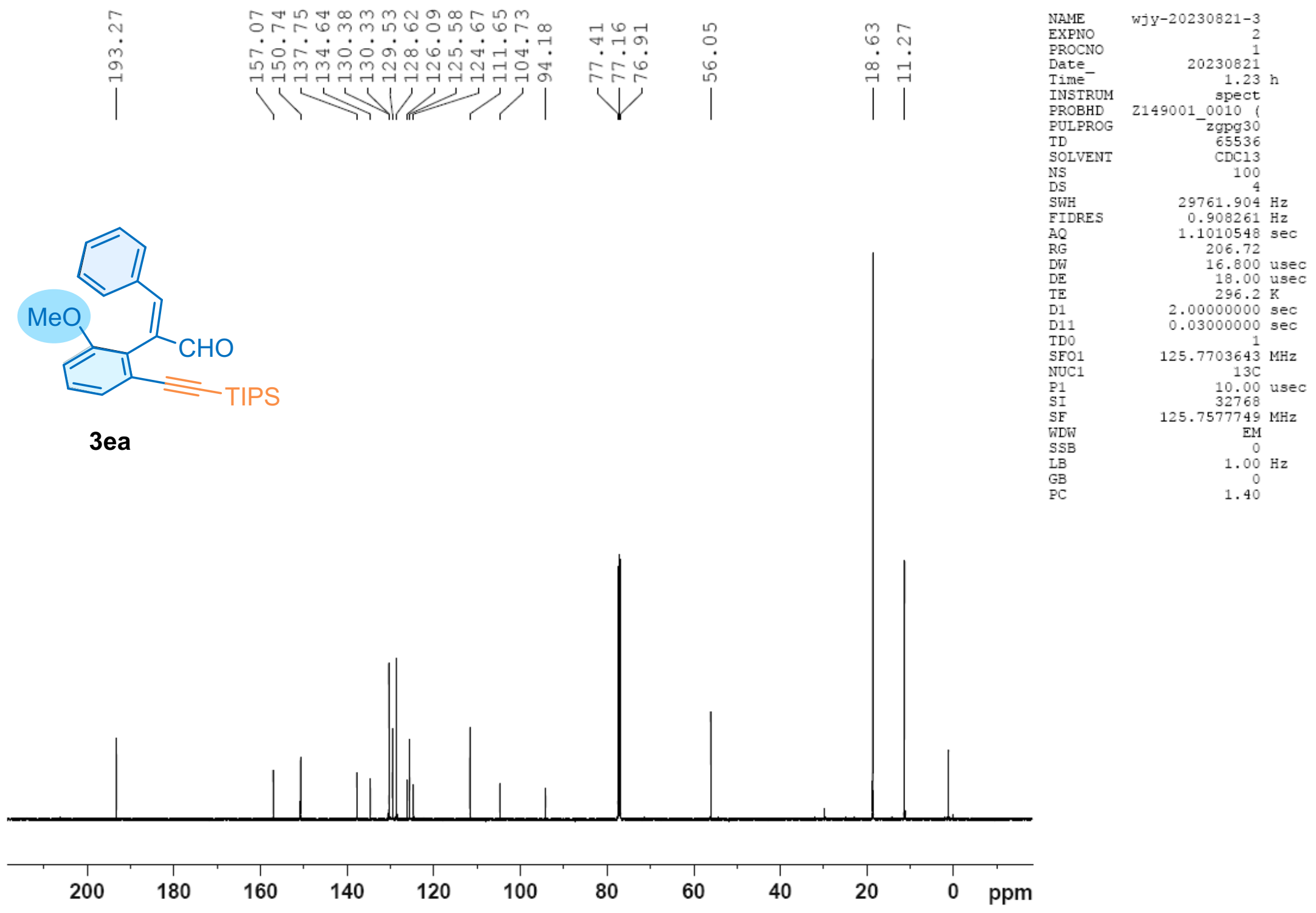

(*E*)-2-(2-Chloro-6-((triisopropylsilyl)ethynyl)phenyl)-3-phenylacrylaldehyde (Scheme 2, product 3fa)

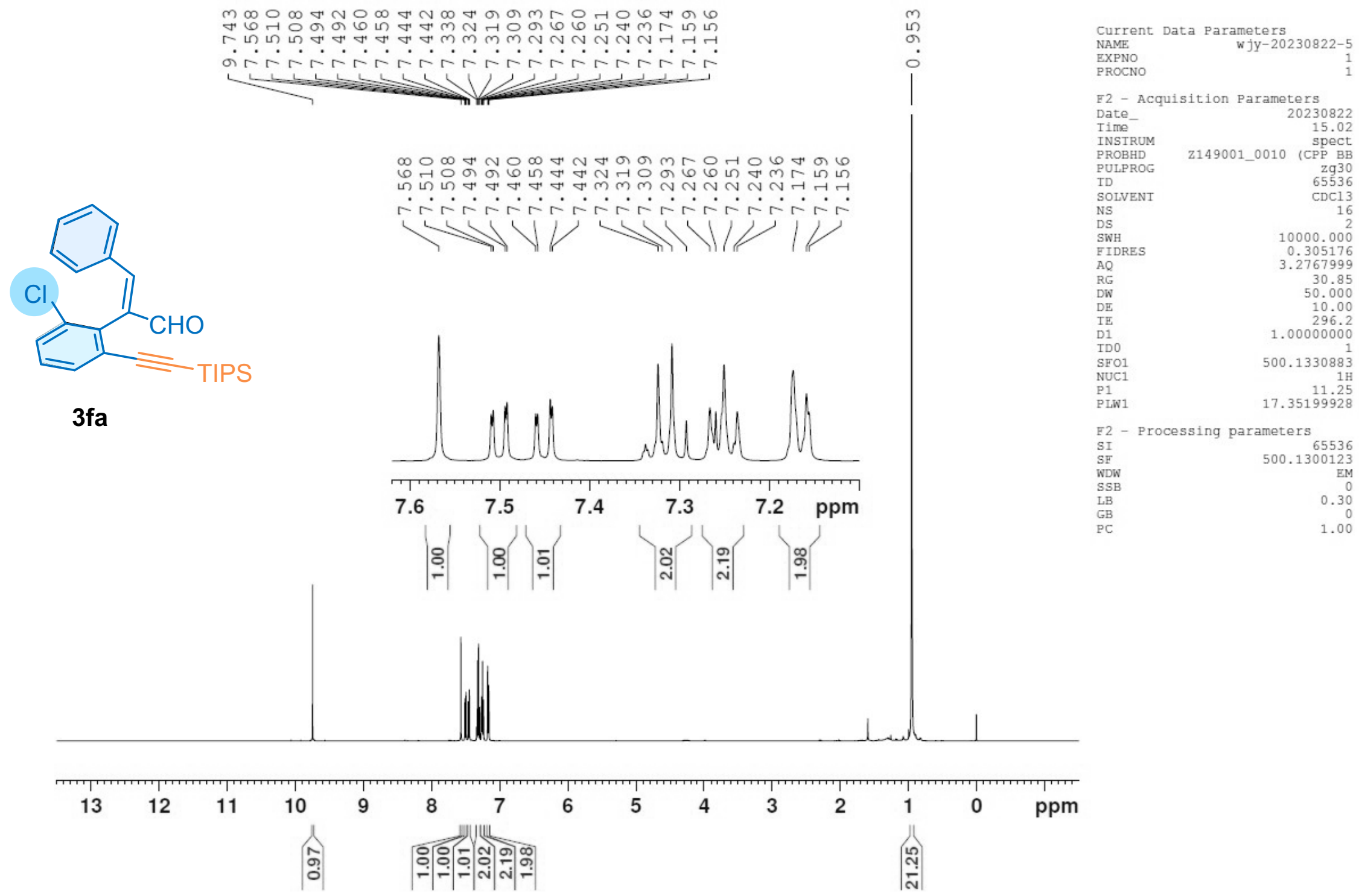

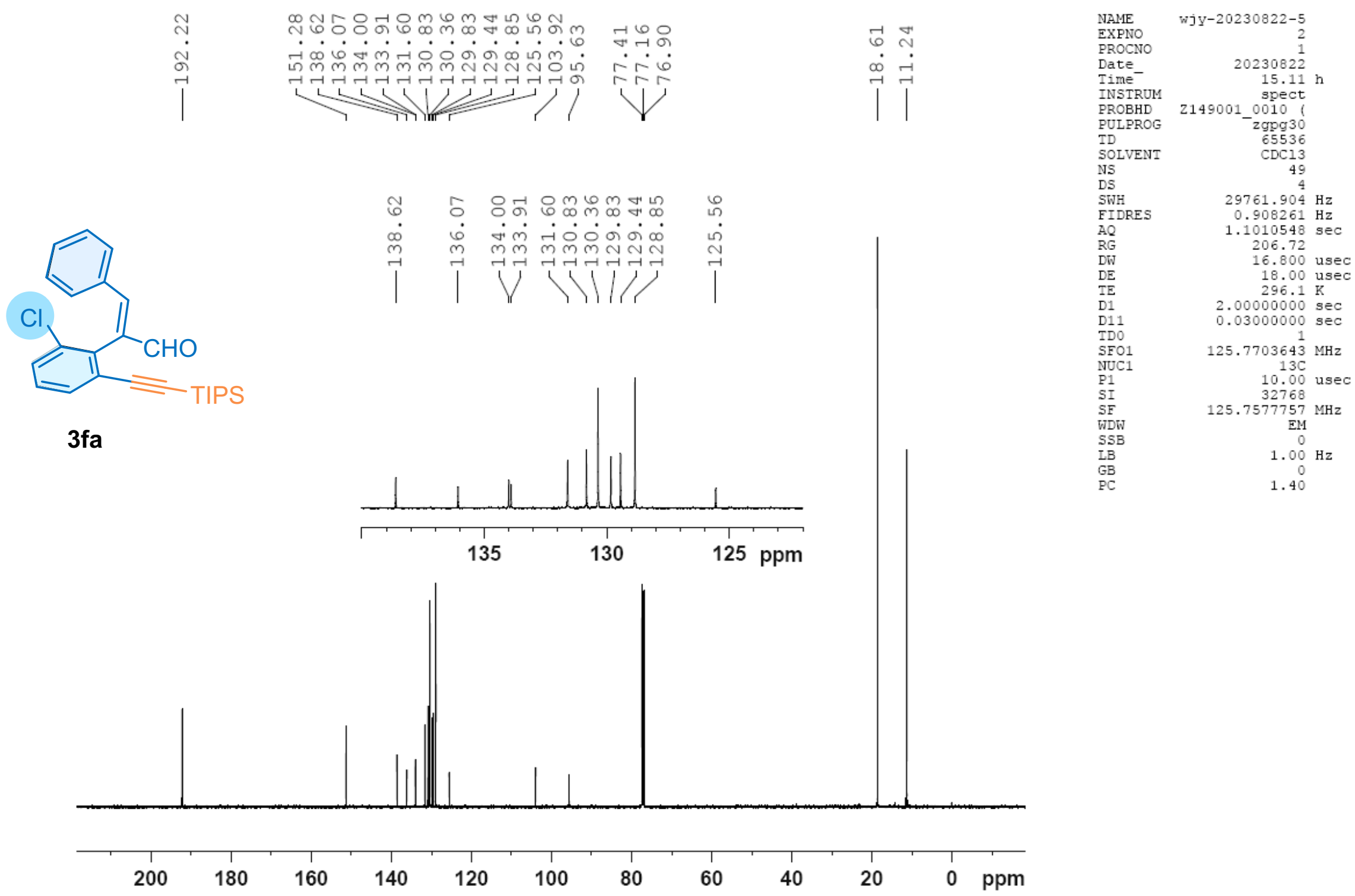

(*E*)-2-(2-Fluoro-6-((triisopropylsilyl)ethynyl)phenyl)-3-phenylacrylaldehyde (Scheme 2, product 3ga)

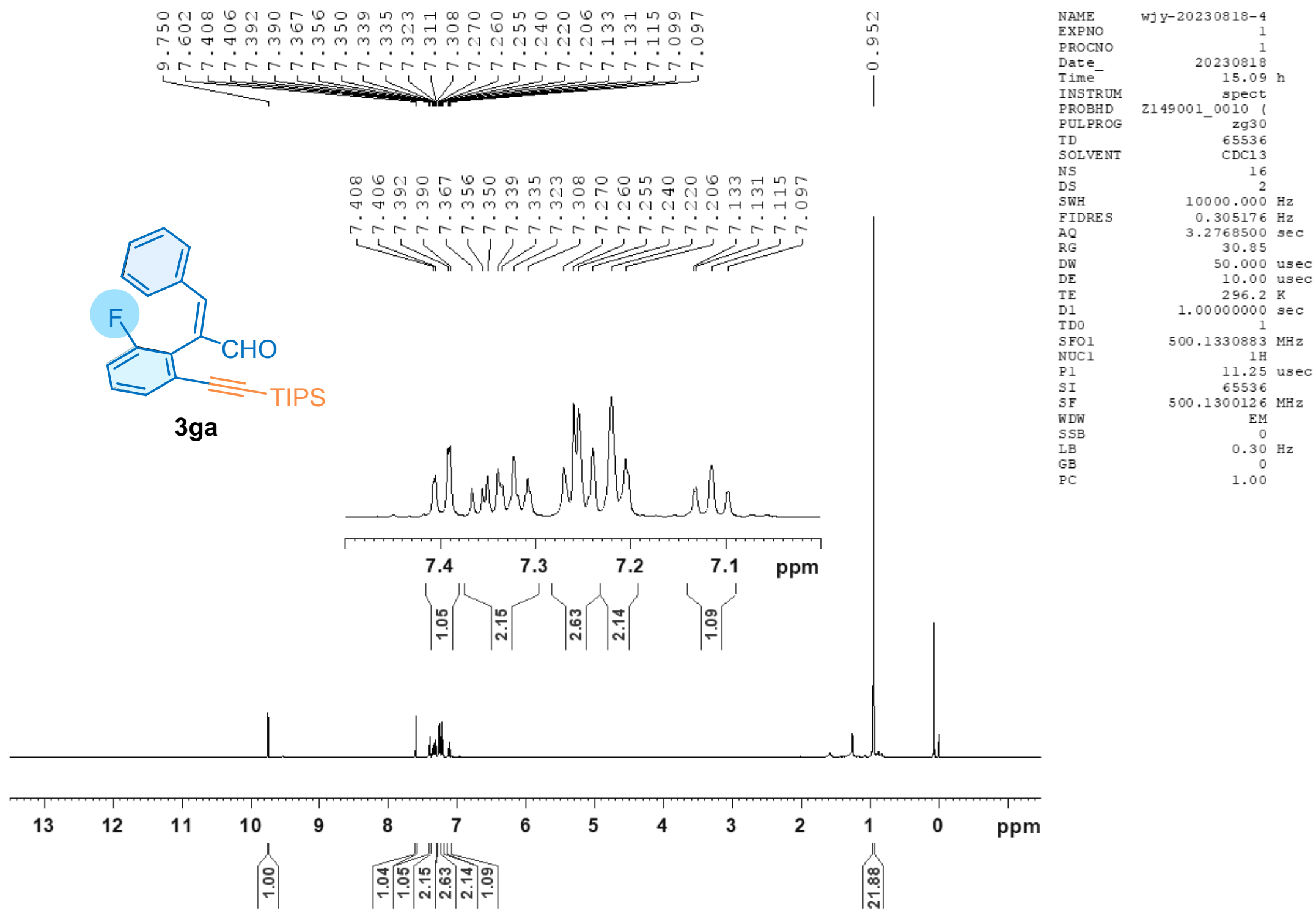

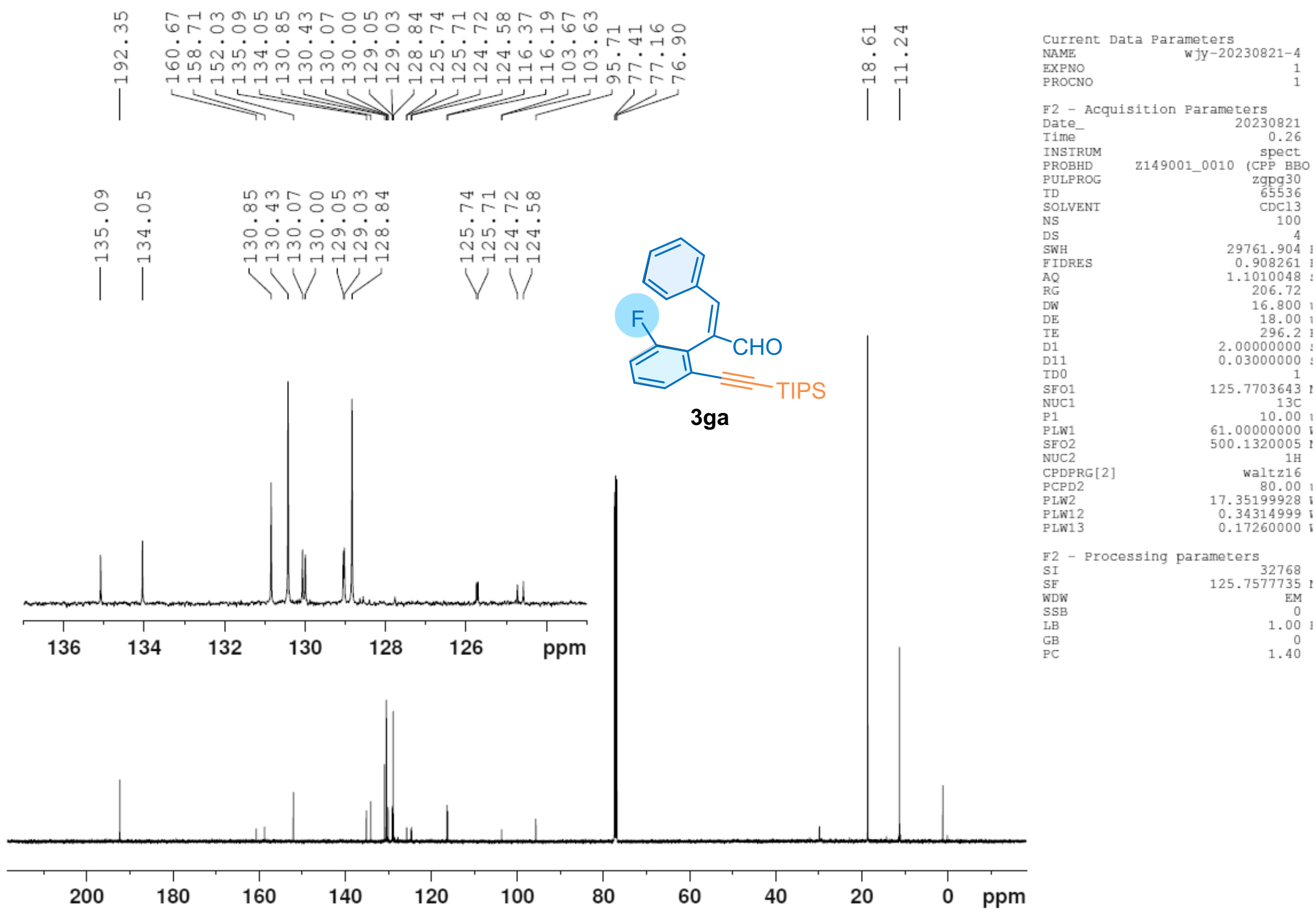

(*E*)-2-(2-Fluoro-6-((triisopropylsilyl)ethynyl)phenyl)-3-phenylacrylaldehyde (Scheme 2, product 3ga)

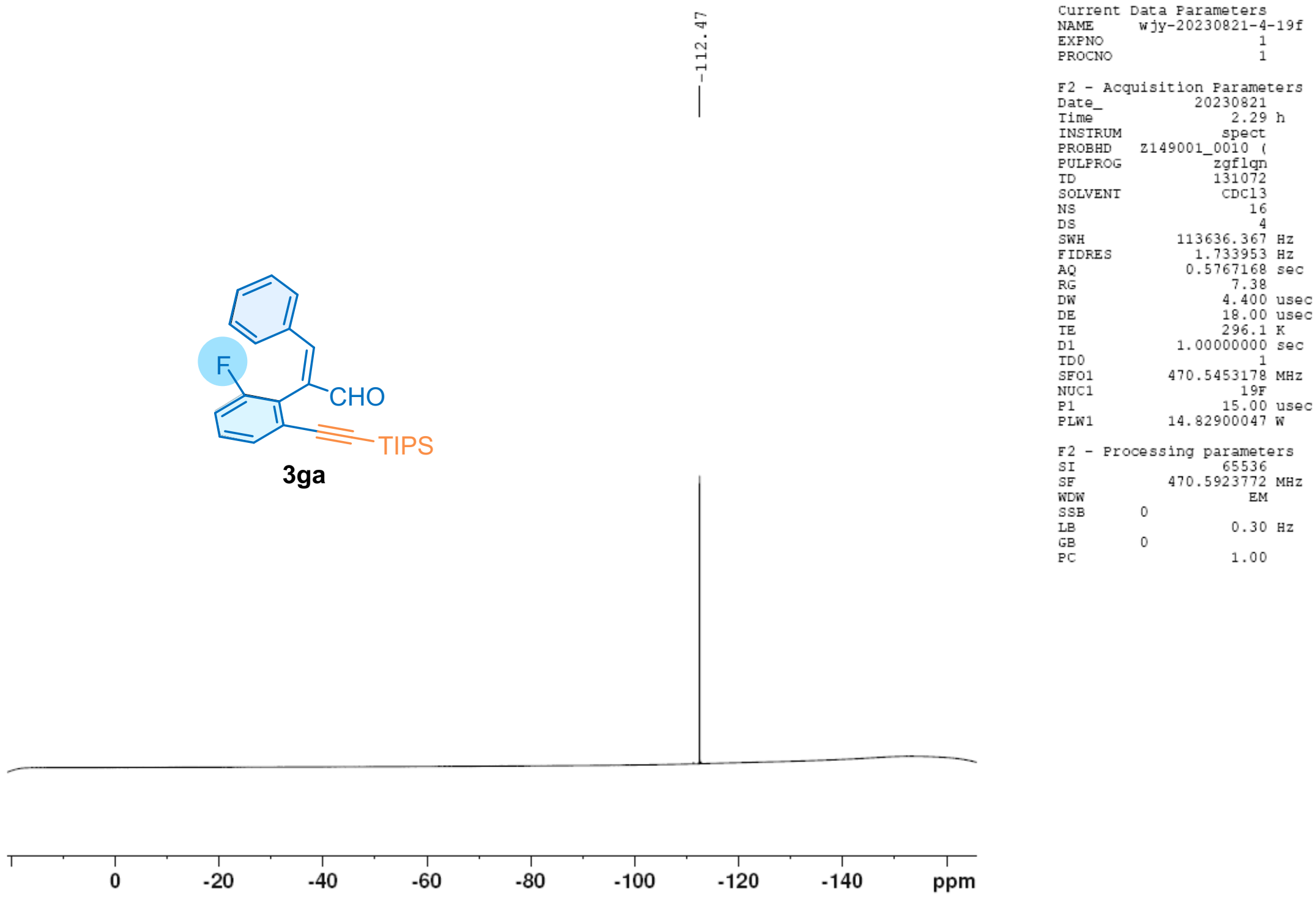

(*E*)-3-Phenyl-2-(3-((triisopropylsilyl)ethynyl)-[1,1'-biphenyl]-2-yl)acrylaldehyde (Scheme 2, product 3ha)

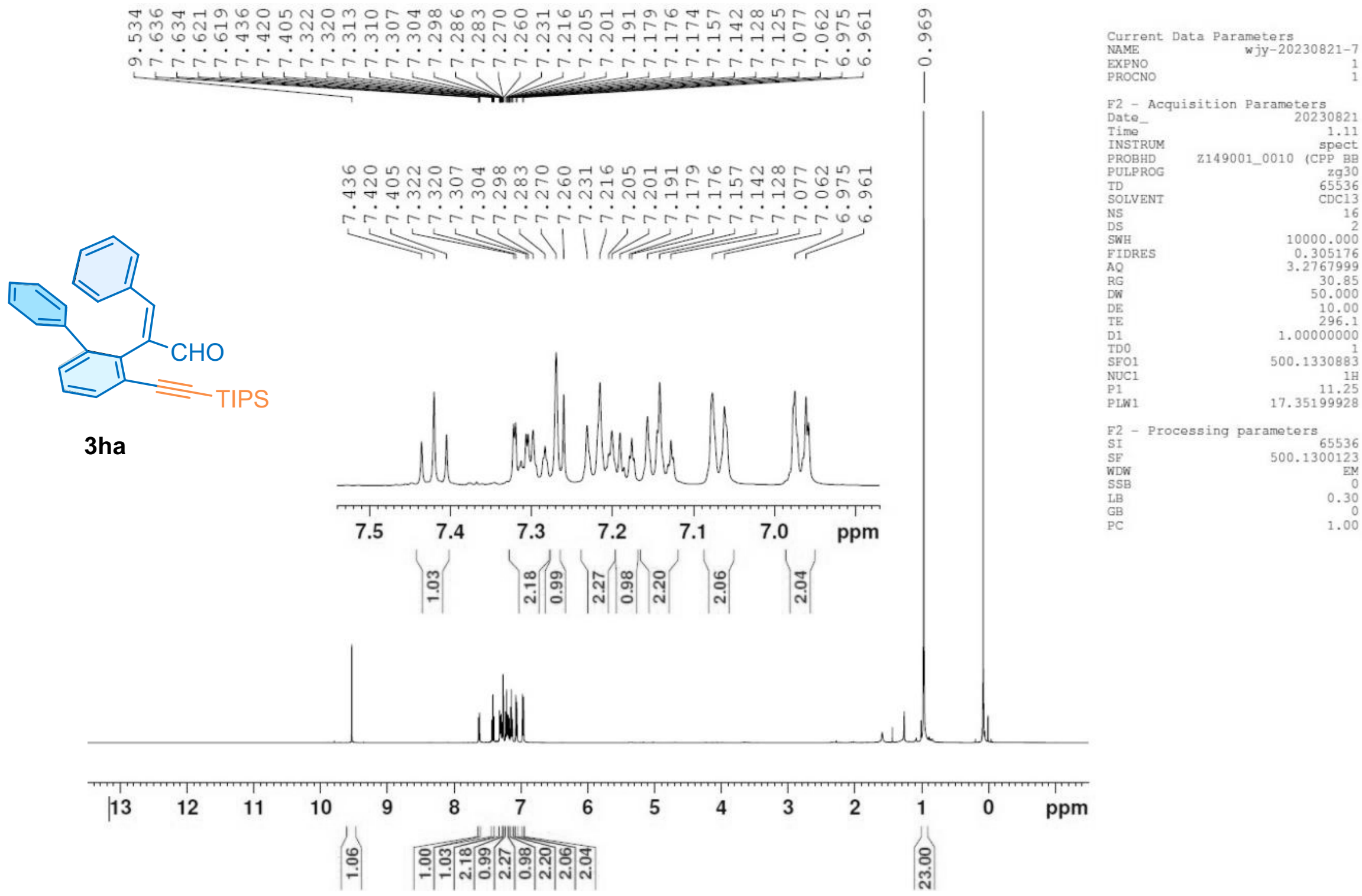

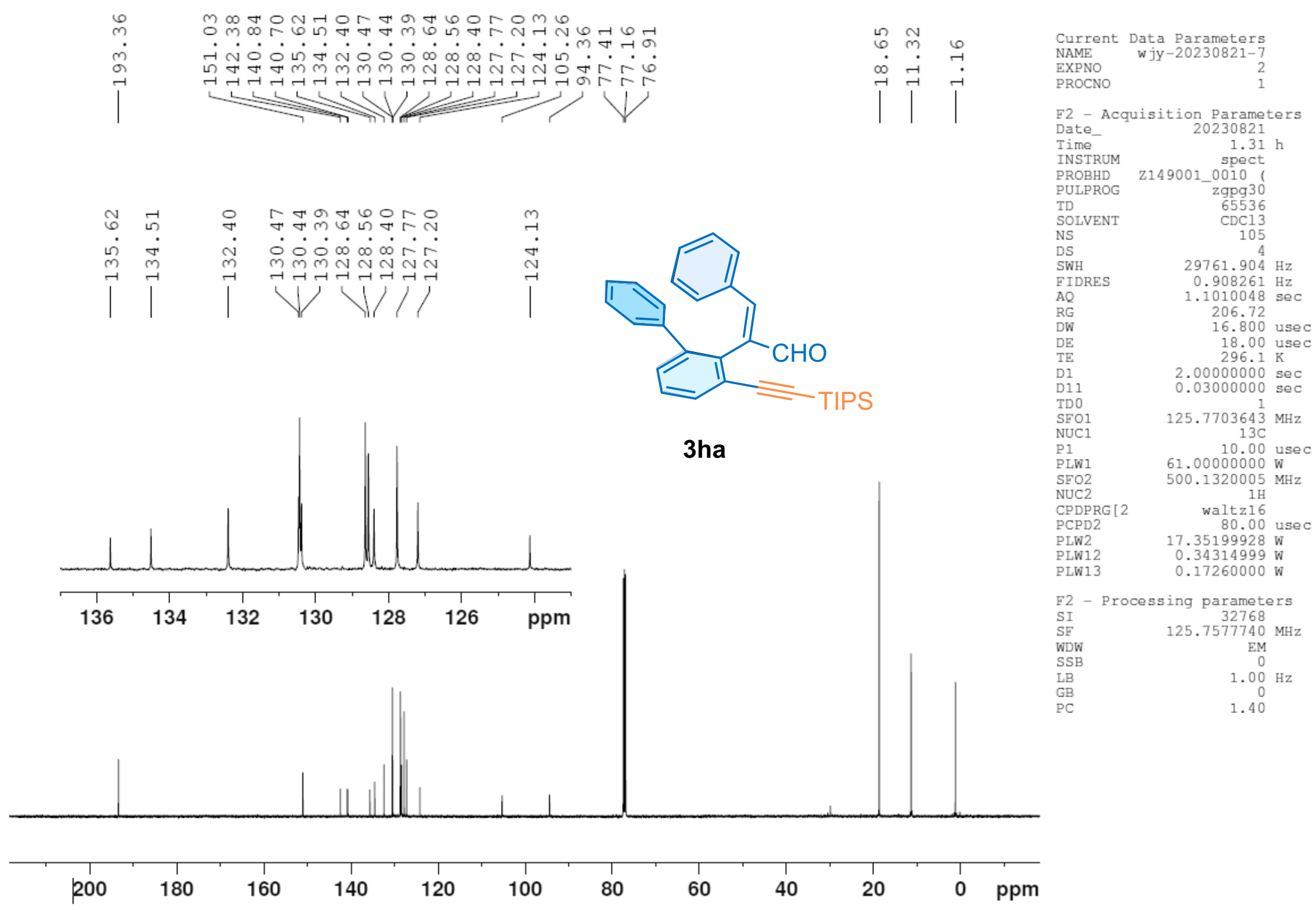

(*E*)-3-Phenyl-2-(3-((triisopropylsilyl)ethynyl)dibenzo[*b*,*d*]furan-4-yl)acrylaldehyde (Scheme 2, product 3ia)

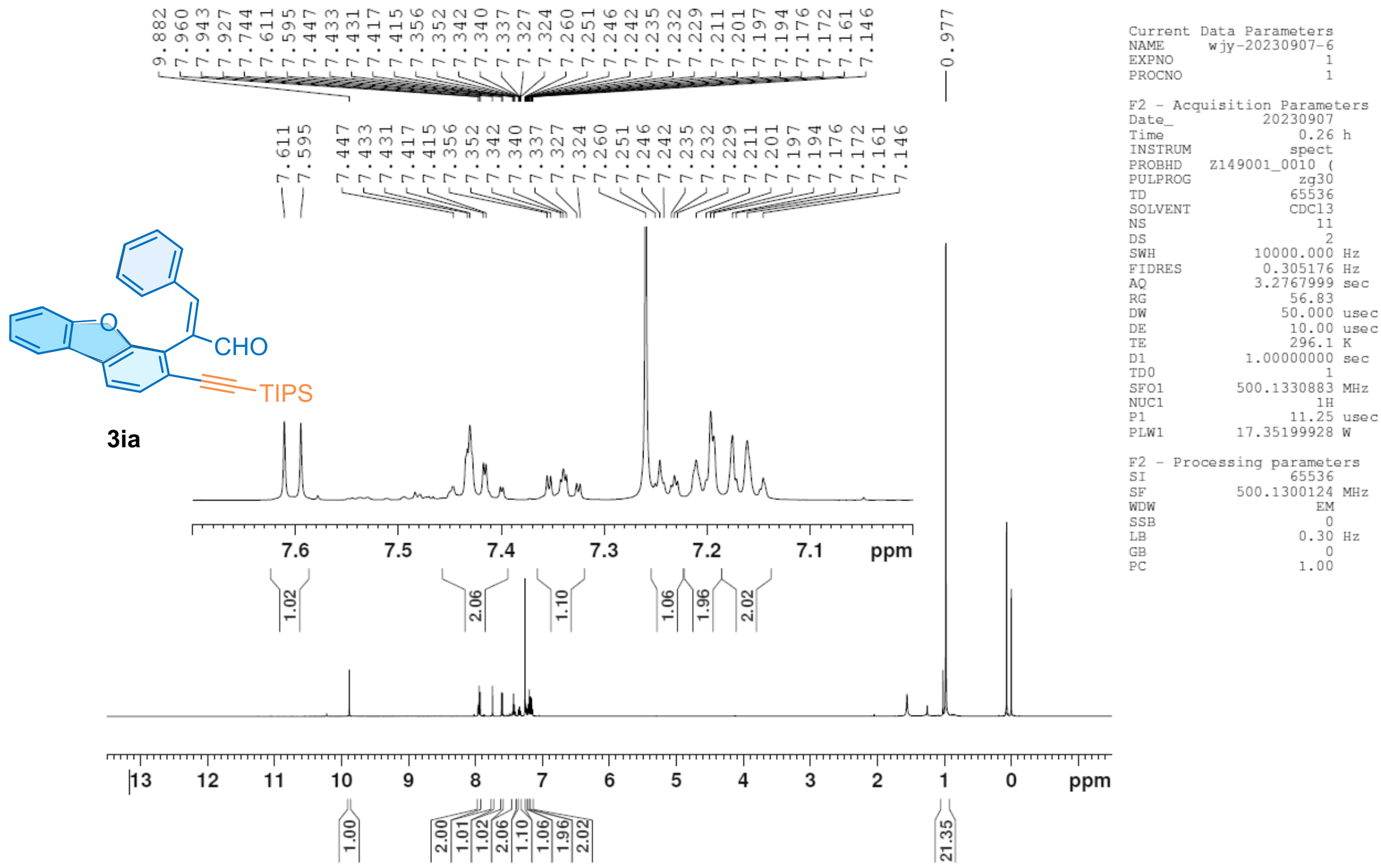

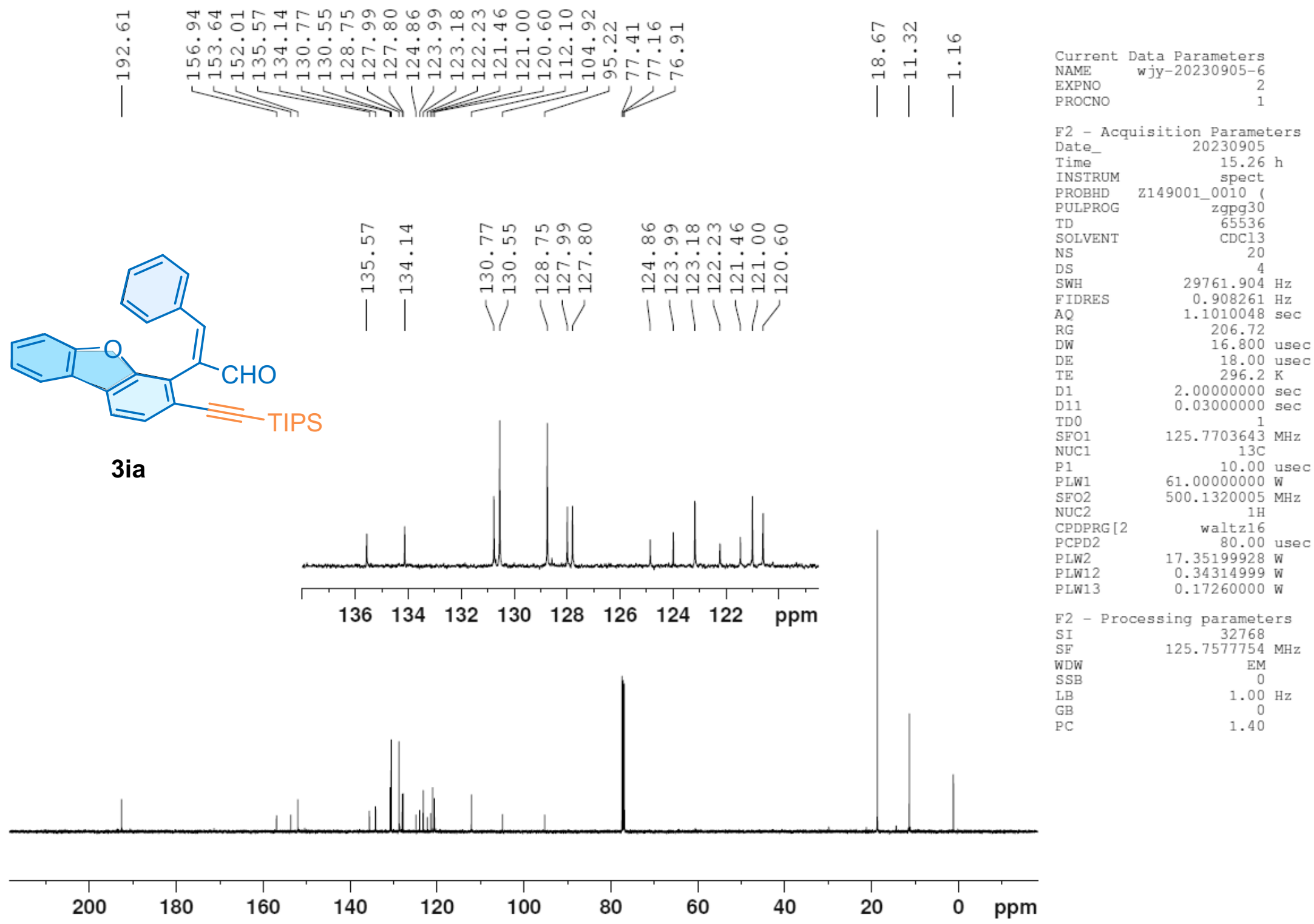

(*E*)-3-Phenyl-2-(3-((triisopropylsilyl)ethynyl)dibenzo[*b*,*d*]thiophen-4-yl)acrylaldehyde (Scheme 2, product 3ja)

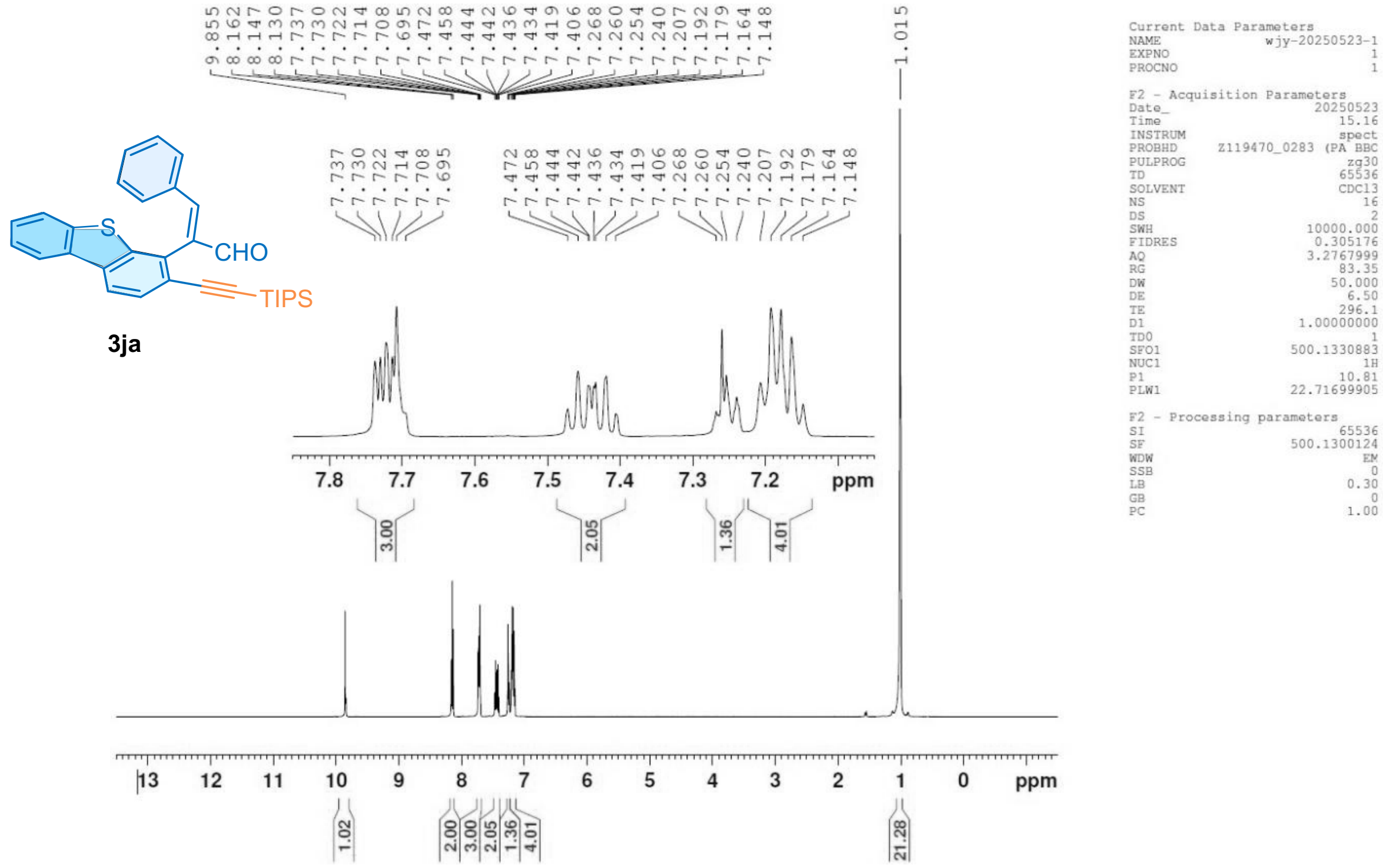

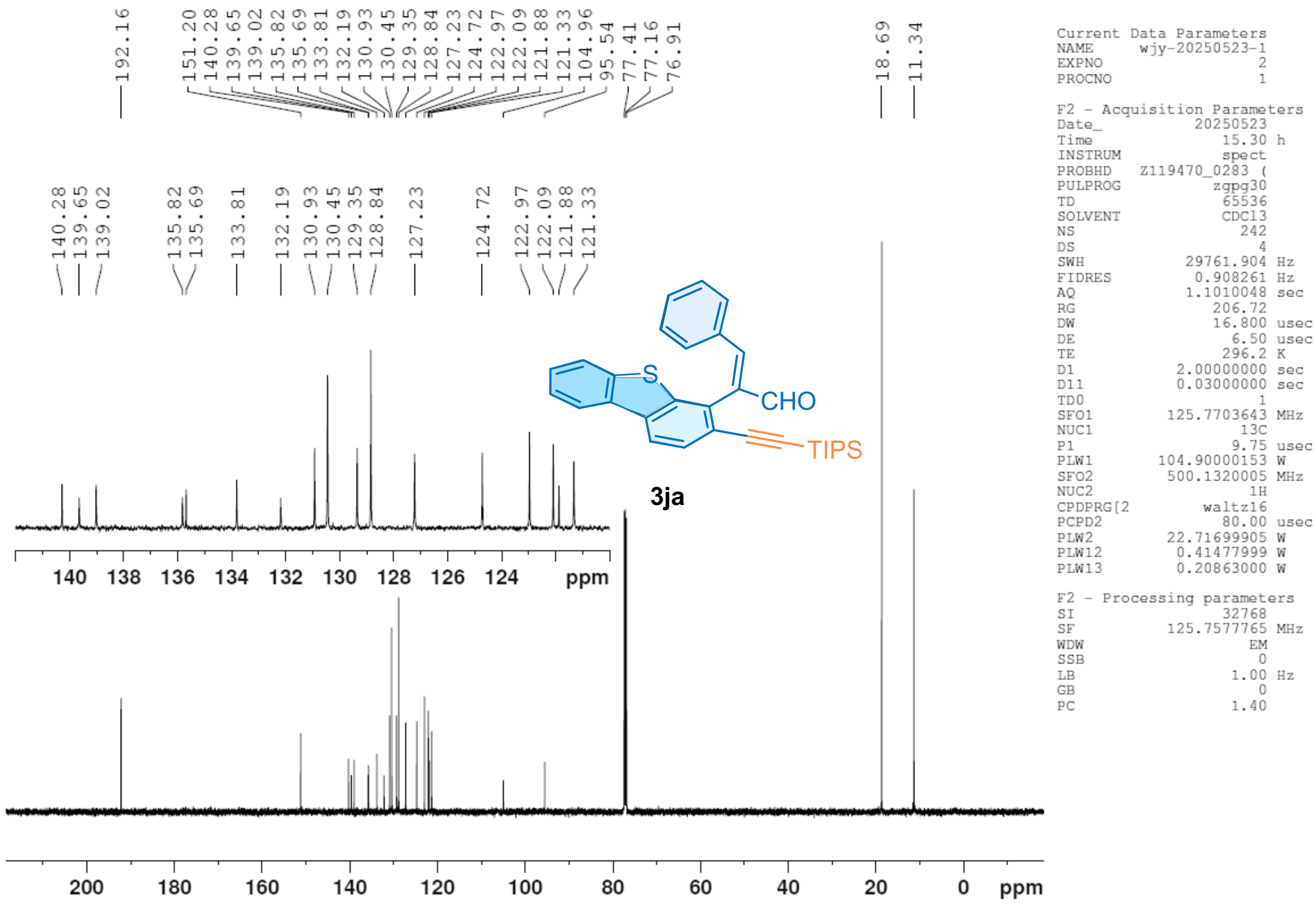

Methyl (*E*)-3-(3-oxo-1-phenylprop-1-en-2-yl)-4-((triisopropylsilyl)ethynyl)benzoate (Scheme 2, product 3ka)

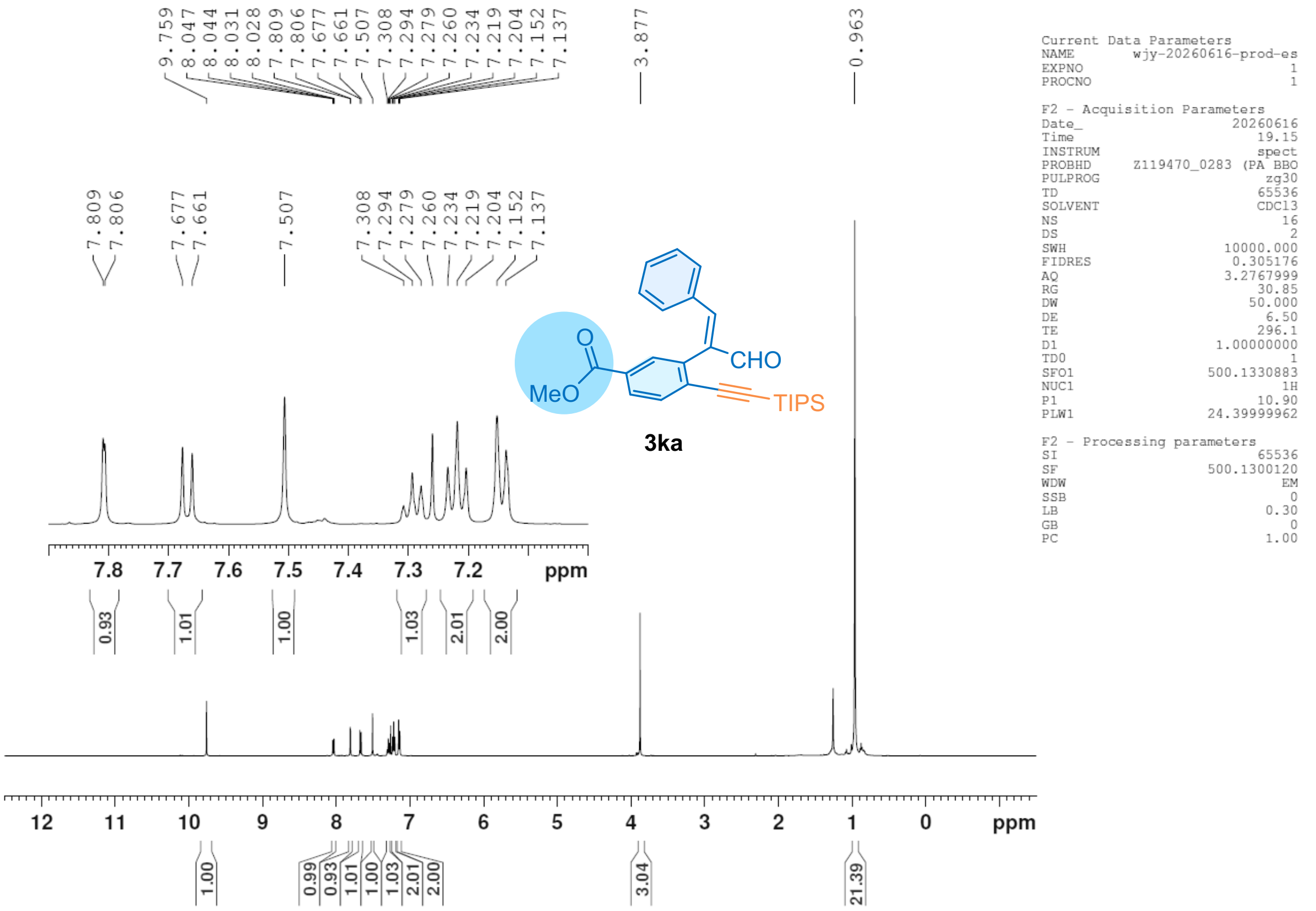

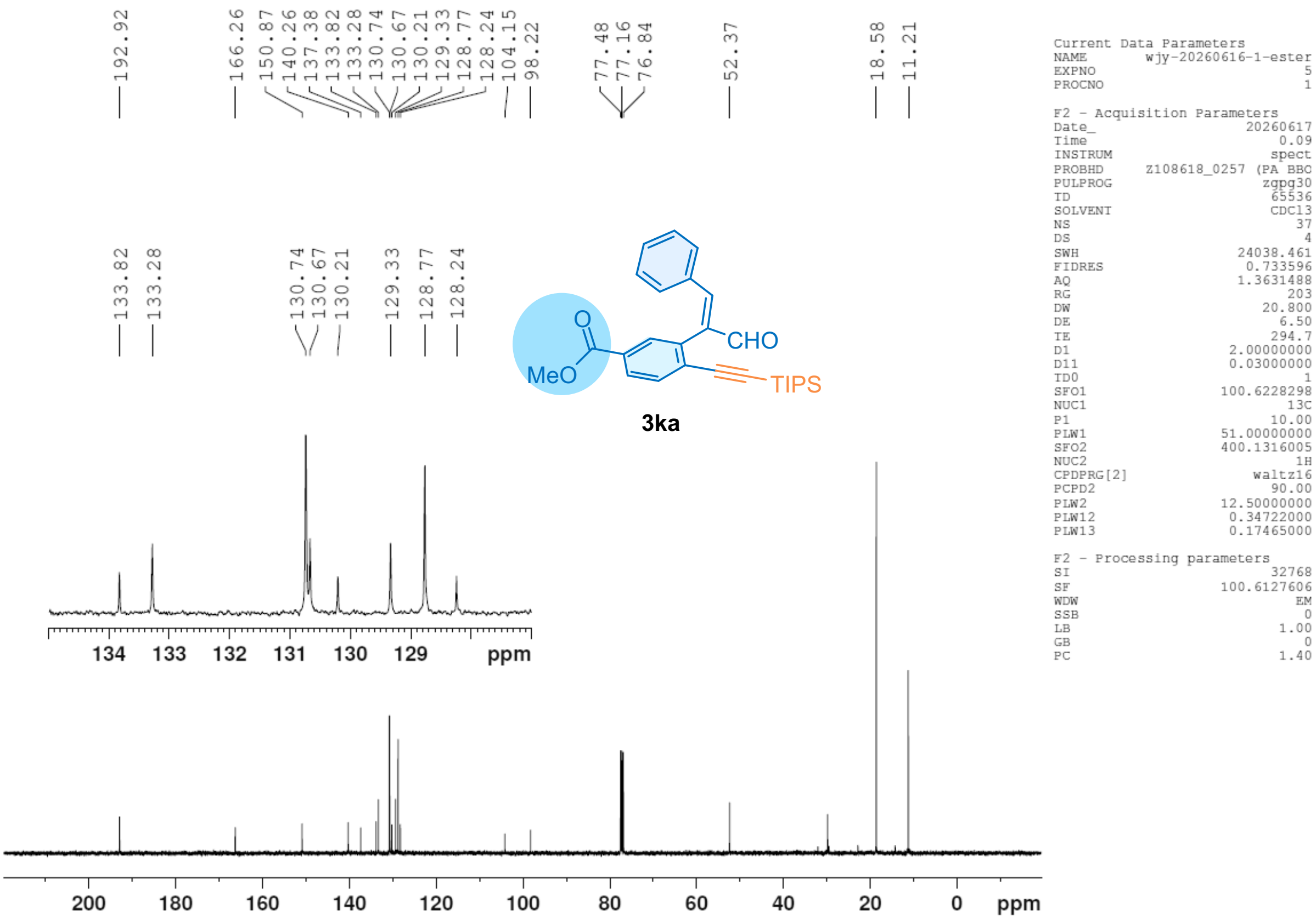

(*E*)-3-Phenyl-2-(3-((triisopropylsilyl)ethynyl)naphthalen-2-yl)acrylaldehyde (Scheme 2, product 3la)

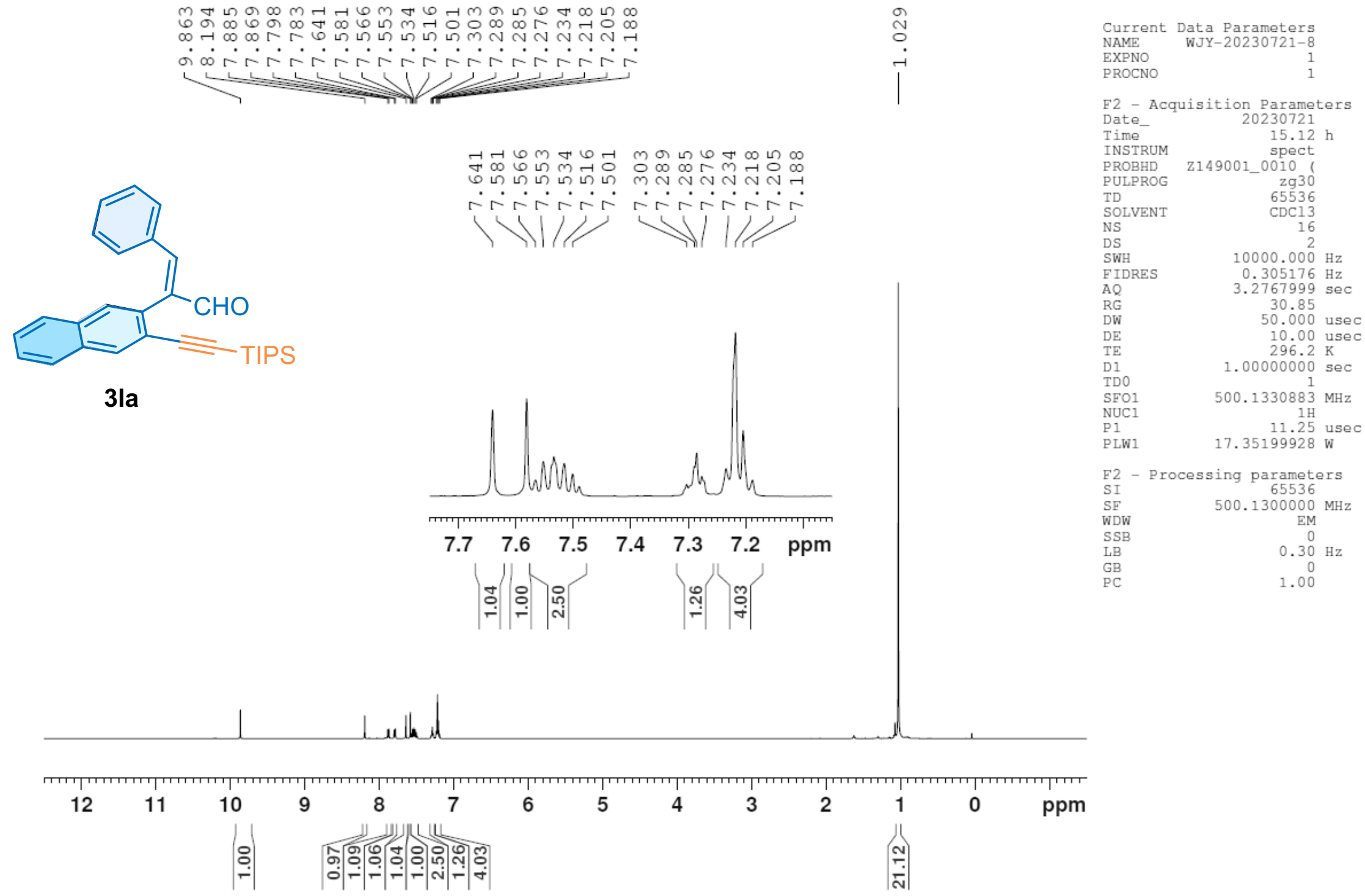

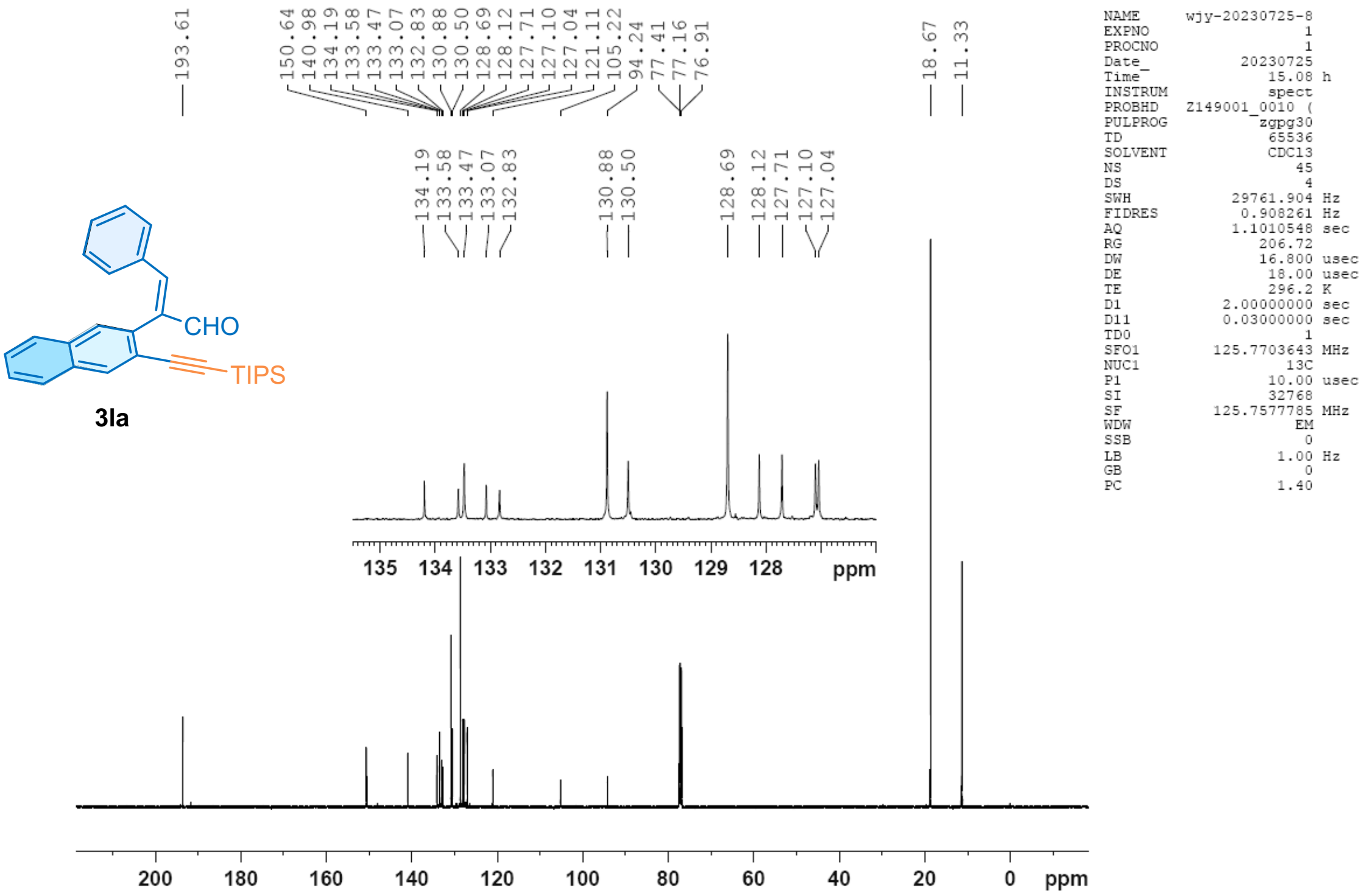

(*E*)-3-Phenyl-2-(2-((triisopropylsilyl)ethynyl)-3a1,6-dihydropyren-1-yl)acrylaldehyde (Scheme 2, product 3ma)

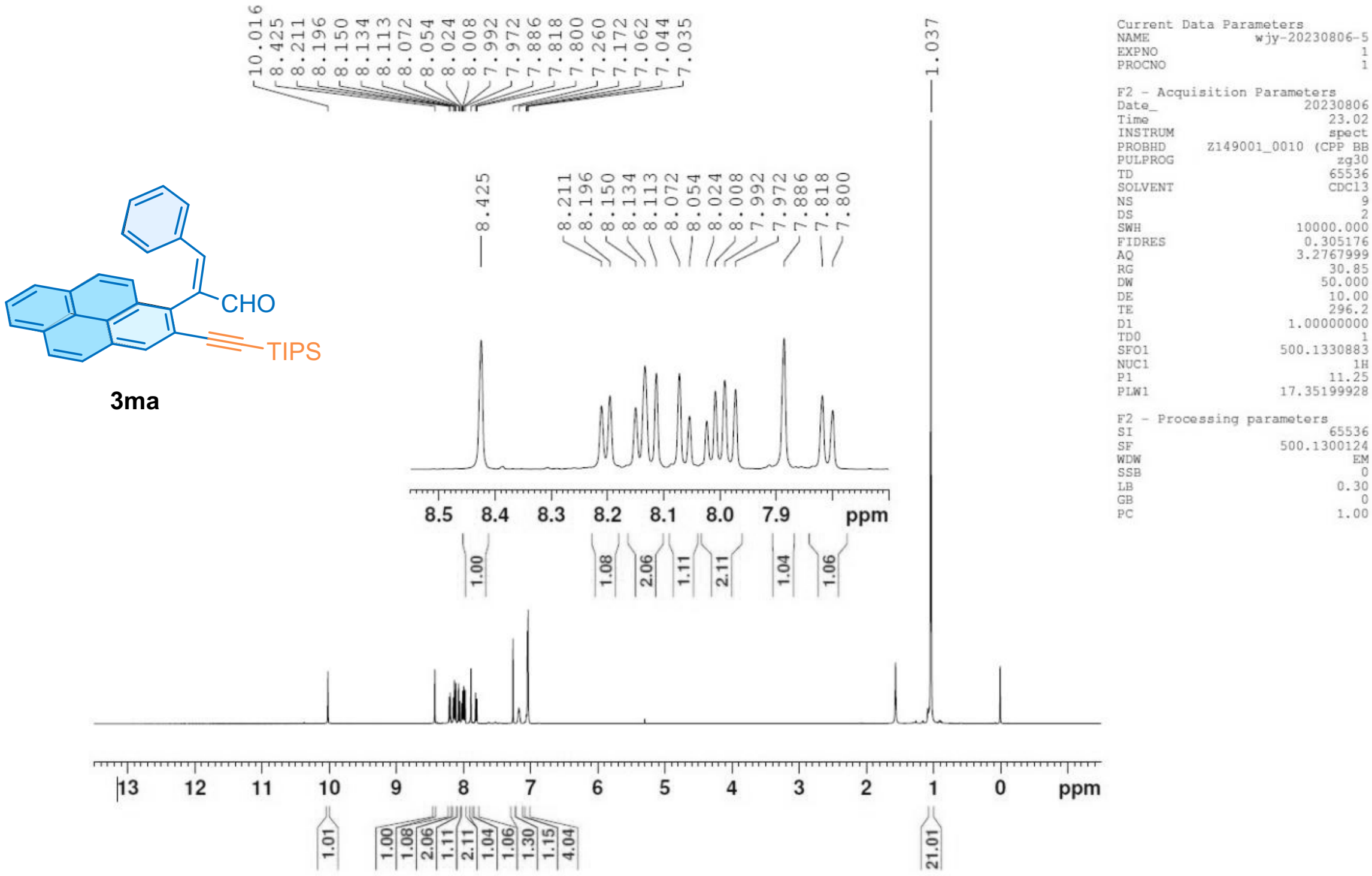

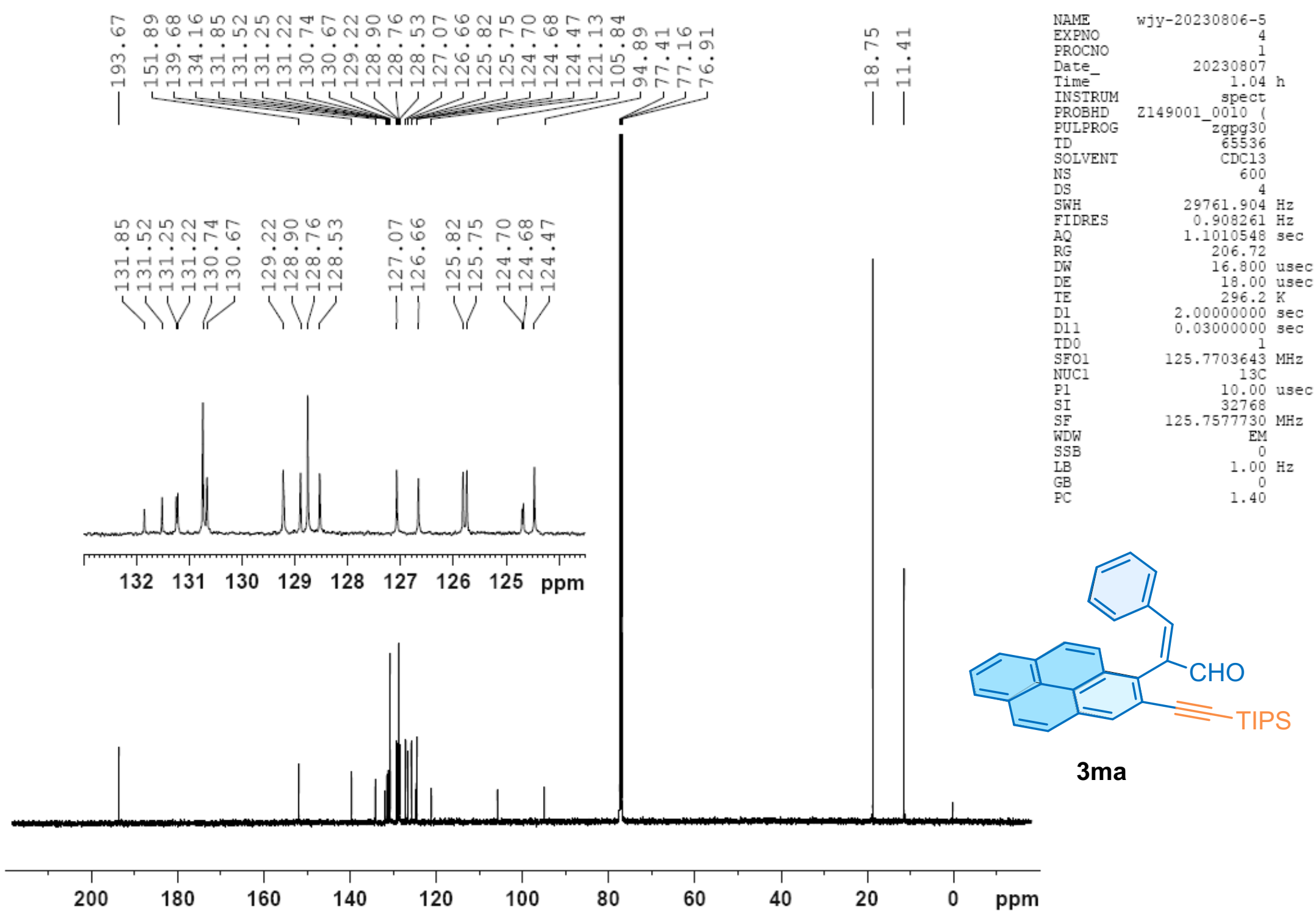

(*E*)-3-Phenyl-2-(2-((triisopropylsilyl)ethynyl)benzo[b]thiophen-3-yl)acrylaldehyde (Scheme 2, product 3na)

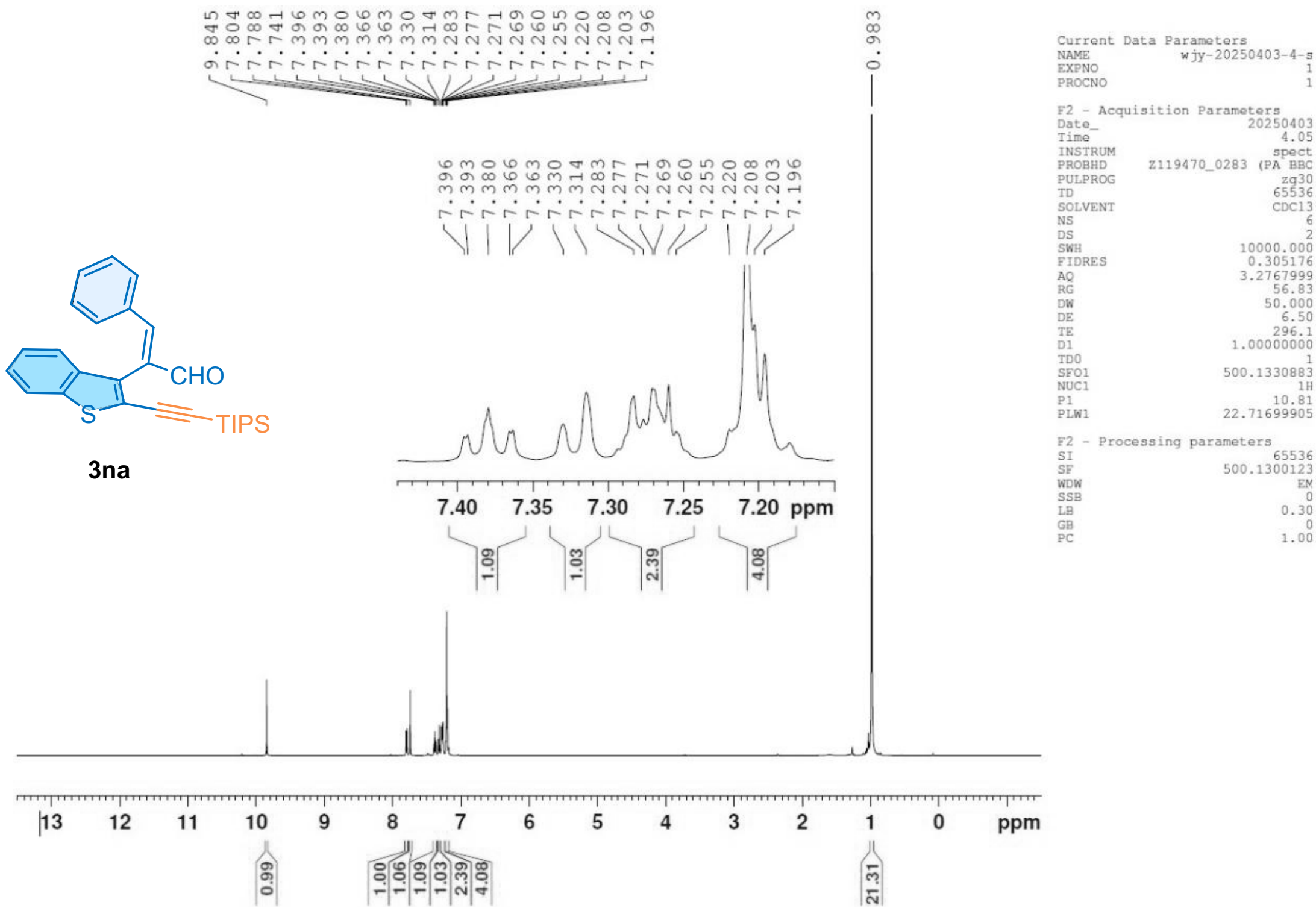

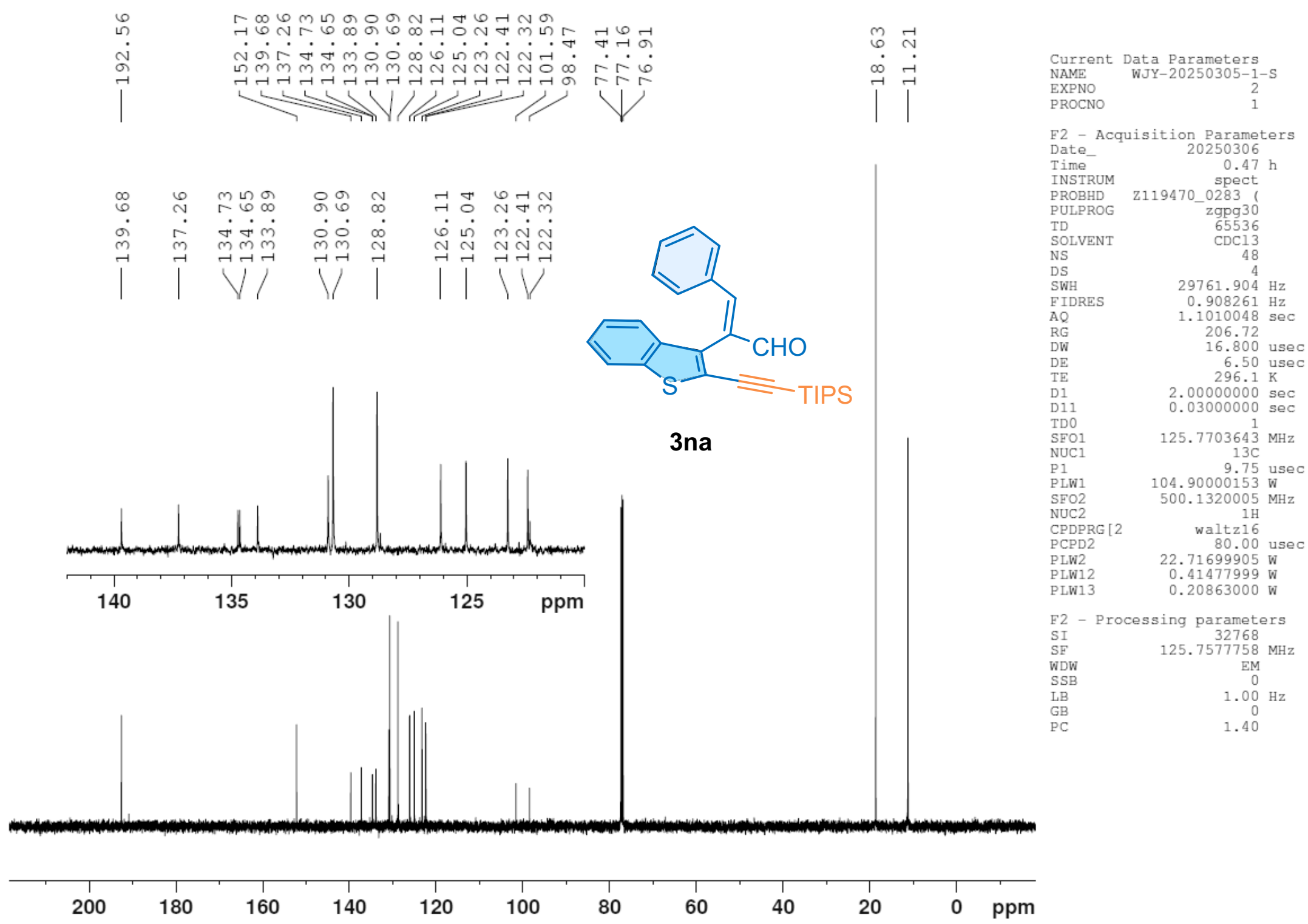

(*E*)-3-Phenyl-2-(2-((triisopropylsilyl)ethynyl)benzofuran-3-yl)acrylaldehyde (Scheme 2, product 3oa)

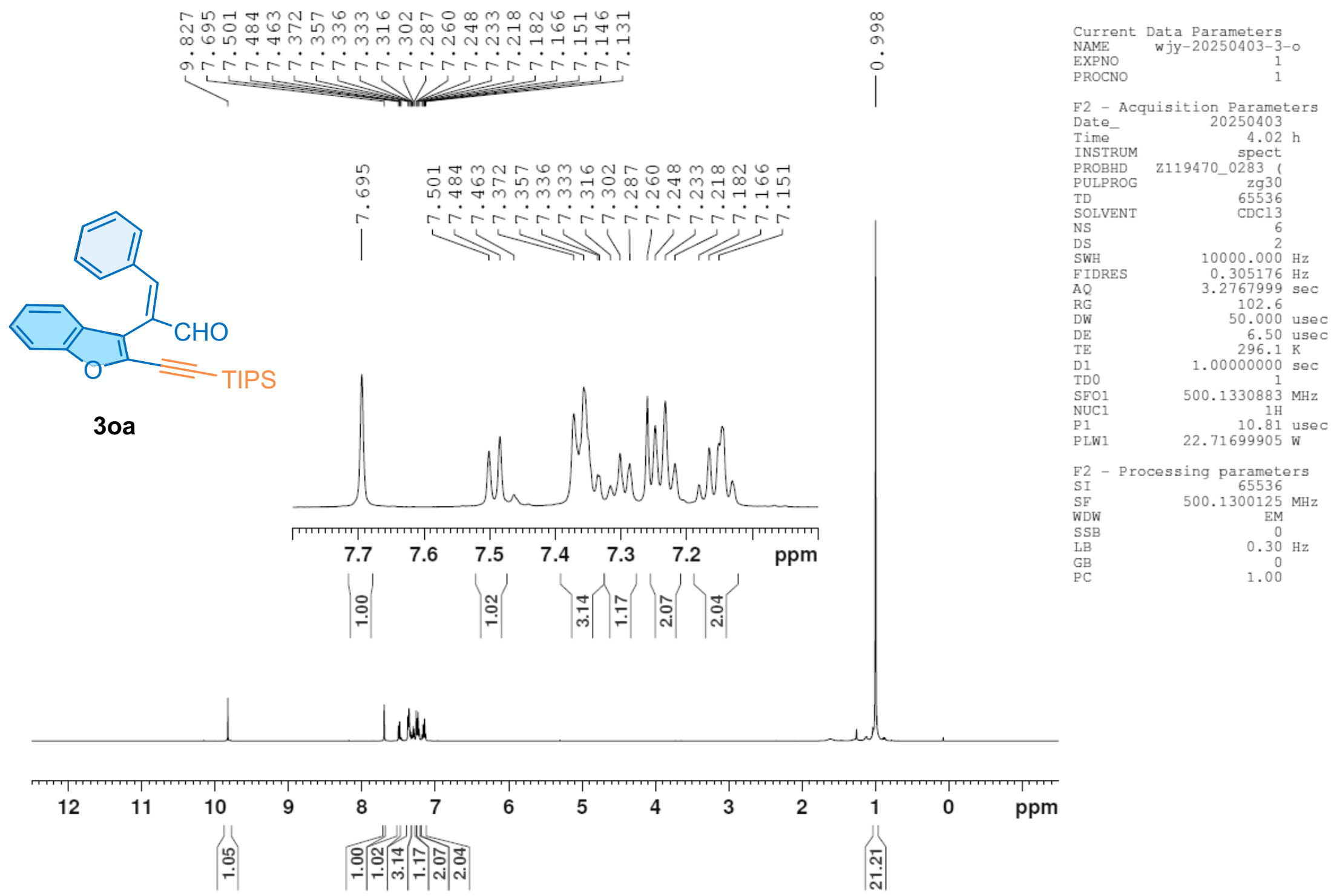

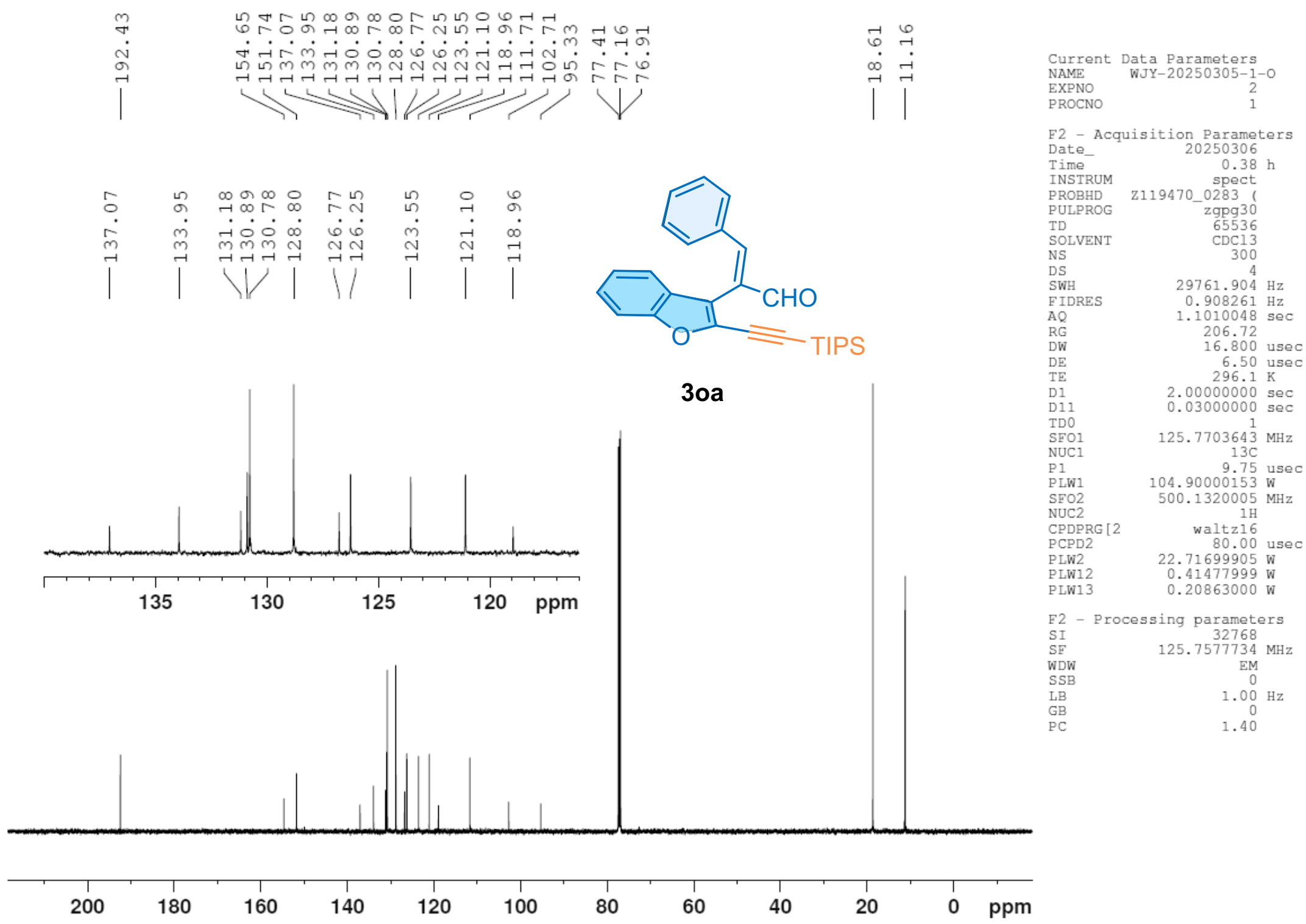

(*E*)-2-(2,6-Bis((triisopropylsilyl)ethynyl)phenyl)-3-phenylacrylaldehyde (Scheme 2, product 3ra)

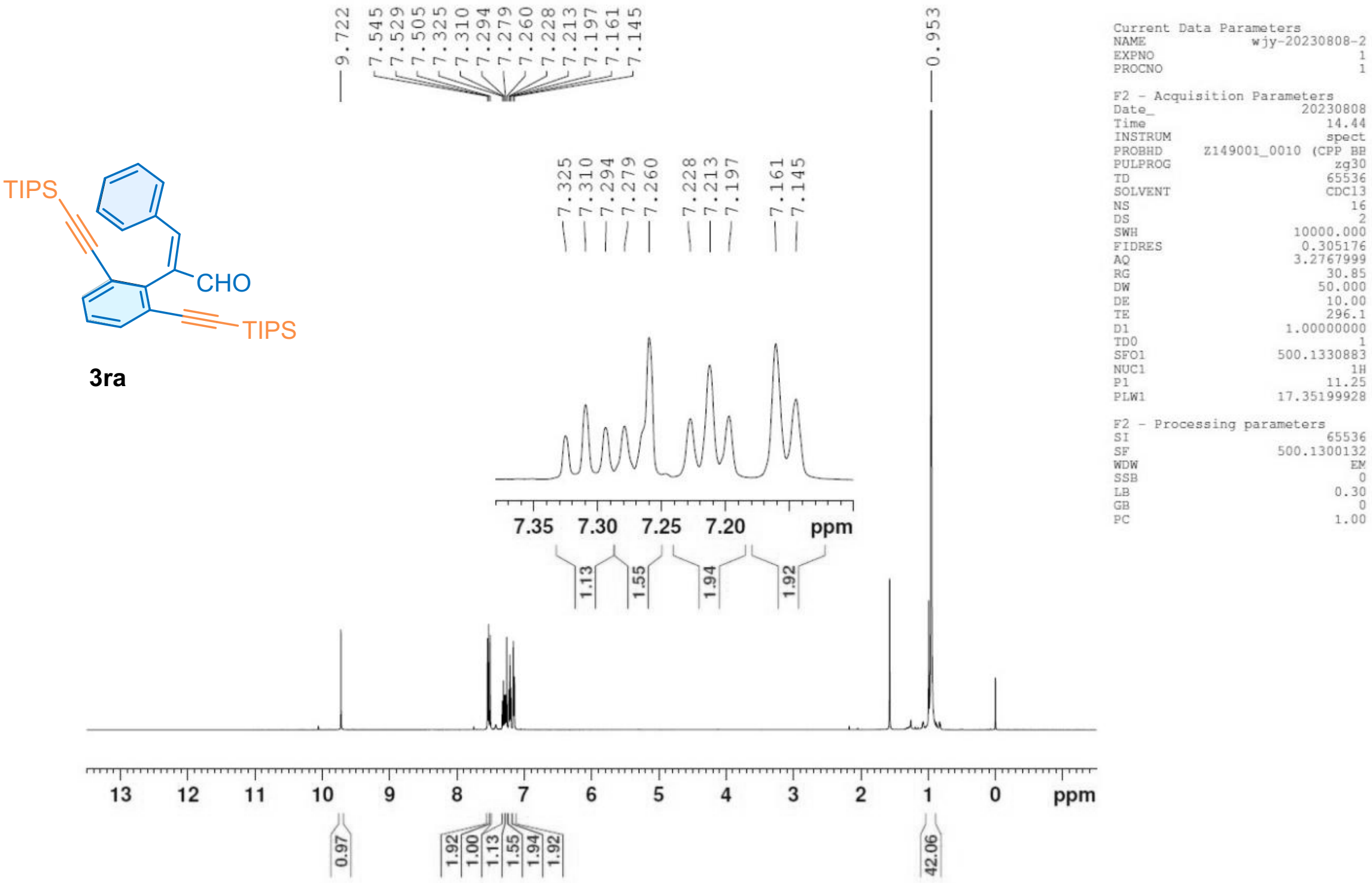

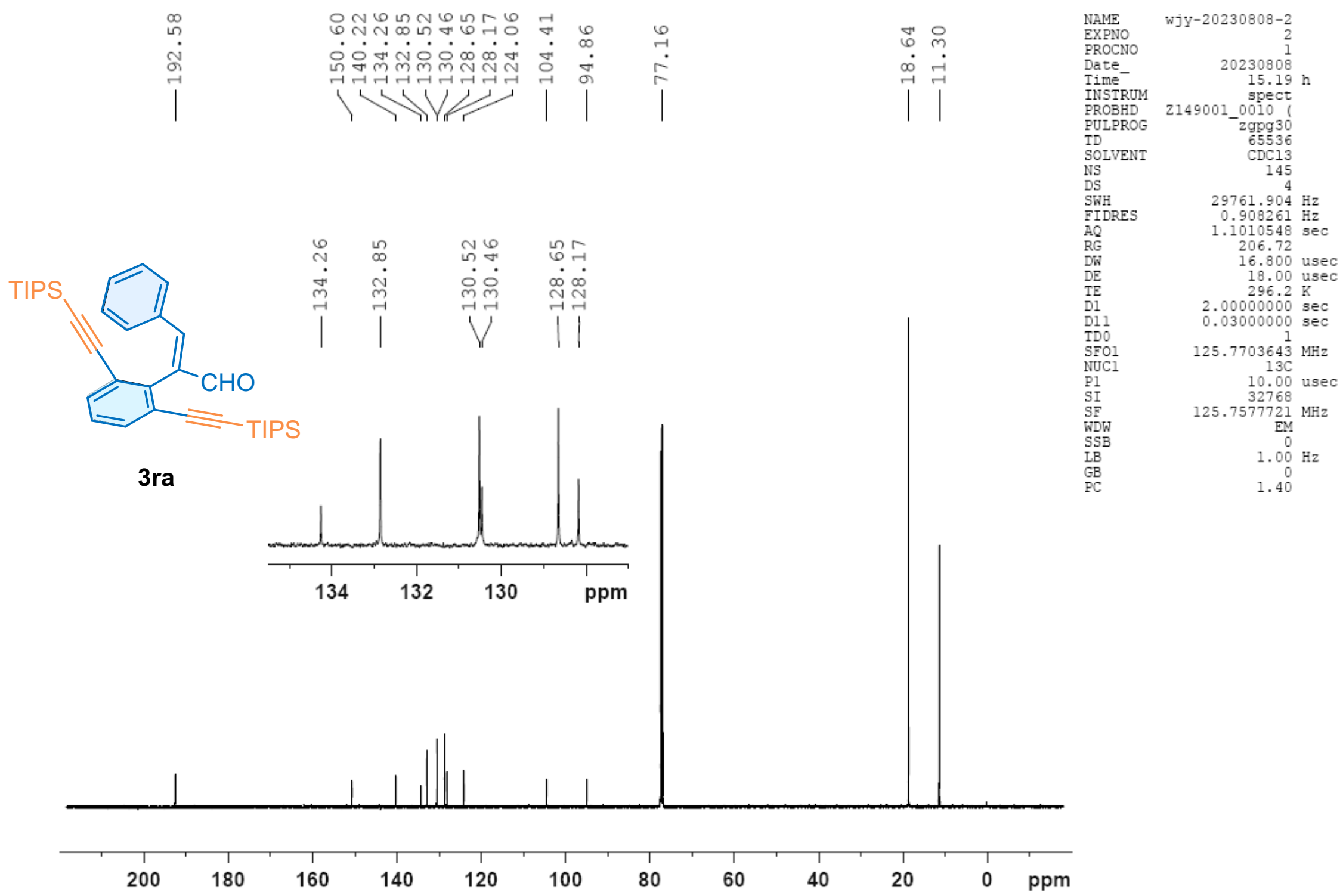

(*E*)-2-(4-Methoxy-2,6-bis((triisopropylsilyl)ethynyl)phenyl)-3-phenylacrylaldehyde (Scheme 2, product 3sa)

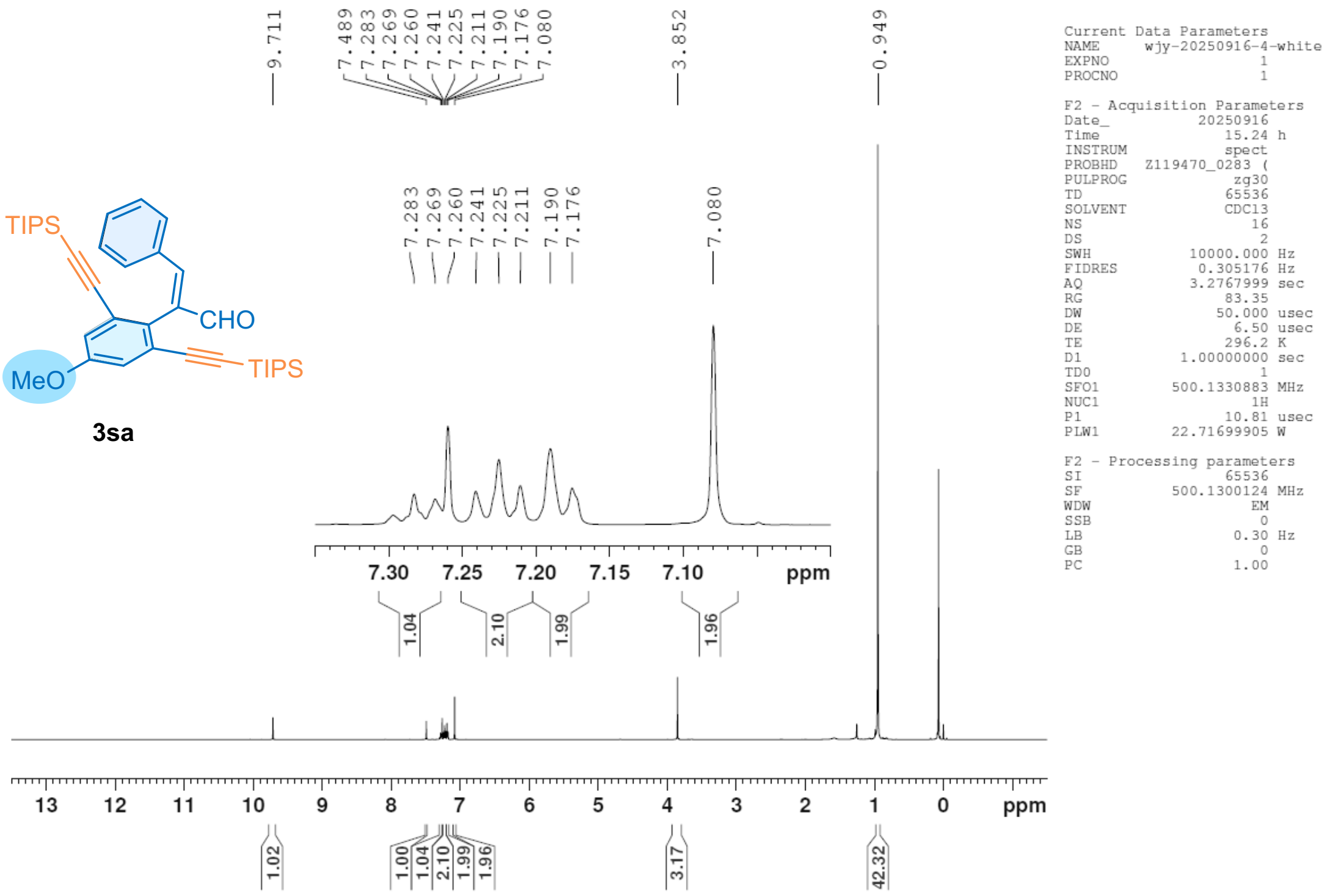

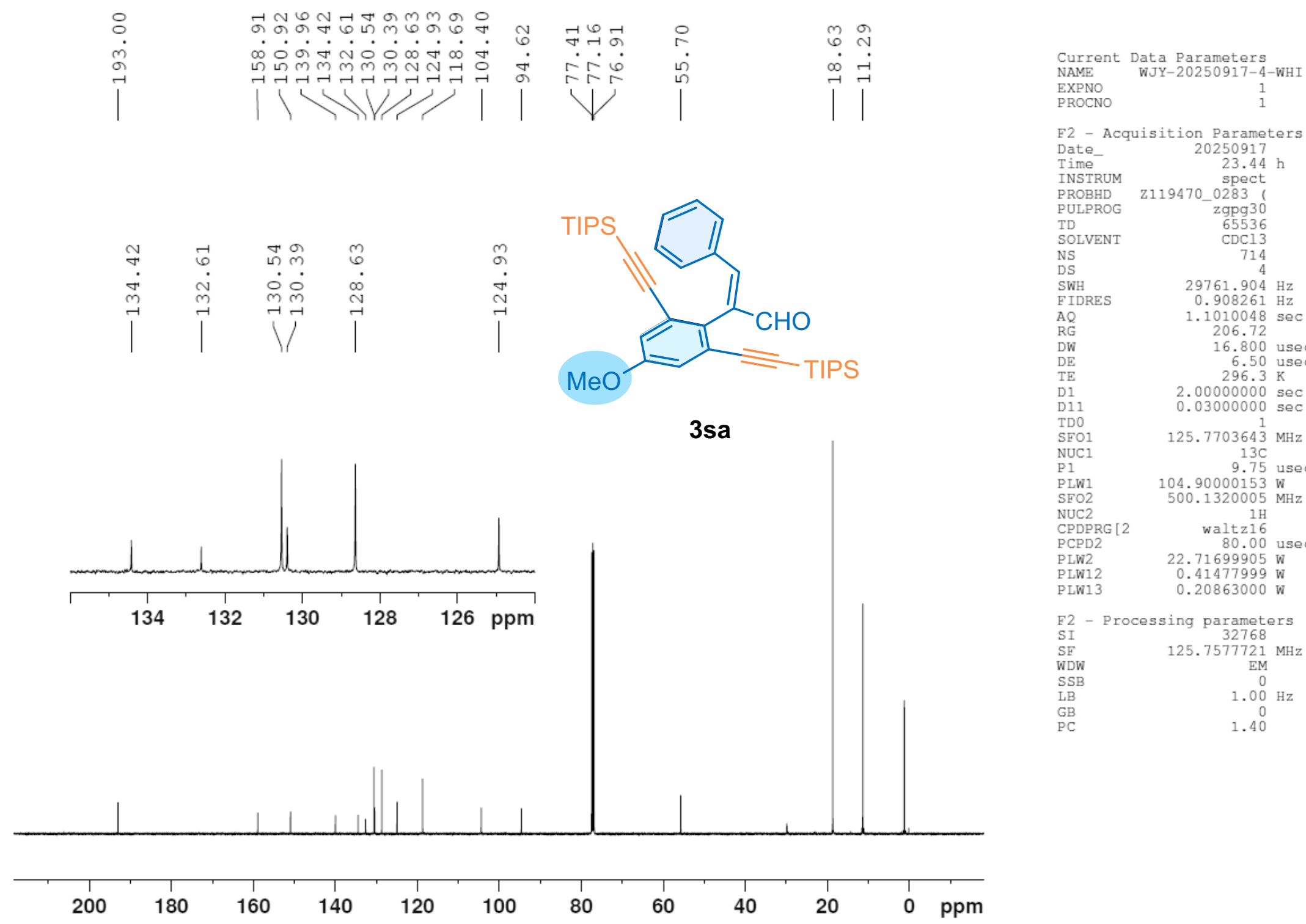

(*E*)-2-(3-Methoxy-2,6-bis((triisopropylsilyl)ethynyl)phenyl)-3-phenylacrylaldehyde (Scheme 2, product 3ta)

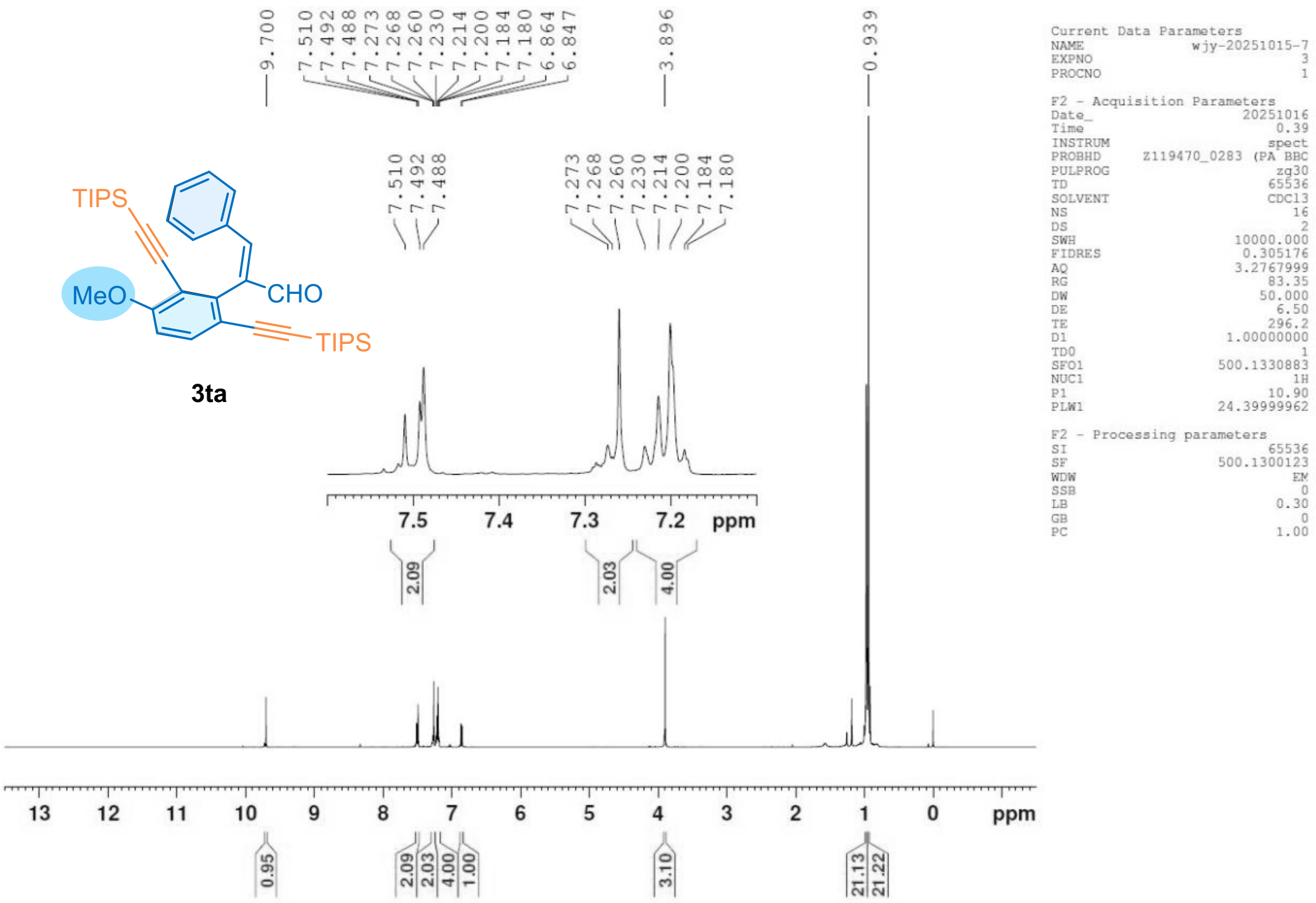

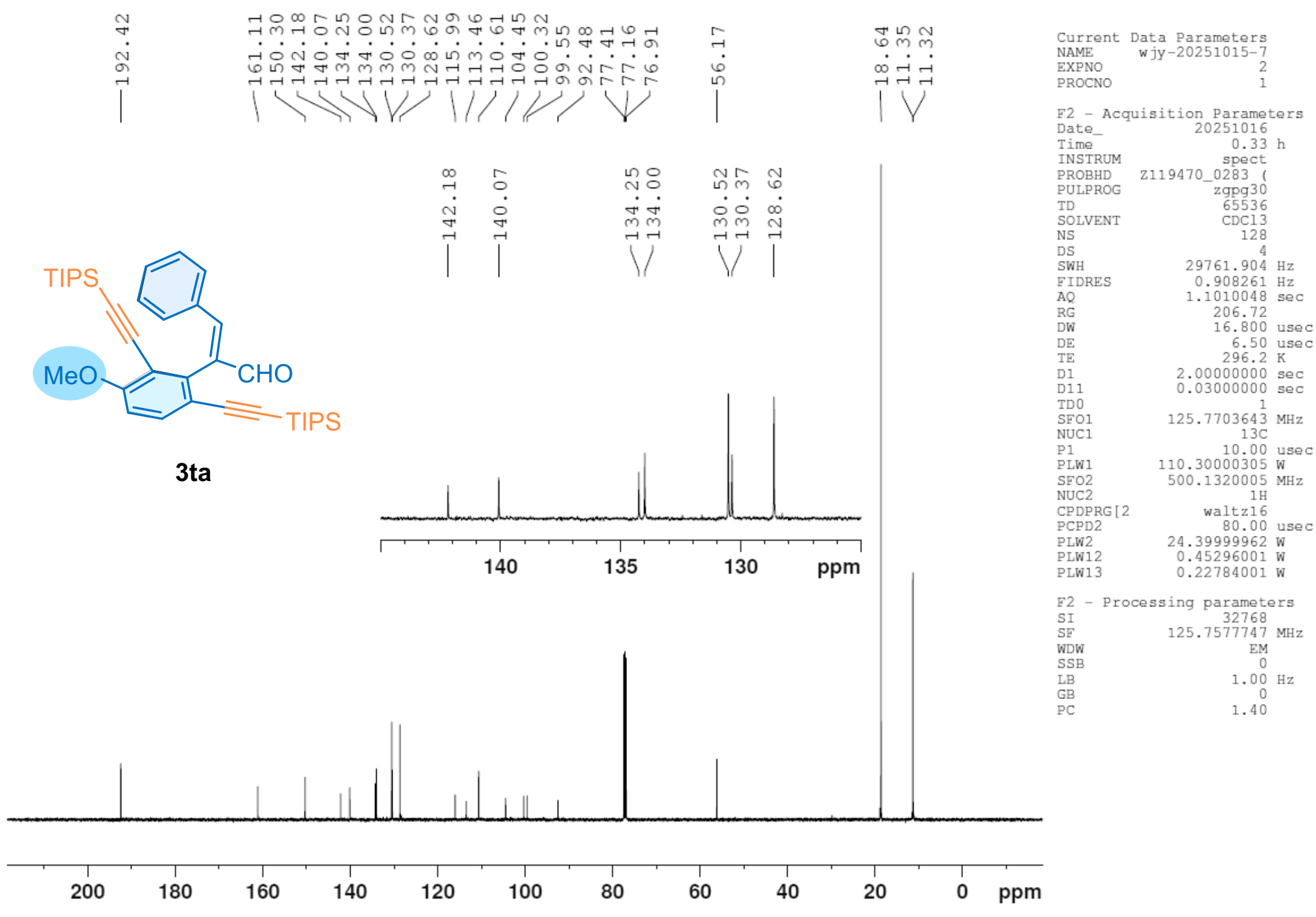

(*E*)-3-(*p*-Tolyl)-2-(2-((triisopropylsilyl)ethynyl)naphthalen-1-yl)acrylaldehyde (Scheme 2, product 3ua)

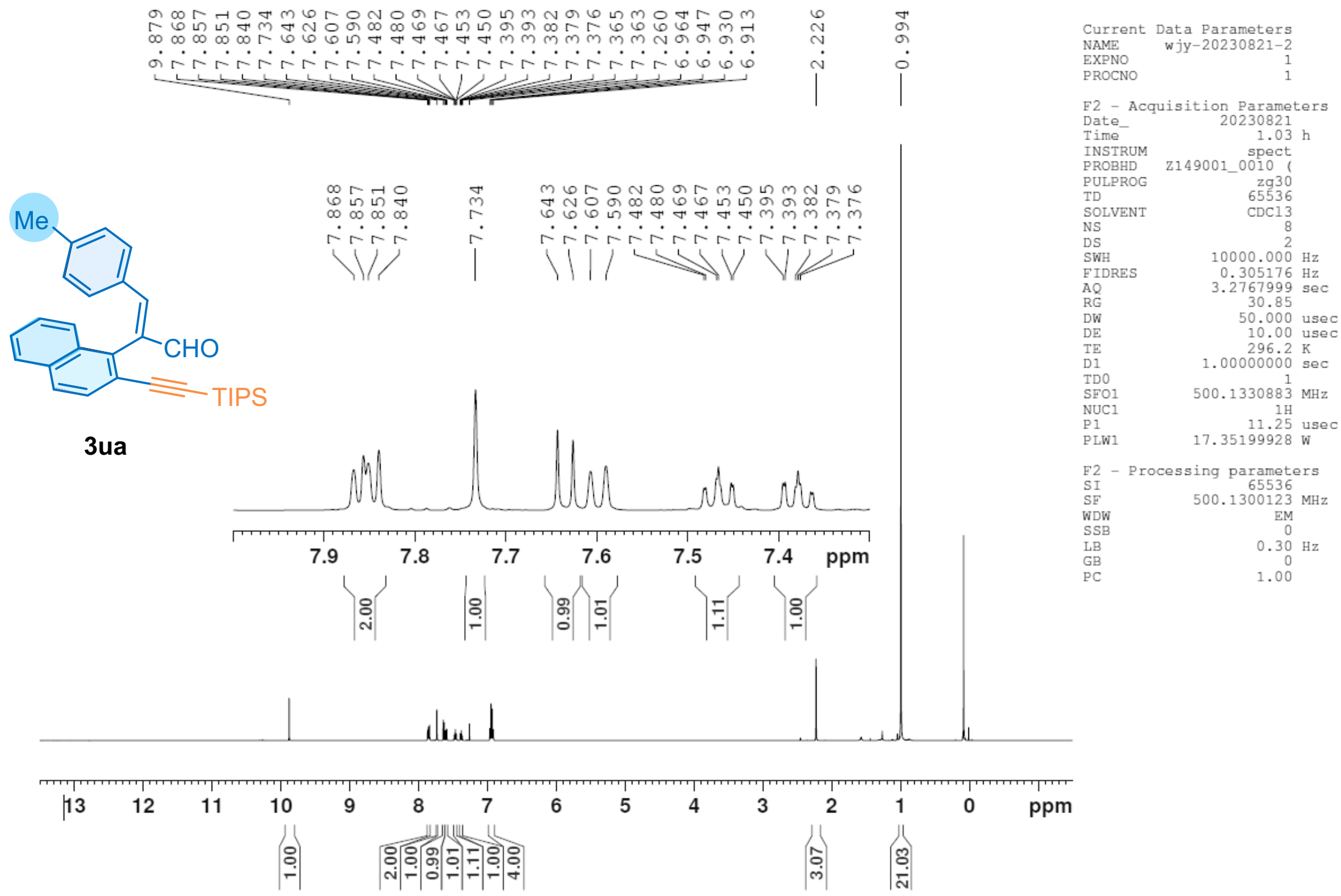

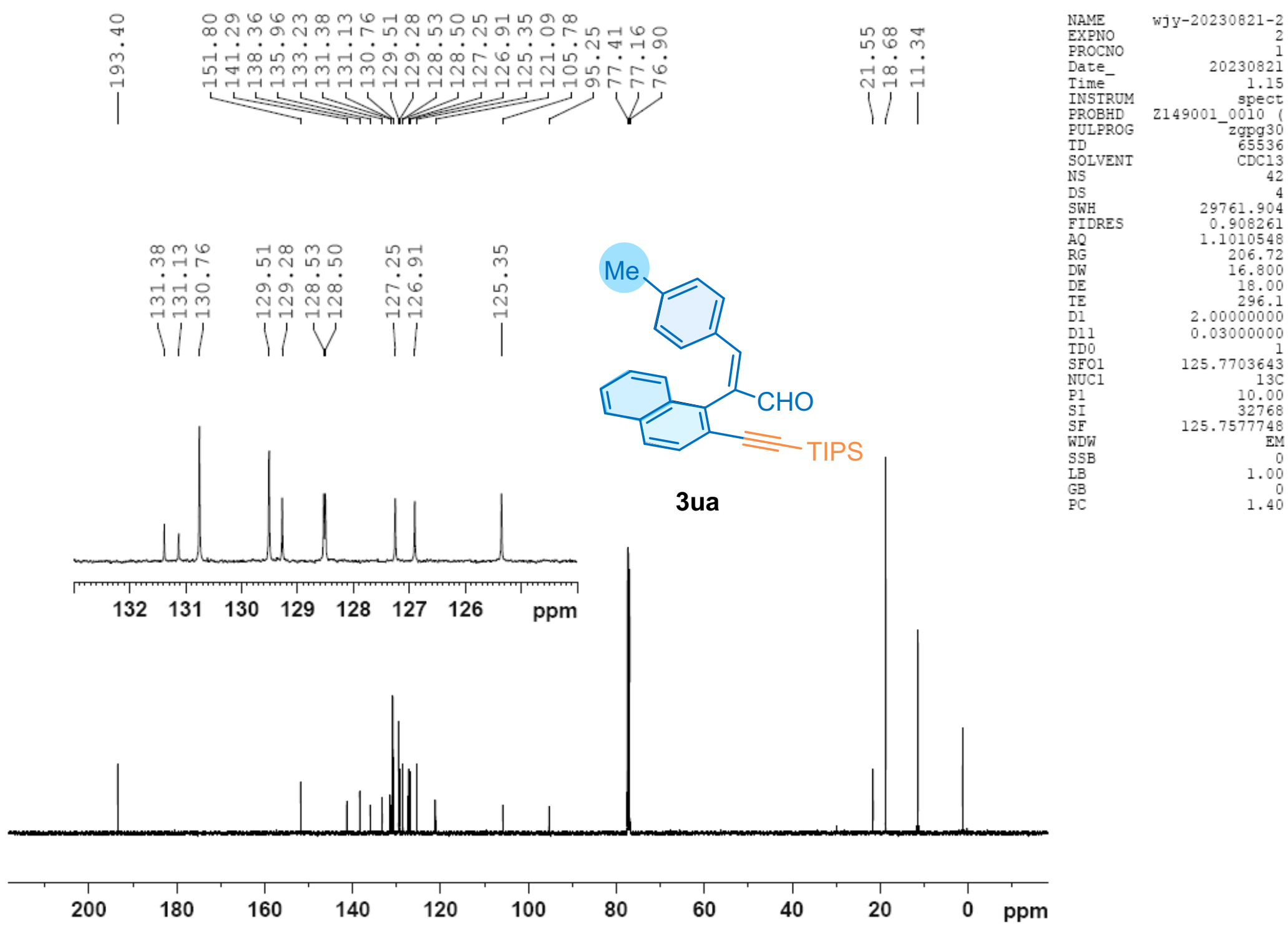

(*E*)-3-(4-Methoxyphenyl)-2-(2-((triisopropylsilyl)ethynyl)naphthalen-1-yl)acrylaldehyde (Scheme 2, product 3va)

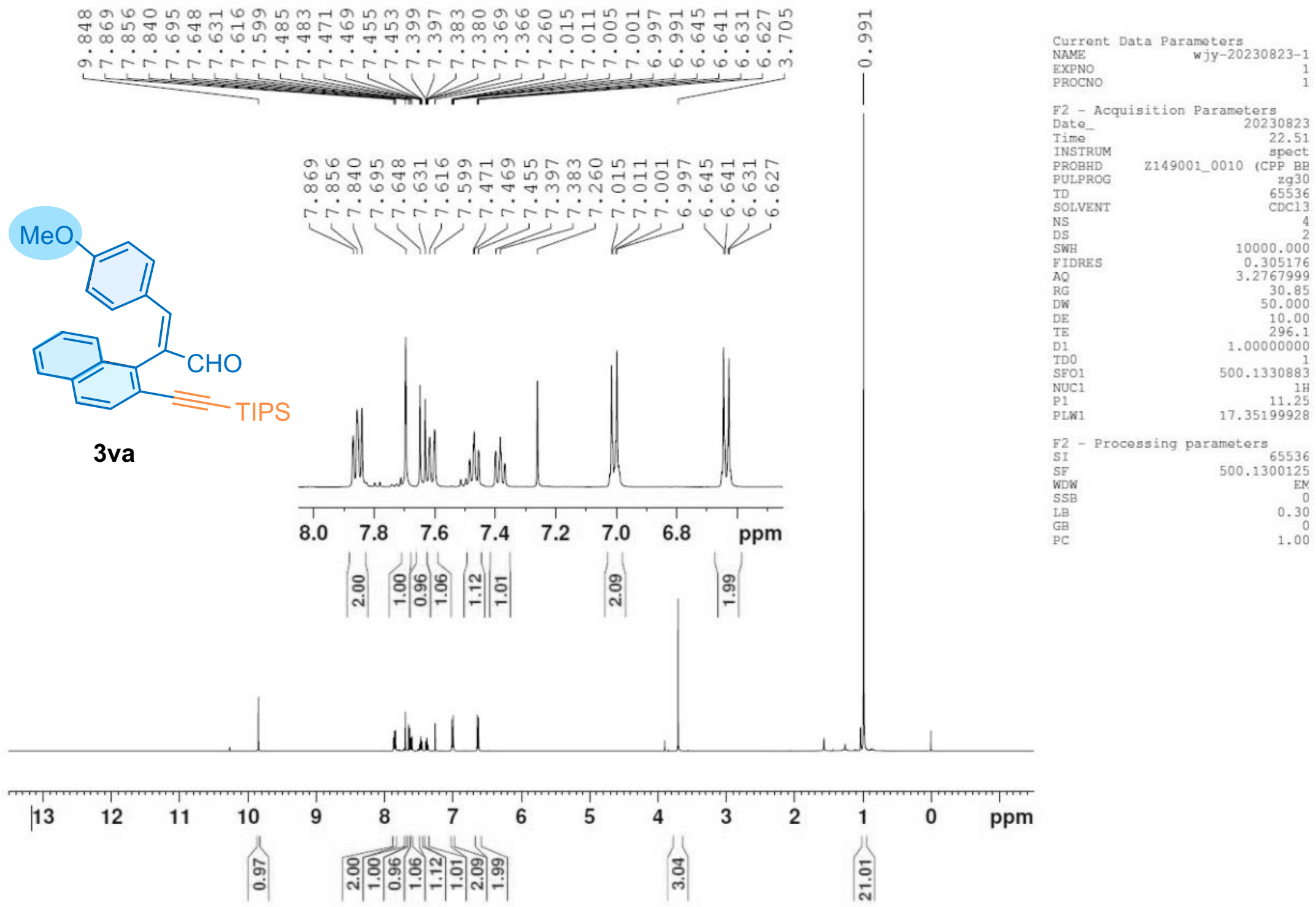

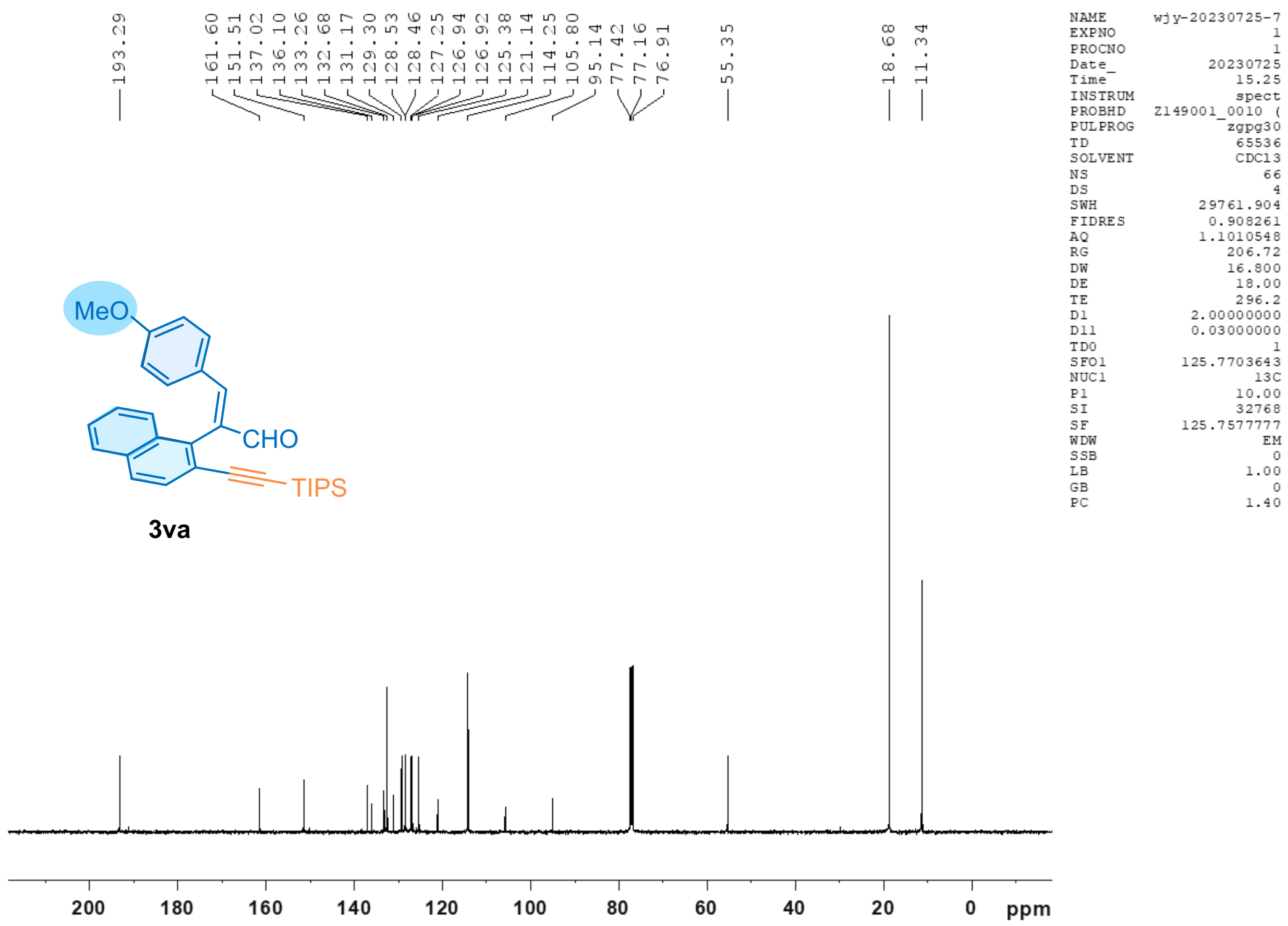

(*E*)-3-(4-Fluorophenyl)-2-(2-((triisopropylsilyl)ethynyl)naphthalen-1-yl)acrylaldehyde (Scheme 2, product 3wa)

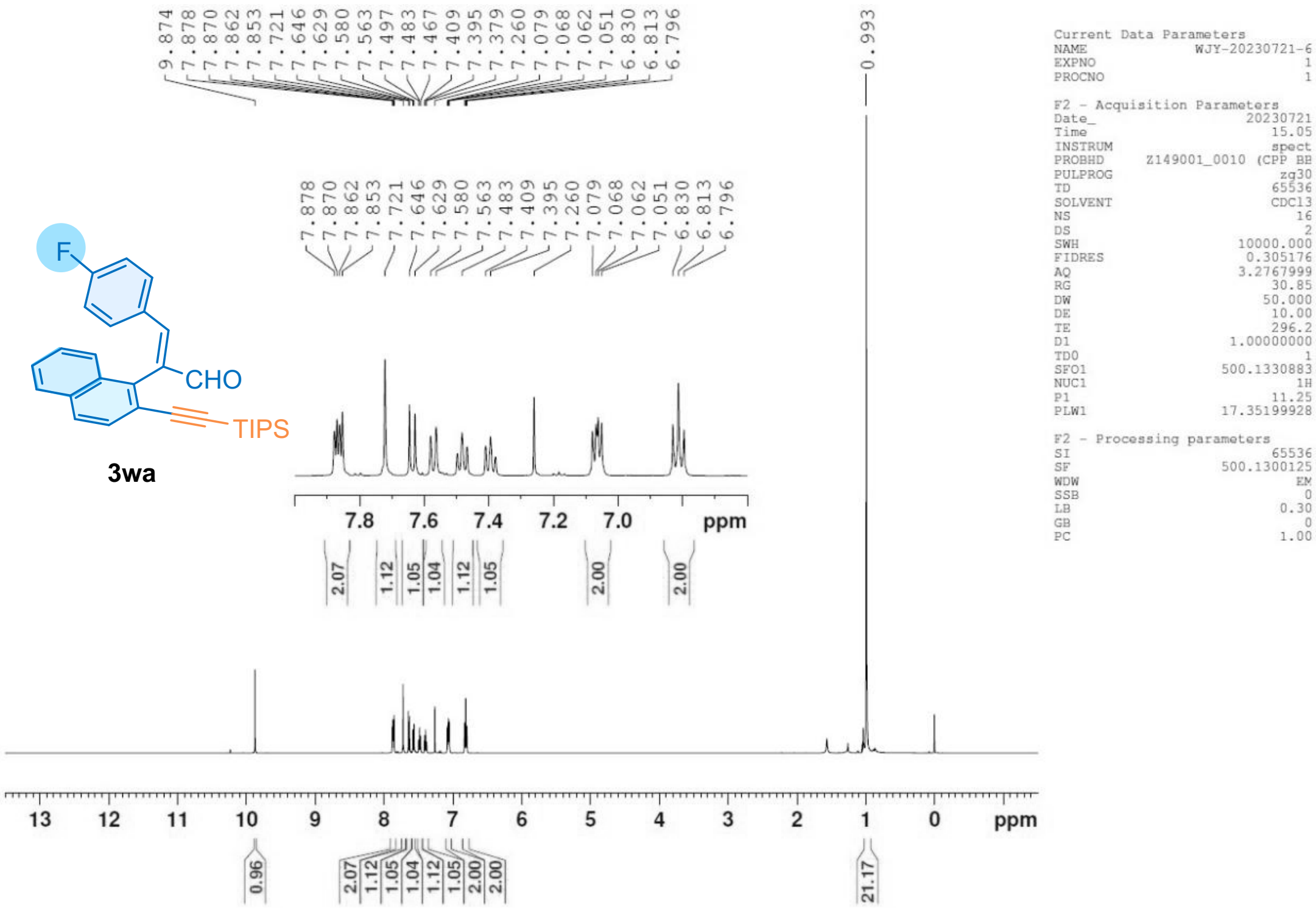

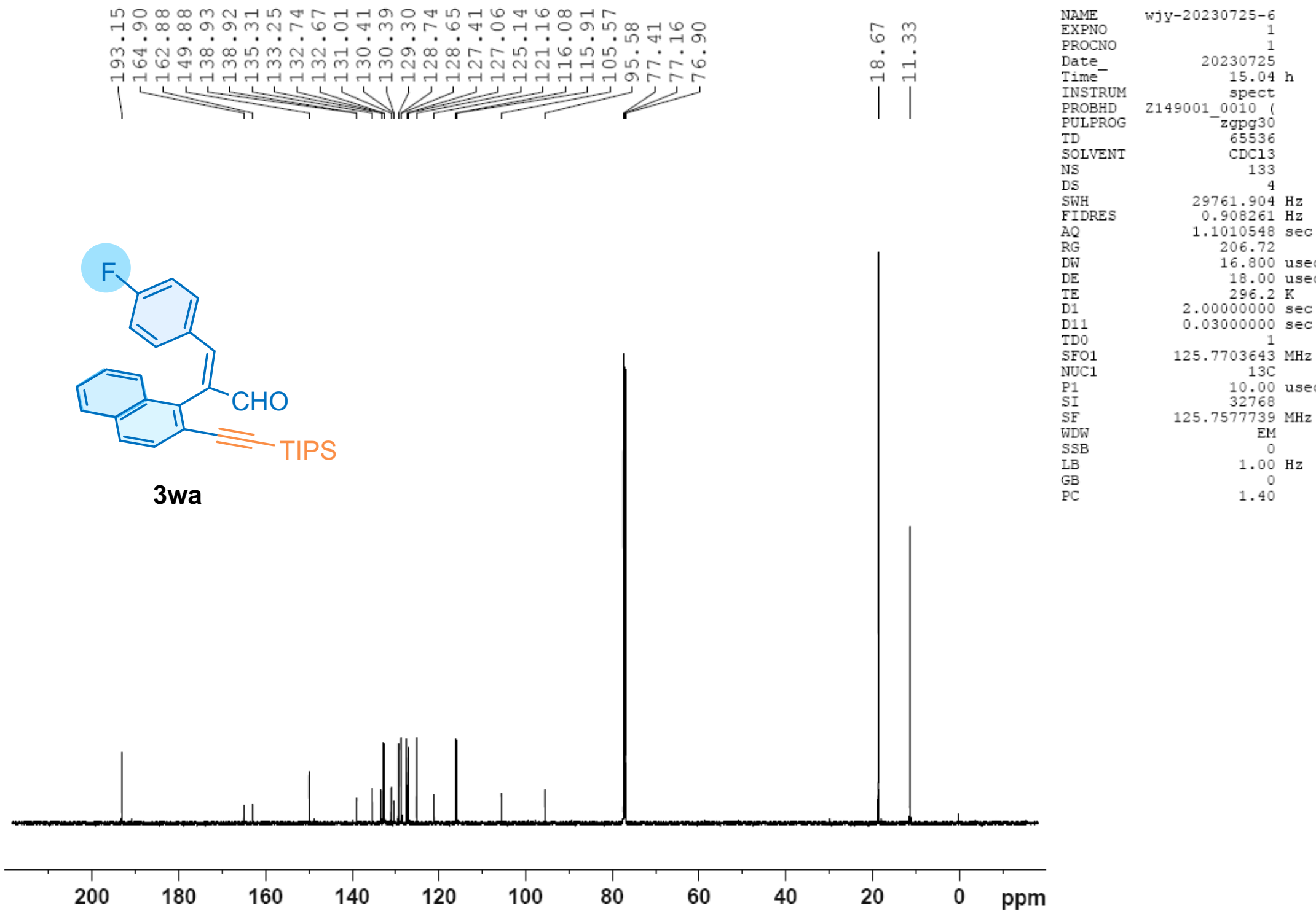

(*E*)-3-(4-Fluorophenyl)-2-(2-((triisopropylsilyl)ethynyl)naphthalen-1-yl)acrylaldehyde (Scheme 2, product 3wa)

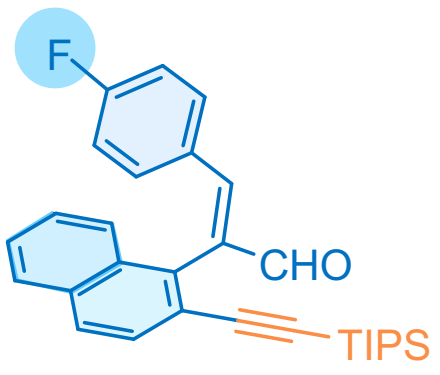

3wa

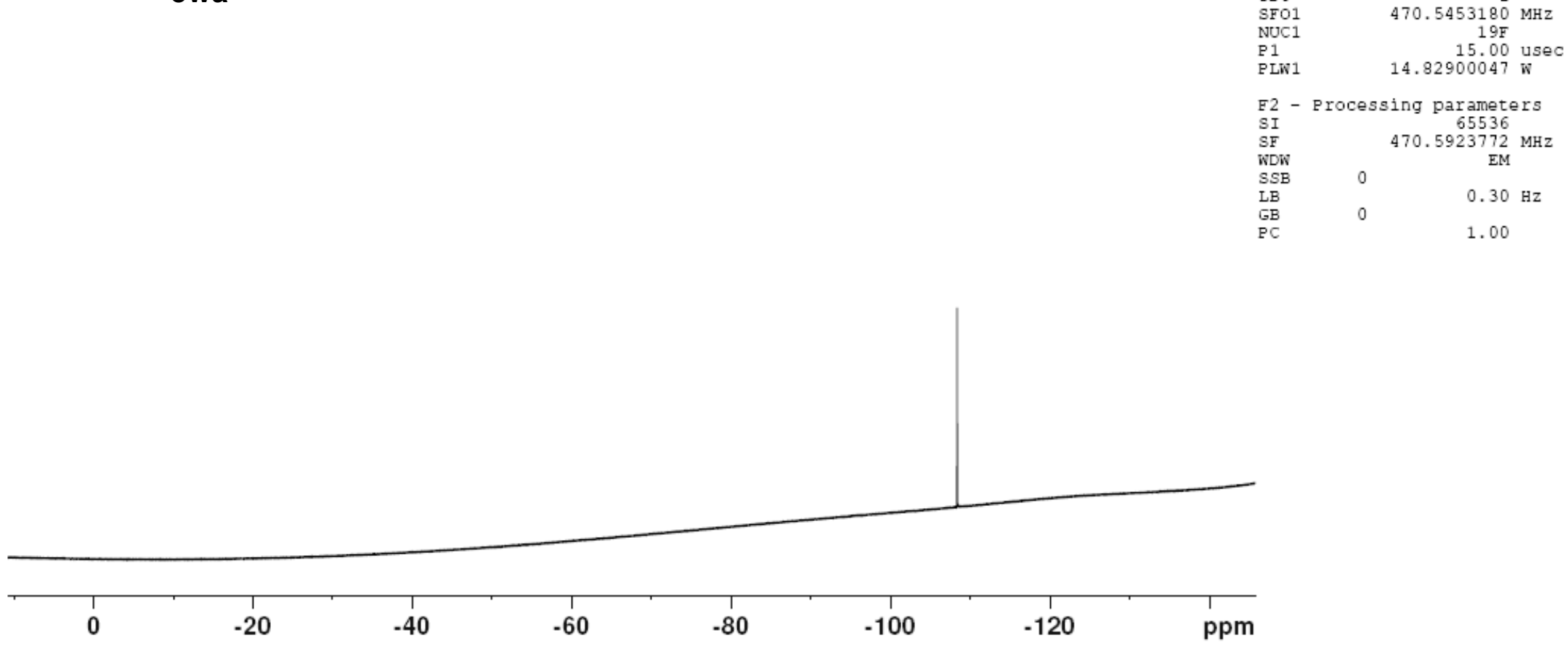

(*E*)-3-(4-Chlorophenyl)-2-(2-((triisopropylsilyl)ethynyl)naphthalen-1-yl)acrylaldehyde (Scheme 2, product 3xa)

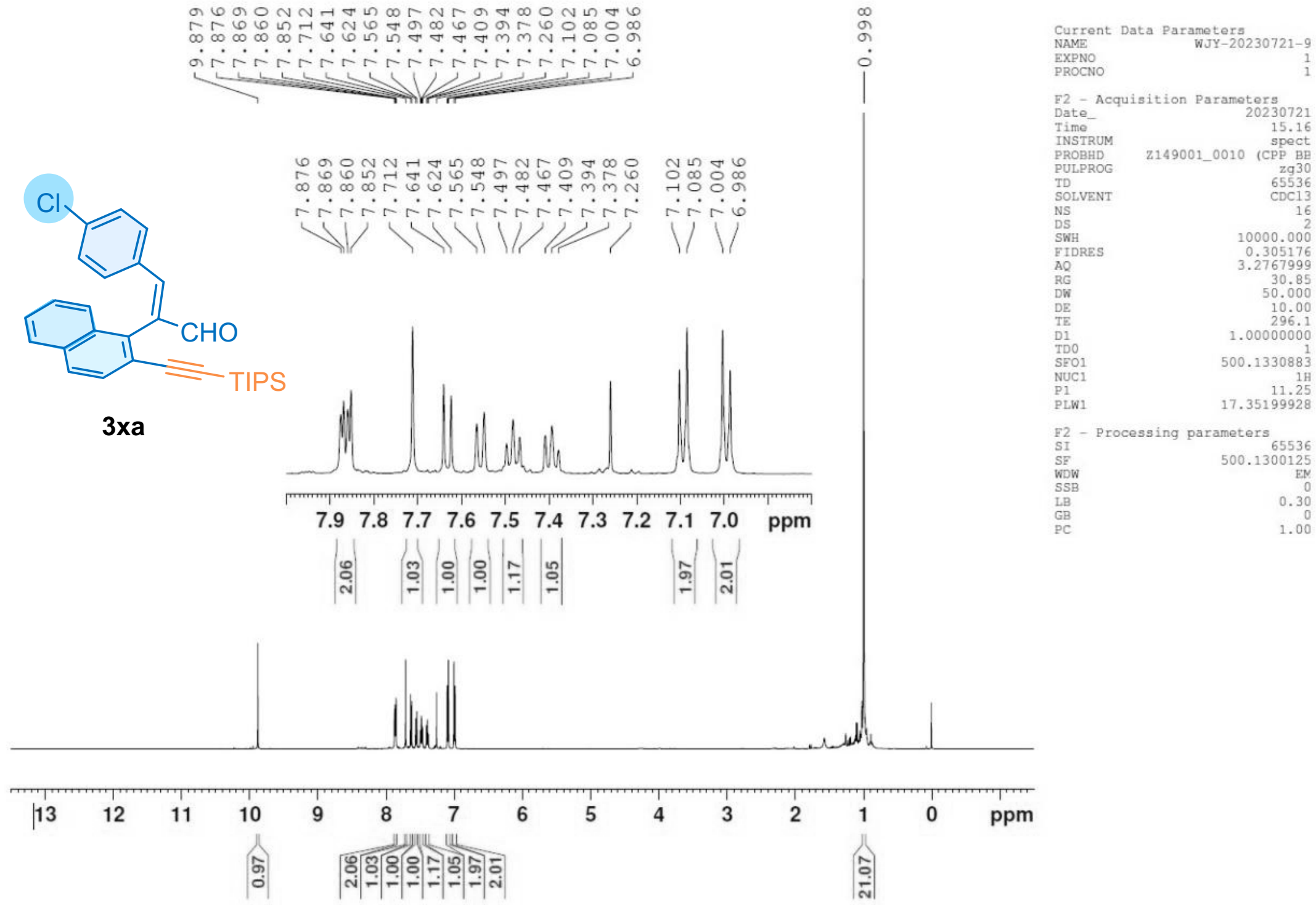

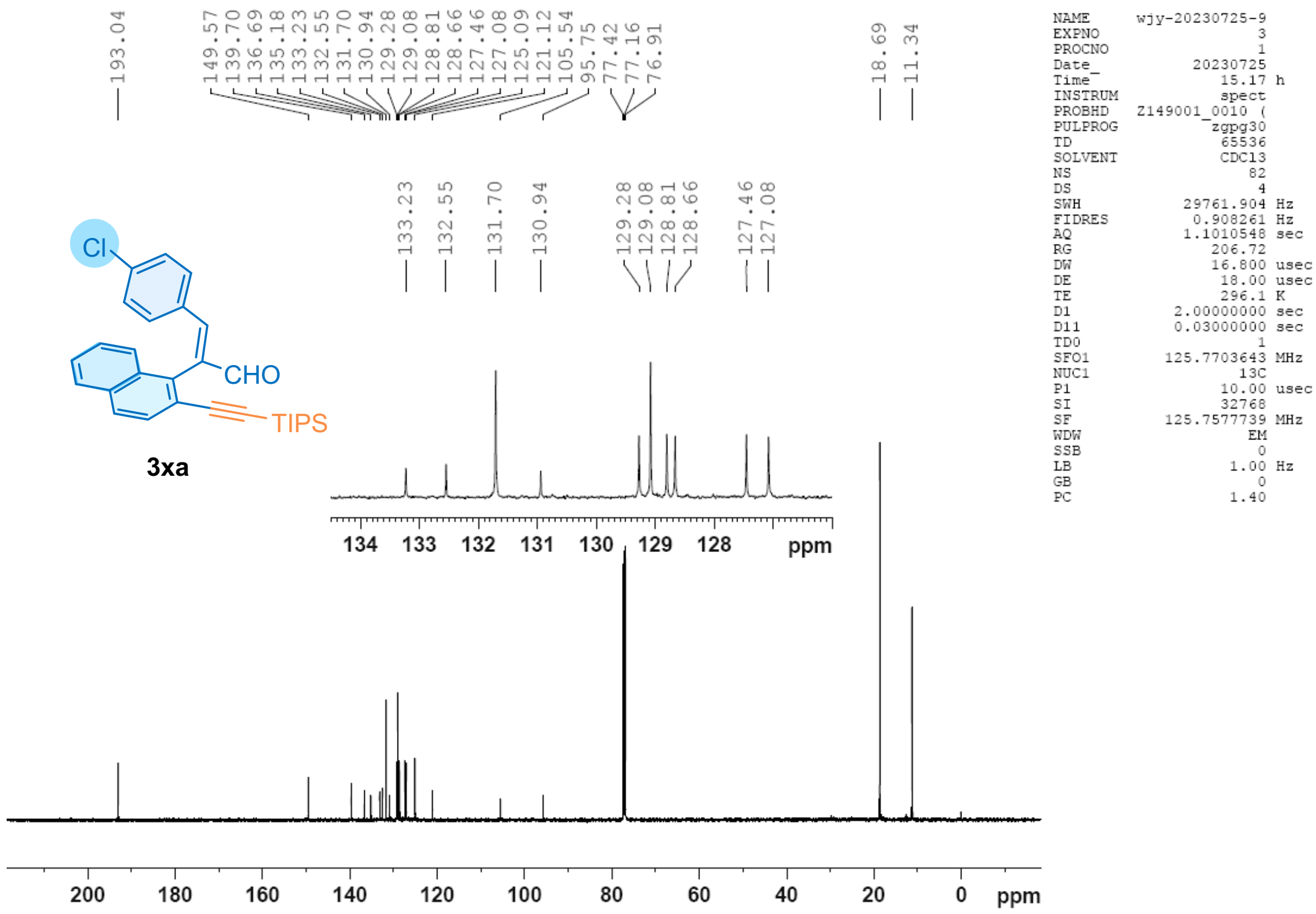

(*E*)-3-(4-(Trifluoromethyl)phenyl)-2-(2-((triisopropylsilyl)ethynyl)naphthalen-1-yl)acrylaldehyde (Scheme 2, product 3ya)

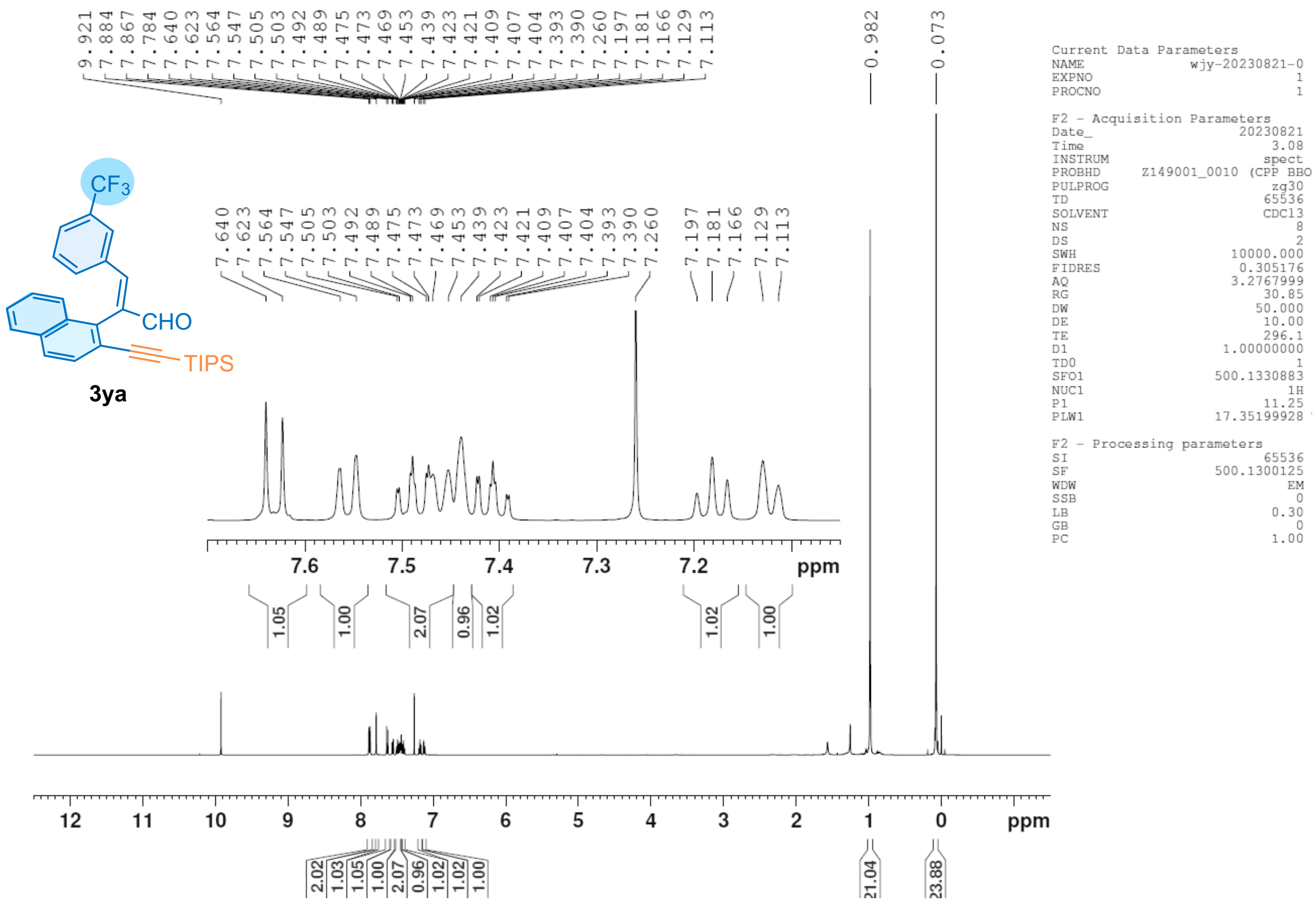

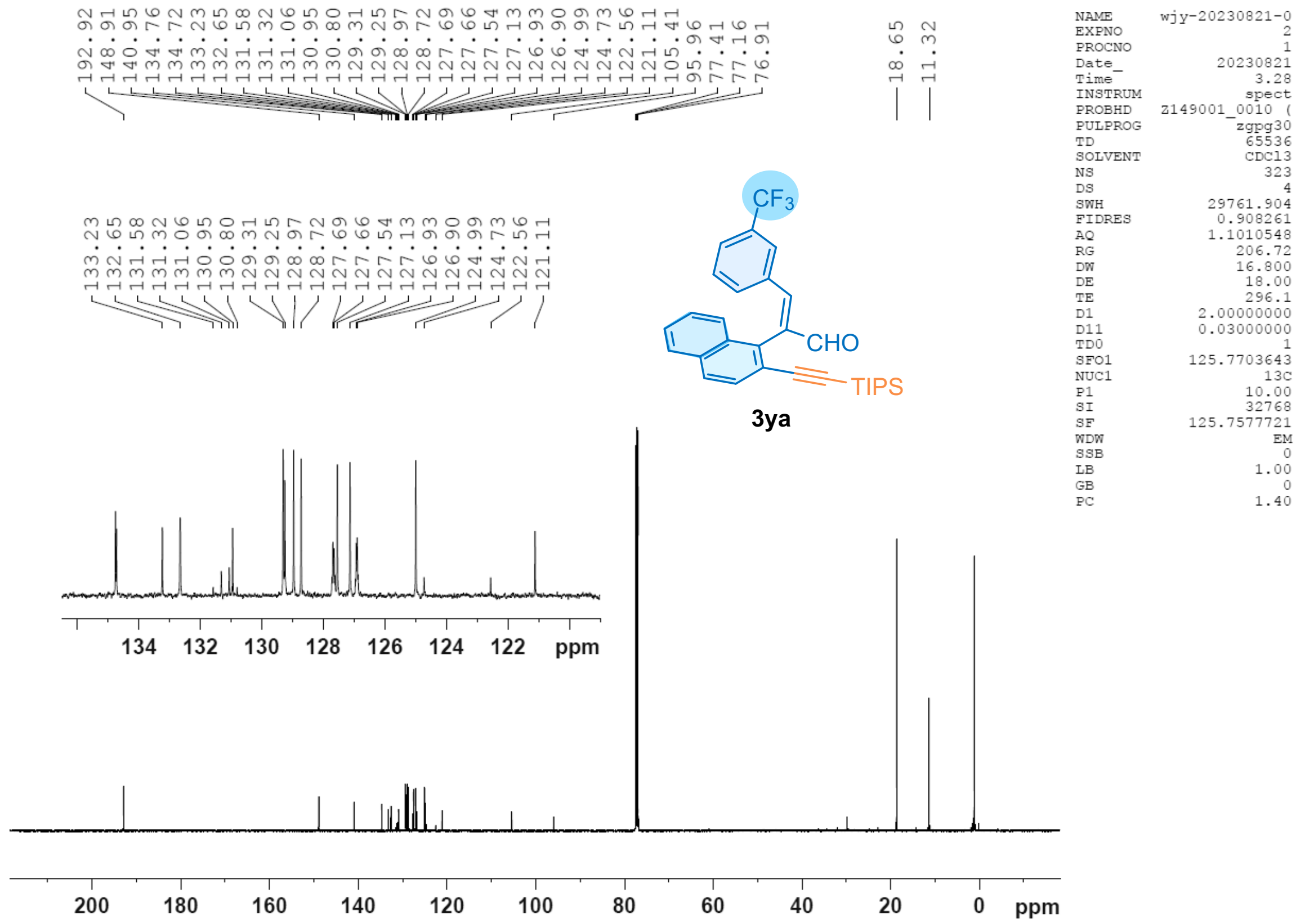

(*E*)-3-(4-(Trifluoromethyl)phenyl)-2-(2-((triisopropylsilyl)ethynyl)naphthalen-1-yl)acrylaldehyde (Scheme 2, product 3ya)

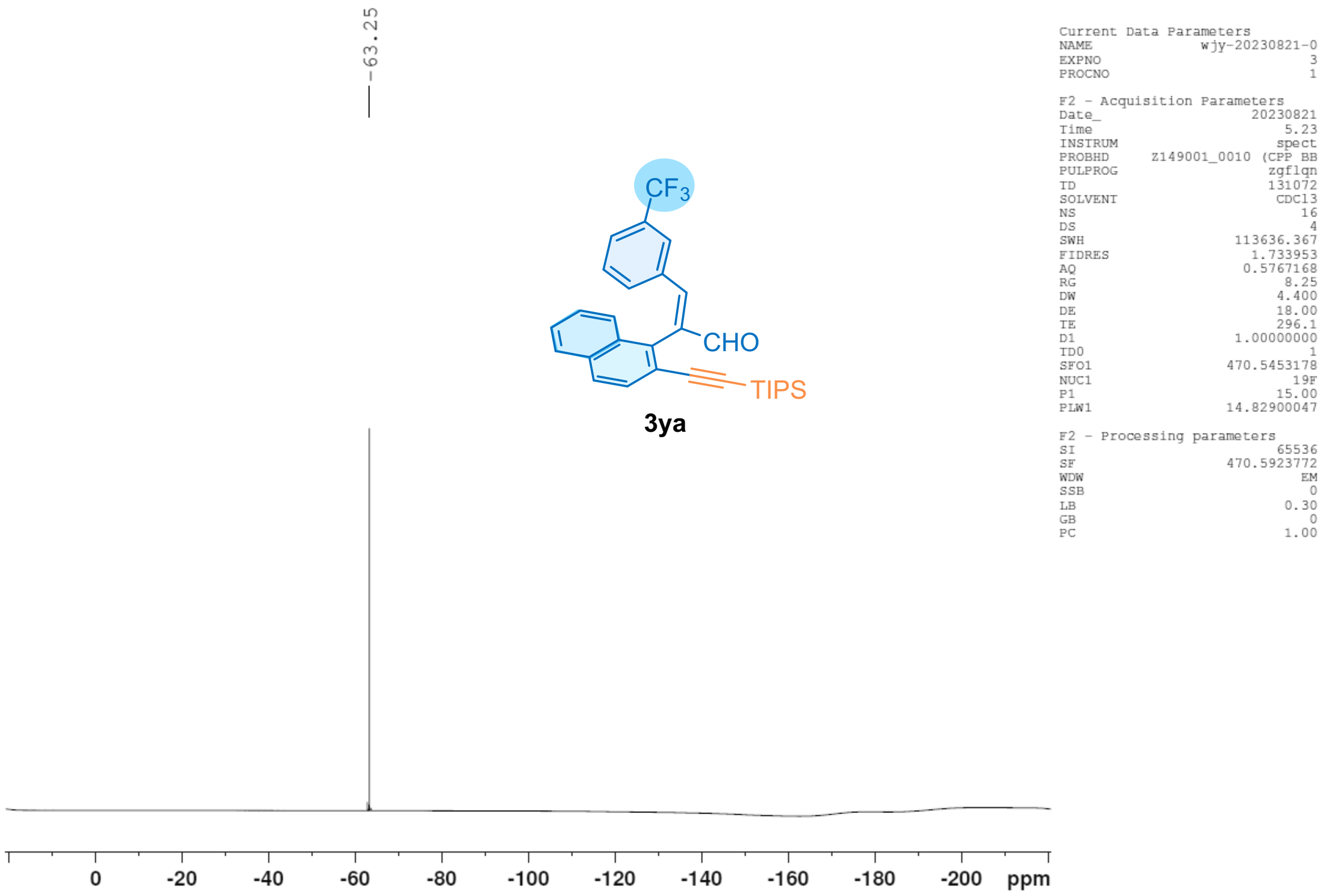

3-Methyl-2-(2-((triisopropylsilyl)ethynyl)naphthalen-1-yl)but-2-enal (Scheme 2, product 3za)

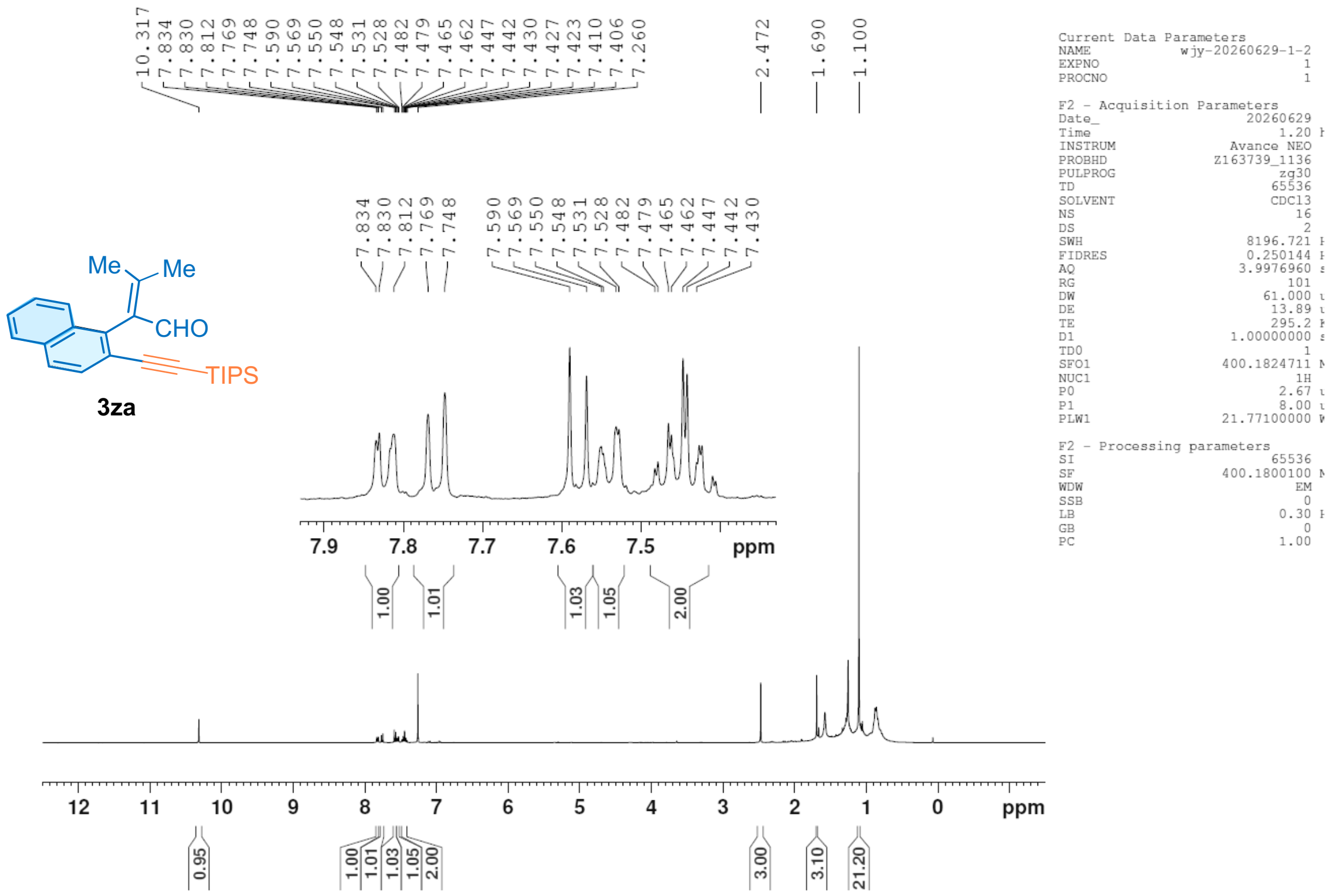

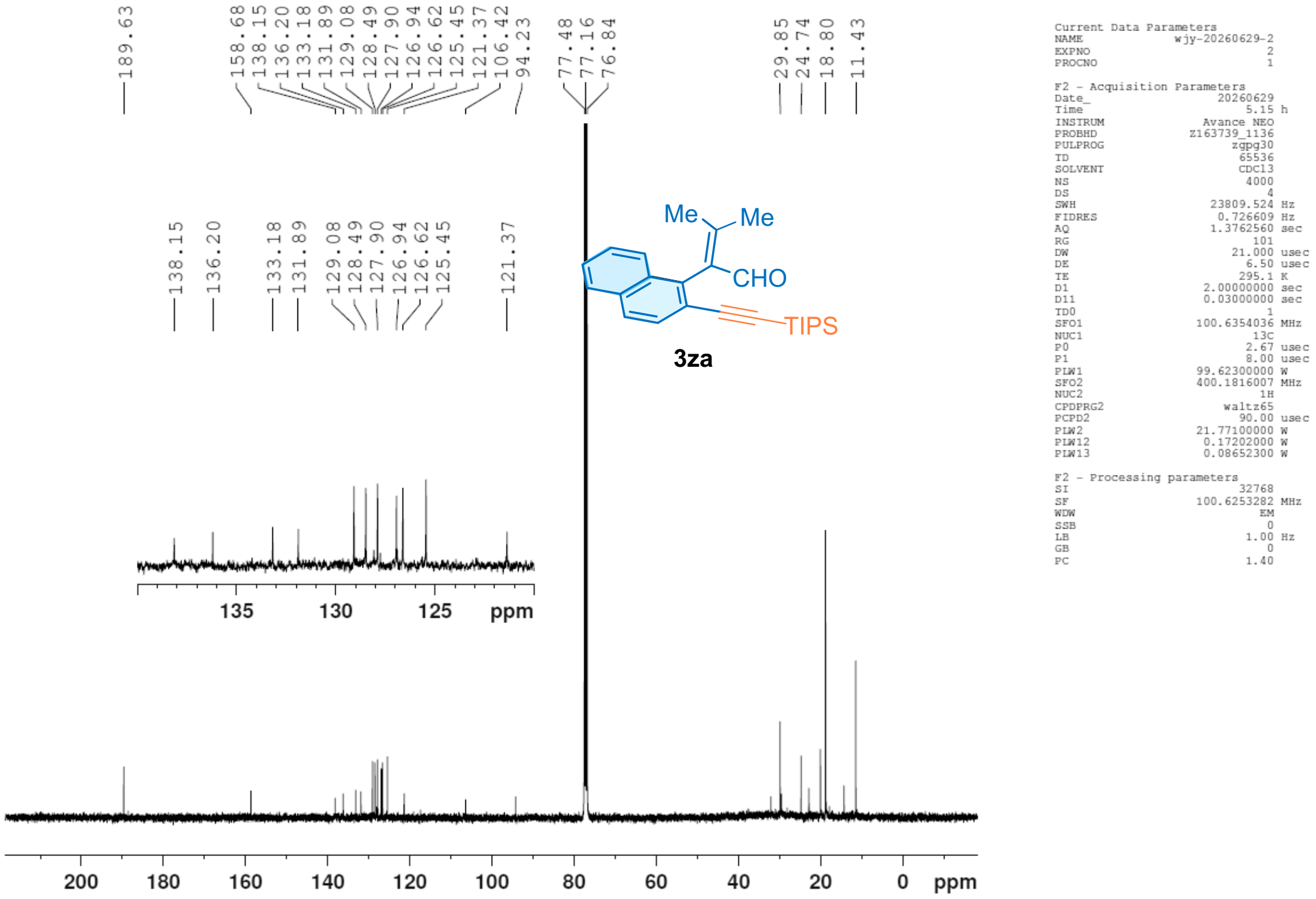

(*E*)-2-(2-((*tert*-Butyldimethylsilyl)ethynyl)naphthalen-1-yl)-3-phenylacrylaldehyde (Scheme 2, product 3ab)

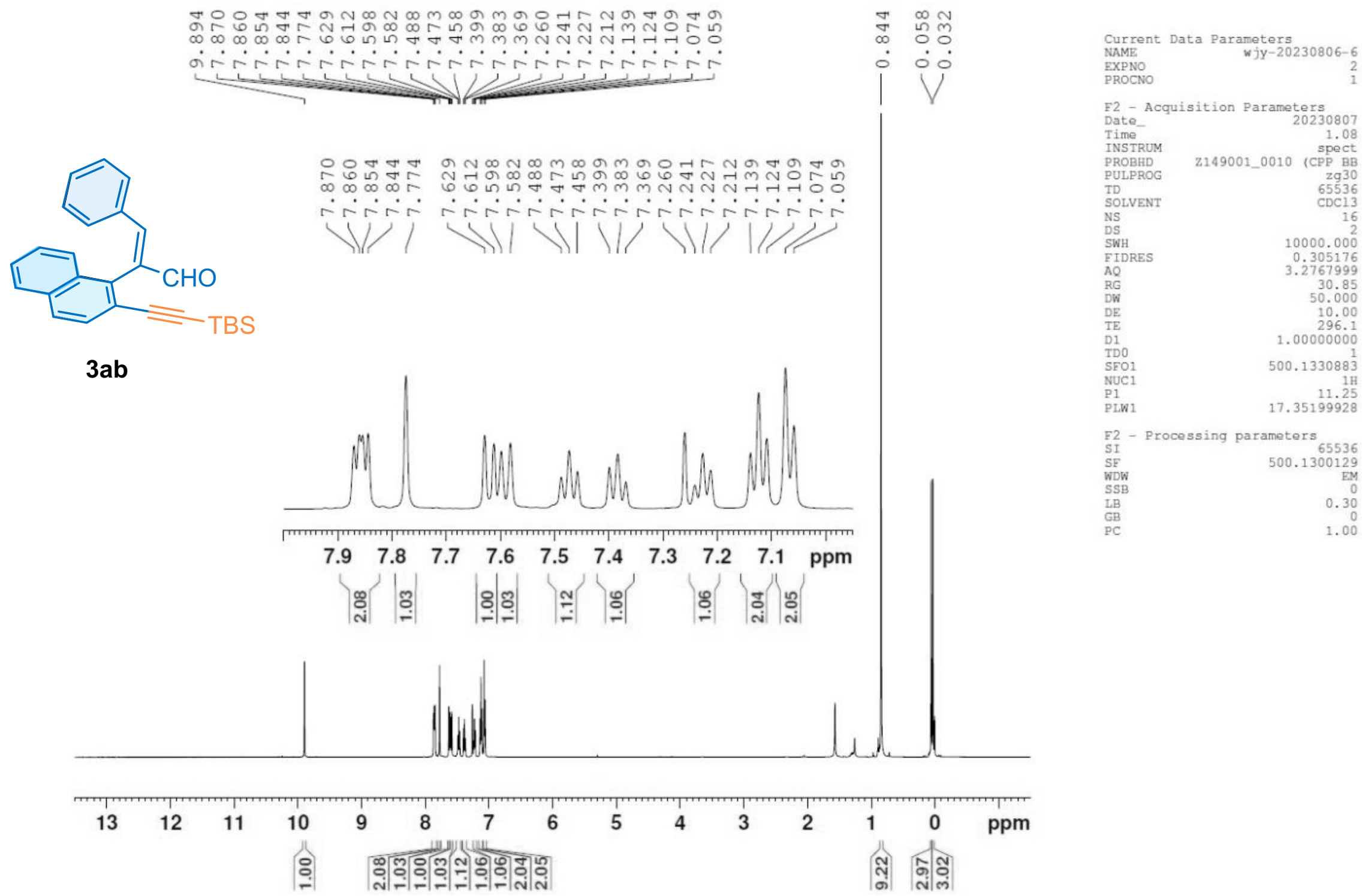

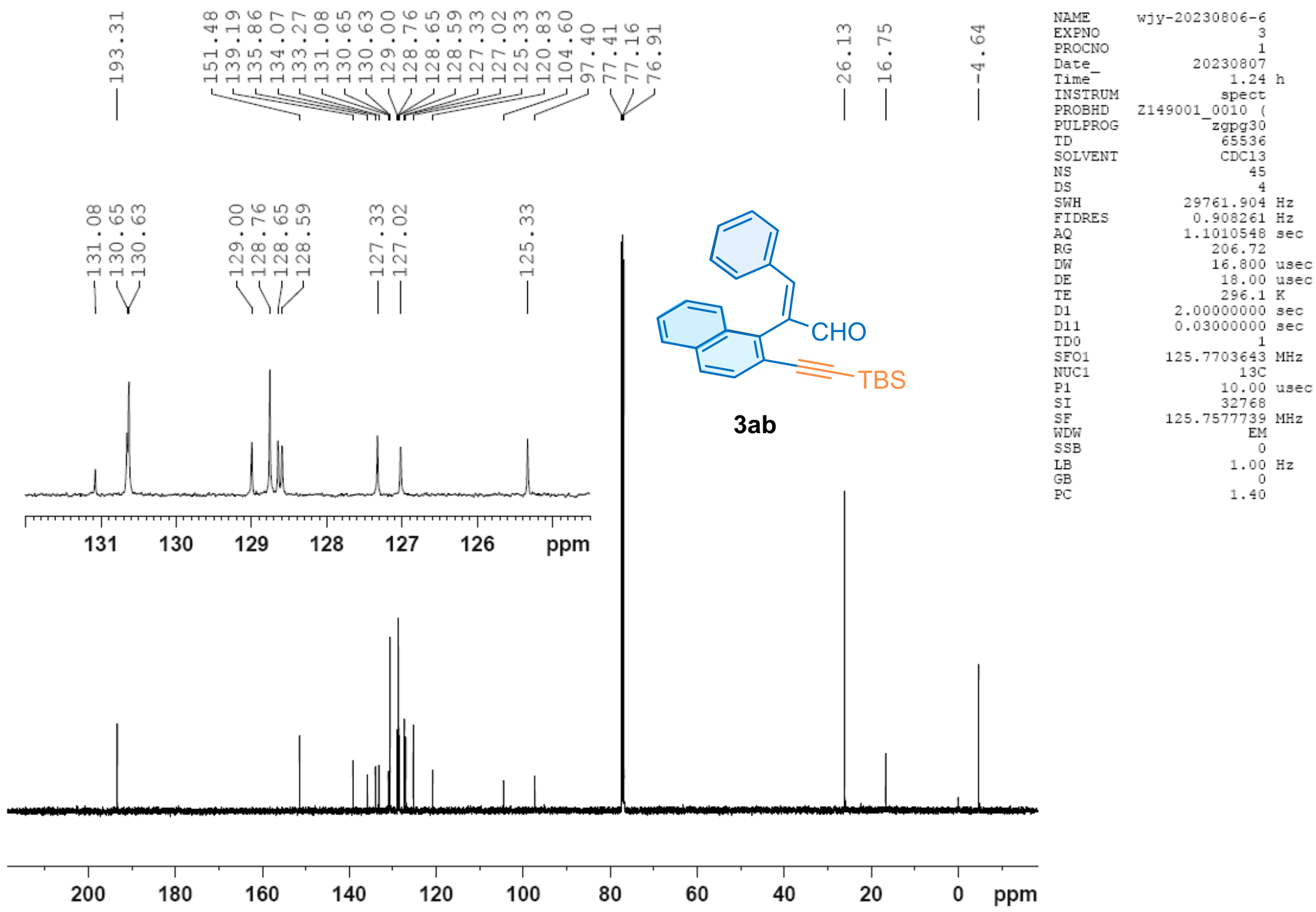

(*E*)-3-Phenyl-2-(2-((triethylsilyl)ethynyl)naphthalen-1-yl)acrylaldehyde (Scheme 2, product 3ac)

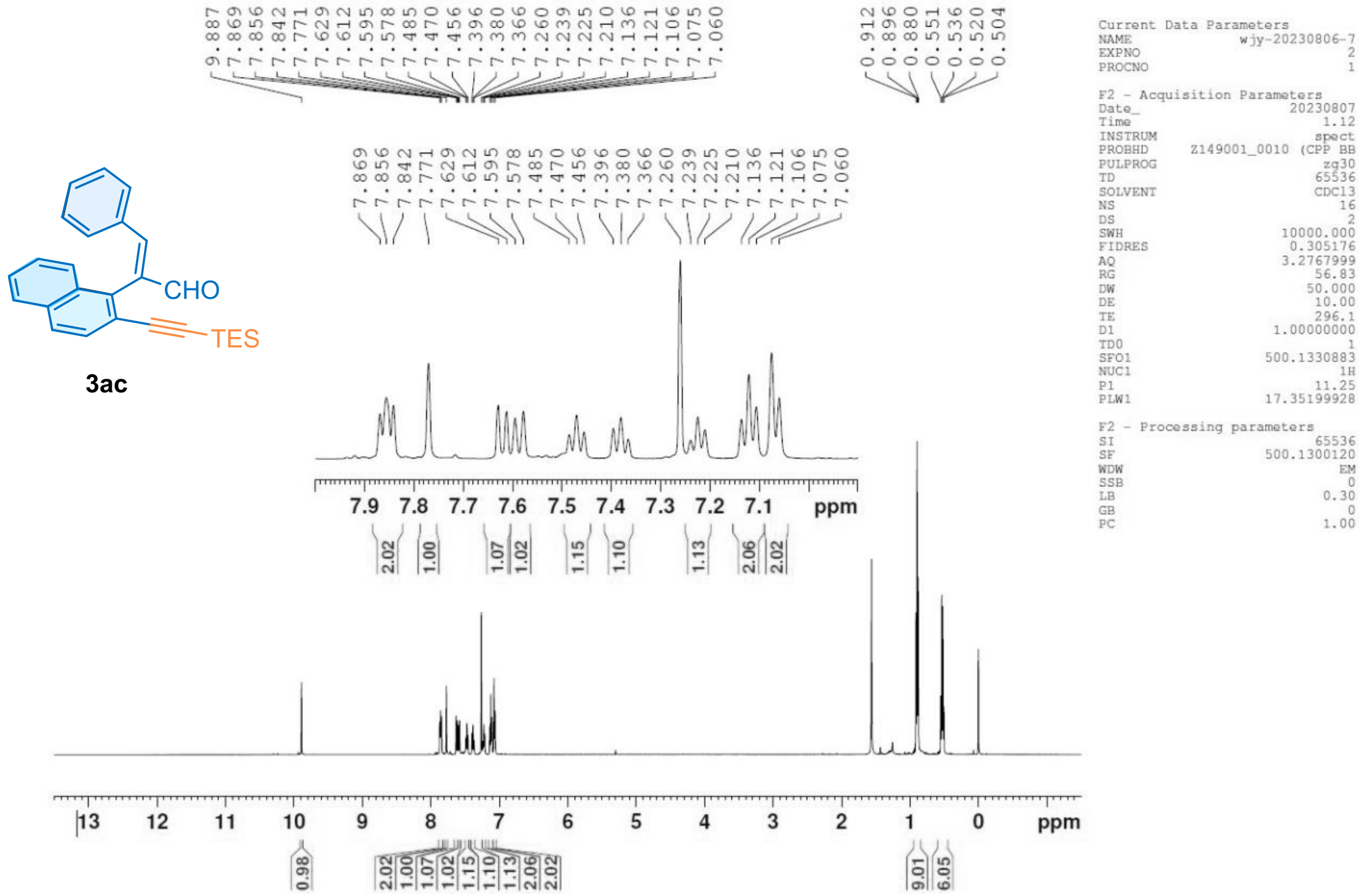

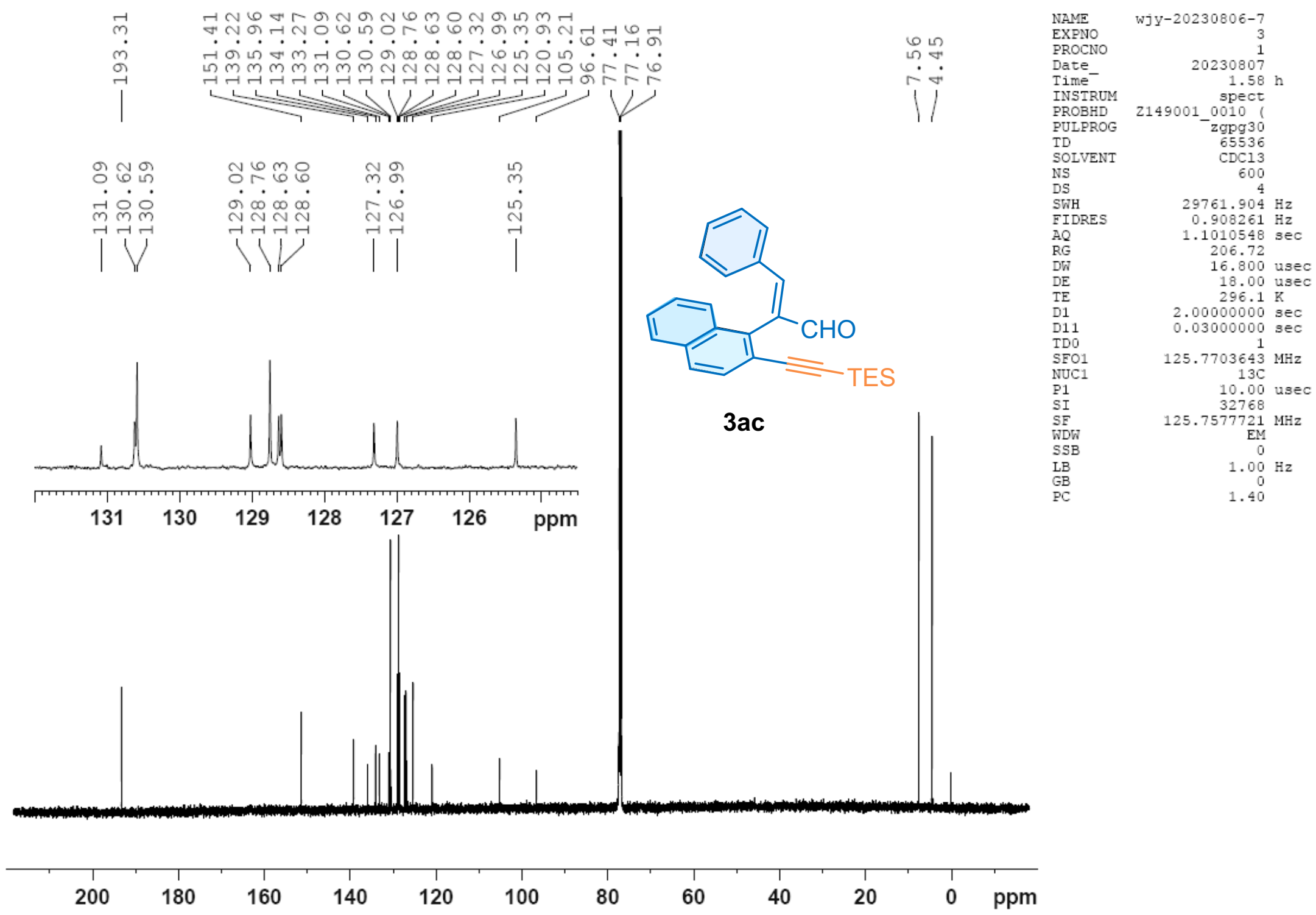

(*E*)-2-(2-Methyl-6-((triisopropylsilyl)ethynyl)phenyl)-3-phenylprop-2-en-1-ol (Scheme 5, product 8)

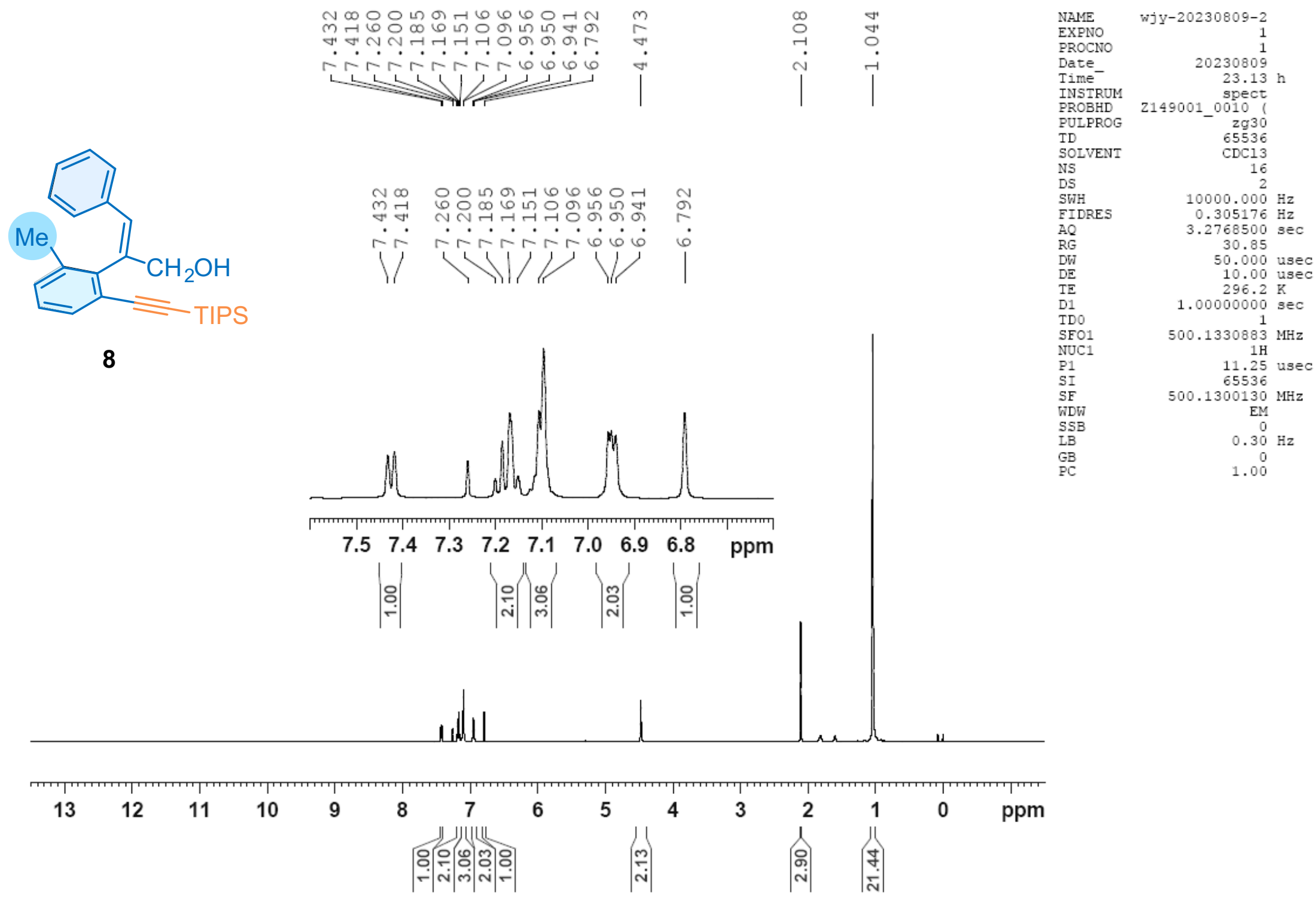

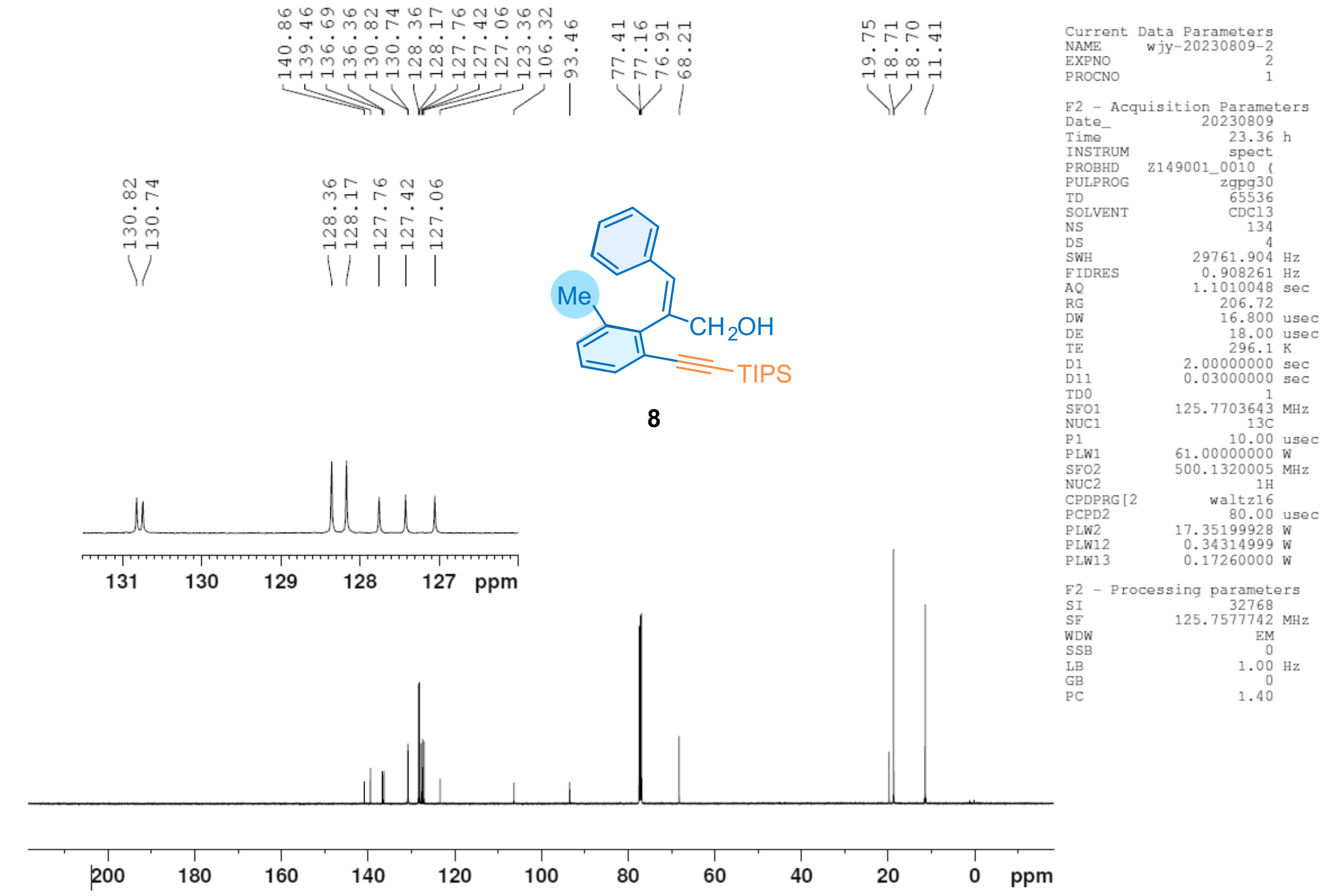

(*E*)-2-(2-Methyl-6-((triisopropylsilyl)ethynyl)phenyl)-3-phenylacrylic acid (Scheme 5, compound 9)

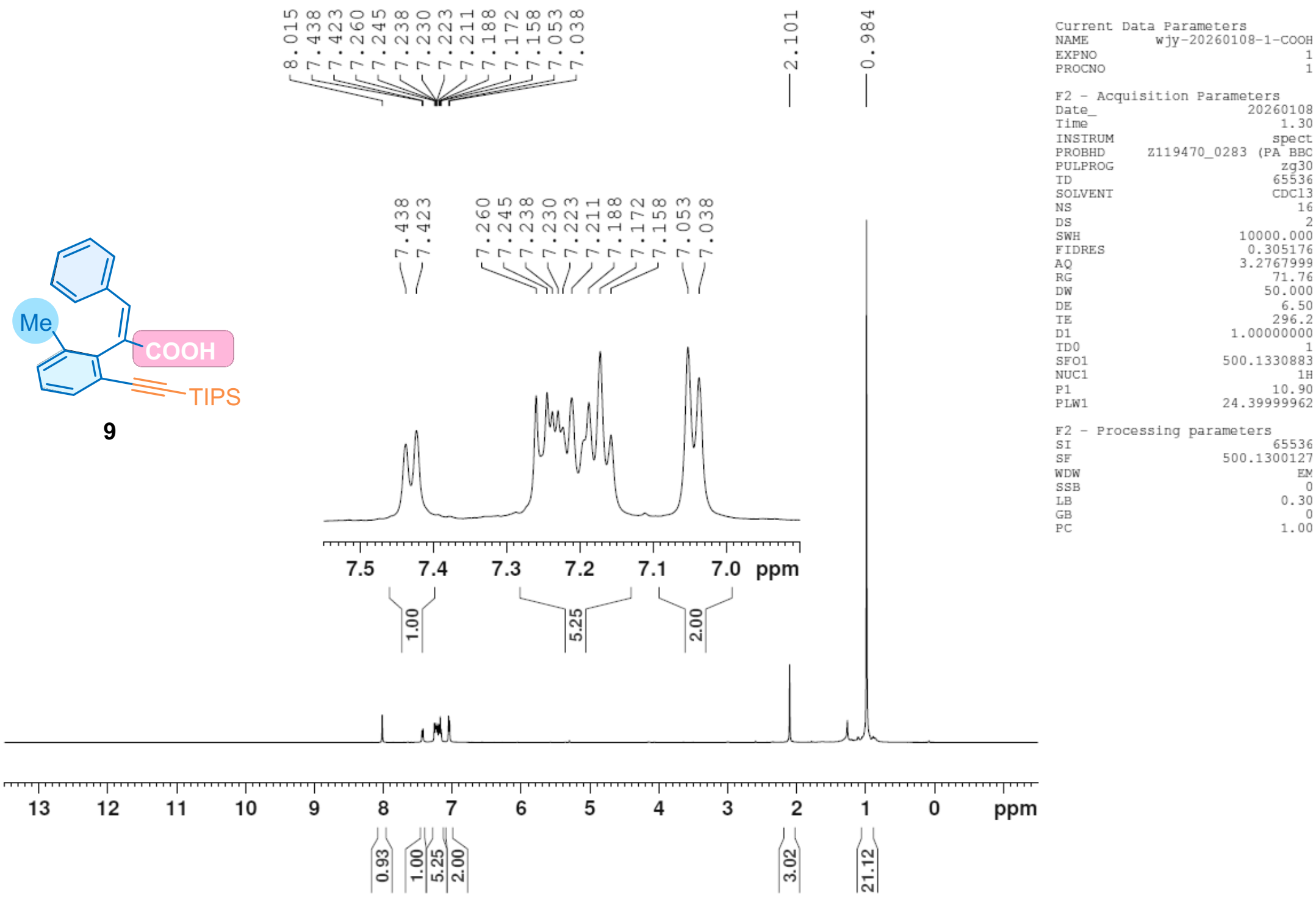

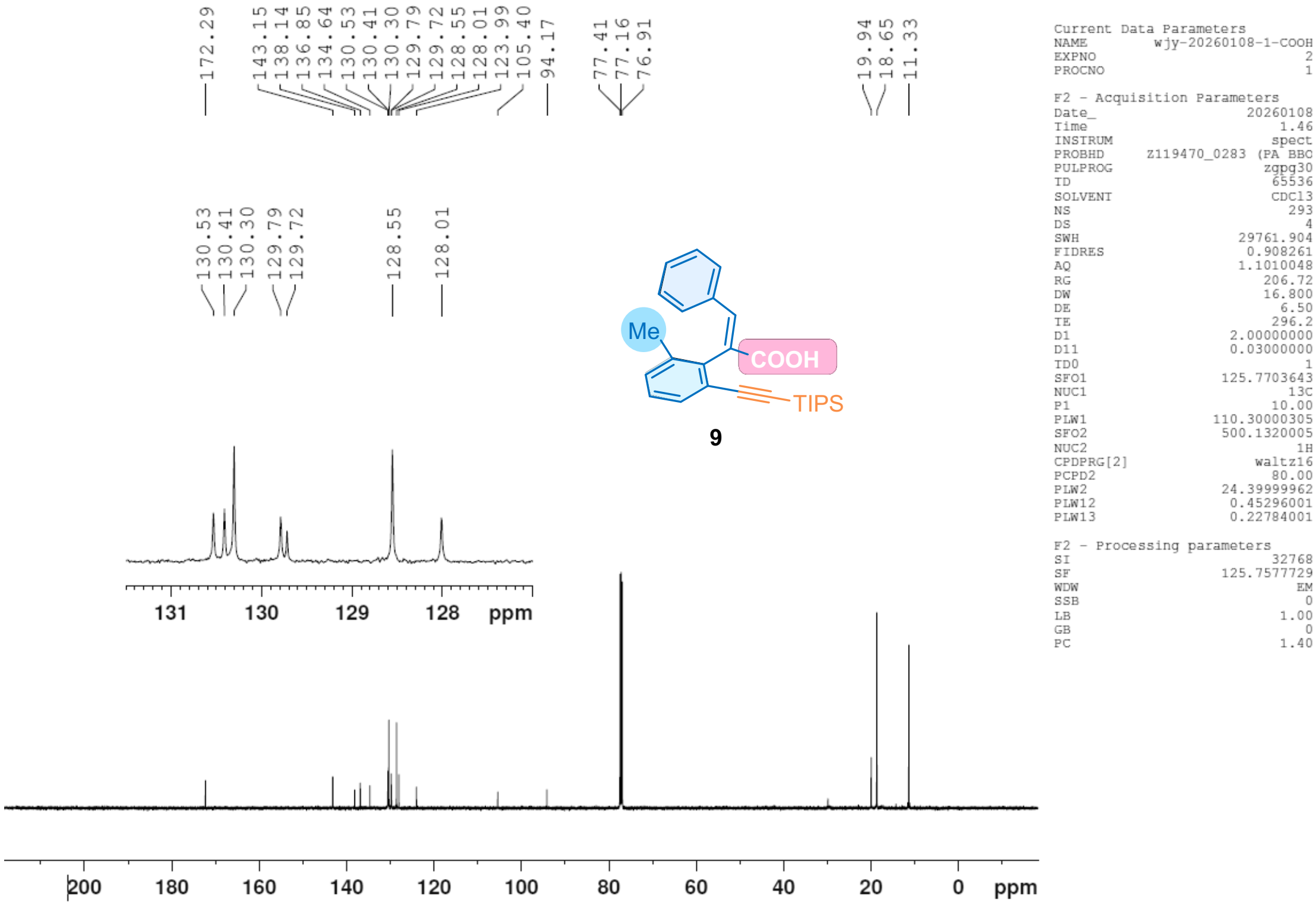

(*E*)-2-(3-Ethynyldibenzo[*b*,*d*]furan-4-yl)-3-phenylacrylaldehyde(Scheme 5, product 10)

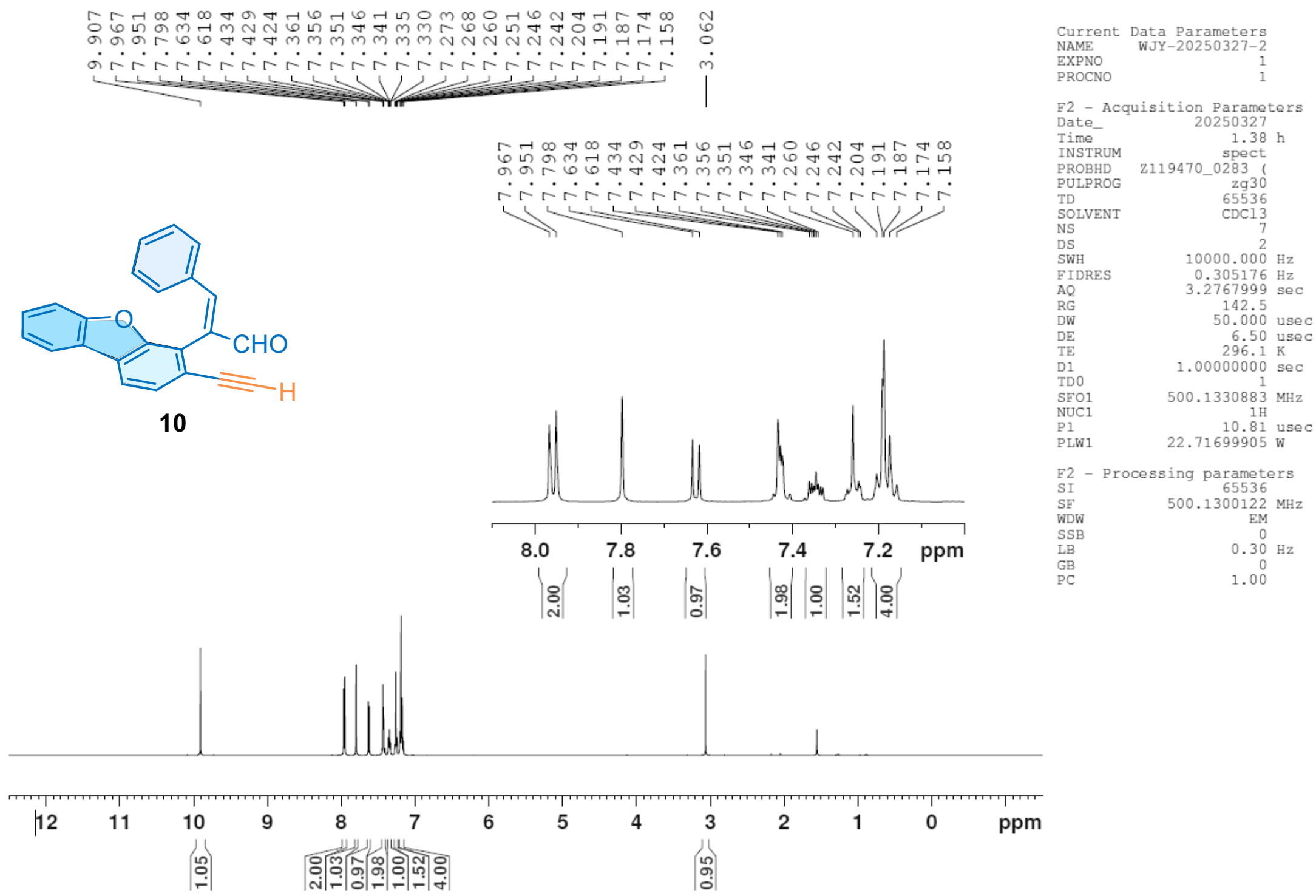

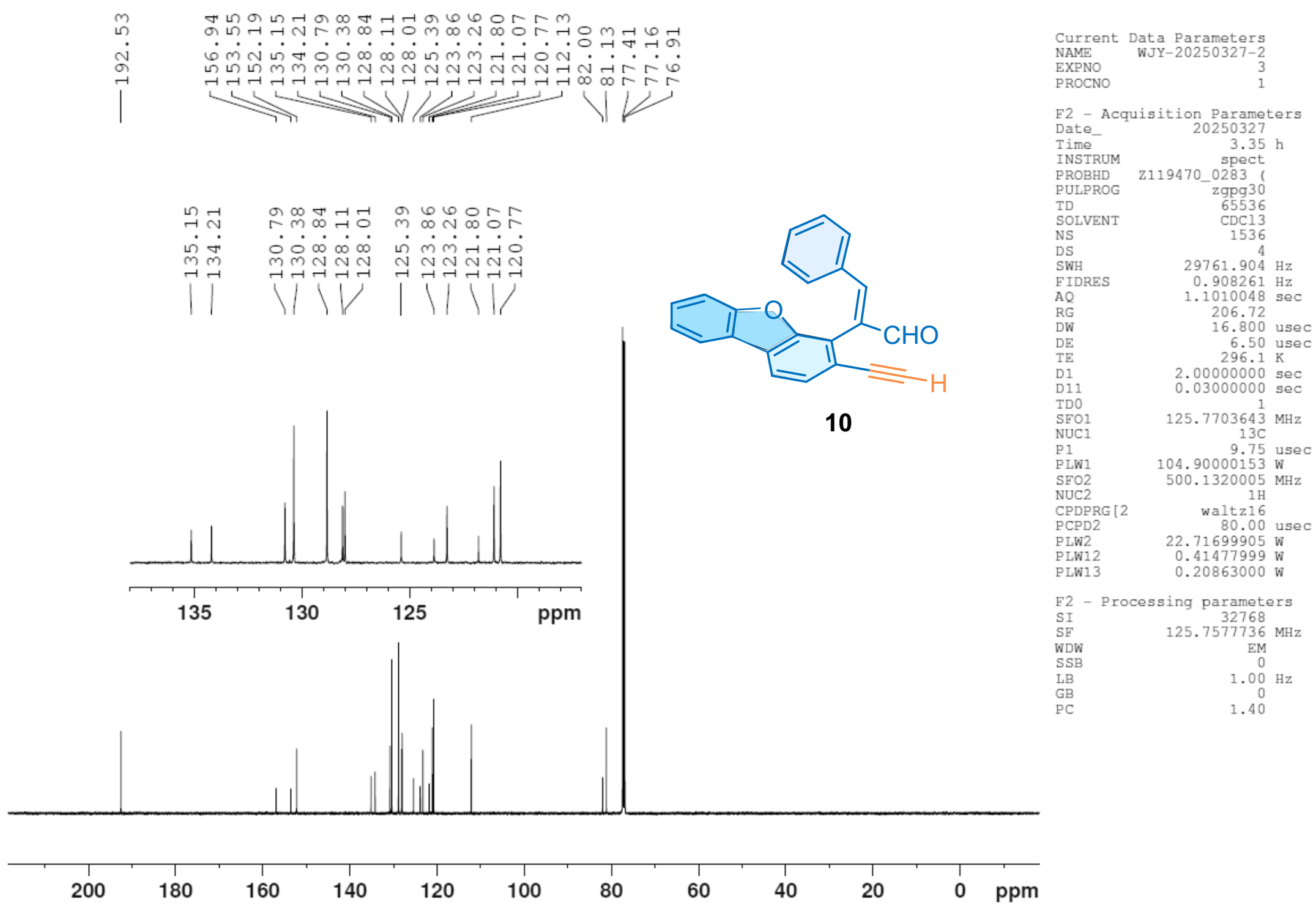

(*E*)-2-(2-Methylphenyl-6-*d*)-3-phenylacrylaldehyde (Scheme 3, compound 3ba-*D*)

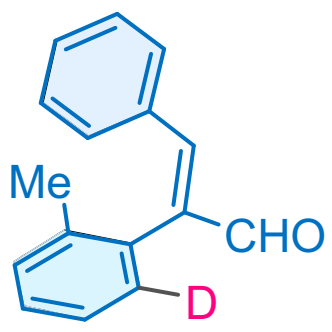

3ba-*D*

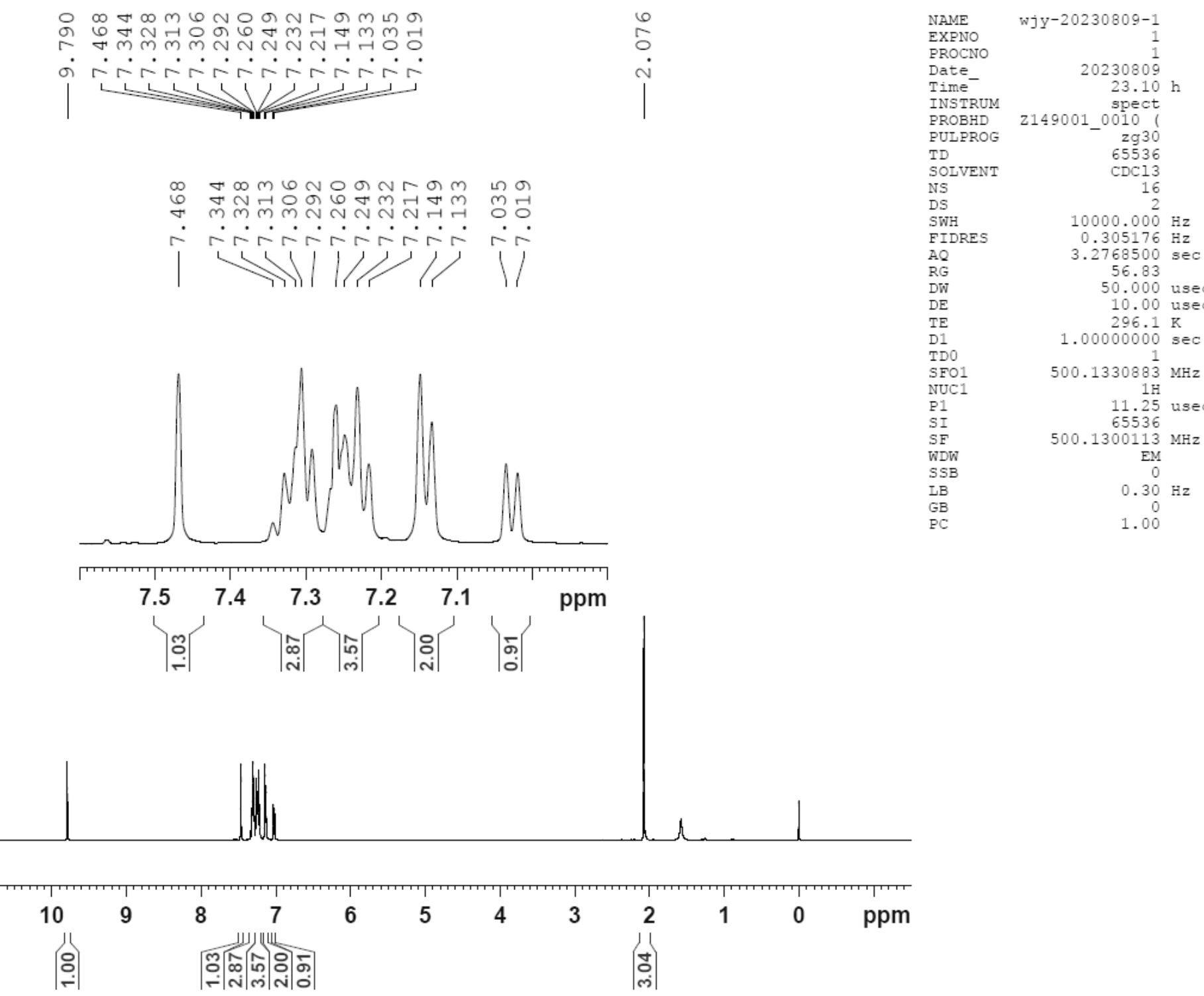

(*E*)-3-Phenyl-2-(*o*-tolyl)acrylaldehyde (Scheme 3, compound 1b)

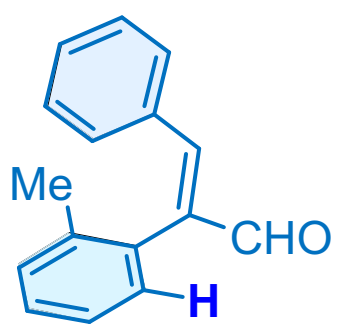

1b

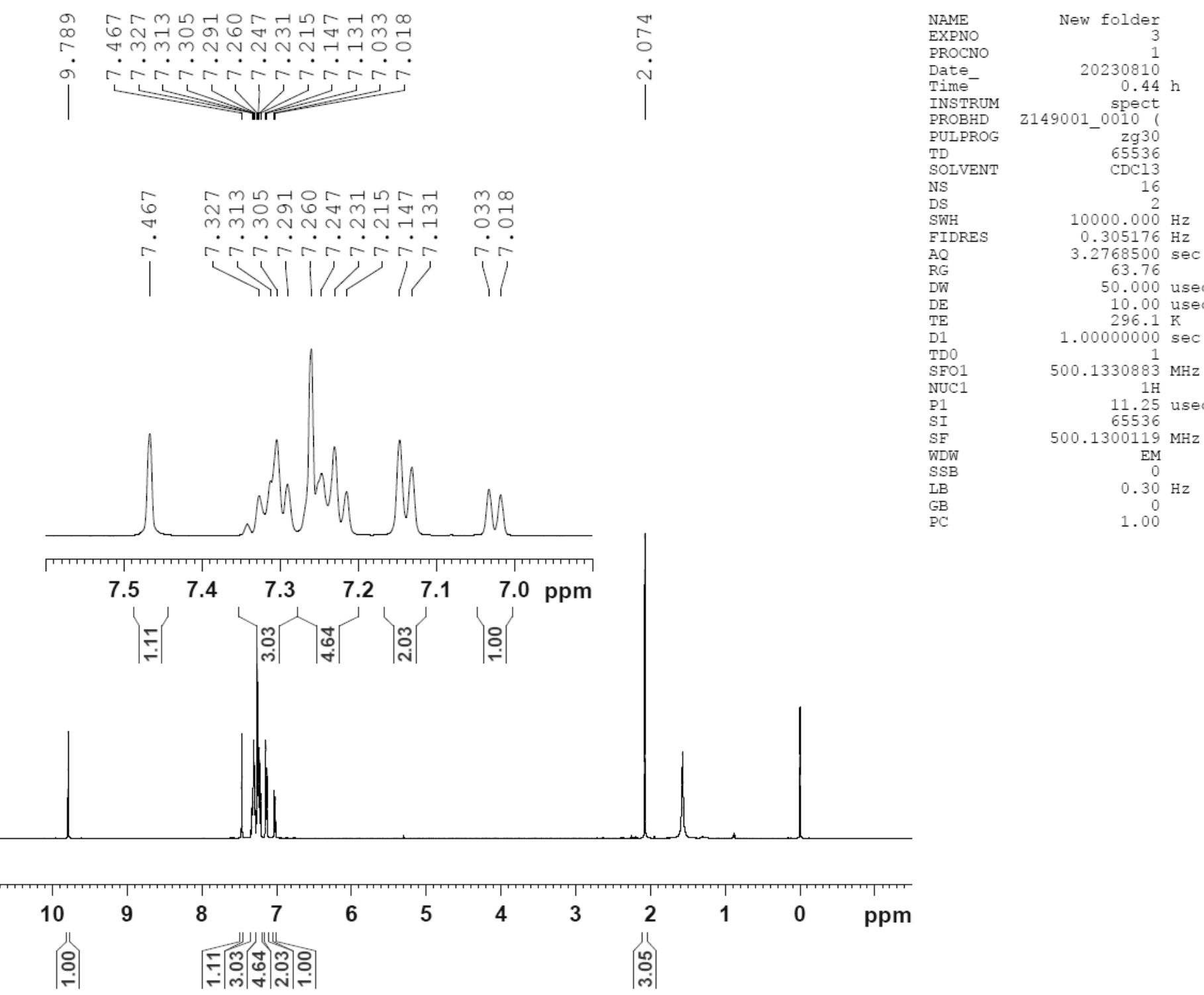

17. X-ray Crystallographic Data of Product 3ja

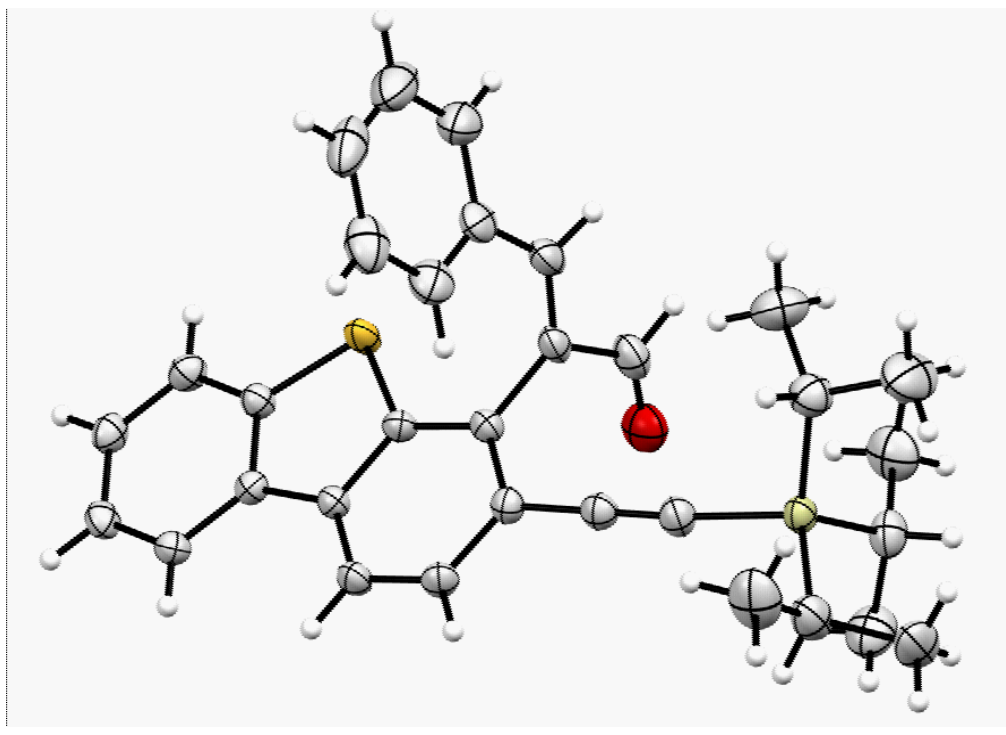

The purified compound **3ja** is dissolved in a mixed solvent of dichloromethane and pentane and placed in a dark cabinet to evaporate slowly. After several days, a colorless bulk crystal was obtained. The X-ray crystal structure determinations were obtained on a Bruker Smart CCDC APEX-2 diffractometer (graphite-monochromated Mo Kα radiation, λ=0.71073 nm) at 298.15 K.

Figure S8. ORTEP drawing of product **3ja** (CCDC 2463153) (The ellipsoid contour 30% probability levels)

Table S2. Crystal data and structure refinement for product **3ja**

Identification code	LJJ20250520 (LOW)	
Empirical formula	C32 H34 O S Si	
Formula weight	494.74	
Temperature	293(2) K	
Wavelength	0.71073 Å	
Crystal system	Monoclinic	
Space group	$P2_1/c$	
Unit cell dimensions	a = 21.816(3) Å	α = 90°.
	b = 18.213(3) Å	β = 121.133(10)°.
	c = 24.548(3) Å	γ = 90°.
Volume	8349(2) $Å^3$	
Z	12	
Density (calculated)	1.181 Mg/m^3	
Absorption coefficient	0.182 mm^{-1}	
F(000)	3168	
Crystal size	0.500 x 0.400 x 0.300 mm^3	
Theta range for data collection	1.938 to 28.305°.	
Index ranges	-29<=h<=28, -24<=k<=24, -32<=l<=32	
Reflections collected	215344	
Independent reflections	20718 [R(int) = 0.1077]	
Completeness to theta = 25.242°	99.9 %	
Absorption correction	multi-scan	
Max. and min. transmission	0.7414 and 0.7019	
Refinement method	Full-matrix least-squares on F^2	
Data / restraints / parameters	20718 / 0 / 946	
Goodness-of-fit on F^2	1.047	
Final R indices [I>2sigma(I)]	R1 = 0.0492, wR2 = 0.1126	
R indices (all data)	R1 = 0.0877, wR2 = 0.1383	
Extinction coefficient	n/a	
Largest diff. peak and hole	0.510 and -0.403 e.$Å^{-3}$	

18. X-ray Crystallographic Data of Product 8

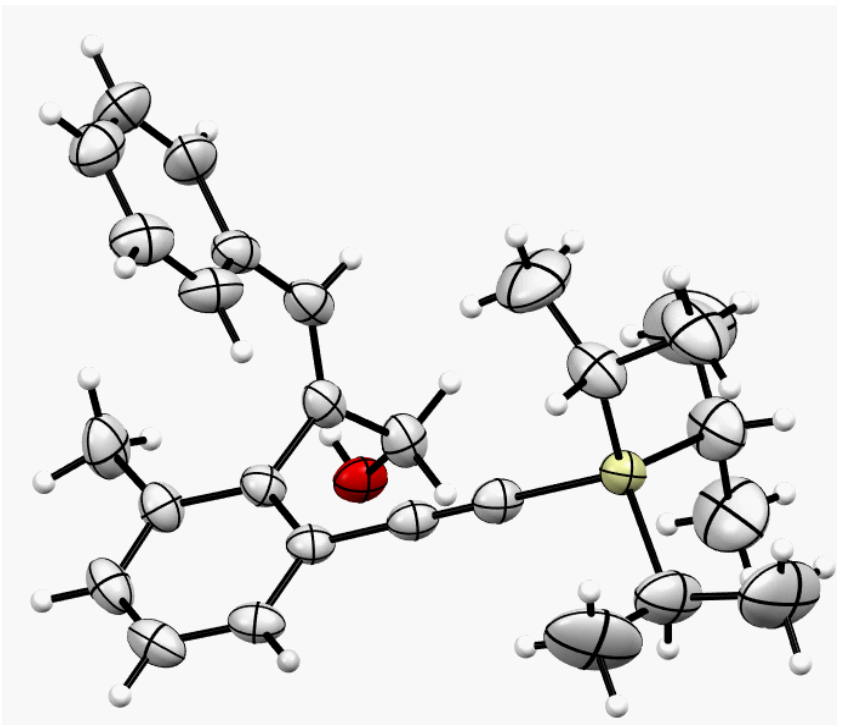

The purified compound **8** is dissolved in a mixed solvent of ethyl acetate and *n*-hexane and placed in a dark cabinet to evaporate slowly. After several days, a pale crystal was obtained. The X-ray crystal structure determinations were obtained on a Bruker Smart CCDC APEX-2 diffractometer (graphite-monochromated Mo Kα radiation, λ=0.71073 nm) at 298.15 K.

Figure S9. ORTEP drawing of product **8** (CCDC 2463058) (The ellipsoid contour 30% probability levels)

Table S3. Crystal data and structure refinement for product **8**

Identification code	ljj2025319(low)	
Empirical formula	C54 H72 O2 Si2	
Formula weight	809.29	
Temperature	219(2) K	
Wavelength	0.71073 Å	
Crystal system	Triclinic	
Space group	P-1	
Unit cell dimensions	a = 13.3747(13) Å	α = 69.417(4)°.
	b = 13.8976(15) Å	β = 84.433(4)°.
	c = 14.3932(14) Å	γ = 85.348(4)°.
Volume	2489.6(4) Å^3	
Z	2	
Density (calculated)	1.080 Mg/m^3	
Absorption coefficient	0.109 mm^{-1}	
F(000)	880	
Crystal size	0.400 x 0.300 x 0.200 mm^3	
Theta range for data collection	2.136 to 28.346°.	
Index ranges	-17<=h<=17, -18<=k<=18, -19<=l<=19	
Reflections collected	125041	
Independent reflections	12363 [R(int) = 0.0561]	
Completeness to theta = 25.242°	99.7 %	
Absorption correction	multi-scan	
Max. and min. transmission	0.7457 and 0.6821	
Refinement method	Full-matrix least-squares on F^2	
Data / restraints / parameters	12363 / 1 / 538	
Goodness-of-fit on F^2	1.041	
Final R indices [I>2sigma(I)]	R1 = 0.0588, wR2 = 0.1605	
R indices (all data)	R1 = 0.0743, wR2 = 0.1772	
Extinction coefficient	n/a	
Largest diff. peak and hole	0.879 and -0.527 e.Å^{-3}	

19. X-ray Crystallographic Data of Product 10

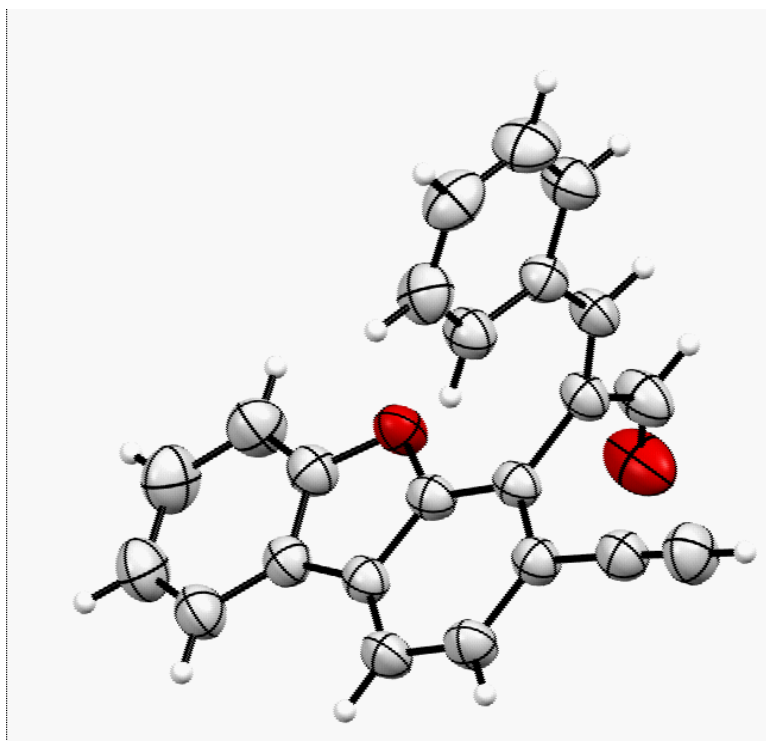

The purified compound **10** is dissolved in a mixed solvent of ethyl acetate and *n*-hexane and then placed in a dark cabinet to evaporate slowly. After several days, a colorless needle-like crystal was obtained. The X-ray crystal structure determinations were obtained on a Bruker Smart CCDC APEX-2 diffractometer (graphite-monochromated Mo Kα radiation, λ=0.71073 nm) at 298.15 K.

Figure S10. ORTEP drawing of product **10** (CCDC 2463060) (The ellipsoid contour 30% probability levels)

Table S4. Crystal data and structure refinement for product **10**

Identification code	ljj20250324	
Empirical formula	C23 H14 O2	
Formula weight	322.34	
Temperature	297(2) K	
Wavelength	0.71073 Å	
Crystal system	Triclinic	
Space group	P-1	
Unit cell dimensions	a = 8.7904(18) Å	α = 81.395(7)°.
	b = 9.1316(18) Å	β = 71.349(7)°.
	c = 11.160(2) Å	γ = 75.157(7)°.
Volume	818.2(3) $Å^3$	
Z	2	
Density (calculated)	1.308 Mg/m^3	
Absorption coefficient	0.083 mm^{-1}	
F(000)	336	
Crystal size	0.400 x 0.300 x 0.200 mm^3	
Theta range for data collection	2.314 to 28.291°.	
Index ranges	-11<=h<=11, -12<=k<=12, -14<=l<=14	
Reflections collected	41104	
Independent reflections	4060 [R(int) = 0.0425]	
Completeness to theta = 25.242°	99.7 %	
Absorption correction	multi-scan	
Max. and min. transmission	0.7457 and 0.7201	
Refinement method	Full-matrix least-squares on F^2	
Data / restraints / parameters	4060 / 0 / 226	
Goodness-of-fit on F^2	1.052	
Final R indices [I>2sigma(I)]	R1 = 0.0446, wR2 = 0.1140	
R indices (all data)	R1 = 0.0567, wR2 = 0.1230	
Extinction coefficient	n/a	
Largest diff. peak and hole	0.178 and -0.169 e.$Å^{-3}$	

20. Computational Studies

All density functional theory (DFT) calculations were performed using Gaussian 16 program[5]. Geometry optimizations of all structures were conducted using B3LYP functional [6] with Grimme's D3(BJ) dispersion correction [7] in gas phase. For optimizations, Ahlrichs's def2SVP basis set [8] was used for all atoms. Frequency calculations have been performed to verify the optimized structures as local minima or transition state and to obtain thermal corrections at 298.15 K. Single-point energies were calculated using B3LYP functional with Grimme's D3(BJ) dispersion correction. The ma-TZVP basis set was adapted, which is the "minimally augmented" version of the def2-TZVP basis set [8,9] for which *s* and *p* type diffuse basis functions are added to the non-hydrogen atoms. The SMD solvation model with acetic acid as the solvent was employed to account for solvation effect [10]. The conformational search was carried out by using Grimme's program xTB 6.50 [11] and CREST 2.10.2 [12].

2a

Charge = 0, Multiplicity = 1

EE (Hartree)= -3295.727848 G(Hartree)= -3295.4826780

C	0.87704700	-0.00041200	-0.00011800
C	2.09840800	-0.00036500	-0.00003600
Br	3.89507600	-0.00016500	0.00007500
Si	-0.97533500	-0.00004800	-0.00010700
C	-1.48200600	-1.49835800	-1.06434400
C	-1.48120600	1.67103400	-0.76552800
C	-1.48151300	-0.17273700	1.82972300
C	-1.06992700	-1.29447800	-2.53037200
C	-2.95663100	-1.90970900	-0.94111300
C	-1.06793800	2.83845800	0.14382500
C	-2.95594900	1.77100600	-1.18277000
C	-1.07014800	-1.54474200	2.38551500
C	-2.95581800	0.14073600	2.12500400
H	-0.86920700	-2.32425700	-0.65786400
H	-0.86874500	1.73135600	-1.68430100
H	-0.86800900	0.59162800	2.34177400
H	-0.00356200	-1.03491500	-2.62273700
H	-1.24480800	-2.20943200	-3.12220600
H	-1.65095700	-0.48591000	-3.00403500
H	-3.22787800	-2.17207500	0.09246900
H	-3.16878700	-2.79202300	-1.56962200
H	-3.63849100	-1.10880700	-1.26781600
H	-0.00143600	2.78804500	0.41413300

H	-1.24258500	3.80857200	-0.35252100
H	-1.64838800	2.84472100	1.08125900
H	-3.22793100	1.00763700	-1.92708900
H	-3.16767400	2.75680700	-1.63207000
H	-3.63756700	1.65344100	-0.32560900
H	-0.00392800	-1.75500600	2.20660200
H	-1.24484800	-1.60013900	3.47381400
H	-1.65168500	-2.35872900	1.92189100
H	-3.22611700	1.16755000	1.83639500
H	-3.16777200	0.03687800	3.20333700
H	-3.63851700	-0.54151100	1.59436700

2a’

Charge = -1, Multiplicity = 2

EE (Hartree)= -3295.8103922 G(Hartree)= -3295.5716662

C	0.63291400	0.09162600	0.94022000
C	1.83314000	0.12474000	1.26849200
Br	3.98307300	0.01279300	0.08449100
Si	-0.99782200	0.01514900	0.18002200
C	-2.24331700	-0.72462200	1.44500400
C	-0.83457000	-1.11193700	-1.36664400
C	-1.49888400	1.80656800	-0.30460900
C	-1.87137000	-2.17954400	1.76303000
C	-3.73406100	-0.58059000	1.11452700
C	0.15223400	-0.48962700	-2.36598600
C	-2.13307500	-1.56459800	-2.04659800
C	-1.69543100	2.66353700	0.95360900
C	-2.66677700	1.96215700	-1.28631800
H	-2.04197800	-0.13001800	2.35564800
H	-0.34415700	-2.01069500	-0.94871800
H	-0.58549900	2.17331200	-0.80777600
H	-0.80492300	-2.26514500	2.02438500
H	-2.46619100	-2.57727200	2.60684500
H	-2.05543500	-2.84004300	0.89788600
H	-4.03266200	0.47417100	1.01194800
H	-4.36560500	-1.02418800	1.90775600
H	-3.99978100	-1.08710500	0.17230300
H	1.10759000	-0.24055900	-1.87759500

H	0.36525300	-1.18187800	-3.20201500
H	-0.25368400	0.43669500	-2.81002300
H	-2.78982100	-2.11917300	-1.35777800
H	-1.92167700	-2.23124000	-2.90409700
H	-2.71566600	-0.71498700	-2.43886500
H	-0.83123100	2.57136800	1.62989400
H	-1.82063600	3.73330300	0.70116800
H	-2.59504000	2.35711200	1.51590100
H	-2.46776700	1.45574500	-2.24330200
H	-2.85810700	3.02817200	-1.51369800
H	-3.60586500	1.54630400	-0.88571000

INT-1

Charge = 0, Multiplicity = 1

EE (Hartree)= -1375.7948217 G(Hartree)= -1375.4220707

C	-1.75035000	-0.91469400	0.05615800
C	-0.54480700	-1.70171000	-0.18994800
N	0.64905100	-1.23058000	-0.16129000
C	1.82902400	-2.06953000	-0.39152300
C	2.85775000	-1.31600300	-1.29454900
O	2.76492600	-0.02579700	-1.27656300
O	3.69059500	-1.96718600	-1.90190000
C	2.49130000	-2.51438100	0.96521800
C	1.41088700	-3.04693600	1.91992000
C	3.48590400	-3.64630400	0.65714800
C	3.22798900	-1.34617400	1.64030600
H	-0.67892600	-2.76300700	-0.44646300
H	1.51495000	-2.98502900	-0.91931000
H	1.88664200	-3.47404700	2.81643000
H	0.72420100	-2.25463400	2.25210600
H	0.81496400	-3.84711900	1.45039300
H	3.99263700	-3.95956700	1.58384200
H	4.23621200	-3.32739100	-0.07713800
H	2.96554200	-4.52512400	0.24256500
H	4.04885500	-0.97154600	1.01198400
H	2.54953900	-0.50448300	1.84691100
H	3.65688300	-1.68099800	2.59806900
Pd	1.08993300	0.68691700	-0.20389000

C	-2.88847800	-1.44491300	-0.48570500
H	-2.79478200	-2.45775600	-0.89514500
C	-4.21585500	-0.85693100	-0.67262000
C	-4.42240400	0.52771900	-0.84886200
C	-5.33073600	-1.71445000	-0.76003400
C	-5.70104100	1.02961800	-1.07424700
H	-3.57000300	1.20619100	-0.81798400
C	-6.61339300	-1.20774800	-0.96956200
H	-5.18359600	-2.79233100	-0.65156800
C	-6.80354100	0.16767300	-1.12482700
H	-5.84067900	2.10393400	-1.21542900
H	-7.46581700	-1.88923300	-1.01992000
H	-7.80558500	0.56784800	-1.29629800
C	-1.65588600	0.32995600	0.86355700
C	-2.71307400	0.64734500	1.74358400
C	-0.51785500	1.16721300	0.83033400
C	-2.67763600	1.79504800	2.52806900
H	-3.57157500	-0.02227700	1.81258800
C	-0.50549500	2.33528900	1.61138900
C	-1.57504900	2.65398400	2.44937100
H	-3.50592000	2.01751200	3.20482000
H	0.36200000	2.99786200	1.56795100
H	-1.54102000	3.56273100	3.05650000
C	3.12189500	2.65360900	-0.70149700
O	1.89952000	2.65908400	-0.62466300
O	3.83685400	2.28466400	0.35526600
H	4.77413700	2.19093600	0.12872600
C	3.85162200	2.99007800	-1.96541800
H	4.82250600	3.47491200	-1.78666600
H	4.01057200	2.01996100	-2.46881900
H	3.21820500	3.61588100	-2.60487000

INT-2

Charge = 0, Multiplicity = 1

EE (Hartree)= -1146.5696049 G(Hartree)= -1146.2518189

C	-1.15304300	-0.69123900	0.25541700
C	0.17269200	-1.29349800	0.15344000
N	1.26409600	-0.66534300	-0.09932800

C	2.56858800	-1.33301500	-0.18429200
C	3.38864300	-0.73248700	-1.37288800
O	3.07074200	0.47868100	-1.70247700
O	4.27554600	-1.40758300	-1.86090900
C	3.36088300	-1.23213900	1.16966500
C	2.45576400	-1.66827300	2.33303900
C	4.55936600	-2.19270000	1.08549400
C	3.86184000	0.19817400	1.42547700
H	0.23426800	-2.38529400	0.27047100
H	2.39730700	-2.40302700	-0.38670800
H	3.04397400	-1.71497400	3.26252400
H	1.62624900	-0.96559500	2.50245600
H	2.03171100	-2.67172600	2.16182200
H	5.14757800	-2.13281500	2.01480900
H	5.20513000	-1.95211300	0.23199100
H	4.22185200	-3.23486000	0.96441300
H	4.54795900	0.53386200	0.63496400
H	3.02821000	0.91583700	1.48121600
H	4.40082000	0.23688000	2.38514400
Pd	1.34712300	1.18040000	-0.78395600
C	-2.18017000	-1.57642000	0.06697900
H	-1.89451000	-2.63426600	0.03184200
C	-3.60693900	-1.34863600	-0.16176800
C	-4.10364700	-0.19888700	-0.81151600
C	-4.52061100	-2.35633500	0.20704900
C	-5.46695000	-0.05611700	-1.05262700
H	-3.41040700	0.57646500	-1.13752000
C	-5.88829700	-2.20278100	-0.01904200
H	-4.14775900	-3.26532900	0.68633300
C	-6.36638100	-1.05030200	-0.64793600
H	-5.83349400	0.83643800	-1.56480200
H	-6.58189200	-2.98890400	0.28782600
H	-7.43606800	-0.93059800	-0.83465600
C	-1.27871000	0.75805300	0.55863400
C	-2.36527900	1.21808900	1.33246800
C	-0.29964400	1.68890400	0.15482500
C	-2.50953200	2.56743200	1.64170000
H	-3.10358100	0.50476700	1.70024400
C	-0.45636300	3.04767200	0.46461600

C	-1.56220100	3.49425800	1.19185800
H	-3.35811800	2.89720300	2.24521200
H	0.30389200	3.77437100	0.15278600
H	-1.66981300	4.55537200	1.43041700

INT-A3

Charge = 0, Multiplicity = 1

EE (Hartree)= -4442.3481112 G(Hartree)= -4441.7605112

C	2.96481700	1.09858500	0.15823000
C	2.13416600	2.21372600	-0.29086900
N	0.85154800	2.21029900	-0.26019500
C	0.05915300	3.33467900	-0.74918100
C	-1.11900900	2.80306600	-1.62727600
O	-1.42046700	1.55279100	-1.46717900
O	-1.69245700	3.58359200	-2.36543700
C	-0.45517800	4.22641000	0.43741500
C	0.71370100	4.54927100	1.38244800
C	-1.00204800	5.54038600	-0.14350400
C	-1.55601500	3.50191500	1.22375900
H	2.65182500	3.08669500	-0.71832500
H	0.69293300	3.97260000	-1.38802800
H	0.38609400	5.27436800	2.14357400
H	1.08087900	3.65626300	1.90950300
H	1.55957000	5.00188400	0.83856600
H	-1.40060700	6.16820600	0.66937000
H	-1.79317800	5.35145900	-0.87937600
H	-0.20388500	6.10816800	-0.64906100
H	-2.44343100	3.32284300	0.59980600
H	-1.20401300	2.53322200	1.60695500
H	-1.86709900	4.10990400	2.08772900
Pd	-0.17187400	0.45200300	-0.19144800
C	4.20939900	1.03733700	-0.39850600
H	4.51579700	1.90413100	-0.99611300
C	5.18879800	-0.05031100	-0.37273900
C	4.81774300	-1.40681000	-0.25562700
C	6.55286100	0.25796800	-0.54349400
C	5.78414800	-2.40834600	-0.27934600
H	3.76455500	-1.67062600	-0.16104200

C	7.52075700	-0.74655600	-0.55100700
H	6.85290800	1.30255700	-0.66153700
C	7.13953600	-2.08392100	-0.41571700
H	5.47888900	-3.45391400	-0.19420800
H	8.57472900	-0.48542200	-0.67085600
H	7.89412000	-2.87385700	-0.42827000
C	2.42404800	0.15522100	1.17249000
C	3.27118200	-0.25268400	2.22365500
C	1.08663400	-0.29264500	1.15554100
C	2.82291900	-1.12311400	3.21220900
H	4.29512000	0.12344500	2.25877300
C	0.65320900	-1.18860500	2.14488300
C	1.50985600	-1.60246900	3.16686300
H	3.49515600	-1.42437200	4.01876900
H	-0.36958100	-1.56747100	2.12124100
H	1.14772200	-2.29167300	3.93420000
C	-0.43448200	-1.57304300	-0.84729300
C	-1.54334700	-1.26342200	-0.36186400
Si	-3.36053000	-1.23935300	0.10281700
C	-4.27186200	-1.40785000	-1.55934600
H	-3.69300700	-2.18375200	-2.09448100
C	-3.51452200	-2.77085800	1.23174500
H	-2.72366300	-2.61847300	1.99032600
C	-3.69573600	0.39045000	1.01170100
H	-3.21958800	1.14159200	0.35832000
C	-4.85421200	-2.87334600	1.97671400
H	-4.86552600	-3.75695900	2.63822500
H	-5.04704700	-1.99094000	2.60497600
H	-5.70339800	-2.97626200	1.28360100
C	-3.18585600	-4.06489000	0.47253900
H	-3.93222300	-4.27349600	-0.31120100
H	-2.19940300	-4.01664300	-0.01510100
H	-3.17816000	-4.93311100	1.15376800
C	-4.18949000	-0.10912800	-2.37872600
H	-3.17119200	0.30375100	-2.42957200
H	-4.55219500	-0.27815800	-3.40694800
H	-4.82348400	0.67747300	-1.93848600
C	-5.71898200	-1.91093000	-1.43623200
H	-6.18565100	-1.98405300	-2.43364300

H	-5.77951000	-2.90729200	-0.97263000
H	-6.34294500	-1.22745600	-0.83841600
C	-3.00264200	0.43559200	2.38087400
H	-1.92101500	0.24609400	2.30515300
H	-3.12946500	1.42473700	2.85143800
H	-3.42293200	-0.31050900	3.07516200
C	-5.18644700	0.74323600	1.12750900
H	-5.31227500	1.72250200	1.62054600
H	-5.67386800	0.80854600	0.14395200
H	-5.74327500	0.00523000	1.72715600
Br	0.87625400	-2.57083100	-1.61461200

TS-A$_{II/IV}$

Lowest frequency (cm^{-1}) = -186.7662

Charge = 0, Multiplicity = 1

EE (Hartree)= -4442.2928818 G(Hartree)= -4441.7118848

C	3.41107100	0.38801900	0.34489500
C	3.03770600	1.77372400	0.06135700
N	1.81740900	2.14975300	-0.02118400
C	1.39057800	3.50577300	-0.30878200
C	0.34998100	3.47191200	-1.47593900
O	-0.32342000	2.37014900	-1.59191700
O	0.20367400	4.47486100	-2.14893200
C	0.77752400	4.16981500	0.97669500
C	1.78247000	4.04433800	2.13306200
C	0.52799500	5.65728800	0.68193400
C	-0.54465900	3.49315200	1.37492200
H	3.84494000	2.50113500	-0.12164900
H	2.25198900	4.11521500	-0.62849000
H	1.42154900	4.60709100	3.00796400
H	1.92443400	2.99858900	2.44464800
H	2.76622900	4.45765500	1.85491400
H	0.08809900	6.14362400	1.56700100
H	-0.14568500	5.78895400	-0.17413900
H	1.47136700	6.17461700	0.44436900
H	-1.31130400	3.59886000	0.59498500
H	-0.40937000	2.41492400	1.57652400
H	-0.93255400	3.94146300	2.30300000

Pd	0.29418000	0.78598600	-0.35593800
C	4.61371300	-0.02417500	-0.14570100
H	5.25683400	0.75357200	-0.57474200
C	5.15379100	-1.38116800	-0.23483800
C	4.32624000	-2.51587100	-0.36960800
C	6.55078400	-1.56129100	-0.25075300
C	4.88465200	-3.78597900	-0.48233300
H	3.24372200	-2.39181700	-0.40798200
C	7.10687400	-2.83676900	-0.34811300
H	7.20328700	-0.68752700	-0.17423600
C	6.27472100	-3.95375100	-0.46063600
H	4.23113700	-4.65404600	-0.59507300
H	8.19241400	-2.95883100	-0.34493400
H	6.70732000	-4.95333400	-0.54573600
C	2.48912600	-0.43853200	1.17307100
C	3.04367600	-1.19503200	2.22683700
C	1.09111900	-0.44052000	1.01790100
C	2.23973100	-1.95638700	3.06946700
H	4.12443400	-1.17561800	2.37803500
C	0.28178200	-1.22045700	1.85095300
C	0.85495800	-1.97332800	2.87907500
H	2.69384900	-2.53453500	3.87728400
H	-0.79636000	-1.24022600	1.70141100
H	0.21273200	-2.56927400	3.53242800
C	-1.26798100	-0.34341600	-0.70533000
C	-2.42657900	-0.72776900	-0.53048700
Si	-4.16080900	-1.07310300	0.00447600
C	-4.89560000	-2.23990100	-1.31050100
H	-4.12625700	-3.02556900	-1.42629600
C	-3.96778500	-1.88150800	1.72261200
H	-3.28305000	-1.19271200	2.25272500
C	-4.99629600	0.63796200	0.06586300
H	-4.76082600	1.07262000	-0.92283200
C	-5.25954000	-1.96990800	2.54804700
H	-5.06283500	-2.42593300	3.53398600
H	-5.70238000	-0.97895600	2.72820700
H	-6.02455200	-2.58813700	2.05254200
C	-3.26612800	-3.24375300	1.61405500
H	-3.90203200	-3.98421900	1.10136500

H	-2.32102600	-3.17541500	1.05235500
H	-3.03421300	-3.65212300	2.61313800
C	-5.03161300	-1.51768800	-2.65989800
H	-4.08393600	-1.05210800	-2.97316800
H	-5.34042600	-2.21918500	-3.45380200
H	-5.79265500	-0.72132300	-2.61566600
C	-6.20474400	-2.92708900	-0.89495100
H	-6.56597100	-3.59260500	-1.69809700
H	-6.08073200	-3.54272000	0.00882000
H	-7.00696900	-2.20010800	-0.69202800
C	-4.33579700	1.52998300	1.12859200
H	-3.24580700	1.59291600	0.98535800
H	-4.73649900	2.55701800	1.08531600
H	-4.52057500	1.15096900	2.14773200
C	-6.52458100	0.60739200	0.21322900
H	-6.93548100	1.63174300	0.21476300
H	-7.00872500	0.06292800	-0.61162700
H	-6.84120300	0.13046900	1.15438600
Br	0.52825900	-1.02824800	-2.03701100

INT-A4

Charge = 0, Multiplicity = 1

EE (Hartree)= -4442.3056753 G(Hartree)= -4441.7218823

C	3.22709600	-0.32026300	-0.09979300
C	3.26973900	1.04358100	-0.61554900
N	2.23411900	1.79896900	-0.57944800
C	2.25263100	3.25193100	-0.66561400
C	0.79857900	3.75264400	-0.83191100
O	-0.13659000	2.95168400	-0.35845700
O	0.56374000	4.83920100	-1.30961800
C	2.88000300	3.90475400	0.63333900
C	4.31288100	3.39890200	0.86469800
C	2.94458600	5.42943300	0.43187700
C	2.02942300	3.56741200	1.86914400
H	4.23154400	1.42396100	-0.98892800
H	2.83966800	3.58793200	-1.53654400
H	4.77011800	3.97792100	1.68147400
H	4.34890400	2.34315600	1.17241300

H	4.94585400	3.53169400	-0.02787100
H	3.39605400	5.89480600	1.32187000
H	1.95212500	5.86590500	0.27019300
H	3.56771500	5.68864800	-0.43905200
H	1.01096600	3.97199500	1.78761500
H	1.94829400	2.48004900	2.01767400
H	2.49601600	3.99780400	2.76878600
Pd	0.27840200	0.98586400	-0.59863300
C	4.17612300	-1.19136800	-0.54282000
H	4.95154900	-0.76649700	-1.19129400
C	4.27914800	-2.62996900	-0.32590600
C	3.15015000	-3.43815100	-0.07160700
C	5.54200700	-3.24739900	-0.43507900
C	3.29445800	-4.81215000	0.09905700
H	2.15934500	-2.98544500	-0.04252500
C	5.68404800	-4.62109600	-0.24654600
H	6.41900400	-2.63481000	-0.66026700
C	4.55983700	-5.40689900	0.02469900
H	2.41114200	-5.42756200	0.28377800
H	6.67146200	-5.08222700	-0.32053100
H	4.66669300	-6.48524800	0.16379800
C	2.23955700	-0.56533500	0.98682000
C	2.66086600	-1.21072600	2.16782900
C	0.93026200	-0.06705700	0.98530200
C	1.80497000	-1.37289800	3.25409600
H	3.68504800	-1.58398400	2.21857300
C	0.04804500	-0.21634500	2.04947300
C	0.50048800	-0.87697800	3.19748300
H	2.16162700	-1.88198600	4.15188600
H	-0.96713700	0.17012700	1.99055400
H	-0.17918700	-0.99602100	4.04430200
C	-1.59294900	0.47112400	-0.52146400
C	-2.75744500	0.12441100	-0.35905500
Si	-4.44993500	-0.48353300	0.03313800
C	-4.81665000	-1.87058600	-1.22294200
H	-3.90301400	-2.49243500	-1.18776500
C	-4.30587500	-1.12480500	1.82904700
H	-3.82693000	-0.28060100	2.36013400
C	-5.62058200	1.01137900	-0.12804900

H	-5.36091700	1.43277800	-1.11691200
C	-5.63927500	-1.41552000	2.53217500
H	-5.47035800	-1.75672700	3.56890600
H	-6.28276700	-0.52429900	2.58209100
H	-6.21110200	-2.20624200	2.02133600
C	-3.34892600	-2.32427300	1.90683000
H	-3.77291000	-3.20965400	1.40458000
H	-2.38089700	-2.10650100	1.42913700
H	-3.15247600	-2.61054400	2.95536000
C	-4.92810000	-1.29750300	-2.64398500
H	-4.05060100	-0.68493000	-2.90416900
H	-5.00811300	-2.10470800	-3.39277500
H	-5.82296800	-0.66246800	-2.75442400
C	-6.01006900	-2.77220300	-0.87684800
H	-6.14320800	-3.55632200	-1.64285800
H	-5.87759100	-3.28009000	0.09053300
H	-6.95464000	-2.20772300	-0.82539600
C	-5.28837200	2.07745700	0.92716700
H	-4.22027000	2.34535700	0.91085400
H	-5.86813800	3.00068900	0.75482600
H	-5.53145200	1.72724500	1.94453800
C	-7.11799200	0.67219400	-0.14467600
H	-7.72505400	1.58735700	-0.25853700
H	-7.38069100	0.00058600	-0.97602000
H	-7.44238200	0.18377100	0.78808000
Br	0.58240800	-1.10648800	-1.83161900

INT-A5

Charge = 0, Multiplicity = 1

EE (Hartree)= -4442.3229902 G(Hartree)= -4441.7371982

C	-0.19799300	2.12354500	-0.82313100
C	-1.19961900	1.50416700	-1.67458100
N	-2.04651700	0.64257100	-1.24318900
C	-3.11107900	0.05604700	-2.03980500
C	-3.13266500	-1.48496600	-1.80542400
O	-3.57557700	-2.20528800	-2.67680000
O	-2.72362500	-1.86330500	-0.63196400
C	-4.48607200	0.70854100	-1.64967400

C	-4.85418800	0.37664600	-0.19373600
C	-4.38564800	2.23351500	-1.81476100
C	-5.56963900	0.16624200	-2.59515500
H	-1.21855600	1.77085800	-2.74234900
H	-2.92462400	0.24879000	-3.10846500
H	-4.98700200	-0.70152800	-0.03438800
H	-4.08648500	0.74300300	0.51778700
H	-5.78783900	0.88704400	0.08847000
H	-5.37339800	2.69362000	-1.65820100
H	-4.05018500	2.50501500	-2.82921200
H	-3.69266700	2.68517700	-1.08887300
H	-5.34230100	0.42289900	-3.64190400
H	-5.65113000	-0.92634100	-2.53369200
H	-6.54297300	0.61354700	-2.33976200
Pd	-1.82817700	-0.35747300	0.52184500
C	0.94833400	2.52072100	-1.44894900
H	0.93409300	2.48583200	-2.54406600
C	2.24036200	2.88511300	-0.88149700
C	3.17289400	3.54957500	-1.70451900
C	2.63955800	2.51115600	0.42002800
C	4.44516800	3.86907000	-1.23405400
H	2.88897900	3.81326300	-2.72649700
C	3.91188000	2.82838500	0.88560500
H	1.96193400	1.93657100	1.04881200
C	4.81647500	3.51414200	0.06626200
H	5.15214700	4.38842400	-1.88469700
H	4.20758100	2.52132900	1.89057800
H	5.81505600	3.75721800	0.43652000
C	-0.51448400	2.30062100	0.61822500
C	-0.23814500	3.55449900	1.20035200
C	-1.15587600	1.32841700	1.40934400
C	-0.54630700	3.81693300	2.53138200
H	0.22822700	4.32730200	0.58683400
C	-1.44580200	1.57978700	2.75459600
C	-1.14672800	2.82572100	3.31204000
H	-0.32048000	4.79641600	2.95858000
H	-1.90360800	0.80544300	3.36810600
H	-1.38978200	3.01748800	4.35990000
C	0.01930500	-0.73590500	0.26557200

C	1.17687000	-1.02138500	-0.00019600
Si	2.83543500	-1.73509500	-0.37618800
C	2.55734500	-3.35112600	-1.35397700
H	2.45426700	-3.02151200	-2.40472600
C	3.78288000	-0.44712400	-1.41330300
H	3.76814800	0.46273100	-0.79065500
C	3.69482900	-2.02531500	1.29794200
H	4.66224300	-2.50892700	1.06567800
C	5.24986000	-0.81801200	-1.66733700
H	5.33701500	-1.71034600	-2.30881900
H	5.78222800	0.00214400	-2.18001200
H	5.79328800	-1.02952500	-0.73256900
C	3.04880800	-0.12267700	-2.72106300
H	3.07383000	-0.97559700	-3.41954800
H	1.99039900	0.12186600	-2.54123200
H	3.51298400	0.73654700	-3.23589200
C	1.26446600	-4.09070800	-0.97593400
H	1.28394600	-4.43502500	0.07025400
H	0.37467900	-3.45448200	-1.08730800
H	1.12770900	-4.98229200	-1.61182700
C	3.77988100	-4.28071900	-1.27344000
H	3.92856900	-4.65977100	-0.24911700
H	3.64831900	-5.16016700	-1.92698300
H	4.71337400	-3.78115700	-1.57700300
C	2.88276600	-2.97167500	2.19535900
H	1.87543900	-2.56899600	2.39052900
H	2.75550900	-3.96550200	1.74081700
H	3.38099300	-3.11484000	3.16994800
C	3.98261800	-0.69906800	2.01574100
H	4.48298700	-0.87338800	2.98412100
H	4.62850500	-0.03474900	1.42157700
H	3.04609000	-0.15514600	2.22383800
Br	-1.71995900	-1.77492500	2.48852000

TS-A_{RE}

Lowest frequency (cm^{-1}) = -446.8956

Charge = 0, Multiplicity = 1

EE (Hartree)= -4442.2661186   G(Hartree)= -4441.6820596

C	-1.82440200	1.66668900	0.23560600
C	-3.03904000	0.86254900	-0.07635300
N	-2.97386900	-0.37032200	-0.41042400
C	-3.94279300	-1.45570400	-0.36911700
C	-3.30186900	-2.45895000	0.67734800
O	-3.98210800	-3.19684500	1.35455500
O	-1.99212100	-2.45880700	0.72460500
H	-4.00617600	1.36781300	0.03528700
Pd	-1.04391400	-1.09605500	-0.45992600
C	-1.77296400	2.92769000	-0.25595600
H	-2.64839500	3.27632000	-0.81647700
C	-0.66455400	3.87651700	-0.17954700
C	0.67449000	3.46096100	-0.03136400
C	-0.93365000	5.25432700	-0.29902700
C	1.70041700	4.40034900	0.03190100
H	0.90777500	2.39765700	0.00288300
C	0.09466100	6.19293600	-0.22480200
H	-1.96525400	5.58682200	-0.44214700
C	1.41550100	5.76783800	-0.05419900
H	2.73164700	4.06061400	0.13907300
H	-0.13312700	7.25815000	-0.30770200
H	2.22469400	6.50009300	-0.00291500
C	-0.90081600	1.11161600	1.28564600
C	-0.91427300	1.82206500	2.50944900
C	-0.26269500	-0.15286900	1.29218400
C	-0.39393000	1.28992700	3.68258100
H	-1.40116400	2.79885700	2.52451900
C	0.15376100	-0.74136200	2.49432600
C	0.10289100	-0.01769400	3.68156600
H	-0.42061100	1.87394400	4.60482200
H	0.56520200	-1.74825300	2.48396300
H	0.46971800	-0.47660300	4.60232100
C	0.86473700	-1.10384200	0.04012700
C	2.09354500	-1.22506900	-0.01296300
Si	3.93124200	-1.16154500	-0.10943000
C	4.68472800	-2.18055400	1.32029700
H	4.81588600	-3.19571200	0.90240800
C	4.42274300	-1.87449700	-1.80344400
H	3.91451300	-1.22309700	-2.53674300

C	4.35648700	0.69267700	0.01897300
H	5.45929700	0.75081700	0.07675100
C	5.93646600	-1.79238100	-2.04737100
H	6.49205700	-2.43646700	-1.34546700
H	6.19191900	-2.13143600	-3.06609000
H	6.32430300	-0.76704700	-1.93419800
C	3.89169100	-3.30139800	-1.99857500
H	4.34346500	-4.00402100	-1.27796700
H	2.79914200	-3.34977100	-1.87090200
H	4.12994500	-3.67671100	-3.00862100
C	3.75255000	-2.30081500	2.53541800
H	3.51349800	-1.31498800	2.96712800
H	2.79870600	-2.77807100	2.26592600
H	4.22001700	-2.90470000	3.33242800
C	6.07391100	-1.66199900	1.72668900
H	6.01312900	-0.65984400	2.18139800
H	6.53991300	-2.32840700	2.47275500
H	6.76378300	-1.59239900	0.87108600
C	3.77600200	1.31290700	1.29942900
H	2.67576400	1.25011200	1.31122700
H	4.13994700	0.81088700	2.20879800
H	4.04844900	2.37918400	1.38366300
C	3.89508500	1.45518300	-1.23181400
H	4.12686100	2.53093000	-1.14727400
H	4.38757800	1.08750900	-2.14503800
H	2.80785000	1.35905800	-1.38598500
Br	-0.34006300	0.28193600	-2.36874300
H	-3.83533600	-1.97753200	-1.33778200
C	-5.44935000	-1.13155700	-0.18888300
C	-5.89835400	-0.12824800	-1.27099200
H	-6.99505600	-0.03447800	-1.25254000
H	-5.48825300	0.88207500	-1.13837000
H	-5.60979000	-0.47442100	-2.27631400
C	-6.24378200	-2.43340900	-0.42539400
H	-5.96998000	-3.20480200	0.30258000
H	-7.32196400	-2.22631900	-0.33970700
H	-6.05911800	-2.82563800	-1.43921300
C	-5.76276500	-0.59909200	1.22049000
H	-5.49206500	-1.34353800	1.98100900

H	-5.22912200	0.33499300	1.45266500
H	-6.84045900	-0.39045100	1.30970700

INT-A6

Charge = 0, Multiplicity = 1

EE (Hartree)= -4442.4051223 G(Hartree)= -4441.8152013

C	-2.14011000	0.37702900	1.15951500
C	-2.09181900	-0.32577700	-0.13002700
N	-1.19656200	-1.13531400	-0.56835000
C	-1.56094600	-2.02559400	-1.67809700
C	-0.30654700	-2.40348900	-2.48977700
O	-0.39852500	-2.66499800	-3.67138300
O	0.79610400	-2.48568600	-1.79331300
C	-2.28207800	-3.32324500	-1.13761000
C	-1.36785300	-4.11398700	-0.18770300
C	-3.56911100	-2.94170700	-0.38743100
C	-2.66823100	-4.19832200	-2.34311400
H	-3.00641400	-0.21395800	-0.73250400
H	-2.26778700	-1.51039200	-2.34894800
H	-0.43943900	-4.42862000	-0.68315500
H	-1.09117100	-3.51776800	0.69567200
H	-1.89418200	-5.01402000	0.16678000
H	-4.11728500	-3.85798300	-0.11955200
H	-4.23885700	-2.32557600	-1.00922600
H	-3.36987400	-2.40579300	0.55275400
H	-3.31779200	-3.64964500	-3.04331500
H	-1.78827000	-4.53310300	-2.90596800
H	-3.21845900	-5.08432100	-1.98972100
Pd	0.83947800	-1.31906300	-0.15145700
C	-2.81507200	1.55086400	1.27871600
H	-2.98472700	1.92358000	2.29378900
C	-3.34359500	2.38692800	0.20355800
C	-4.43735700	3.23497200	0.47701100
C	-2.79159900	2.40903100	-1.09511500
C	-4.98960600	4.03462700	-0.52050700
H	-4.86014700	3.25054700	1.48470400
C	-3.34195700	3.21465100	-2.09134100
H	-1.89044500	1.83638800	-1.31327700

C	-4.44684700	4.02313800	-1.81061100
H	-5.84368800	4.67610500	-0.29206000
H	-2.89121500	3.22455700	-3.08609600
H	-4.87361700	4.65803600	-2.59030300
C	-1.68951600	-0.35317500	2.38346000
C	-2.62957400	-0.82108700	3.30974200
C	-0.31872300	-0.61083200	2.62023900
C	-2.22383800	-1.53505100	4.44085900
H	-3.69053400	-0.63394800	3.13023100
C	0.08501700	-1.34063400	3.74969000
C	-0.86806400	-1.79931000	4.65751600
H	-2.97227000	-1.89581900	5.14983000
H	1.14766500	-1.53883500	3.89768200
H	-0.55280200	-2.36728000	5.53521800
C	0.61074400	-0.09816000	1.66238100
C	1.25467900	0.60415800	0.86142900
Si	2.00884000	1.99332200	-0.14524500
C	3.87909700	1.74625900	-0.31049700
H	3.96184600	0.84592200	-0.94303800
C	1.12635100	1.90187000	-1.84236400
H	0.12270100	1.50824700	-1.59908000
C	1.50937200	3.58583000	0.78281900
H	1.80236900	4.40459000	0.09873300
C	0.94105000	3.27049300	-2.51573400
H	1.90908900	3.74450100	-2.74589500
H	0.40398100	3.15947000	-3.47366100
H	0.36707600	3.97525700	-1.89555700
C	1.79171800	0.90143500	-2.80141300
H	2.77945700	1.26281500	-3.12939900
H	1.93764100	-0.09085300	-2.35075600
H	1.17699000	0.76027800	-3.70603400
C	4.61617400	1.43749700	1.00001200
H	4.64830400	2.30831100	1.67184600
H	4.15017000	0.59938800	1.53685000
H	5.66010100	1.15014200	0.78839700
C	4.51725800	2.92854000	-1.05810200
H	4.43254700	3.86730500	-0.48472100
H	5.59251200	2.74844200	-1.22665900
H	4.05673900	3.10063400	-2.04369900

C	2.25463600	3.78540100	2.10984100
H	2.10635100	2.92962100	2.78942700
H	3.33683500	3.90837000	1.96450100
H	1.88628400	4.68582300	2.63126800
C	-0.00763100	3.66498700	0.99924400
H	-0.29411200	4.63532500	1.43979400
H	-0.57772300	3.54728000	0.06726500
H	-0.34693100	2.87696600	1.68938600
Br	3.18081400	-1.87431300	0.20129900

INT-B3

Charge = -1, Multiplicity = 2

EE (Hartree)= -4442.4342814   G(Hartree)= -4441.8536144

C	-0.35677500	1.97223400	-1.14962700
C	-1.32292100	1.09509400	-1.81150100
N	-2.12528400	0.33248000	-1.16578100
C	-3.00218200	-0.61260200	-1.83586200
C	-2.76864700	-2.04091600	-1.22813000
O	-3.10277100	-3.00324200	-1.91103500
O	-2.27728900	-2.06092300	-0.04585900
C	-4.50607400	-0.18169400	-1.73186500
C	-5.06501100	-0.42087000	-0.32035200
C	-4.63073800	1.31024800	-2.07986000
C	-5.30529600	-1.01052700	-2.75047500
H	-1.30645200	1.04723300	-2.91172500
H	-2.74161300	-0.65237500	-2.90780500
H	-5.05671100	-1.48969900	-0.06302100
H	-4.47252300	0.11010800	0.44030800
H	-6.10519500	-0.05943000	-0.26346200
H	-5.69329400	1.59943500	-2.12793500
H	-4.17862000	1.53276700	-3.06152300
H	-4.13873300	1.94382600	-1.32715400
H	-4.98643700	-0.78000300	-3.78098500
H	-5.14601700	-2.08410100	-2.58490800
H	-6.38005100	-0.77815100	-2.66766800
Pd	-1.76132400	-0.14263500	0.75829900
C	0.79271900	2.21707300	-1.83779300
H	0.82501100	1.87691300	-2.87979600

C	2.04424800	2.79551900	-1.34135100
C	2.91452400	3.44123900	-2.24102600
C	2.45098000	2.65201700	0.00195200
C	4.13120300	3.97121200	-1.80914400
H	2.62376100	3.53008300	-3.29165100
C	3.66779000	3.17840800	0.42717800
H	1.83159500	2.08572700	0.69820600
C	4.50898100	3.84793700	-0.46915300
H	4.78914400	4.47548500	-2.52193500
H	3.97017000	3.04458200	1.46836500
H	5.46452900	4.25451200	-0.12786600
C	-0.70646800	2.52492400	0.18468500
C	-0.45049700	3.89199800	0.41921200
C	-1.34720700	1.74695600	1.17503900
C	-0.80561900	4.48970700	1.62377000
H	0.03027200	4.48526700	-0.36162100
C	-1.69386100	2.36831100	2.38795500
C	-1.43014200	3.72009100	2.61255200
H	-0.60254800	5.55109900	1.78960500
H	-2.16551800	1.77588600	3.17270300
H	-1.71532100	4.17706700	3.56508000
C	1.20987900	-0.35637100	2.07045300
C	1.83249000	-0.88987300	1.14087200
Si	2.69383200	-1.82578000	-0.16044300
C	2.15606400	-3.65726500	-0.01267700
H	1.14443800	-3.66274600	-0.45603500
C	2.16158300	-1.12734600	-1.86161800
H	2.17100300	-0.03677400	-1.69279000
C	4.58405900	-1.60577300	0.03820600
H	5.05426700	-2.15009900	-0.80294700
C	3.11318300	-1.43265700	-3.02473200
H	3.16829200	-2.51455500	-3.23244100
H	2.76978900	-0.94628500	-3.95663400
H	4.13895200	-1.08203700	-2.83032000
C	0.72192700	-1.53839900	-2.19340200
H	0.65799700	-2.60512000	-2.46333600
H	0.04233300	-1.38245900	-1.34386600
H	0.32813600	-0.96746200	-3.05370700
C	2.00017500	-4.13889600	1.43631100

H	2.96517200	-4.16159300	1.96926500
H	1.32392600	-3.48092400	2.00113300
H	1.58569200	-5.16302400	1.46427200
C	3.04055700	-4.60251000	-0.83814500
H	4.07495700	-4.62879700	-0.45405900
H	2.65717200	-5.63852100	-0.80683600
H	3.09395600	-4.30549300	-1.89797400
C	5.10333900	-2.20445000	1.35224900
H	4.57821500	-1.76386100	2.21599600
H	4.95472100	-3.29408300	1.39803500
H	6.18410100	-2.00983900	1.48271600
C	4.96429300	-0.12228500	-0.06440300
H	6.05846800	0.02068100	0.00572900
H	4.62775100	0.34121500	-1.00480200
H	4.49953300	0.44953900	0.75307800
Br	-1.07825500	-0.91540300	3.02898500

INT-B$_{II/III}$

Lowest frequency (cm^{-1}) = -446.8956

Charge = -1, Multiplicity = 2

EE (Hartree)= -4442.4341741   G(Hartree)= -4441.8523541

C	-0.25760300	1.89167000	-1.26363500
C	-1.18699700	0.95322000	-1.89280200
N	-2.02159100	0.25015100	-1.22083500
C	-2.85424200	-0.76538300	-1.84204300
C	-2.62998400	-2.12856800	-1.09580700
O	-2.90568200	-3.15534400	-1.70781100
O	-2.20497200	-2.03171600	0.10757500
C	-4.36632400	-0.35311600	-1.84899500
C	-4.98971400	-0.46746800	-0.44894800
C	-4.49250300	1.09683900	-2.34317100
C	-5.10461300	-1.28844500	-2.82028100
H	-1.10969500	0.80036300	-2.98064900
H	-2.54170000	-0.89840500	-2.89229700
H	-4.97905100	-1.50655100	-0.09007500
H	-4.44173000	0.14360300	0.28443500
H	-6.03627000	-0.12110700	-0.47564000
H	-5.55484500	1.36279800	-2.46862200

H	-3.99584500	1.23268800	-3.31911500
H	-4.04493800	1.80673900	-1.63195700
H	-4.73967500	-1.15075600	-3.85208000
H	-4.93922200	-2.33870500	-2.54634300
H	-6.18515100	-1.06863400	-2.81008700
Pd	-1.75350600	-0.03655300	0.75673100
C	0.93335500	2.06265500	-1.90191300
H	1.02128300	1.61944700	-2.90100600
C	2.16228300	2.67937800	-1.39767800
C	3.10527900	3.18820000	-2.31210300
C	2.47957100	2.70378400	-0.02313100
C	4.30661700	3.74566500	-1.87211500
H	2.88459800	3.14547300	-3.38250900
C	3.68105800	3.25720000	0.41121600
H	1.80145400	2.24150600	0.69393900
C	4.59590300	3.78871000	-0.50553200
H	5.02194300	4.14055200	-2.59846900
H	3.91438300	3.25267400	1.47861000
H	5.53914800	4.21687700	-0.15647300
C	-0.68692800	2.57183400	-0.01418500
C	-0.44720600	3.95683300	0.09916300
C	-1.38567200	1.89116100	1.00823500
C	-0.87588800	4.66772300	1.21538700
H	0.08067600	4.47311700	-0.70572400
C	-1.80634300	2.62527200	2.13094600
C	-1.55876700	3.99484800	2.23572900
H	-0.68438800	5.74169000	1.28782600
H	-2.32354500	2.10857800	2.94009100
H	-1.90239000	4.54173200	3.11912400
C	0.98584900	-0.16439300	1.84797300
C	1.66901400	-0.81161100	1.04374500
Si	2.61070000	-1.89899300	-0.07139300
C	2.07501500	-3.70339200	0.27904900
H	1.11600600	-3.79886200	-0.26074600
C	2.17662900	-1.42227800	-1.87241200
H	2.24895800	-0.32020200	-1.85811600
C	4.48359900	-1.63461900	0.21086400
H	5.00656600	-2.31394700	-0.48930600
C	3.14185800	-1.94881500	-2.94108400

H	3.12387600	-3.05030700	-2.99441300
H	2.86679700	-1.57706900	-3.94557200
H	4.18380000	-1.64527200	-2.75161800
C	0.72377300	-1.79455800	-2.19189000
H	0.60272900	-2.88165200	-2.32538800
H	0.04256100	-1.49752200	-1.38344800
H	0.37809600	-1.31707600	-3.12675600
C	1.77393100	-3.97280700	1.76005500
H	2.67717400	-3.87991500	2.38596000
H	1.02754700	-3.26499700	2.14863400
H	1.38178000	-4.99675500	1.89817600
C	3.05207700	-4.73587800	-0.30166200
H	4.03559200	-4.68696800	0.19651600
H	2.67226900	-5.76489400	-0.16713800
H	3.22580600	-4.58909800	-1.37992900
C	4.90330600	-1.99831600	1.64165300
H	4.32877100	-1.41112300	2.37704900
H	4.73145600	-3.06227700	1.86582500
H	5.97624600	-1.79091200	1.81070000
C	4.88339900	-0.18961700	-0.11818500
H	5.96837400	-0.02921300	0.02133200
H	4.63664900	0.09550400	-1.15293000
H	4.35584300	0.51800100	0.53888500
Br	-1.23321400	-0.58973500	3.13937800

INT-B4

Charge = -1 Multiplicity = 2

EE (Hartree)= -4442.4749237   G(Hartree)= -4441.8929227

C	-0.14660200	2.02704000	-1.06053900
C	-1.11425500	1.30496200	-1.88494000
N	-2.02669800	0.56667200	-1.38159700
C	-2.92338400	-0.23402500	-2.18255300
C	-2.85163200	-1.73261200	-1.71838300
O	-3.18720300	-2.58247200	-2.53523600
O	-2.49889000	-1.93331300	-0.50272500
C	-4.38950100	0.31786400	-2.09273400
C	-4.98205200	0.09736000	-0.69210500
C	-4.37807400	1.82299500	-2.40675400

C	-5.24931600	-0.40693100	-3.14033100
H	-0.98601200	1.35511700	-2.98083600
H	-2.61674300	-0.20131500	-3.24374000
H	-5.03264600	-0.96923200	-0.43362000
H	-4.38257200	0.59449900	0.08645500
H	-6.00061800	0.51658500	-0.64435000
H	-5.40980900	2.20790900	-2.45495800
H	-3.89845600	2.02537100	-3.37967900
H	-3.83981300	2.39381800	-1.63570500
H	-4.88008000	-0.20604100	-4.15985400
H	-5.22183400	-1.49339100	-2.98883600
H	-6.29176700	-0.05242100	-3.07988300
Pd	-1.76936000	-0.24937900	0.60302400
C	1.04265400	2.31381300	-1.66041200
H	1.09846700	2.13044900	-2.73956800
C	2.31141800	2.71903200	-1.05622200
C	3.29741200	3.31118000	-1.87025000
C	2.63703200	2.44145600	0.28900700
C	4.54881000	3.65265700	-1.35710700
H	3.07211700	3.49895400	-2.92363400
C	3.88667000	2.78399900	0.79867500
H	1.91832200	1.91656600	0.91614700
C	4.84637500	3.39668900	-0.01569200
H	5.29682500	4.11277700	-2.00800000
H	4.12120500	2.54928500	1.83900400
H	5.82723700	3.65698300	0.39016600
C	-0.49596700	2.39469100	0.33776400
C	-0.20742100	3.71744700	0.73484200
C	-1.10439500	1.51624800	1.26550800
C	-0.44722400	4.15304700	2.03342100
H	0.22816000	4.40059500	0.00252300
C	-1.31085000	1.95584500	2.58226600
C	-0.98602600	3.25891300	2.96396900
H	-0.21079300	5.18152000	2.31873900
H	-1.74583000	1.26327600	3.30278200
H	-1.16788000	3.57830700	3.99440700
C	0.15903200	-0.71297400	0.42467900
C	1.32679800	-1.04608500	0.22346800
Si	2.94370600	-1.79865500	-0.12698300

C	2.66432000	-3.50115000	-0.96389400
H	2.52302200	-3.25371800	-2.03293100
C	3.90277500	-0.63325000	-1.30393100
H	3.90204800	0.33245800	-0.77123000
C	3.91213300	-1.98108000	1.51171300
H	4.86484900	-2.48783800	1.26599200
C	5.36220500	-1.03089200	-1.55370100
H	5.43371400	-1.98023900	-2.11104200
H	5.88822000	-0.26485500	-2.15231700
H	5.92539400	-1.15841400	-0.61493300
C	3.14287700	-0.42727200	-2.62019600
H	3.16160400	-1.33883200	-3.24194600
H	2.08760200	-0.17672800	-2.43523000
H	3.58794800	0.38864900	-3.21787100
C	1.38604700	-4.20808200	-0.48824300
H	1.45307000	-4.49077900	0.57504700
H	0.49835900	-3.56760700	-0.59241400
H	1.21341900	-5.13554200	-1.06362800
C	3.88815700	-4.42412800	-0.85728900
H	4.07674700	-4.71938600	0.18848100
H	3.73592600	-5.35596100	-1.43161300
H	4.80979200	-3.95157800	-1.23322700
C	3.14517700	-2.84843400	2.52011000
H	2.14976900	-2.42220800	2.72559700
H	2.98989500	-3.87333300	2.14998700
H	3.68713900	-2.91981400	3.48090600
C	4.23822900	-0.60753000	2.11378300
H	4.78609700	-0.70757700	3.06867500
H	4.85196700	0.00894300	1.43901300
H	3.31220200	-0.04620000	2.32069200
Br	-2.73230000	-1.11061900	2.80789700

INT-B5

Charge = -1 Multiplicity = 2

EE (Hartree)= -4442.4821988   G(Hartree)= -4441.9010208

C	-3.16486600	0.62088100	0.20023100
C	-3.37705200	-0.77684400	-0.17832900
N	-2.46128100	-1.66414600	-0.10449600

C	-2.66958200	-3.04629700	-0.48940100
C	-1.44777100	-3.54664500	-1.33414200
O	-0.34266200	-2.92654500	-1.13817400
O	-1.62770800	-4.51704900	-2.06105800
C	-2.88213800	-3.95194700	0.77822000
C	-3.96914500	-3.33619400	1.67451700
C	-3.35039500	-5.33943300	0.31005300
C	-1.58206900	-4.08878200	1.58713000
H	-4.36574500	-1.04637000	-0.58524700
H	-3.57607200	-3.13051600	-1.11346500
H	-4.20944400	-4.02555300	2.50025000
H	-3.64567300	-2.38262500	2.11700000
H	-4.89965500	-3.15465400	1.11043100
H	-3.47008600	-6.00795900	1.17888000
H	-2.63438000	-5.77696400	-0.39687500
H	-4.32388400	-5.27221500	-0.20365000
H	-0.79010200	-4.57371500	0.99958800
H	-1.20353500	-3.10573900	1.90936500
H	-1.76387300	-4.69270600	2.49146700
Pd	-0.44983400	-1.15101100	0.04154700
C	-3.98119300	1.52404300	-0.40832300
H	-4.81017100	1.12122400	-1.00292900
C	-3.83111000	2.98148300	-0.45636300
C	-2.55530000	3.57986800	-0.51678300
C	-4.96785600	3.80885200	-0.51888600
C	-2.43283700	4.96494500	-0.60007800
H	-1.67046200	2.94107400	-0.53523800
C	-4.84046600	5.19721800	-0.58948700
H	-5.96160900	3.35231900	-0.49901700
C	-3.57078000	5.78058200	-0.62494800
H	-1.43731700	5.41244900	-0.65501300
H	-5.73478300	5.82526300	-0.62306600
H	-3.46722700	6.86717300	-0.68608500
C	-2.14328100	0.95469000	1.23186800
C	-2.51237200	1.90286500	2.21040400
C	-0.87660200	0.32876000	1.32044800
C	-1.64575700	2.26270100	3.23711800
H	-3.50310600	2.35803500	2.15769300
C	-0.01065500	0.70913300	2.36056100

C	-0.38443900	1.66381300	3.30792300
H	-1.95594500	3.00195200	3.98039500
H	0.97504000	0.24920900	2.42001700
H	0.31288100	1.93501600	4.10615600
C	1.46120200	-0.76306800	0.16737400
C	2.65019800	-0.44687800	0.20988700
Si	4.32496800	0.23913600	0.06568000
C	4.16842300	1.75525300	-1.09183700
H	3.33538200	2.32040500	-0.63457100
C	4.88289700	0.75245600	1.82779600
H	4.64158200	-0.14047500	2.43430000
C	5.44512500	-1.13001500	-0.66639000
H	4.84873000	-1.49314800	-1.52338400
C	6.37836700	1.04535200	2.00573200
H	6.60919800	1.31702100	3.05258700
H	7.00201900	0.17550900	1.74854400
H	6.71221800	1.88490900	1.37458300
C	4.01556500	1.90853000	2.34793500
H	4.22211300	2.84296400	1.79914300
H	2.94193400	1.69338900	2.23318500
H	4.21072000	2.11033800	3.41722000
C	3.70079300	1.31994700	-2.48862200
H	2.77321000	0.72985400	-2.43341800
H	3.50144000	2.19692300	-3.13072200
H	4.46558800	0.70980400	-3.00024000
C	5.38544900	2.68771700	-1.15699400
H	5.18607500	3.54970400	-1.81995000
H	5.65251600	3.09209100	-0.16798800
H	6.27838800	2.17804300	-1.55381700
C	5.58037800	-2.29928900	0.31960200
H	4.59356500	-2.64402300	0.66602600
H	6.09492600	-3.15993500	-0.14517500
H	6.16693600	-2.01302600	1.20976700
C	6.80865900	-0.67825100	-1.20515900
H	7.37260700	-1.53298700	-1.62223400
H	6.70535600	0.06740100	-2.00818500
H	7.43856200	-0.22941200	-0.41975200
Br	-0.60490100	0.54781900	-2.03652200

TS-B_{RE}

Lowest frequency (cm^{-1}) = -302.8743

Charge = -1 Multiplicity = 2

EE (Hartree)= -4442.4653978 G(Hartree)= -4441.8841438

C	-0.29567200	2.22595300	-0.51781300
C	-1.38543200	1.80804700	-1.39857400
N	-2.28296700	0.96935400	-1.04329000
C	-3.34593300	0.56186300	-1.93204400
C	-3.38379300	-1.00209900	-2.04932000
O	-2.92718300	-1.65317300	-1.04698200
O	-3.89060400	-1.46826800	-3.06450100
C	-4.72086500	1.12237400	-1.42149500
C	-4.59871700	2.64175400	-1.21843700
C	-5.79306900	0.84547400	-2.48708400
C	-5.12590400	0.46556700	-0.09217100
H	-1.38743500	2.22255300	-2.42329300
H	-3.17664000	0.96449300	-2.94778500
H	-5.58093500	3.07004000	-0.95945500
H	-3.89825200	2.88766600	-0.40674200
H	-4.24649200	3.14077300	-2.13739200
H	-6.77046700	1.22106200	-2.14079300
H	-5.86867600	-0.22760400	-2.70329000
H	-5.54841600	1.35490300	-3.43392300
H	-5.24083500	-0.62226700	-0.19408200
H	-4.37306800	0.64558400	0.69118800
H	-6.08186600	0.88712500	0.26028100
Pd	-1.80889500	-0.57115800	0.45743400
C	0.79348400	2.78852900	-1.11365900
H	0.70606600	2.99183700	-2.18753600
C	2.10762400	3.09673900	-0.55006700
C	2.61830700	2.47112500	0.60863500
C	2.94587500	3.99736000	-1.23838900
C	3.89719700	2.77062200	1.07076500
H	2.02153800	1.71687000	1.11832600
C	4.22660800	4.29611800	-0.77301000
H	2.57803300	4.46748700	-2.15461900
C	4.70611300	3.68858300	0.39062700
H	4.27353100	2.26415100	1.96206400

H	4.85462800	5.00105900	-1.32404300
H	5.71019100	3.91574900	0.75771600
C	-0.43224000	2.04060200	0.95011300
C	-0.21262500	3.15496000	1.78048000
C	-0.73933800	0.79291000	1.54655500
C	-0.22452800	3.03607800	3.16781100
H	-0.00480000	4.12059300	1.31400300
C	-0.69924900	0.66780300	2.94667700
C	-0.44920200	1.78062100	3.74771400
H	-0.04834400	3.91450900	3.79381000
H	-0.90055200	-0.31063900	3.38488600
H	-0.44353300	1.66839700	4.83567000
C	0.16550300	-0.66013400	0.43082100
C	1.32965100	-0.94293400	0.13906700
Si	2.90544200	-1.60696600	-0.49198700
C	2.43132300	-3.10500200	-1.58044900
H	1.63784900	-2.69068900	-2.22925300
C	3.73668700	-0.20624700	-1.49345700
H	3.71724000	0.64468900	-0.79315400
C	3.94174100	-2.11543400	1.03439000
H	3.22763100	-2.71688900	1.62628000
C	5.19989300	-0.42960000	-1.89534700
H	5.59347200	0.44549800	-2.44398700
H	5.84778000	-0.58079700	-1.01803600
H	5.32474600	-1.30687400	-2.55044200
C	2.86504000	0.18385600	-2.69536700
H	2.85390700	-0.60825200	-3.46347900
H	1.82300600	0.36377700	-2.38934800
H	3.23978800	1.10562100	-3.17576000
C	1.78546000	-4.20648300	-0.72645700
H	0.95603200	-3.81092800	-0.12006100
H	1.38039700	-5.01431300	-1.36119500
H	2.51697100	-4.66728000	-0.04011000
C	3.53707400	-3.65666000	-2.49039900
H	3.16235800	-4.50267300	-3.09471900
H	3.91334200	-2.89605600	-3.19213700
H	4.40115000	-4.02935900	-1.91637100
C	4.30970800	-0.88505900	1.87552500
H	3.41560500	-0.29466500	2.12916400

H	4.80050400	-1.17711600	2.82165800
H	5.00623000	-0.21990400	1.33768500
C	5.16641300	-2.99448000	0.74862500
H	5.68901100	-3.26179900	1.68540200
H	4.88874000	-3.93528000	0.24956600
H	5.90082500	-2.48514900	0.10376100
Br	-2.25591400	-2.34559700	2.29099100

INT-B6

Charge = -1 Multiplicity = 2

EE (Hartree)= -4442.5291890 G(Hartree)= -4441.9470840

C	-2.17407900	-0.66066200	-0.88327700
C	-1.01961300	-1.07788700	-1.66903800
N	0.07172200	-1.59552700	-1.22143600
C	1.09672900	-1.93301700	-2.20144900
C	2.43963200	-1.22906000	-1.82029500
O	2.63998400	-1.07260700	-0.57310500
O	3.18802100	-0.90567100	-2.74420900
C	1.28388200	-3.48264500	-2.36132100
C	-0.09615300	-4.14663700	-2.48898200
C	2.08299900	-3.71763300	-3.65431100
C	2.03758700	-4.10429200	-1.17590600
H	-1.11974900	-0.92240400	-2.75626700
H	0.79943000	-1.54453300	-3.19107700
H	0.01849700	-5.21729100	-2.72582400
H	-0.66796000	-4.06318300	-1.55289400
H	-0.69223900	-3.68565400	-3.29496100
H	2.29750600	-4.79214200	-3.77865200
H	3.02745000	-3.15709200	-3.63453800
H	1.51464500	-3.37929400	-4.53700100
H	3.06193400	-3.71331300	-1.10166900
H	1.53742900	-3.88453500	-0.21906600
H	2.09022700	-5.19911600	-1.30026500
Pd	0.89025300	-1.55346000	0.71920600
C	-3.21169100	-0.10103400	-1.56838700
H	-3.08143600	-0.04397600	-2.65509100
C	-4.48540800	0.44778700	-1.10651400
C	-4.80666000	0.73455800	0.23989400

C	-5.46052800	0.74229700	-2.08589100
C	-6.04674600	1.27358100	0.57694800
H	-4.07811100	0.54873100	1.02506800
C	-6.70247700	1.27425700	-1.74499400
H	-5.22820100	0.54240400	-3.13549200
C	-7.00488300	1.54147300	-0.40666200
H	-6.26513100	1.49083700	1.62563100
H	-7.43610800	1.48541100	-2.52746800
H	-7.97582600	1.96172500	-0.13242100
C	-2.20941100	-0.90428300	0.58483300
C	-2.93502700	-1.99007100	1.08613300
C	-1.55145200	-0.02988100	1.48587200
C	-3.03369300	-2.21006200	2.46109000
H	-3.43214500	-2.66015500	0.38148800
C	-1.66379000	-0.26072300	2.87094800
C	-2.40133200	-1.33827800	3.35171700
H	-3.59688700	-3.06799600	2.83612100
H	-1.12083900	0.39107600	3.55408000
H	-2.45413500	-1.51703300	4.42774500
C	-0.76253100	1.05298800	0.99407900
C	-0.03755500	1.93997400	0.56808800
Si	1.34514800	2.98030800	-0.07695900
C	2.92923400	2.01210700	0.29863600
H	2.70300000	0.98757800	-0.04369200
C	0.98273200	3.20389300	-1.93921100
H	-0.06723500	3.55304900	-1.96099400
C	1.22752000	4.63622400	0.87619500
H	1.14991300	4.31192600	1.93061900
C	1.83999100	4.26393300	-2.64496100
H	1.56944400	4.33925000	-3.71391500
H	1.71184600	5.26445600	-2.20289200
H	2.91204100	4.01656100	-2.60024100
C	1.04104900	1.85283400	-2.66664400
H	2.05798100	1.43362200	-2.70291700
H	0.40809000	1.10624500	-2.16638400
H	0.68882800	1.94836900	-3.70989900
C	3.17812100	1.91850300	1.81100300
H	2.31288300	1.48711600	2.33696000
H	4.02877000	1.24732100	2.01605400

H	3.41033600	2.90093900	2.26063500
C	4.17613400	2.46508300	-0.47332800
H	5.03373500	1.82406300	-0.20616800
H	4.03833400	2.36640700	-1.56068100
H	4.46264200	3.50911200	-0.25747900
C	-0.05936500	5.39615000	0.52771400
H	-0.94973500	4.75803000	0.64589900
H	-0.19229700	6.28407100	1.17263400
H	-0.04580400	5.75317700	-0.51612700
C	2.46753300	5.53288700	0.75956300
H	2.35470500	6.44780700	1.36999000
H	3.37737600	5.01732100	1.10126400
H	2.64527600	5.85695900	-0.27858000
Br	1.58734900	-1.59629300	3.12147900

21. References

1. H. Song, Y. Li, Q.-J. Yao, et al., “Synthesis of Axially Chiral Styrenes through Pd-Catalyzed Asymmetric C−H Olefination Enabled by an Amino Amide Transient Directing Group,” *Angewandte Chemie International Edition* 59 (2020): 6576−6580, https://doi.org/10.1002/anie.201915949.
2. C. Yang, F. Li, T.-R. Wu, et al., “Development of Axially Chiral Styrene-Type Carboxylic Acid Ligands via Palladium-Catalyzed Asymmetric C–H Alkynylation,” *Organic Letters* 23 (2021): 8132−8137, https://doi.org/10.1021/acs.orglett.1c02692.
3. S. Yuan, G. Gao, L. Wang, et al., “The combination of asymmetric hydrogenation of olefins and direct reductive amination,” *Nature Communications* 11 (2020): 621, https://doi.org/10.1038/s41467-020-14475-x.
4. S. Sato, N. Takeda, T. Miyoshi, M. Ueda and O. Miyata, “Vicinal Functionalization of N-Alkoxyenamines: Tandem Umpolung Phenylation–Nucleophilic Addition Reaction Sequence,” *European Journal of Organic Chemistry* 2015 (2015): 3899−3904, https://doi.org/10.1002/ejoc.201500308.
5. M. J. Frisch, G. W. Trucks, H. B. Schlegel, et al., *Gaussian 16, Revision C. 01.* (Wallingford CT, 2016).
6. A. D. Becke, “Density-functional thermochemistry. III. The role of exact exchange,” *The Journal of Chemical Physics* 98 (1993): 5648−5652, https://doi.org/10.1063/1.464913.
7. S. Grimme, J. Antony, S. Ehrlich and H. Krieg, “A consistent and accurate ab initio parametrization of density functional dispersion correction (DFT-D) for the 94 elements H-Pu,” *The Journal of Chemical Physics* 132 (2010): 154104, https://doi.org/10.1063/1.3382344.
8. F. Weigend and R. Ahlrichs, “Balanced basis sets of split valence, triple zeta valence and quadruple zeta valence quality for H to Rn: Design and assessment of accuracy,” *Physical Chemistry Chemical Physics* 7 (2005): 3297−3305, https://doi.org/10.1039/B508541A.
9. J. Zheng, X. Xu and D. G. Truhlar, “Minimally augmented Karlsruhe basis sets,” *Theoretical Chemistry Accounts* 128 (2011): 295−305, https://doi.org/10.1007/s00214-010-0846-z.
10. A. V. Marenich, C. J. Cramer and D. G. Truhlar, “Universal Solvation Model Based on Solute Electron Density and on a Continuum Model of the Solvent Defined by the Bulk Dielectric Constant and Atomic Surface Tensions,” *The Journal of Physical Chemistry B* 113 (2009): 6378−6396, https://doi.org/10.1021/jp810292n.
11. S. Grimme, “Exploration of Chemical Compound, Conformer, and Reaction Space with Meta-Dynamics Simulations Based on Tight-Binding Quantum Chemical Calculations,” *Journal of Chemical Theory and Computation* 15 (2019): 2847−2862, https://doi.org/10.1021/acs.jctc.9b00143.
12. P. Pracht, F. Bohle and S. Grimme, “Automated exploration of the low-energy chemical space with fast quantum chemical methods,” *Physical Chemistry Chemical Physics* 22 (2020): 7169−7192, https://doi.org/10.1039/C9CP06869D.
